# Proteomic Analysis Reveals the Vital Role of Synaptic Plasticity in the Pathogenesis of Temporal Lobe Epilepsy

**DOI:** 10.1155/2022/8511066

**Published:** 2022-07-11

**Authors:** Xu Qian, Ji-Qiang Ding, Xin Zhao, Xin-Wen Sheng, Zhao-Rui Wang, Qi-xing Yang, Jing-Jun Zheng, Jia-Gui Zhong, Teng-Yue Zhang, Shu-Qiao He, Wei-Dong Ji, Wei Li, Mei Zhang

**Affiliations:** ^1^Department of Clinical Pharmacology, Key Laboratory of Molecular Target & Clinical Pharmacology, School of Pharmaceutical Sciences, Guangzhou Medical University, Guangzhou, Guangdong 511436, China; ^2^Department of Neurosurgery, The First Affiliated Hospital of Jinan University, Guangzhou, Guangdong 510632, China; ^3^Center for Translational Medicine, The First Affiliated Hospital, Sun Yat-sen University, Guangzhou, Guangdong 510080, China; ^4^Department of Neurosurgery, The Sixth Affiliated Hospital of Jinan University, Dongguan, Guangdong 523560, China

## Abstract

Temporal lobe epilepsy (TLE) is a chronic neurological disorder that is often resistant to antiepileptic drugs. The pathogenesis of TLE is extremely complicated and remains elusive. Understanding the molecular mechanisms underlying TLE is crucial for its diagnosis and treatment. In the present study, a lithium-pilocarpine-induced TLE model was employed to reveal the pathological changes of hippocampus in rats. Hippocampal samples were taken for proteomic analysis at 2 weeks after the onset of spontaneous seizure (a chronic stage of epileptogenesis). Isobaric tag for relative and absolute quantization (iTRAQ) coupled with liquid chromatography-tandem mass spectrometry (LC–MS/MS) technique was applied for proteomic analysis of hippocampus. A total of 4173 proteins were identified from the hippocampi of epileptic rats and its control, of which 27 differentially expressed proteins (DEPs) were obtained with a fold change > 1.5 and *P* < 0.05. Bioinformatics analysis indicated 27 DEPs were mainly enriched in “regulation of synaptic plasticity and structure” and “calmodulin-dependent protein kinase activity,” which implicate synaptic remodeling may play a vital role in the pathogenesis of TLE. Consequently, the synaptic plasticity-related proteins and synaptic structure were investigated to verify it. It has been demonstrated that CaMKII-*α*, CaMKII-*β*, and GFAP were significant upregulated coincidently with proteomic analysis in the hippocampus of TLE rats. Moreover, the increased dendritic spines and hippocampal sclerosis further proved that synaptic plasticity involves in the development of TLE. The present study may help to understand the molecular mechanisms underlying epileptogenesis and provide a basis for further studies on synaptic plasticity in TLE.

## 1. Introduction

Epilepsy is a devastating and complex neurological disease characterized with spontaneous recurrent seizures (SRS) [1]. It affects approximately 70 million people around the world [2, 3]. Temporal lobe epilepsy (TLE) remains as one of the most severe and common drug-resistant types of focal acquired epilepsies [4]. The pathological mechanism of epilepsy is highly complicated, and its precise understanding is fundamentally important for an early diagnosis, therapy, and prognosis of patients with epilepsy [5]. So far, many studies showed that the pathological mechanism of epilepsy involves abnormal ion channel expression, inflammation, neuronal death, gliosis, and synaptic remodeling [6, 7]. In the development of TLE, the major pathological changes is hippocampus sclerosis, characterized by hippocampus atrophy, neuron loss, and glial cell proliferation, which lead to cognitive and memory impairments [8, 9]. Accompanied with psychological and physical stress, epileptic patients are at increased risk of disability and death [1, 3]. Due to its complicated pathogenesis, it is necessary to find more effective targets for the treatment of epilepsy.

In recent decades, with the rapid development of proteomic-based techniques, iTRAQ has been employed in a large range of studies for the relative quantification of proteins [10–12]. Therefore, analysis of iTRAQ-based quantitative proteome may provide new information pertaining to the progression of TLE. Chronic animal models could reproduce most of characteristics which is homologous with human diseases. Rats and mice represent the common animal species used to reproduce TLE model. Sprague Dawley rats are more frequently employed in the study of epilepsy due to its low mortality [13]. In the present study, we established a chronic rat model of TLE and identified differentially expressed proteins (DEPs) in hippocampus by iTRAQ integrated with LC–MS/MS analysis. The possible biological roles of these DEPs in TLE progression were evaluated by bioinformatics analyses. These findings may provide some new insights into the pathogenesis of TLE.

## 2. Animals and Methods

### 2.1. Animals

Male Sprague Dawley rats (200 ± 20 g) were obtained from Experimental Animal Center of Guangzhou University of Chinese Medicine (SCXK(YUE) 2013-0034). The animals were housed in cages under standard environmental conditions (12 h light-dark circle, temperature 22°C, free access to food and water). This study was carried out in strict accordance with the recommendations in the Guide for the Care and Use of Laboratory Animals published by the United States National Institutes of Health (NIH publication No. 80-23, revised 1996). All protocols were approved by the Animal Research Committee, Guangzhou Medical University, Guangzhou, China.

#### 2.1.1. Temporal Lobe Epilepsy Model

Pilocarpine-induced epilepsy rat model was considered as TLE according to a previous report [13]. Rats were intraperitoneally given lithium chloride (127 mg·kg^−1^, Sigma-Aldrich) and pilocarpine hydrochloride (30 mg·kg^−1^, Sigma-Aldrich). Atropine (1 mg·kg^−1^, King York) was used to reduce the peripheral cholinergic effects. The evoked seizures were scored according to Racine's scale. Only those rats that attained stage IV or V were employed in our experiment. Diazepam (10 mg·kg^−1^, KingYork) was injected to stop seizures 60 min after the onset of status epilepticus (SE). Rats received intensive care including keeping warm and sugar saline oral administration. SE is followed by a latent period and later by the appearance of SRS. The rats were sacrificed at two weeks after the onset of spontaneous seizure, and hippocampi were collected for proteomic analysis. All 8 rats received EEG monitoring and Morris water maze test. For immunohistochemistry analysis and western blotting assay, five rats were included. Three rats were applied to proteomic detection.

#### 2.1.2. Electrode Implantation and Electroencephalogram (EEG) Recording

The rats were anaesthetized with isoflurane, and then, electrodes were implanted by surgery after two weeks of SE induction. The electrodes were fixed to rat's skull at a position of 4.2 mm behind the coronal suture and 2.5 mm from the sagittal suture. Recovery period between surgery and EEG recording lasted for one week. The EEG was recorded in free moving rats for 7 days. The Spike 2 software (CED, UK) was used to monitor and analyze EEG.

#### 2.1.3. Morris Water Maze Test

Morris water maze tests were performed to evaluate learning and memory at one week after the onset of spontaneous seizures in rats. In the acquisition phase, each rat was given four trials per day for five consecutive days, during which rats were allowed to swim to search the underwater platform. In each test, the distance travelled, time reaching to underwater platform, and mean swimming speed were recorded by a computerized video tracking system. After five days of acquisition training, a probe trial was performed on the sixth day. The time spent in the target quadrant was calculated within 120 s.

#### 2.1.4. Nissl Staining and Immunofluorescence Analysis

Rats were deeply anaesthetized using 1.5% isoflurane, and the thoracic cavity was opened rapidly to expose the heart. A catheter was inserted into the ascending aorta, and rats were perfused with heparin saline, followed by cold 4% paraformaldehyde (PFA). The brains were removed and postfixed in 4% PFA overnight at 4°C. Brains were dehydrated, transparentized, and embedded in paraffin. Serial coronal sections containing hippocampus were cut and collected sequentially.

Nissl staining was employed to assess hippocampus formation. Briefly, paraffin sections were deparaffinized, rehydrated, and stained with 1% toluidine blue. Every 15th staining section was chosen for quantitative analysis (five sections per rat). Images of CA1, CA3, and hilus of hippocampus sections were obtained with 40x magnification. The neurons were measured and quantified by using ImageJ/NIH image analysis system.

Immunohistochemical analysis was carried out according to standard protocol. Hippocampal slices were incubated overnight with NeuN primary antibody (mouse mAb, 1 : 100, CST) and GFAP primary antibody (rabbit mAb, 1 : 200, CST) at 4°C. After washed, the slices were incubated with IgG-alexa fluor-555 (goat anti-mouse, 1 : 1000, CST) and IgG-alexa fluor-647 (goat anti-rabbit, 1 : 1000, CST) in the dark for 2 hours. Subsequently, the DAPI dye solution (CST) was used to stain nucleus for 10 min. The slices were observed with confocal microscope, and images of CA1, CA3, and hilus of hippocampus sections were obtained with 40x magnification.

#### 2.1.5. Golgi Staining

Golgi staining was used to analyze the structure of dendritic spines according to our previous research [14]. The brains were fixed and embedded, and hippocampal sections were cut into 100 *μ*m thick and mounted with glycerin gelatin. Images of hippocampal sections were obtained via a digital slice scanner. There is evidence that terminal branches are more plastic than nonterminal branches, so numbers of terminal branches of dendrite were used to evaluate the localized dendritic remodeling [15]. Dendritic segments (25 *μ*m in length each) of 10 neurons per animal were selected in the region of dentate gyrus for counting numbers of dendritic spines. Densities of dendritic spines were expressed as spines per unit length.

#### 2.1.6. Western Blotting

The hippocampal samples were separated using 10% SDS-PAGE and transferred to PVDF membranes. Then, membranes were probed with mouse anti-CaMKII-*α* and II-*β* (1 : 1000, CST), rabbit anti-GFAP (1 : 1000, CST), rabbit anti-F-actin (1 : 1000, CST), and mouse anti-GAPDH (1 : 10000, ZSGB-BIO) at 4°C overnight. After washed with TBST, the membranes were incubated with goat anti-rabbit (1 : 3000, CST) for 1 h at room temperature. Blots were developed using a chemiluminescent substrate and analyzed with the Quantity One software (version 4.4).

### 2.2. iTRAQ-8 Plex Labeling and Mass Spectrometry

Proteolysis of hippocampus was in accordance with the FASP method [16]. The protein samples were treated with iTRAQ lysis buffer, and then, DTT and iodoacetamide were added for reduction and alkylation of proteins. The protein samples were washed with TEAB buffer for two times and then digested with trypsin at a dilution of 1 : 50 for 16 h. The pooled protein samples were labeled using iTRAQ reagent 8-plexkit according to the manufacturer's protocol (AB SCIEX). The iTRAQ-labeled protein samples were separated by dual coordination reverse chromatographic column (ZORBAX Extended-C18, 2.1 × 50 mm, 5 *μ*m) with Agilent 1100 HPLC (USA). The mobile phase is composed of buffer A (10 mM ammonium formate, 5% acetonitrile in water, and pH 10) and buffer B (10 mM ammonium formate, 90% acetonitrile aqueous solution, and pH 10). The elusion was performed at 2-40% linear gradient within 55 minutes. The flow rate of column was maintained at 0.3 mL/min, and the detection wavelength was set at 215 nm. The effluents were collected every 1 minute within the gradient range. Subsequently, separated peptides were further analyzed using Eksigent nanoLC-Ultra™ liquid chromatography and Triple TOF LC-MS/MS 5600 plus Mass Spectrometer (SCIEX, USA).

### 2.3. Proteomic Data Analysis and Differentially Expressed Protein Identification

The tandem mass spectra map was processed using SCIEX's Protein Pilot software (v4.5), and data was searched in the Rattus norvegicus protein database. The FASTA files of all Rattus norvegicus protein data were downloaded from UniProt database (https://www.uniprot.org/, April 2017), including 37,686 protein entries. The following criteria were used for the identification and quantification of proteins: false positive rate (FPR) was set at 1%; the error of the first-order mass spectrum was 20 PPM, and the error of the second-order mass spectrum was 0.1 Da. Quality control of data was evaluated by the Protein Pilot Descriptive Statistics Template (PDST) 3.0 software (SCIEX, USA). Labelling efficiency of iTRAQ 8 plex@N-term and iTRAQ 8plex (K) was 97.55% and 99.97%, respectively. The missed cleavages for single site and dual sites were 11.3% and 0.3%, respectively. The mass accuracy of parent ion was within 5 ppm. Target-decoy search strategy and Bonferroni method were used to control global FPR. Only protein differences with *P* value < 0.05 and fold change > 1.5 (or <0.67), which corresponded to 99% of confidence, were considered as differential expression. Volcano plot, principal components analysis, and hierarchical cluster analysis were performed with software PDST 3.0.

### 2.4. Bioinformatics Analysis

The OmicsBean software was used to conduct the enrichment analysis of DEPs by which gene ontology (GO) and Kyoto encyclopedia of genes and genomes (KEGG) pathway were analyzed. The query proteins were conducted for blast P search before enrichment analysis. Quick GO database was used to annotate the GO classification, which consisted of biological processes (BP), molecular functions (MF), and cellular components (CC). *P* value < 0.05 and gene count ≥ 2.0 were set as the threshold of significance. For KEGG pathway enrichment analysis, the information of DEPs was mapped into KEGG database to obtain the enriched pathway. A protein-protein interaction (PPI) and pathway-protein network were constructed by STRING database. The minimum required PPI score was set at a 90% of confidence.

### 2.5. Statistical Analyses

Behavioral score, immunohistochemistry, and western blotting analysis are expressed as mean ± SD. The differences between two groups were evaluated by Student's *t*-test using the GraphPad Prism software. Differences were considered statistically significant at *P* < 0.05. Bonferroni correction was applied to control the global FPR in proteomic analysis, and Benjamini method was used to compare the significance of DEPs in bioinformatics analysis.

## 3. Results

### 3.1. Behavioral and Pathological Changes in TLE Rats

#### 3.1.1. Features of SRS and EEG in TLE Rats

**Figure 1:**
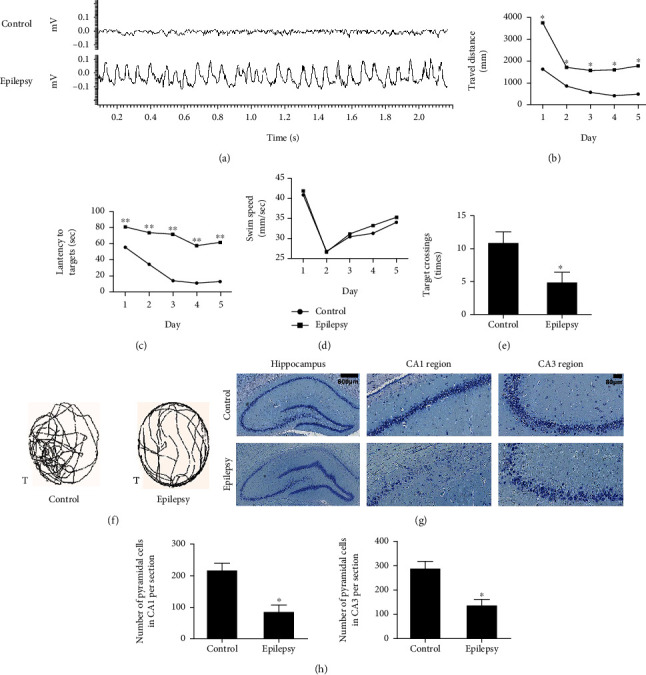
The behavioral changes and hippocampal injury in TLE rats. (a) Manifestation of EEG in rats. (b–f) Morris water maze test. (b) Swim path length; (c) latency to mount the underwater platform; (d) mean swim speed. E: the number of crossings of the target quadrant; F: the tracks of searching for the target quadrant in the probe trial (T: target quadrant). ^∗^*P* < 0.05 compared to control group; ^∗∗^*P* < 0.01 compared to control group (means ± SD, *n* = 8). (g, h) Nissl staining of hippocampus. (g) Formation of hippocampus, CA1 and CA3 regions; (h) quantitative analysis of pyramidal neurons in CA1 and CA3 regions. ^∗^*P* < 0.05 compared to control group (means ± SD, *n* = 5). *n* refers to the numbers of rats.

Rat TLE model was validated with EEG monitoring. SRS occurred at an average of 12 days after SE induction in TLE rats, which is in agreement with our previous studies [14, 17]. Frequency and duration of SRS in TLE rats are shown in Table 1. The occurrence of SRS was accompanied with abnormal electroencephalographic signs, during which TLE rats manifested a generalized clonic-tonic (stages 4-5) seizures associated with the loss of postural control. The EEG of TLE rats showed as high amplitude and frequency paroxysmal discharges (Figure 1(a)). This shows rat TLE model is reliable for the subsequent study.

#### 3.1.2. A Deficit of Cognitive Function in TLE Rats

The impact on behavior was evaluated since cognitive function is known to be altered in TLE. Learning and memory in epileptic rats were assessed using the Morris water maze. The travel distance, latency to target, and mean swimming speed for 5 training days were recorded (Figures 1(b)–1(d)). Epileptic rats exhibited reduced learning in searching for the underwater platform, which was indicated as the increased latency to target and path length during the training trials. No difference in swimming speed was found between TLE rats and controls in the five training days (Figure 1(d)). These results demonstrated an impaired spatial memory in epileptic rats that is not due to motor defect. Epileptic rats spent less time in the target quadrant in probe trial, and the number of crossings of the former platform was reduced (Figures 1(e) and 1(f)). These results indicate that there is a cognitive deficit in TLE rats.

#### 3.1.3. Hippocampal Injuries in TLE Rats

Hippocampal neuronal injury is the primary neuropathological feature of TLE. In order to observe the changes, Nissl staining was used to examine neuronal damage in epileptic rats. Our results revealed that pyramidal neurons were arranged regularly and exhibited structural integrity with clear nucleoli in the CA1 and CA3 regions of the hippocampus of control rats (Figure 1(g)). The CA1 regions of epileptic rats exhibited destruction of the layered structure of pyramidal neurons and an evident neuronal loss. The CA3 regions also exhibited neuronal loss, a disordered arrangement of pyramidal neurons with an irregular shape, opaque cytoplasm, and shrunken nuclei. These results show hippocampal injuries occur during epileptogenesis in TLE rats.

### 3.2. Analysis of Quantitative Proteomics by iTRAQ Coupled with LC–MS/MS

**Figure 2:**
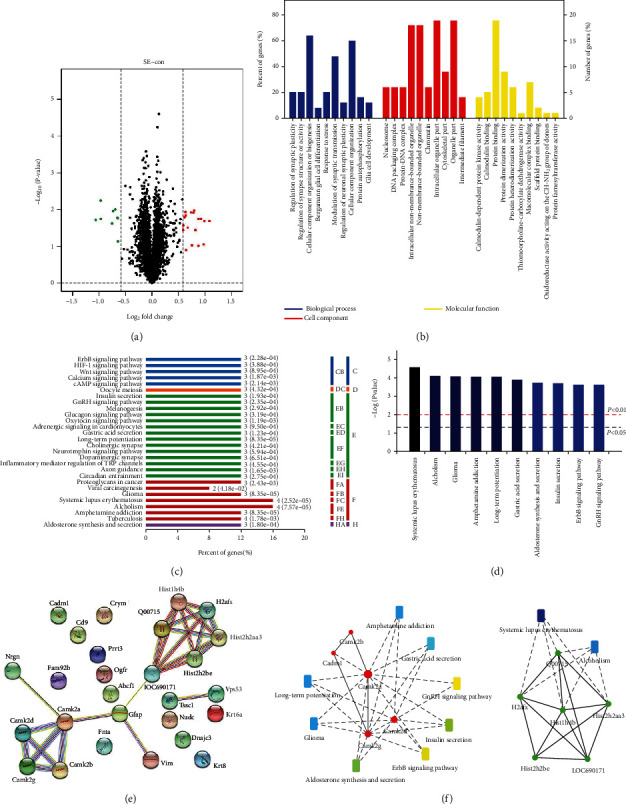
Bioinformatics analysis of hippocampus and functional annotation of DEPs. (a) Volcano plot of 4173 proteins. *X*-axis represents the fold change of DEPs (log 2), and the *Y*-axis indicates the *P* value (−log 10). Red points and green points represent upregulated and downregulated proteins with significant difference, respectively. (b–d) Functional annotation of 27 DEPs based on bioinformatics analysis. (b) The identified 27 DEPs were annotated into biological process, cellular component, and molecular function for GO term analysis. Entries in each category were listed according to *P* value (*P* value increased gradually from left to right.); (c) the 28 pathways enriched by KEGG analysis on 27 DEPs. (d) the top 10 pathways with significant differences by KEGG analysis; (e) protein-protein interaction network of 27 DEPs; (f) KEGG pathway-protein network analysis.

The rat TLE model has been validated as showing the characteristic signs of TLE. The cellular differences during epileptogenesis will be investigated in the following study. iTRAQ proteomic analysis was used to gain insight into the molecular mechanisms of pathogenesis of TLE rats, by which a total of 4173 proteins were identified. Hierarchical clustering analysis revealed the data between TLE rats and control rats could be completely distinguished (shown in supplementary Fig S1). The heatmaps gave a visualized change in abundance of 4173 proteins (shown in supplementary Fig S2). 27 of 4173 proteins were differentially expressed (*P* value < 0.05 and fold change > 1.5 or <0.67), including 18 upregulated and 9 downregulated proteins (Figure 2(a)). Among 27 DEPs with the pronounced differences were GFAP and CaMK family (CaMKII-*α*, II-*β*, and II-*γ*). Detailed information of 27 DEPs is listed in Table 2. Additionally, the data set of 4173 proteins and the fold change and *P* value of each protein were provided as supplementary data (shown in Tab S1).

### 3.3. Bioinformatics Analysis of DEPs in Hippocampus

Bioinformatics analysis was used to analyze the DEPs, which were categorized according to GO terms and KEGG pathway. Enriched proteins count mainly concentrated in biological process based on GO terms and KEGG pathway analysis (Figures 2(b)–2(d)). For the further analysis of GO terms, the 27 DEPs were assigned for classification and annotation according to BP, MF, and CC. The diagram of enrichment analysis showed the top 10 items with significance in BP, MF, and CC, respectively (Figure 2(b)). From the BP perspective, the top ten items with significant differences were “regulation of synaptic plasticity,” “regulation of synaptic structure,” “cellular component organization or biogenesis,” and so on. In terms of MF, “calmodulin-dependent protein kinase activity,” “calmodulin binding,” and “protein binding” were the high rank items. As for CC, the most enriched items included “nucleosome,” “DNA packaging complex,” and “protein-DNA complex.”

KEGG pathways analysis revealed that 28 pathways including “ErbB signaling pathway,” “HIF-1 signaling pathway,” “Wnt signaling pathway,” “calcium signaling pathway,” and “cAMP signaling pathway” were related to epileptogenesis (Figure 2(c)). Among the top 10 pathways “systemic lupus erythematosus,” “alcoholism,” “glioma,” “amphetamine addiction,” and “long-term potentiation” manifested a significant difference (Figure 2(d)).

27 DEPs and the top ten KEGG pathways were mapped to STRING database for the analysis of PPI and pathway-protein interaction, respectively. In the PPI network constructed by 27 DEPs, there was a strong linkage among the members of CaMKII family besides the members of histone family (Figure 2(e)). In addition, GFAP showed a link with CaMKII-*α* and histone H3. KEGG pathway-protein network was enriched into two distinct modules. The proteins interacted with “systemic lupus erythematosus” and “alcoholism” pathway centralized in histone family, while the proteins interacted with the other 8 KEGG pathways such as “ErbB signaling pathway” and “long-term potentiation” centralized in CaMKII family (Figure 2(f)).

### 3.4. Validation of iTRAQ-Based Proteomic Results for Selected DEPs

**Figure 3:**
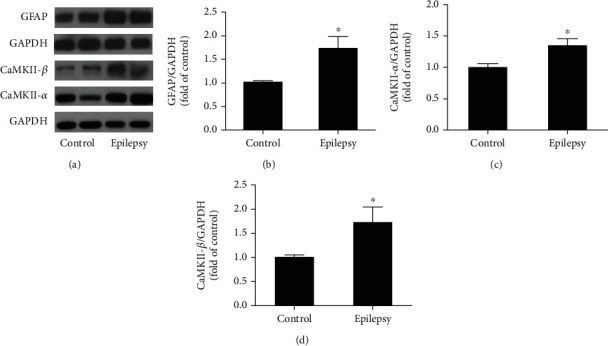
The validation of the pronounced DEPs in the hippocampus of TLE rats. (a) The expression of CaMKII-*α*, II-*β*, and GFAP in hippocampus; (b–d) the quantitative analysis of CaMKII-*α*, II-*β*, and GFAP. ^∗^*P* < 0.05, control vs. epilepsy (mean ± SD, *n* = 5). *n* refers to the numbers of rats.

Proteomic analysis revealed that the most significantly altered proteins were GFAP and the members of CaMKII family, highlighting their implication in epileptogenesis. Therefore, to further confirm this, we verified the expression of GFAP, CaMKII-*α*, and II-*β* in the hippocampus of TLE rats. Western blotting was used as the method of validation, and blots were the representative of 5 replicates. It has been revealed that the increased expression of GFAP, CaMKII-*α*, and II-*β* was detected in whole lysates, consistent with the upregulation detected in the proteomic analysis (Figures 3(a)–3(d)). These results also demonstrated a good reliability and validity of the proteomic analysis with iTRAQ.

### 3.5. Changes of Synaptic Structure and Gliosis in the Hippocampus of TLE Rats

**Figure 4:**
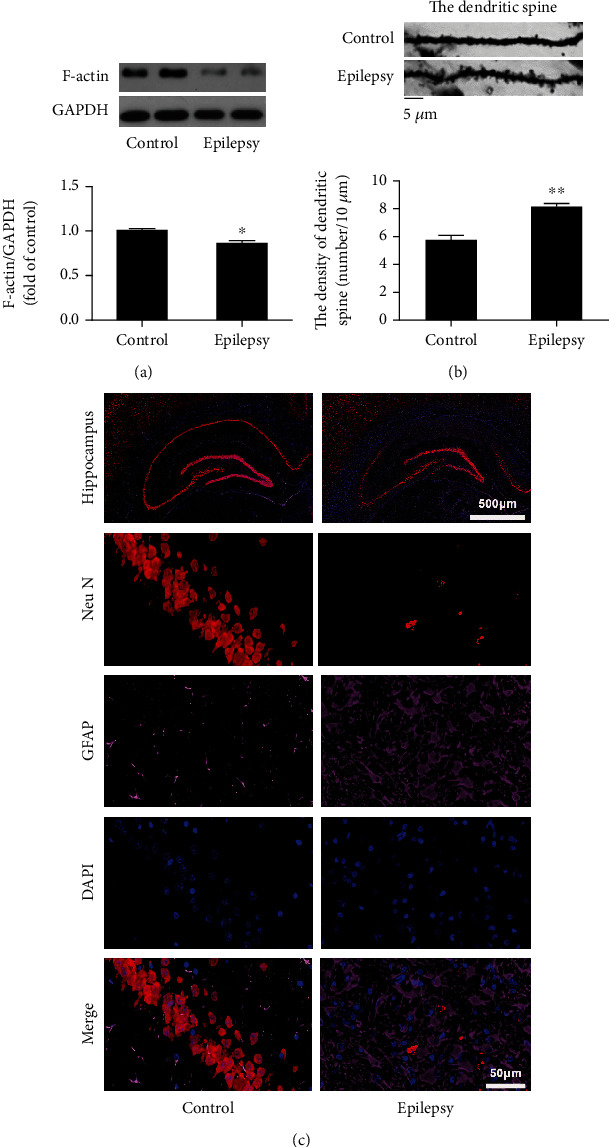
Increased dendritic spines and gliosis in the hippocampus of TLE rats. (a) The expression and quantitative analysis of F-actin in hippocampus; (b) the density of dendritic spines in the dentate gyrus of hippocampus; (c) the expression of GFAP and NeuN in the CA1 region of hippocampus. ^∗^*P* < 0.05, control vs. epilepsy (mean ± SD, *n* = 5). *n* refers to the numbers of rats.

Bioinformatics analysis indicated that the regulation of synaptic plasticity is the most important biological process, implying its vital role in the pathogenesis of TLE. F-Actin is the major cytoskeletal protein in spine, and the structural modification of dendritic spines plays a critical role in synaptic plasticity [18]. In order to determine the alteration of synaptic structure, the densities of dendritic spines and F-actin were observed in the hippocampus of epileptic rats. Increased dendritic spines and decreased expression of F-actin indicated synaptic remodeling occurred in the hippocampus of epileptic rats (Figures 4(a) and 4(b)). These results further demonstrated synaptic plasticity involves the pathogenesis of TLE.

Hippocampus sclerosis is the major pathological feature of TLE, in which gliosis may contribute to its development. In present experiment, both proteomic analysis and validation of protein expression revealed that the level of GFAP increased in the hippocampus of TLE rats. In order to observe the alteration of hippocampal structure, immunohistochemical stain was conducted to detect the manifestation of GFAP and gliosis in TLE rats. It has been demonstrated an upregulated expression of GFAP in the CA1 region of hippocampus was accompanied with gliosis and neuronal loss (Figure 4(c)).

## 4. Discussion

In the present study, iTRAQ proteomic analysis was employed to investigate the underlying mechanisms of epileptogenesis in rat TLE model. Abnormal EEG, cognitive and memory impairments, and hippocampal damage have been shown in epileptic rat, which is in accordance with the known pattern of TLE, thereby confirming that the TLE model in our present study is reliable for the subsequent investigation. iTRAQ coupled with LC-MS/MS proteomic technique could profile the protein patterns and identify the variation of protein expression in various disease states, which has been widely used to analyze biological samples [10–12]. Hippocampus is the primary site in the epileptogenesis of TLE. Therefore, proteomic analysis of hippocampus will be helpful to reveal the potential mechanisms of epileptogenesis.

With iTRAQ-based quantitative proteomic analysis, 4173 proteins were identified from the hippocampus of TLE rats, of which 27 DEPs with the significant difference were obtained through the filtration of fold change and *P* value. The most pronounced proteins were GFAP and CaMKII family, suggesting their implication in epileptogenesis. According to GO analysis of 27 DEPs, the annotation of biological processes has revealed that the top 10 items mainly involved in the regulation of synaptic plasticity and structure, cellular component organization, and glia cell differentiation. As far as molecular function was concerned, CaMKII family is the most significant molecule, which is in correspondence with the proteomic analysis. For the analysis of cell component, cytoskeletal part ranked in the top ten items, implying its essential role in the pathogenesis of TLE. Briefly, GO term analysis demonstrated that synaptic plasticity is the major biological processes of epileptogenesis, and CaMKII family and cytoskeletal components involve in the pathogenesis of TLE.

KEGG pathways analysis of 27 DEPs demonstrated that 28 pathways are related with epileptogenesis, of which “glioma” and “long-term potentiation” were included in the top ten items. Glioma in the present circumstance means that the abnormal proliferation of glia cell occurs in the hippocampus of TLE rats. Similarly, glia cell differentiation was identified to be the important event during epileptogenesis according to GO analysis. Long-term potentiation is an important form of synaptic plasticity [19], which is in accordance with the results of GO analysis, by which synaptic plasticity was proved to be the major biological processes involving epileptogenesis. Noteworthy, “systemic lupus erythematosus” manifested a significant difference by KEGG analysis. Recently, it has been reported that there is a high prevalence of epilepsy in systemic lupus erythematosus (SLE) patients [20, 21], implying the linkage between epilepsy and SLE, which also account for why “systemic lupus erythematosus” was identified as the most significant KEGG pathway in the present study. PPI analysis indicated that there is a strong internal linkage among the members of CaMKII family. Moreover, 8 of the top ten KEGG pathways uniformly showed an interaction within CaMKII family. Thus, it can be inferred that CaMKII family serves as vital molecules during epileptogenesis.

A variety of research has explored the pathogenesis of epilepsy based on proteomic analysis with different epileptic model and samples. Studies on epileptic patients in multiple brain regions have shown a dysregulation in pathways associated with mitochondrial function, protein synthesis, synaptic transmission, and remodeling of the neuronal network architecture [22]. Investigation in mesial temporal lobe epilepsy (MTLE) patients with or without hippocampal sclerosis (HS) has revealed that there is a remarkable alteration of synaptic proteins and glia-associated proteins [23, 24]. Additionally, proteomic analysis of sclerotic hippocampus revealed the packaging of vesicular neurotransmitters is altered in TLE patients [25]. Bioinformatics analysis of dentate gyrus proteome in TLE-HS patients indicated that DEPs are enriched in synaptic vesicle, mitochondrion, cell-cell adhesion, and regulation of synaptic plasticity [26]. In addition, proteomic investigation of MTLE patients with granule cell dispersion found that the upregulated proteins are involved in the developmental cellular migratory processes, including cytoskeletal remodeling and axon guidance [27]. Analysis of plasma from children with rolandic and refractory epilepsy demonstrated that immune or inflammatory response may play an important role in the development of epilepsy [28, 29]. Taken together, proteomic analysis from patients indicates that there are some common changes with synapse, mitochondrion, and glia cell during epileptogenesis.

Studies on epileptic animal model have shown the differences by proteomic analysis. DEPs are focused on the modulators of disease-associated inflammatory signaling at different stages of epiletogenesis in electrically induced rat model [30]. Similarly, proteins associated with gliosis and inflammation show a prominent change in a mouse MTLE model [31]. In an immature rat TLE model, proteomic screen shows an abnormal alteration of synaptic-related proteins, such as synapsin-1, dynamin-1, and neurogranin [24]. Additionally, multiomics analysis shows a pronounced upregulation of GFAP and vimentin in TLE rat [32]. Inconsistency with the studies mentioned above, proteins linked with cell stress, cellular plasticity, ubiquitin ligase complex or metabolite homeostasis, and Ca^2+^ regulatory network show an evident alterations with different epileptic animal model [33–35]. A comprehensive analysis of proteome has been done in which 8 modules of interconnected protein groups reflecting distinct molecular aspects of epileptogenesis are identified in hippocampus and the enriched pathways include Rho family signaling, HIPPO signaling, 14-3-3 family signaling, chemokine signaling, and axonal guidance signaling [36]. Although there are differences in the experimental design and characteristics of the models, including animal species, kindled methods, and analytic techniques, some common results such as inflammatory response and gliosis still have been obtained by proteomic analysis.

In the present study, proteomic analysis has shown that the pronounced proteins were GFAP and CaMKII family. Furthermore, bioinformatics analysis revealed that synaptic plasticity is the major biological processes, and CaMKII is a vital molecular during epileptogenesis. On the basis of this, we make a further validation on the proteins involving in gliosis and synaptic plasticity, including GFAP, the members of CaMKII and F-actin. The functional role of GFAP in astrocytes has been widely accepted, which is involved in the process of regeneration, synaptic remodeling, and reactive gliosis [37, 38]. Hippocampal sclerosis is the most common feature in TLE, which is due to the proliferation and hypertrophy of astrocytes resulted from increased glia-associated proteins [39, 40]. An increased GFAP and typical gliosis have been observed within the hippocampus in our present research, which further consolidate the insight that increased GFAP is involved in hippocampal gliosis and epileptogenesis.

CaMKII is the major postsynaptic protein at excitatory synapses, which is fundamentally important for synaptic plasticity [41]. The activity of CaMKII-*α* is essential for synaptic plasticity [42], whereas the primary function of CaMKII-*β*, a structural subunit, possesses F-actin binding and bundling properties, providing essential functions for synapse formation and dendritic morphology [43]. In the present study, GO analysis revealed that CaMKII family, cytoskeletal components, and synaptic plasticity were involved in the pathogenesis of TLE. As the major cytoskeletal protein in spine, F-actin's stabilization depends on CaMKII activity [44]. Therefore, the expression of CaMKII-*α* and II-*β* and F-actin and the alteration of synaptic structure have been detected in the hippocampus of TLE rats. The upregulated expression of CaMKII-*α*, CaMKII-*β*, and F-actin and the increased density of dendritic spine suggest a potential link between CaMKII and synaptic remodeling within hippocampus. A significant upregulation of CaMKII has been observed in the hippocampus of TLE patients and different epileptic models [41, 45, 46], whereas upregulation of CaMKII is not consistent with an increased activity [41, 46]. The reasons for this consistence remain to be elucidated. It should be noted that CaMKII has not been reported as DEP by proteomic analysis in TLE except a research on Dravet syndrome in mouse model, in which CaMKII presented as a downregulated protein [47]. Despite the implication in the etiology of seizure activity, further studies are required to determine a causal link between CaMKII and synaptic remodeling as well as the therapeutic potential of CaMKII inhibitors for the treatment of epilepsy.

In conclusion, our study demonstrated that GFAP, CaMKII-*α* and II-*β*, and synaptic plasticity were involved in the pathogenesis of TLE by proteomic analysis and further investigation provided an anatomical evidence for the role of synaptic plasticity during epileptogenesis. However, it must be mentioned that iTRAQ-based proteomic analysis has the advantages in both high throughput and the precision in the abundance measurements, whereas the accuracy of the estimations of differential expression may be affected due to ratio compression [48]. As a result, the identification of relative few differential proteins by iTRAQ technique in our present study may limit a comprehensive understanding of the mechanism underlying epileptogenesis.

## Figures and Tables

**Table 1:** Characteristics of SRS in TLE rats (mean ± SD, *n* = 8).

Groups	Seizure/week (times)	Seizure score	Seizure duration
Control	\	\	\
Epilepsy	5.42 ± 1.68	IV/V	IV/V

**Table 2:** Information of the 27 DEPs based on iTRAQ proteomics analysis.

Swiss-Prot accession number	Gene	Changes	Fold change	*P* value	Other studies		References
					Human	Animal model	
P47819	GFAP	Up	1.83	0.013	☑up	☑up	[24, 30–33, 35, 47]
P11275	Camk2a	Up	1.90	0.018	□	☑down	[47]
F1LUE2	Camk2b	Up	1.74	0.011	□	□	
P11730	Camk2g	Up	1.67	0.012	□	□	
P15791	Camk2d	Up	1.72	0.012	□	□	
Q63525	Nudc	Up	1.84	0.018	□	□	
Q6AYP5	Cadm1	Up	1.55	0.012	□	□	
Q10758	Krt8	Up	1.69	0.093	□	□	
P40241	Cd9	Up	1.53	0.016	□	☑up	[31, 33]
Q6MG08	Abcf1	Up	1.99	0.021	□	□	
Q5RKJ4	Fnta	Up	1.50	0.036	□	□	
G3V8C3	Vim	Up	1.56	0.127	☑up	☑up	[31–33, 47]
Q9R0T3	Dnajc3	Up	2.12	0.020	□	□	
Q4FZU2	Krt6a	Up	1.97	0.088	□	□	
B4F763	Vps53	Up	1.56	0.014	□	□	
Q3MID9	Ogfr	Up	1.51	0.027	□	□	
F1LNX7	Tssc1	Up	1.60	0.031	□	□	
D3ZCB9	Fam92b	Up	1.77	0.036	□	□	
Q04940	Nrgn	Down	0.64	0.017	☑down	☑down	[24]
P62804	Hist1h4b	Down	0.51	0.006	□	□	
Q00715	Q00715	Down	0.62	0.010	□	□	
D3ZXP3	H2afx	Down	0.48	0.019	□	□	
M0RBX6	LOC690171	Down	0.59	0.024	□	□	
P0CC09	Hist2h2aa3	Down	0.51	0.018	□	□	
D4A817	Hist2h2be	Down	0.60	0.011	□	□	
Q9QYU4	Crym	Down	0.64	0.074	□	□	
D3ZWQ0	Prrt3	Down	0.64	0.017	□	□	

## Data Availability

The data that support the findings of this study are available from the corresponding author upon reasonable request.

## Supplementary Materials

Supplementary Materials Tab S1: the data set of 4173 proteins. Figure S1: the principal component analysis (PCA) and quantitative protein cluster analysis. Figure S2: the heatmap clustering analysis of 27 DEPs. Figure S3: the enriched protein counts by GO term and KEGG pathway analysis of 4173 proteins. Figure S4: an original mass spectrometry data (uploaded to Proteome X change Datasets). Figure S5: original western blotting images: (1) GFAP and GAPDH; (2) CaMKII-*α* and GAPDH; (3) CaMKII-*β* and GAPDH; (4) F-actin and GAPDH.
