# Supplementary material for: Proteomic Analysis Reveals the Vital Role of Synaptic Plasticity in the Pathogenesis of Temporal Lobe Epilepsy
Source: Neural Plast. 2022 Jul 11;2022:8511066. doi: 10.1155/2022/8511066 (PMC9293557; doi:10.1155/2022/8511066)

SUPPLEMENTARY MATERIAL

**Supplementary Figures**	**Pages 2-5**
**Original western blotting images**	**Pages 6-9**

Supplementary Figures

Figure S1. Theprincipal component analysis（PCA）andquantitative protein cluster analysis. 113, 114 and 115: control group(con), 116, 117and 118:epilepsy group(SE). The control group can be completely distinguished from the epilepsy group.

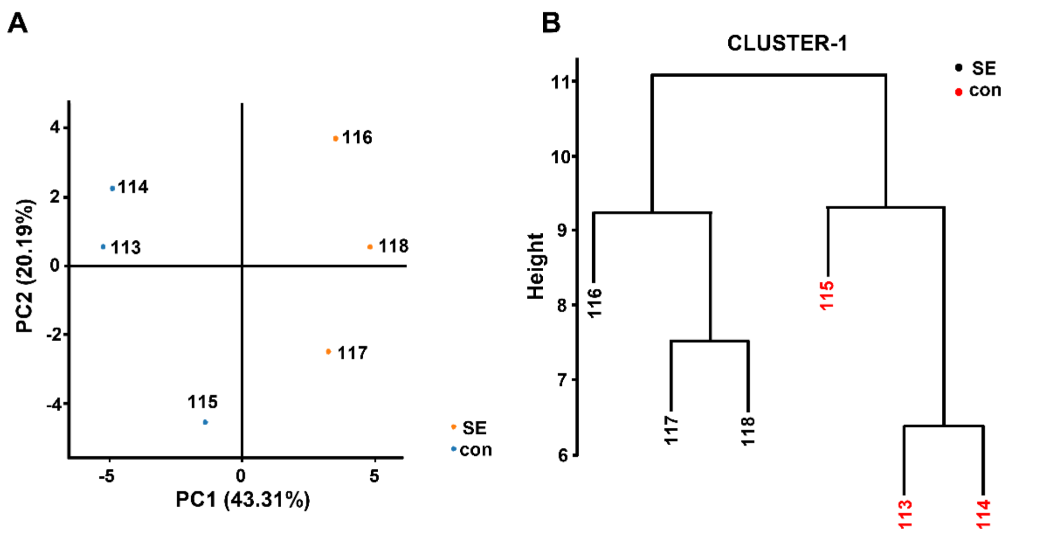

Figure S2. The heatmap clustering analysisof 27 DEPs. 113, 114 and 115: control group(con), 116,117and 118:epilepsy group(SE). The red color represents upregulated DEPs, and the green color represents downregulated proteins.

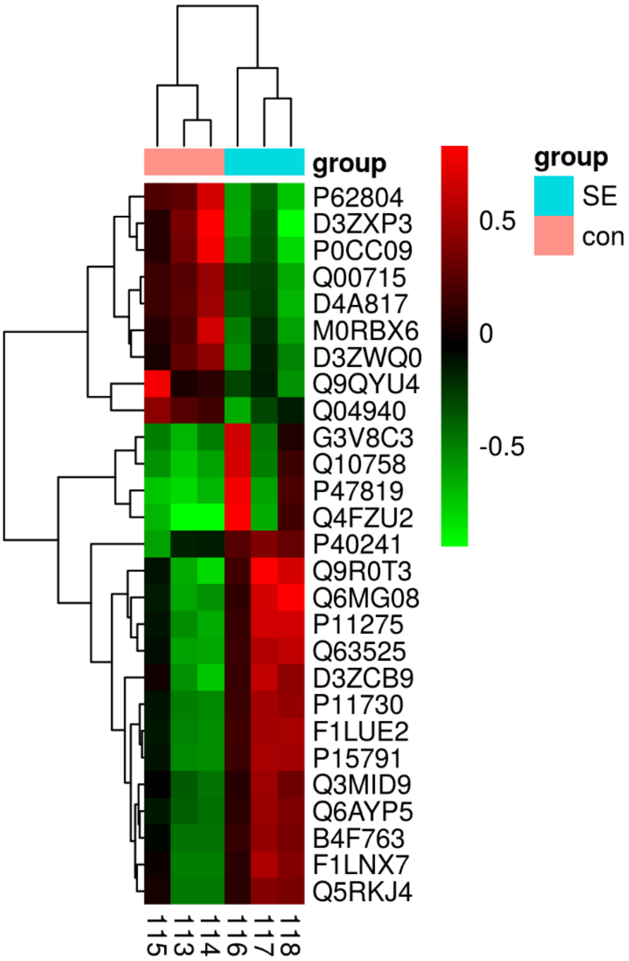

Figure S3.Theenriched protein counts by GO term and KEGG pathway analysis of 4173 proteins.

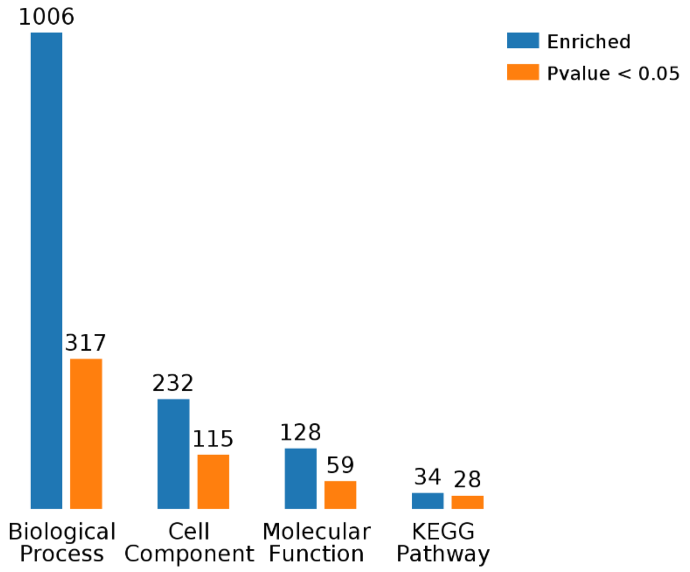

Figure S4.PXD 003481 is an original mass spectrometry data,which has been uploaded to *ProteomeXchangeDatasets*.

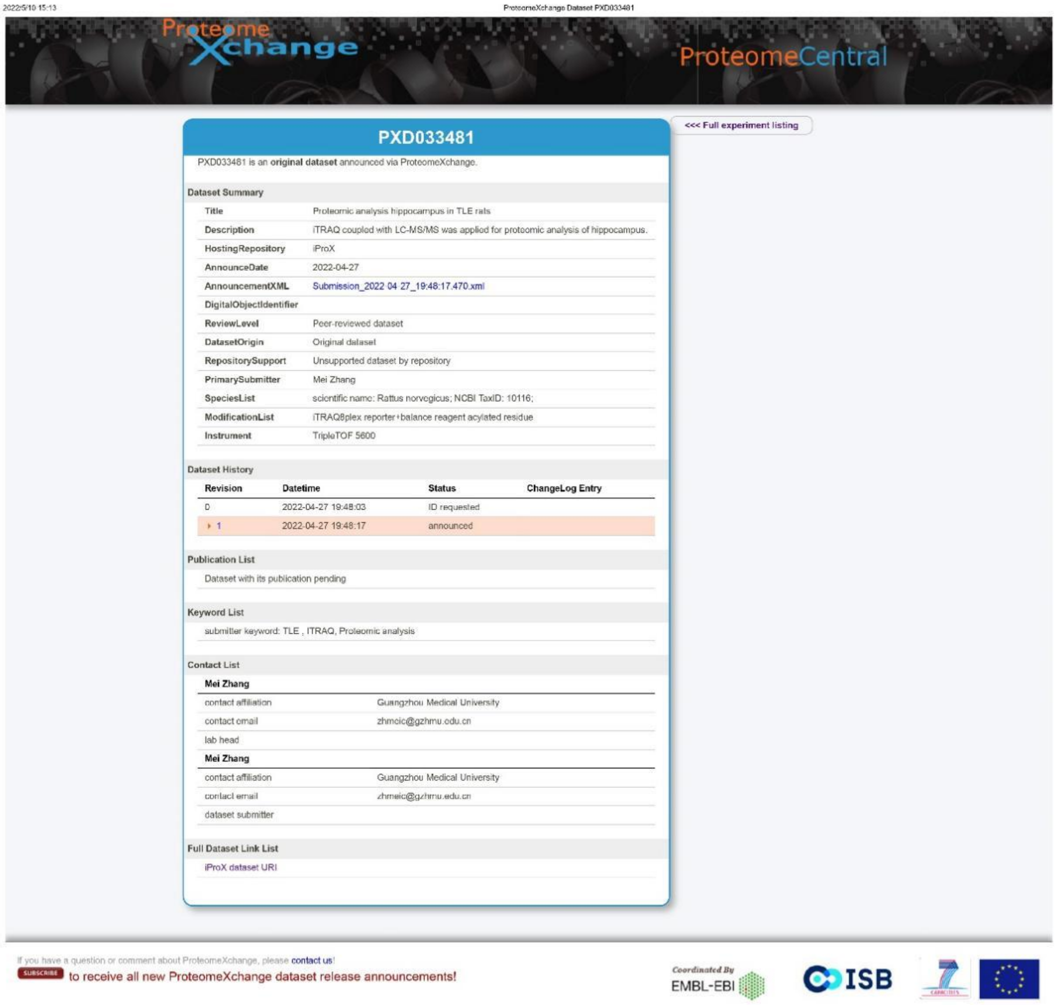

Figure S5.Original western blotting images.

(1)GFAP and GAPDH

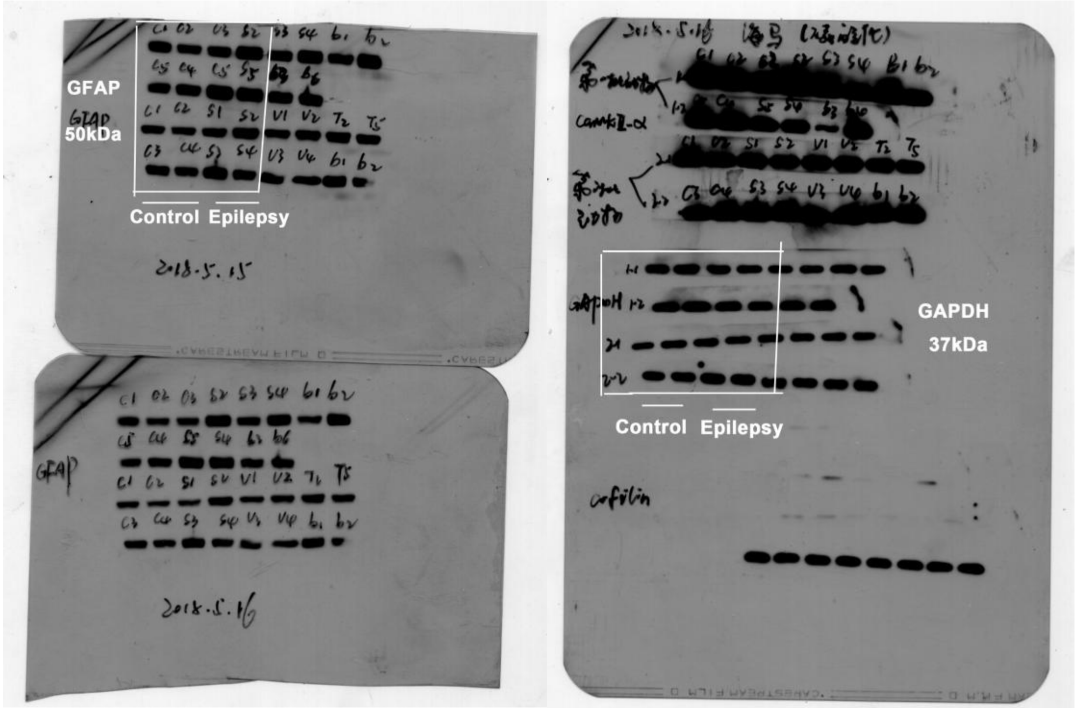

(2) CaMKII-α and GAPDH

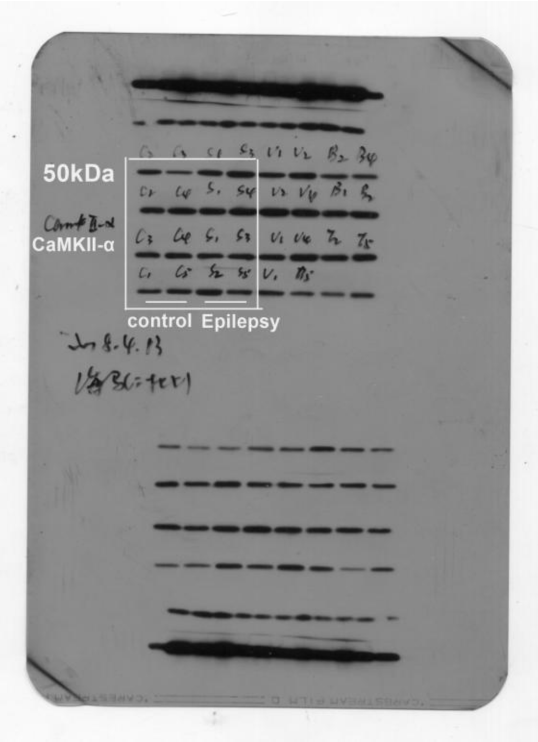

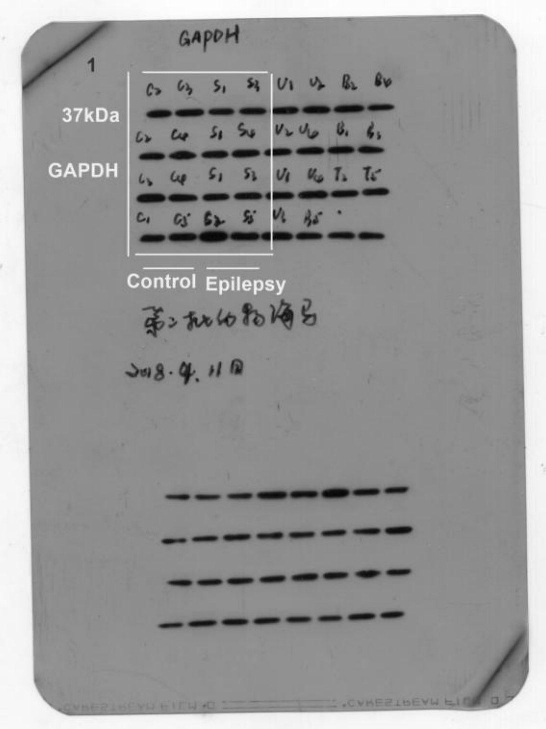

(3) CaMKII-β and GAPDH

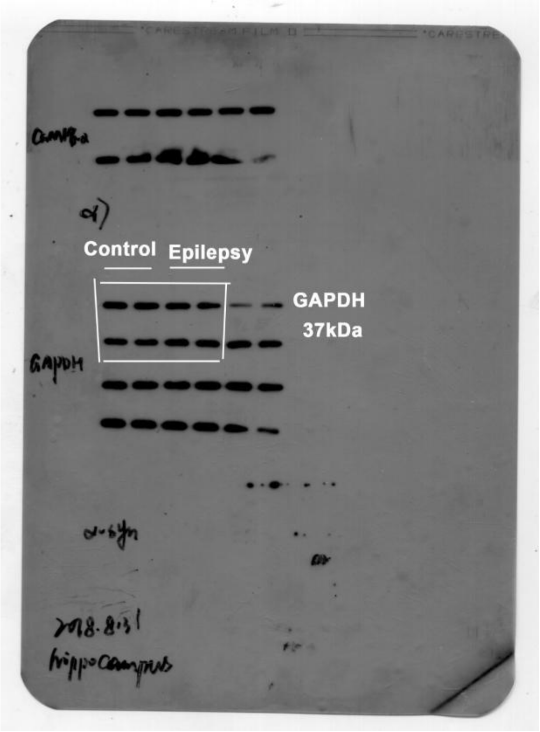

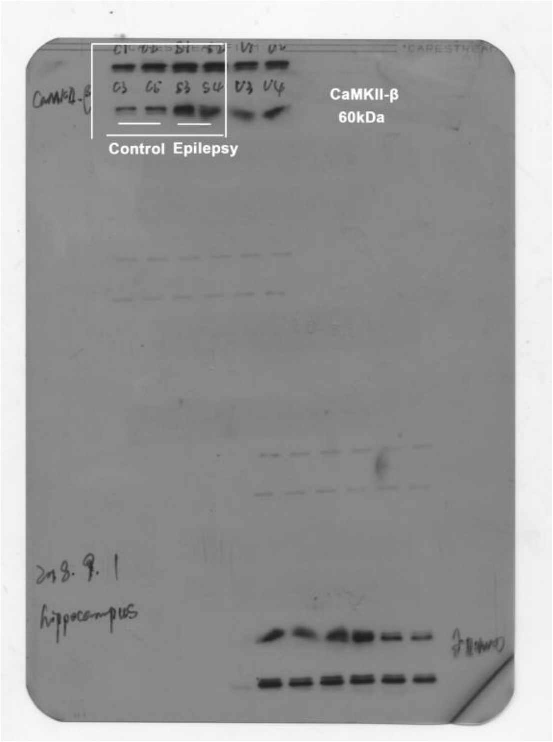

(4) F-actinand GAPDH

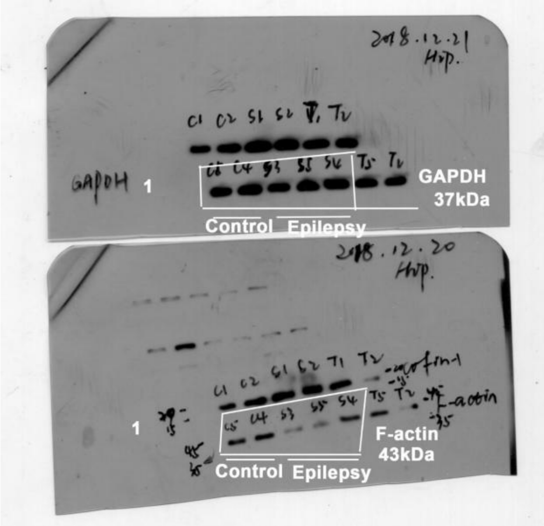

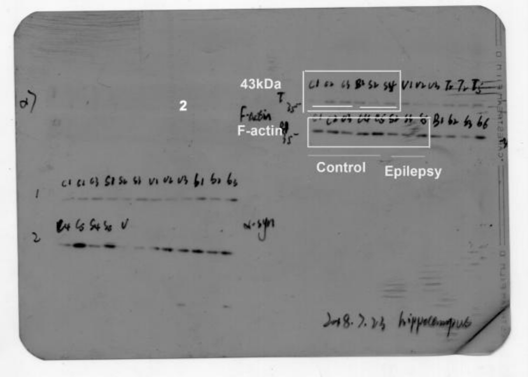

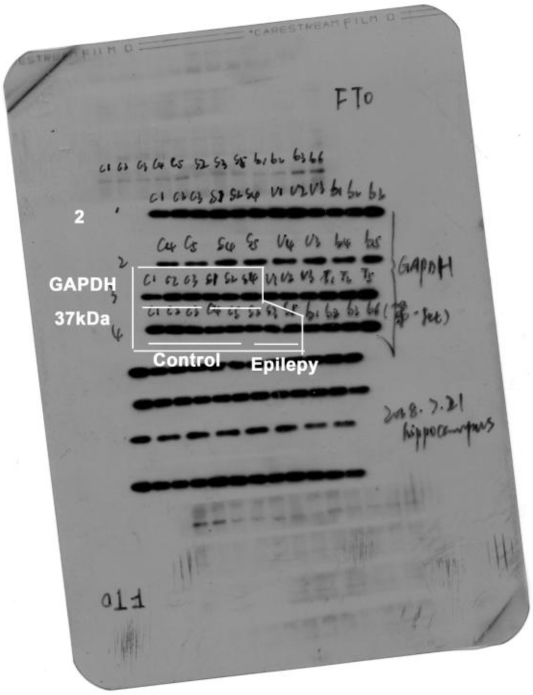

Supplement: Supplementary Materials — Tab S1: the data set of 4173 proteins. Figure S1: the principal component analysis (PCA) and quantitative protein cluster analysis. Figure S2: the heatmap clustering analysis of 27 DEPs. Figure S3: the enriched protein counts by GO term and KEGG pathway analysis of 4173 proteins. Figure S4: an original mass spectrometry data (uploaded to Proteome X change Datasets). Figure S5: original western blotting images: (1) GFAP and GAPDH; (2) CaMKII-α and GAPDH; (3) CaMKII-β and GAPDH; (4) F-actin and GAPDH. [file 8511066.f1.zip › Supplementary data_figure S1-S5.pdf]
