# Supplementary material for: Proteomic Analysis Reveals the Vital Role of Synaptic Plasticity in the Pathogenesis of Temporal Lobe Epilepsy
Source: Neural Plast. 2022 Jul 11;2022:8511066. doi: 10.1155/2022/8511066 (PMC9293557; doi:10.1155/2022/8511066)
Supplement: Supplementary Materials — Tab S1: the data set of 4173 proteins. Figure S1: the principal component analysis (PCA) and quantitative protein cluster analysis. Figure S2: the heatmap clustering analysis of 27 DEPs. Figure S3: the enriched protein counts by GO term and KEGG pathway analysis of 4173 proteins. Figure S4: an original mass spectrometry data (uploaded to Proteome X change Datasets). Figure S5: original western blotting images: (1) GFAP and GAPDH; (2) CaMKII-α and GAPDH; (3) CaMKII-β and GAPDH; (4) F-actin and GAPDH. [file 8511066.f1.zip › Tab S1 data set of 4173 Proteins (3).pdf]

N	Unused	%Cov(95)	Accession	Name	Peptides(95%)	114:113	115:113	116:113	117:113	118:113	Fold change	P value
1	366.66	71.03	A0A0G2JZ69	Spectrin alpha chain, non-erythrocytic 1	289	1.66	2.21	1.75	1.69	1.41	0.93393885	0.0516
2	219.35	55.05	G3V6S0	Spectrin beta chain	192	1.57	1.32	1.36	1.54	1.15	0.97292466	0.45
3	195.78	38.14	M0R9X8	Cytoplasmic dynein 1 heavy chain 1	174	1.04	1.19	1.32	1.45	1.45	0.97522026	0.127
4	176.12	62.41	F1LNK0	Microtubule-associated protein	152	1.89	2.78	1.60	0.82	1.63	0.88515377	0.0756
5	166.86	53.60	F1MA36	Spectrin beta chain	151	2.01	1.96	0.67	1.58	1.25	0.94861856	0.25
6	158.47	54.51	P11442	Clathrin heavy chain 1	171	0.60	0.77	1.17	1.57	1.41	0.98261625	0.686
7	156.01	60.32	P06687	Sodium/potassium-transporting ATPase subunit alpha-3	225	0.91	0.33	1.19	1.12	1.26	1.07474917	0.308
8	152.27	33.74	F7F9U6	Plectin	129	1.19	1.27	3.13	1.87	1.91	0.98746396	0.573
9	140.62	32.92	A0A0G2K6R9	Ankyrin 2	125	1.11	1.13	0.92	1.13	1.18	0.99801956	0.926
10	129.22	81.57	P85108	Tubulin beta-2A chain	375	1.38	2.38	2.58	2.54	2.49	0.97198099	0.722
11	119.46	67.59	P21575	Dynamin-1	113	1.15	1.09	0.54	1.18	1.20	0.89378516	0.0587
12	118.66	84.70	Q6P9V9	Tubulin alpha-1B chain	317	null	null	null	null	null	0.98760086	0.785
13	117.95	34.79	P34926	Microtubule-associated protein 1A	86	0.74	0.96	0.86	0.77	0.86	0.90312651	0.00187
14	110.56	72.72	A0A0G2K6U1	Vesicle-fusing ATPase	97	1.08	1.31	1.12	1.94	1.98	0.96687413	0.141
15	107.71	36.57	F1LRL9	Microtubule-associated protein 1B	91	1.05	1.39	1.20	0.67	1.41	0.95184595	0.00655
16	106.08	66.72	P63018	Heat shock cognate 71 kDa protein	170	null	null	null	null	null	1.02044611	0.654
17	100.12	53.90	P62944	AP-2 complex subunit beta	83	1.10	0.87	1.03	1.41	1.41	0.96962581	0.192
18	98.22	83.24	P11980	Pyruvate kinase PKM	98	0.70	0.73	0.51	0.52	0.67	0.96593633	0.234
19	94.38	24.46	G3V984	Protein bassoon	73	0.77	1.57	0.81	1.03	1.33	0.97440943	0.471
20	93.83	55.12	P05708	Hexokinase-1	86	0.64	1.31	0.97	1.25	0.82	0.95627668	0.249
21	90.49	29.06	P12785	Fatty acid synthase	61	0.93	0.75	1.15	1.20	1.47	0.9893823	0.548
22	90.44	52.39	P82995	Heat shock protein HSP 90-alpha	75	2.61	1.64	2.36	1.89	2.19	0.96700818	0.379
23	88.88	70.03	P61765	Syntaxin-binding protein 1	87	1.28	1.00	1.38	1.87	2.09	1.00751408	0.863
24	88.49	60.77	Q9ER34	Aconitate hydratase, mitochondrial	106	0.44	1.05	0.77	0.95	0.79	0.94415786	0.104
25	85.72	53.80	G3V8V3	Alpha-1,4 glucan phosphorylase	58	1.20	1.08	1.25	1.14	0.91	0.92338231	0.0927
26	85.36	36.57	P11505	Plasma membrane calcium-transporting ATPase 1	64	1.54	0.59	0.70	2.07	2.05	0.96372939	0.543
27	84.94	51.42	Q63198	Contactin-1	58	1.07	1.37	1.87	2.13	1.80	1.00331874	0.923
28	84.86	74.18	P02770	Serum albumin	58	1.74	5.01	5.70	5.70	5.15	1.45195828	0.0182
29	83.76	67.81	P15999	ATP synthase subunit alpha, mitochondrial	130	0.46	1.26	0.75	0.90	0.74	0.93387412	0.169
30	82.94	22.55	F1M3W5	Dmx-like 2	60	0.98	1.06	0.86	1.24	1.21	0.96881963	0.139
31	82.4	59.68	P46462	Transitional endoplasmic reticulum ATPase	59	0.90	1.01	1.89	1.06	2.07	1.00538635	0.821
32	81.68	58.51	Q6GMN8	Actn1 protein	55	1.28	1.56	1.37	1.16	1.02	0.92146419	0.121
33	81.62	36.46	D4ABN3	Synaptojanin-1	51	1.12	1.72	0.79	1.49	1.26	0.92146419	0.036
34	81.05	74.86	G3V6D3	ATP synthase subunit beta	157	0.66	1.61	1.19	0.39	1.03	0.89751005	0.0716
35	80.14	49.54	D3ZUY8	Adaptor protein complex AP-2, alpha 1 subunit	57	1.11	0.29	0.93	1.12	1.53	0.99946642	0.989
36	80	28.14	G3V9Y1	Myosin, heavy polypeptide 10, non-muscle, isoform CRA_b	54	0.92	1.06	0.93	1.15	1.04	1.00751408	0.61
37	79.88	76.98	P47819	Glial fibrillary acidic protein	97	1.00	0.99	1.37	1.02	1.11	1.83400809	0.01342
38	78.28	81.48	A0A0G2K3K2	Actin, cytoplasmic 1	258	null	null	null	null	null	1.1011416	0.00544
39	77.83	55.11	P09951	Synapsin-1	105	0.65	1.63	0.84	1.72	1.69	0.92594602	0.144
40	77.19	77.84	P63039	60 kDa heat shock protein, mitochondrial	72	1.07	1.49	1.56	0.69	1.28	0.97996355	0.144
41	76.78	30.46	G3V852	RCG55135, isoform CRA_b	51	0.65	0.95	1.18	1.01	0.88	0.96855105	0.272
42	76.59	11.42	A0A0G2K9T4	Microtubule-actin cross-linking factor 1	74	0.81	0.70	0.99	0.76	0.83	1.0188912	0.0648
43	75.59	73.08	P47942	Dihydropyrimidinase-related protein 2	140	1.21	0.88	0.90	0.50	1.25	0.94102177	0.277
44	74.91	74.69	Q66HF1	NADH-ubiquinone oxidoreductase 75 kDa subunit, mitochondrial	60	0.69	0.83	0.83	0.91	0.60	1.10573065	0.0972
45	74.63	27.68	A0A0G2K4Y7	Unconventional myosin-Va	49	1.09	0.91	1.16	1.20	0.86	0.96079464	0.0271
46	74.08	43.12	P52873	Pyruvate carboxylase, mitochondrial	45	1.02	0.77	0.99	1.19	1.10	0.98071102	0.272
47	74.05	74.88	M0R5J4	Uncharacterized protein	111	0.52	1.47	2.65	1.57	3.13	1.10343374	0.127
48	73.74	53.17	P47860	ATP-dependent 6-phosphofructokinase, platelet type	58	1.94	1.53	4.33	2.86	3.84	1.10496449	0.0368
49	73.53	42.52	0A0G2K0M8	Neural cell adhesion molecule 1	57	1.26	1.69	1.72	0.32	0.96	0.94245785	0.284
50	72.73	47.67	P05197	Elongation factor 2	43	1.43	0.98	2.58	1.84	2.44	1.02647607	0.404
51	72.1	70.50	D4A133	ATPase H+-transporting V1 subunit A	91	1.02	1.17	1.01	1.33	1.28	0.93627225	0.0259
52	71.49	51.19	F1LRV4	Heat shock 70 kDa protein 4	49	0.75	1.04	1.16	1.31	1.39	0.95939711	0.0227

53	71.42	57.45	P06686	Sodium/potassium-transporting ATPase subunit alpha-2	189	1.34	0.40	2.49	1.54	1.84	1.06414888	0.313
54	70.31	56.95	O35303	Dynamin-1-like protein	49	0.88	1.19	1.02	0.96	0.97	0.90125046	0.00073
55	69.83	75.68	P04797	Glyceraldehyde-3-phosphate dehydrogenase	110	1.01	0.59	1.51	0.53	1.96	1.04210453	0.421
56	69.74	50.87	F1M9V7	Aminopeptidase puromycin-sensitive	43	0.95	1.28	1.24	1.63	1.36	0.97765679	0.418
57	69.07	76.26	P16617	Phosphoglycerate kinase 1	79	0.60	1.17	1.94	1.51	2.19	1.02342118	0.232
58	69.05	40.66	P19491	Glutamate receptor 2	47	1.36	1.29	1.38	1.84	2.05	1.01903246	0.695
59	68.32	43.50	Q5XI78	2-oxoglutarate dehydrogenase,	39	0.97	1.05	1.22	1.11	0.90	1.04964393	0.418
60	68.27	70.48	P13233	2',3'-cyclic-nucleotide 3'-phosphodiesterase	72	1.22	0.21	1.64	2.33	3.19	1.21756602	0.205
61	67.76	38.09	A0A0G2K5E7	ATP-citrate synthase	41	1.12	0.96	0.73	1.20	0.87	0.98322943	0.497
62	67.51	68.45	G3V8C3	Vimentin	49	1.72	2.47	23.77	3.94	7.11	1.56049096	0.12742
63	67.21	38.54	P11507	Sarcoplasmic/endoplasmic reticulum calcium ATPase 2	52	0.76	0.53	1.24	0.97	1.85	0.98986247	0.781
64	66.84	54.52	Q64428	Trifunctional enzyme subunit alpha, mitochondrial	50	0.28	0.88	1.25	1.11	1.10	1.03943533	0.344
65	66.48	34.58	D4A8U7	Dynactin 1, isoform CRA_a	42	1.20	1.31	1.34	1.46	1.31	0.96700818	0.0243
66	65.95	33.86	Q8K3M6	ERC protein 2	37	0.88	1.20	0.98	1.01	1.22	1.00016845	0.993
67	65.53	38.10	Q9QXY2	SRC kinase signaling inhibitor 1	34	1.05	1.92	0.83	1.19	1.17	0.96761162	0.0563
68	65.47	40.08	Q5U300	Ubiquitin-like modifier-activating enzyme 1	53	0.94	1.01	1.50	1.49	1.38	0.97779233	0.337
69	65.31	62.33	P00507	Aspartate aminotransferase, mitochondrial	55	0.28	0.84	0.86	1.67	1.02	1.0067392	0.934
70	65.23	35.12	P97536	Cullin-associated NEDD8-dissociated protein 1	36	0.86	0.41	0.98	0.79	1.04	1.00694857	0.678
71	64.55	64.40	Q6P502	T-complex protein 1 subunit gamma	56	1.11	0.81	1.38	1.28	1.19	0.96674011	0.38
72	64.44	30.49	A0A096MJE6	Tenascin R, isoform CRA_b	55	1.00	0.84	1.24	0.88	1.27	1.00300584	0.916
73	64.2	35.32	P97685	Neurofascin	41	0.93	1.16	1.11	1.10	0.95	1.01832636	0.628
74	62.87	58.91	Q5RKI0	WD repeat-containing protein 1	44	0.68	0.85	1.66	1.42	1.56	1.0018177	0.942
75	62.24	39.59	R9PXU6	Vinculin	40	0.99	0.95	1.72	1.74	1.57	1.03261324	0.146
76	62.18	59.12	D4ACB8	Chaperonin subunit 8 (Theta) (Predicted), isoform	41	0.98	1.05	1.00	1.13	0.99	0.9641971	0.0991
77	61.97	82.78	P62815	V-type proton ATPase subunit B, brain isoform	82	1.03	0.81	1.25	0.95	1.42	0.98931373	0.836
78	61.75	27.10	M0RAP5	SET-binding factor 1	42	1.03	1.11	1.06	1.34	2.00	1.07251662	0.281
79	61.62	49.39	P06761	78 kDa glucose-regulated protein	57	0.91	0.98	1.47	0.64	1.47	1.03068251	0.539
80	61.48	37.08	Q2TA68	Dynamin-like 120 kDa protein, mitochondrial	40	0.70	0.99	1.07	1.27	1.33	1.04702798	0.158
81	61.42	53.74	G3V826	Transketolase	39	0.64	0.19	0.32	0.76	0.66	1.0420323	0.318
82	61.39	74.72	P50398	Rab GDP dissociation inhibitor alpha	67	1.20	1.25	1.10	0.65	1.32	0.91955005	0.0073
83	61.03	25.38	A0A140UHX6	Spectrin beta chain	53	0.95	1.18	0.70	0.97	0.86	0.98630107	0.465
84	60.8	86.81	P05065	Fructose-bisphosphate aldolase A	107	0.61	0.74	1.46	0.90	1.61	1.0341891	0.35
85	60.54	52.14	F1M953	Stress-70 protein, mitochondrial	53	0.77	1.06	1.22	0.55	1.56	0.99536668	0.924
86	59.85	68.41	Q5XIM9	T-complex protein 1 subunit beta	43	1.09	1.00	1.08	1.10	1.09	0.92916067	0.074
87	59.82	33.39	P10687	1-phosphatidylinositol 4,5-bisphosphate phosphodiesterase beta-1	35	1.38	0.90	1.24	1.14	1.50	0.99804723	0.923
88	59.82	58.47	F1LX07	Solute carrier family 25 member 12	44	0.64	1.03	0.68	1.16	0.67	0.92594602	0.144
89	59.77	60.46	P11275	Calcium/calmodulin-dependent protein kinase type II subunit alpha	77	0.52	2.73	4.25	5.20	6.92	1.90263655	0.01782
90	59.56	48.83	P35571	Glycerol-3-phosphate dehydrogenase,	35	0.91	1.26	0.91	1.26	1.20	0.98193539	0.543
91	59.37	82.72	P07323	Gamma-enolase	105	1.24	1.11	1.04	0.70	1.64	0.94921052	0.34
92	59.18	47.82	Q52KS1	ATP-dependent 6-phosphofructokinase	56	0.93	0.84	1.29	1.82	1.06	1.05489543	0.301
93	58.96	63.77	Q68FQ0	T-complex protein 1 subunit epsilon	48	1.19	0.81	1.02	0.91	1.06	0.95992926	0.24
94	58.87	64.86	O35567	Bifunctional purine biosynthesis protein PURH	33	0.79	0.75	1.11	0.97	0.96	1.00210244	0.896
95	58.56	27.92	F1LUA1	Early endosome antigen 1	32	0.96	1.00	1.00	1.03	1.12	1.01846754	0.389
96	58.01	51.22	P63319	Protein kinase C gamma	43	0.96	0.61	0.70	1.05	1.06	0.9857543	0.828
97	57.74	49.76	P53534	Glycogen phosphorylase, brain form (Fragment)	62	0.97	0.91	0.96	0.97	0.93	0.89751005	0.0715
98	57.44	27.02	Q6PW34	Neuronal cell adhesion molecule	42	1.25	0.75	2.23	1.15	1.45	1.00940141	0.822
99	57.15	38.58	Q2I6B2	V-type proton ATPase subunit a	50	1.34	1.45	1.08	1.04	1.21	0.92980494	0.0316

100	57.08	43.71	Q66HA8	Heat shock protein 105 kDa	41	1.26	0.62	1.01	0.65	1.45	0.96506632	0.252
101	57.06	40.55	A0A0A0MY0	Endoplasmin	40	0.90	1.27	1.69	1.13	1.63	1.02122446	0.143
102	56.83	47.94	A0A0G2JVH4	MIC	42	0.90	1.38	1.03	0.95	1.06	0.91066983	0.00355
103	56.78	45.27	P12346	Serotransferrin	34	2.11	0.63	3.13	2.83	2.42	1.11187916	0.215
104	56.59	55.86	Q5XI34	Protein phosphatase 2 (Formerly 2A), regulatory subunit A (PR 65), alpha isoform, isoform CRA_a	44	1.01	0.72	0.99	1.04	1.17	0.96319513	0.214
105	56.57	68.09	G3V733	Synapsin II, isoform CRA_a	79	1.01	1.53	0.49	0.69	1.07	0.83798714	0.0318
106	56.12	48.58	P06685	Sodium/potassium-transporting ATPase subunit alpha-1	149	0.82	0.65	0.56	0.65	0.90	1.04673773	0.468
107	55.93	31.38	Q91Z79	Liprin-alpha-3	34	1.20	1.28	0.92	0.89	0.89	0.99157926	0.634
108	55.8	55.49	A0A0H2UHM	Protein disulfide-isomerase	33	0.59	1.03	1.39	0.86	1.28	1.02157845	0.653
109	55.74	36.76	P12839	Neurofilament medium polypeptide	38	0.91	1.11	0.90	0.52	1.21	1.02683189	0.777
110	55.64	26.75	Q9ERH3	WD repeat-containing protein 7	36	0.99	0.51	0.97	1.04	1.33	0.93044966	0.00979
111	55.45	36.86	D4A9L5	Band 4.1-like protein 1	35	1.51	1.53	1.26	1.27	1.51	1.00716496	0.645
112	55.23	40.39	F1LRI7	AP2-associated protein kinase 1	31	1.12	1.17	0.92	0.70	0.95	0.98513954	0.436
113	55.21	55.38	P10860	Glutamate dehydrogenase 1, mitochondrial	51	1.07	0.53	2.01	1.84	1.10	1.02143684	0.784
114	54.84	50.83	P21707	Synaptotagmin-1	50	1.00	1.82	1.12	1.03	0.83	0.89502507	0.0156
115	54.72	59.55	Q7TPB1	T-complex protein 1 subunit delta	34	1.64	0.67	1.24	1.39	1.37	0.96908828	0.297
116	54.66	69.23	D4A5G8	Pyruvate dehydrogenase E1 component subunit alpha	40	0.96	1.05	0.73	1.27	0.73	0.91700404	0.103
117	54.36	71.13	P07335	Creatine kinase B-type	100	1.03	1.28	2.36	1.56	1.67	1.07400447	0.194
118	54.22	56.01	D4ACG7	Copine 6	36	0.98	1.01	0.44	0.99	1.03	0.91003882	0.0327
119	54.03	42.72	B4F772	Heat shock 70 kDa protein 4L	35	1.15	1.04	1.18	0.92	1.60	0.98787472	0.411
120	53.99	58.49	F1LM47	Succinate--CoA ligase [ADP-forming] subunit beta, mitochondrial	35	0.61	0.88	0.75	1.27	1.26	0.98220767	0.646
121	53.83	22.70	D3ZA84	Talin 2	47	0.62	0.79	0.57	0.70	0.64	0.97501749	0.145
122	53.8	56.63	Q6P6V0	Glucose-6-phosphate isomerase	48	0.74	1.74	1.33	1.20	1.20	0.94409242	0.126
123	52.84	58.82	D4AC23	Chaperonin-containing TCP1 subunit 7	35	1.26	0.63	1.10	1.13	1.34	0.99829631	0.927
124	52.73	31.02	P70600	Protein-tyrosine kinase 2-	28	0.94	0.94	0.66	0.99	1.14	0.99368466	0.651
125	52.5	55.95	Q920L2	Succinate dehydrogenase [ubiquinone] flavoprotein subunit, mitochondrial	40	0.76	0.65	1.36	1.14	1.13	0.95224189	0.294
126	52.25	32.72	F1LLX6	Calcium-dependent secretion activator 1	38	0.61	0.71	0.39	0.40	0.73	1.01087177	0.677
127	52.16	35.56	G3V8G4	Brevican core protein	35	0.92	0.48	1.27	1.19	1.39	1.0118532	0.867
128	52	44.75	P31016	Disks large homolog 4	32	0.69	0.72	0.94	1.04	1.20	1.0285415	0.0619
129	51.83	25.92	Q9QUH6	Ras/Rap GTPase-activating protein SynGAP	32	0.88	1.79	1.60	1.67	2.19	0.99698244	0.721
130	51.78	47.92	Q66HM2	AP-2 complex subunit alpha-2	60	0.98	0.32	0.79	1.33	1.06	0.98275248	0.635
131	51.53	12.06	G3V928	LDL receptor-related protein 1	47	0.69	0.58	1.12	0.97	1.02	1.00117905	0.974
132	51.03	37.66	Q9JK11	Reticulon-4	34	0.91	1.12	1.17	0.82	1.57	1.0073046	0.832
133	50.97	45.02	Q499Q4	Phosphoglucomutase 1	29	0.81	0.70	1.26	0.97	1.06	0.97467963	0.469
134	50.95	18.95	C0JPT7	Filamin A	32	0.82	2.33	1.63	2.63	1.50	1.01017133	0.559
135	50.91	23.26	O70511	Ankyrin-3	55	1.18	1.22	1.07	1.14	1.64	1.0115727	0.369
136	50.87	39.26	D4A435	Intercellular adhesion molecule 5	43	1.57	0.33	0.93	1.49	1.12	0.95462101	0.591
137	50.57	43.68	Q6IMZ3	Annexin	31	0.90	0.96	0.99	1.19	0.92	0.98541272	0.497
138	50.22	23.78	F1LM33	Leucine-rich PPR motif-containing protein, mitochondrial	26	1.33	0.86	0.72	1.85	0.49	1.0032631	0.898
139	50.1	65.20	P50554	4-aminobutyrate aminotransferase, mitochondrial	44	0.50	0.47	1.00	0.55	0.72	0.97056719	0.525
140	50.07	53.87	P19527	Neurofilament light polypeptide	50	0.93	0.91	0.82	0.36	1.24	1.01747969	0.885
141	50.04	63.54	Q68FY0	Cytochrome b-c1 complex subunit 1, mitochondrial	67	1.64	1.63	2.05	1.36	1.72	0.93744113	0.0397
142	49.91	74.83	Q62950	Dihydropyrimidinase-related protein 1	80	2.17	1.63	1.39	1.26	1.89	0.93763609	0.128
143	49.52	66.30	Q9JKB7	Guanine deaminase	34	1.25	0.95	0.72	0.57	0.35	0.85856544	0.00807
144	49.38	76.56	A0A0G2JV65	14-3-3 protein zeta/delta	53	1.80	2.31	2.81	3.22	3.08	1.01374866	0.622
145	49.35	40.19	A0A0G2KB92	Serine/threonine-protein kinase DCLK1	29	1.45	1.94	1.66	2.61	2.65	1.00905163	0.844
146	49.3	54.50	P28480	T-complex protein 1 subunit alpha	33	0.99	0.98	1.00	1.01	0.99	0.9789452	0.472
147	49.26	53.50	A0A0G2JTH6	EH domain-containing protein 3	29	1.51	1.42	1.77	1.16	1.64	1.00502403	0.811

148	49.24	26.58	A0A0G2JT63	Cytoplasmic FMR1-interacting protein 2	30	1.51	1.34	1.26	1.75	1.72	0.98105097	0.453
149	48.99	48.22	P13264	Glutaminase kidney isoform, mitochondrial	41	0.93	0.93	1.20	1.17	1.32	0.98602764	0.353
150	48.93	53.01	P08461	Dihydrolipoyllysine-residue acetyltransferase component of pyruvate dehydrogenase complex, mitochondrial	40	0.58	0.79	0.79	0.69	1.09	1.05511482	0.31
151	48.57	42.07	D3ZC55	Heat shock 70kDa protein 12A (Predicted), isoform CRA_a	27	1.00	1.10	0.52	1.13	0.98	0.90563398	0.0404
152	48.56	61.78	P23565	Alpha-internexin	37	0.90	1.27	1.07	0.74	1.28	1.03619821	0.718
153	48.46	42.36	O35814	Stress-induced-phosphoprotein 1	26	1.27	1.64	1.92	0.97	1.85	0.99427029	0.771
154	48.23	51.79	Q3MHS9	Chaperonin containing Tcp1, subunit 6A (Zeta 1)	36	1.17	1.20	1.32	1.31	1.22	0.99137309	0.51
155	47.58	68.34	P04636	Malate dehydrogenase, mitochondrial	62	1.04	0.56	1.01	0.39	1.16	0.90062598	0.107
156	47.49	25.21	Q9ERS3	Voltage-dependent calcium channel subunit alpha-2/delta-1	25	1.84	1.19	1.67	1.61	1.49	0.97738577	0.228
157	47.29	31.27	A0A0G2K7Y2		30	1.03	1.43	1.08	0.87	0.91	0.98322943	0.475
158	47.2	59.51	P32551	Cytochrome b-c1 complex subunit 2, mitochondrial	50	1.39	1.74	1.38	1.80	1.43	0.95131828	0.209
159	47.01	61.79	P17764	Acetyl-CoA acetyltransferase, mitochondrial	39	0.25	0.70	0.85	0.90	1.36	1.03777954	0.327
160	47	35.62	F2Z3Q8	Importin subunit beta-1	36	1.05	0.84	1.25	1.04	1.08	0.97522026	0.163
161	46.99	24.20	P55161	Nck-associated protein 1	40	1.75	1.26	1.33	2.75	3.16	1.01522535	0.619
162	46.9	17.59	A0A0G2JZI2	Glutamyl-prolyl-tRNA synthetase	25	1.29	1.46	1.56	1.39	1.34	0.97996355	0.416
163	46.84	22.13	Q64605	Receptor-type tyrosine-protein phosphatase S	30	0.99	0.95	0.95	0.95	0.93	0.8962667	0.137
164	46.76	39.37	O35095	Neurochondrin	42	0.95	0.16	0.79	0.60	0.95	1.02562263	0.647
165	46.6	35.07	Q6P136	Hyou1 protein	31	1.09	1.20	1.47	0.58	1.51	0.92594602	0.00848
166	46.36	44.85	G3V936	Citrate synthase	40	1.07	1.22	1.25	1.49	0.92	0.92466328	0.251
167	46.12	42.72	A0A0G2JSM7	Adducin 1 (Alpha), isoform CRA_b	37	1.18	1.07	1.13	1.09	1.24	0.96026201	0.111
168	46.01	26.57	Q8VHK2	Caskin-1	24	0.82	0.98	0.90	0.80	0.75	1.01832636	0.542
169	45.99	82.66	B4F7C2	Tubulin beta chain	293	0.69	0.63	0.74	0.58	1.22	0.99130437	0.924
170	45.64	57.87	P13221	Aspartate aminotransferase, cytoplasmic	49	0.67	0.93	0.70	1.20	1.09	0.93044966	0.124
171	45.5	25.72	A0A0G2JZF2	CAP-Gly domain-containing linker protein 2	26	1.02	1.36	1.13	0.36	0.84	1.00573485	0.573
172	45.45	17.06	D3ZES7	Plexin A4	29	0.80	0.74	1.18	1.27	1.29	1.02676071	0.221
173	45.42	64.17	Q9Z0W5	Protein kinase C and casein kinase substrate in neurons protein 1	39	1.56	1.08	1.41	0.52	1.45	0.95356289	0.0334
174	45.07	28.49	A0A0G2K2L8	Peripheral plasma membrane protein CASK	25	0.83	0.72	0.80	0.87	0.78	0.97901305	0.0306
175	44.59	54.42	P04785	Protein disulfide-isomerase	25	0.90	1.06	2.05	0.81	1.42	1.06776932	0.0852
176	44.54	25.32	Q6RJR6	Reticulon-3	28	0.90	1.19	0.76	1.03	1.08	0.97427436	0.367
177	44.19	48.22	G3V7J0	Aldehyde dehydrogenase family 6, subfamily A1, isoform CRA_b	29	1.17	0.93	1.82	2.05	1.29	1.03526492	0.467
178	44.17	32.11	P31596	Excitatory amino acid transporter 2	52	1.74	0.27	1.89	1.85	1.89	1.15829281	0.101
179	44.07	51.63	P63329	Serine/threonine-protein phosphatase 2B catalytic subunit alpha isoform	37	1.39	0.97	1.05	1.72	1.61	1.01171294	0.893
180	43.97	36.92	E9PT29	DEAD-box helicase 17	24	1.43	1.20	1.51	1.43	1.37	1.02533831	0.354
181	43.9	51.17	Q68FX0	Isocitrate dehydrogenase [NAD] subunit beta, mitochondrial	40	1.06	1.27	0.86	1.31	1.17	0.98268436	0.748
182	43.86	34.71	P50475	Alanine--tRNA ligase, cytoplasmic	30	0.79	1.36	1.31	1.07	1.21	0.97542307	0.315
183	43.76	43.11	Q75Q39	Mitochondrial import receptor subunit T	27	1.01	0.57	1.25	1.04	1.32	1.00089456	0.963
184	43.7	48.85	F1LMC7	Septin-7	31	1.47	1.38	1.75	2.09	1.84	0.99899544	0.97
185	43.52	24.26	Q99JD4	CLIP-associating protein 2	26	1.00	1.06	0.88	1.18	1.19	1.02790006	0.588
186	43.41	54.02	Q6GMN2	Brain-specific angiogenesis inhibitor 1-associated protein 2	25	1.21	1.57	0.98	1.45	1.42	0.90751916	0.0292
187	43.29	52.48	Q9JHU0	Dihydropyrimidinase-related protein 5	28	0.95	0.86	0.82	0.77	0.75	0.9347808	0.116
188	43.16	20.37	Q63633	Solute carrier family 12 member 5	24	1.27	0.95	0.83	1.63	0.74	0.97806347	0.264
189	42.7	38.16	A0A0G2JT93	Catenin (Cadherin associated protein), beta 1, isoform CRA_a	31	1.10	0.81	1.22	0.96	1.34	0.95277007	0.118

190	42.62	29.59	Q66X93	Staphylococcal nuclease domain-containing protein 1	25	0.79	0.96	0.86	0.86	0.80	1.00149832	0.935
191	42.62	45.35	A0A0G2K1D2	RAP1, GTP-GDP dissociation stimulator 1 (Predicted), isoform CRA_a	25	1.10	0.90	1.77	1.31	1.47	0.98725864	0.613
192	42.54	33.48	Q5U302	Catenin (Cadherin associated protein), alpha 1	26	1.03	0.91	1.54	1.13	1.33	1.00189408	0.954
193	42.5	33.86	P33124	Long-chain-fatty-acid--CoA ligase 6	24	0.76	0.47	0.94	0.95	0.86	0.99429096	0.919
194	42.03	34.50	A0A0G2K876	Phosphodiesterase	26	1.46	0.42	0.74	0.86	0.79	0.97265495	0.445
195	41.92	59.78	P63086	Mitogen-activated protein kinase 1	27	1.72	1.60	1.10	1.45	0.98	0.92980494	0.0175
196	41.76	56.93	P04642	L-lactate dehydrogenase A chain	31	1.69	1.36	3.53	3.13	3.77	1.05306902	0.188
197	41.7	56.30	P09606	Glutamine synthetase	62	1.14	0.09	1.84	0.72	1.27	1.08899702	0.481
198	41.67	33.44	A0A0G2K0B6	Clathrin coat assembly protein AP180	36	1.21	1.20	0.91	0.71	0.89	0.95946361	0.122
199	41.59	60.31	Q6P6R2	Dihydrolipoyl dehydrogenase,	32	0.64	0.48	1.19	0.78	0.79	0.98493471	0.787
200	41.16	23.94	Q5BJZ3	Nicotinamide nucleotide transhydrogenase	24	0.44	0.59	1.01	1.27	0.97	1.27898558	0.0228
201	40.99	21.17	G3V6P7	Myosin, heavy polypeptide 9, non-muscle	36	0.78	0.90	1.13	0.79	1.03	1.00835246	0.446
202	40.97	29.74	G3V6L4	Kinesin-like protein	28	0.96	0.95	0.92	0.98	0.95	0.99206049	0.518
203	40.9	57.82	Q5BK63	NADH dehydrogenase [ubiquinone] 1 alpha subcomplex subunit 9, mitochondrial	24	0.79	1.16	1.04	1.39	1.10	0.9562104	0.284
204	40.85	21.54	Q64560	Tripeptidyl-peptidase 2	24	1.09	0.48	0.97	1.18	1.09	1.03957944	0.0399
205	40.8	63.71	P62632	Elongation factor 1-alpha 2	34	0.84	0.85	1.58	1.34	1.15	1.01508462	0.697
206	40.57	57.52	F1LNT0	Dihydropyrimidinase-related protein 4	34	1.34	1.16	1.31	0.67	0.90	0.93174043	0.0658
207	40.57	63.42	Q09073	ADP/ATP translocase 2	38	0.46	0.72	1.72	1.96	1.45	1.06503438	0.299
208	40.46	21.79	A0A0G2JU11	Rap guanine nucleotide exchange factor 2	23	1.31	1.07	0.67	1.28	1.32	1.00689274	0.776
209	40.43	51.50	F1LUE2	Calcium/calmodulin-dependent protein kinase II, beta, isoform CRA_c	67	0.68	1.31	4.57	4.33	5.92	1.73507737	0.01056
210	40.22	28.00	Q924S5	Lon protease homolog, mitochondrial	24	0.87	1.10	1.11	1.13	0.89	1.07624013	0.0521
211	40.19	53.80	Q08163	Adenylyl cyclase-associated protein 1	31	1.49	0.83	1.69	1.15	1.98	1.06776932	0.234
212	40.06	32.58	Q6IMY8	Heterogeneous nuclear ribonucleoprotein U	30	1.53	0.48	1.96	1.05	1.66	1.02526724	0.551
213	39.95	39.77	O08651	D-3-phosphoglycerate dehydrogenase	25	0.55	0.28	1.24	0.78	1.22	1.03792342	0.602
214	39.72	38.78	Q6P7A7	Dolichyl-diphosphooligosaccharide--protein glycosyltransferase subunit 1	21	0.76	0.53	0.83	0.86	0.72	1.04376722	0.267
215	39.68	44.79	F1LPP0	Amphiphysin	27	1.60	2.49	0.94	0.85	1.31	0.86874219	0.00838
216	39.66	33.39	P11506	Plasma membrane calcium-transporting ATPase 2	51	0.96	0.37	0.96	1.49	1.50	1.03641371	0.459
217	39.47	51.31	Q5FVI6	V-type proton ATPase subunit C 1	25	0.95	1.00	1.21	1.51	2.01	0.98268436	0.464
218	39.44	30.38	A0A0G2JY26	DnaJ heat shock protein family (Hsp40) member C6	25	0.78	0.82	0.84	1.24	1.34	0.99171673	0.631
219	39.37	54.79	O88989	Malate dehydrogenase, cytoplasmic	42	0.63	1.39	1.51	1.03	1.53	1.00682992	0.682
220	39.3	68.90	A0A0G2K3Q6	Fructose-bisphosphate aldolase	62	1.64	1.51	1.92	1.47	0.51	0.97596411	0.598
221	39.28	31.06	Q9WV63	Kinesin-like protein KIF2A	25	1.28	1.13	0.94	1.14	1.24	0.98691655	0.655
222	39.26	47.80	G3V945	Aldehyde dehydrogenase family 5, subfamily A1	25	0.55	0.30	0.72	0.95	0.84	1.00025996	0.994
223	39.21	44.75	D3ZAA9	MAGUK p55 subfamily member 2	22	1.02	1.03	1.13	0.70	0.82	0.95290216	0.101
224	39.13	36.39	G3V8L3	Lamin A, isoform CRA_b	25	1.15	1.27	1.13	0.72	0.99	0.96226089	0.218
225	39.09	56.50	P59215	Guanine nucleotide-binding protein G(o) subunit alpha	61	1.89	1.07	1.26	1.89	1.91	0.93653187	0.24
226	38.83	48.75	Q68FS4	Cytosol aminopeptidase	24	1.11	1.05	2.09	1.41	1.43	1.04884392	0.141
227	38.62	47.24	A0A140TAH5	AP-2 complex subunit mu	28	1.20	0.87	1.05	1.31	1.42	1.0320408	0.524
228	38.5	49.42	G3V7L6	26S protease regulatory subunit 7	21	0.97	0.98	0.98	0.95	0.97	0.99723816	0.77
229	38.42	19.87	P70587	Leucine-rich repeat-containing protein 7	22	0.89	1.18	1.13	1.42	1.22	1.02009252	0.453
230	38.38	19.04	D4AE00	AP-3 complex subunit beta	23	1.22	0.85	1.08	1.13	1.41	0.99302366	0.742
231	38.31	53.24	E9PSV5	Phosphoserine aminotransferase	24	1.08	0.95	2.00	1.74	1.39	1.0535801	0.316
232	38.22	95.24	A0A0G2JSW3	Globin a4	66	0.59	1.10	0.87	0.72	2.09	1.18181155	0.462
233	38	54.55	O35179	Endophilin-A1	38	1.19	1.58	0.79	0.49	1.28	0.84089642	0.0101
234	37.83	31.82	A0A096MJ42	Disks large homolog 3	24	0.90	1.31	1.15	0.88	1.08	0.99332657	0.593

235	37.81	32.53	D3ZQG6	Tripartite motif-containing protein 2	24	1.03	1.02	1.00	1.00	1.01	0.97359928	0.0388
236	37.8	30.01	A0A0G2K401	Propionyl-CoA carboxylase alpha chain, mitochondrial	20	1.10	0.91	1.01	1.05	1.07	0.9694914	0.149
237	37.67	24.47	F1LUT4	Phospholipid-transporting ATPase	27	1.13	1.09	0.74	0.79	1.31	0.96413027	0.269
238	37.61	50.44	P85834	Elongation factor Tu, mitochondrial	27	0.90	1.43	1.10	0.85	1.47	0.96332867	0.195
239	37.6	50.43	A0A0G2K793	Heat shock protein HSP 90-beta	64	1.85	1.43	2.13	2.15	1.98	0.97853815	0.601
240	37.57	18.96	P14046	Alpha-1-inhibitor 3	28	0.21	1.38	1.50	1.60	1.75	1.10266916	0.115
241	37.52	24.54	A0A1B0GWY	Rho guanine nucleotide exchange factor 2	22	1.25	0.95	0.80	1.17	1.07	0.95488573	0.138
242	37.43	47.34	Q5M964	Fumarate hydratase 1	26	0.58	1.74	0.89	0.85	1.20	0.93984842	0.0514
243	37.4	32.86	P19490	Glutamate receptor 1	36	0.82	0.89	0.95	1.39	1.08	1.09353546	0.0759
244	37.32	29.72	A0A140TAA	Programmed cell death 6-interacting protein	24	0.56	0.65	0.95	0.86	1.01	0.99247317	0.668
245	37.18	85.54	P48500	Triosephosphate isomerase	43	0.86	0.27	1.46	1.10	1.50	1.06680759	0.432
246	36.97	10.47	D3Z9C7	Protein piccolo	31	1.18	1.63	0.92	1.20	1.60	1.00183158	0.935
247	36.88	31.06	Q9JHZ4	GRIP1-associated protein 1	18	1.32	1.34	0.90	0.82	0.94	0.92851685	0.0187
248	36.88	43.53	F1LQJ7	Phosphoenolpyruvate carboxykinase 2 (mitochondrial)	24	0.50	1.01	0.37	0.51	0.52	0.96132756	0.105
249	36.78	58.71	A0A0G2K1C	Actin-related protein 3	27	1.36	0.27	1.45	1.74	1.85	1.07698638	0.354
250	36.74	19.75	F1M5M9	SLIT-R	21	0.95	0.98	0.97	0.98	0.99	0.99653336	0.83
251	36.59	20.26	G3V8T4	DNA damage-binding protein 1	20	1.07	1.06	0.98	0.96	0.96	0.97569355	0.191
252	36.55	32.94	Q03555	Gephyrin	22	0.86	0.86	1.46	1.25	1.37	0.96399663	0.328
253	36.46	70.98	P62260	14-3-3 protein epsilon	46	1.98	2.81	3.25	2.42	2.99	0.97171154	0.465
254	36.33	28.92	Q641Y8	ATP-dependent RNA helicase DDX1	17	1.04	1.01	1.05	0.98	1.04	1.01614057	0.414
255	36.31	37.44	A0A0G2K89	Ezrin	22	0.19	0.48	0.74	0.60	0.82	1.07177346	0.415
256	36.28	15.75	A0A0G2K1L	Tenascin C	25	1.17	1.18	1.96	1.92	1.45	1.01311644	0.557
257	36.28	44.33	P15087	Carboxypeptidase E	24	0.93	1.11	1.14	0.79	1.17	0.93174043	0.0638
258	36.17	31.67	P40329	Arginine--tRNA ligase, cytoplasmic	20	1.07	0.47	1.00	0.87	0.82	1.04066087	0.0638
259	36.12	72.36	P50399	Rab GDP dissociation inhibitor beta	41	2.09	0.83	3.31	2.00	3.56	0.99734877	0.936
260	35.8	75.50	F8WFM2	Beta-soluble NSF attachment protein	31	1.07	0.82	1.21	0.69	1.41	0.99529079	0.853
261	35.69	48.27	B2GV06	Succinyl-CoA:3-ketoacid coenzyme A transferase 1, mitochondrial	29	0.79	1.51	1.21	1.00	0.93	0.95006623	0.153
262	35.6	33.92	D3ZVQ0	Ubiquitinyl hydrolase 1	22	1.24	0.99	1.58	1.05	1.19	0.99109826	0.561
263	35.56	22.47	F1M787	Catenin delta-2	24	1.04	0.98	1.03	1.04	1.02	1.01938569	0.327
264	35.55	23.34	P09216	Protein kinase C epsilon	22	1.17	1.08	0.94	1.04	1.34	1.02775757	0.14
265	35.29	14.68	A0A0G2K67	Spectrin beta chain	31	0.98	1.09	0.90	1.07	1.06	1.01304622	0.638
266	35.28	38.54	P11884	Aldehyde dehydrogenase, mitochondrial	19	0.37	0.90	0.94	1.13	1.24	1.01171294	0.759
267	35.26	35.61	B5DFN2	Adenosylhomocysteinase 2	25	0.60	0.26	0.77	0.44	0.52	0.99600853	0.959
268	35.15	44.92	Q641Y2	NADH dehydrogenase [ubiquinone] iron-sulfur protein 2, mitochondrial	26	0.79	1.12	0.76	0.84	0.84	0.93504001	0.0379
269	35.14	32.46	Q63560	Microtubule-associated protein 6	23	0.94	1.07	0.91	0.77	1.02	0.94566427	0.057
270	35.09	40.67	P52481	Adenylyl cyclase-associated protein 2	24	1.09	0.77	0.86	0.90	1.42	1.00369435	0.881
271	35.08	18.38	A0A0H2UHB	Anion exchange protein	23	0.79	0.65	0.97	0.46	0.37	1.0062648	0.843
272	35.07	61.05	Q62952	Dihydropyrimidinase-related protein 3	52	1.26	0.76	0.73	0.67	1.04	0.91509917	0.062
273	35.06	42.39	P85845	Fascin	23	1.77	0.77	2.00	2.17	1.60	1.00793318	0.847
274	35.03	31.56	Q9Z272	ARF GTPase-activating protein GIT1	19	1.21	0.95	1.15	1.19	1.38	1.00260268	0.82
275	35	25.83	P28037	Cytosolic 10-formyltetrahydrofolate dehydrogenase	18	1.22	1.03	1.08	1.12	0.81	0.93445688	0.172
276	34.96	52.03	Q5M7U6	Actin-related protein 2	39	1.53	0.82	1.17	1.56	1.75	1.01988042	0.742
277	34.93	50.24	A0A0G2JUC	Dynactin subunit 2	22	0.97	1.20	1.03	0.56	0.94	0.98753241	0.849
278	34.93	63.55	Q5XIH7	Prohibitin-2	24	0.95	1.45	1.32	1.27	1.43	0.98739552	0.604
279	34.91	52.82	P02688	Myelin basic protein	80	1.29	0.27	1.34	2.42	3.22	1.15829281	0.348
280	34.84	59.91	Q5XIH3	NADH dehydrogenase (Ubiquinone) flavoprotein 1	24	0.73	0.39	0.69	0.83	0.72	0.98016735	0.611
281	34.79	22.30	G3V6F4		21	null	null	null	null	null	1.01241445	0.179
282	34.72	54.61	A0A0G2KAM	Pyruvate dehydrogenase E1 component subunit beta, mitochondrial	46	0.91	1.20	1.15	0.84	1.03	0.92851685	0.0285
283	34.58	75.27	Q9Z2L0	Voltage-dependent anion-selective channel protein 1	30	0.53	0.82	0.71	0.75	0.54	0.91446509	0.103
284	34.54	33.29	Q5XIN6	LETM1 and EF-hand domain-containing protein 1, mitochondrial	23	0.90	1.20	1.03	1.20	1.05	1.08297505	0.166

285	34.51	47.91	F1LNF7	Isocitrate dehydrogenase [NAD] subunit,	42	0.53	1.09	1.24	1.15	1.42	0.9554816	0.118
286	34.4	45.67	Q4V8I9	UDP-glucose pyrophosphorylase 2	20	0.99	1.29	1.50	1.29	1.36	0.99509075	0.835
287	34.26	11.73	A0A0G2JY08	Myosin XVIIIa	21	1.17	1.14	0.97	1.06	0.96	1.00437638	0.828
288	34.12	12.45	D4A8D5	Filamin B	21	1.07	1.10	1.29	1.20	0.85	1.03907515	0.277
289	34.11	43.94	P50878	60S ribosomal protein L4	21	0.96	1.32	1.80	1.64	2.09	1.0702887	0.00037
290	34.09	19.56	Q8CGU4	Arf-GAP with GTPase, ANK repeat and PH domain-containing protein 2	20	0.85	1.31	1.05	1.49	1.66	0.9208257	0.00075
291	33.97	48.45	A0A0G2JWM	NAD-dependent protein deacetylase sirtuin-2	26	0.97	0.52	1.00	1.49	1.71	1.08147476	0.212
292	33.94	21.81	D3ZZK3	Eph receptor A4	21	1.13	0.90	1.05	1.32	1.53	1.03677296	0.251
293	33.93	34.96	O55096	Dipeptidyl peptidase 3	18	1.69	2.23	1.51	1.82	1.92	0.98037119	0.0909
294	33.91	39.08	Q5M9H2	Acyl-Coenzyme A dehydrogenase, very long chain	19	0.52	0.72	0.98	0.99	0.96	0.98979386	0.681
295	33.87	18.09	D3ZYN2	Kinesin family member 21A	24	0.77	0.90	0.68	0.78	0.94	1.0062369	0.832
296	33.73	34.24	B1WC61	Acad9 protein	19	0.70	1.32	1.42	1.20	1.02	1.01452189	0.503
297	33.72	22.84	F1LRZ7	Neurofilament heavy polypeptide	29	1.16	0.72	1.21	0.63	1.64	1.10037861	0.441
298	33.66	70.24	Q6NYB7	Ras-related protein Rab-1A	28	1.36	2.03	1.87	2.07	2.03	0.97670853	0.316
299	33.61	17.80	F1LN69	Misshapen-like kinase 1	21	0.95	1.28	0.69	0.93	1.02	0.95481954	0.202
300	33.59	37.31	P30835	ATP-dependent 6-phosphofructokinase, liver type	39	0.79	0.90	0.77	0.84	0.90	0.98500298	0.827
301	33.54	30.23	P0DJJ3	SH3-containing GRB2-like protein 3-interacting protein	22	1.04	0.98	0.95	0.46	1.03	1.00538635	0.74
302	33.51	17.16	G3V6T1	Coatomer subunit alpha	19	1.03	0.65	0.91	1.06	1.14	0.93945763	0.0229
303	33.47	63.18	A0A0G2JYB1	F-actin-capping protein subunit beta	20	1.79	1.15	2.36	2.56	3.40	1.07848043	0.109
304	33.44	39.60	P56574	Isocitrate dehydrogenase [NADP], mitochondrial	17	0.46	0.47	0.71	0.99	1.00	1.0222868	0.698
305	33.38	43.54	O08839	Myc box-dependent-interacting protein 1	24	1.12	1.34	1.22	0.72	1.43	0.96767869	0.267
306	33.37	15.99	P55067	Neurocan core protein	24	1.03	0.61	2.88	1.36	2.42	1.21083308	0.0814
307	33.36	38.43	Q08877	Dynamin-3	47	1.50	0.99	1.27	0.73	1.06	0.92594602	0.0694
308	33.29	17.00	F1LYS1	Regulating synaptic membrane exocytosis protein 1	19	0.66	1.03	0.82	0.91	0.97	0.9859593	0.139
309	33.25	27.51	A0A0G2JZK7	Sodium/calcium exchanger	21	0.96	0.59	1.13	1.51	1.08	0.99549778	0.862
310	33.17	16.73	Q63041	Alpha-1-macroglobulin	20	0.98	1.03	0.70	1.02	1.01	0.97589646	0.248
311	33.16	19.87	D3ZJ01	Similar to RIKEN cDNA 2310035C23	18	1.00	1.24	0.82	0.97	0.96	1.10573065	0.0849
312	33.13	12.28	A0A140TAJ5	Phosphatidylinositol 4-kinase alpha	21	0.93	0.58	0.78	0.98	0.90	0.99334722	0.537
313	33.04	20.88	D3ZPC4	Neural cell adhesion molecule L1	20	1.20	0.68	0.77	0.62	0.87	0.97461207	0.227
314	32.99	40.63	P35465	Serine/threonine-protein kinase PAK 1	26	1.31	1.43	1.51	1.24	1.13	1.02690306	0.328
315	32.65	20.23	D4AEH9	Amylo-1, 6-glucosidase, 4-alpha-glucanotransferase (Glycogen debranching enzyme, glycogen storage disease type III) (Predicted), isoform CRA_a	22	0.95	0.94	0.82	0.74	0.67	0.93815617	0.0868
316	32.59	24.25	O88588	Phosphofurin acidic cluster sorting protein 1	20	1.36	1.34	1.13	1.79	1.26	0.97535546	0.441
317	32.59	27.90	A0A0H2UHL	Drebrin	21	1.34	0.86	1.20	0.59	1.36	0.92338231	0.191
318	32.53	43.39	G3V6P2	Dihydrolipoamide S-succinyltransferase (E2 component of 2-oxo-glutarate complex), isoform	21	0.84	1.31	0.98	0.52	0.79	0.88761134	0.0131
319	32.42	48.61	P61265	Syntaxin-1B	26	0.77	0.89	1.20	1.27	1.28	1.00081825	0.979
320	32.38	40.39	A0A0G2K151	Apolipoprotein E	23	1.09	1.17	3.73	0.74	1.43	1.07698638	0.692
321	32.32	44.49	P61980	Heterogeneous nuclear ribonucleoprotein K	24	1.36	1.20	1.67	1.14	1.13	0.98030324	0.529
322	32.28	28.75	Q5XHZ0	Heat shock protein 75 kDa, mitochondrial	21	0.86	1.06	1.07	0.92	0.80	0.95667447	0.166
323	32.24	29.41	D3ZQD3		24	0.69	1.19	0.77	0.88	0.67	0.96326189	0.265
324	32.18	55.09	P42123	L-lactate dehydrogenase B chain	36	0.91	1.79	2.51	2.05	2.13	0.99102956	0.718
325	32.16	22.35	G3V6S5	C-1-tetrahydrofolate synthase, cytoplasmic	21	0.40	0.72	1.21	0.55	0.83	0.99829631	0.955
326	32.12	30.34	F8WFS9	Beta-adducin	26	1.96	1.09	0.98	1.10	1.54	0.98432047	0.625
327	32.1	23.22	D3ZHY9	RAS protein activator like 1 (GAP1 like) (Predicted)	16	1.84	1.64	0.57	1.42	0.65	0.89007573	0.0588
328	32.04	26.65	A0A0G2K1Q	Erythrocyte membrane protein band 4.1-like 3	24	1.07	0.88	0.65	0.81	0.99	0.95760328	0.19
329	32.03	14.62	M0RBJ7	Complement C3	23	1.58	0.98	1.22	1.46	1.28	1.0552611	0.498

330	31.94	59.09	Q5BJT9	Creatine kinase, mitochondrial 1, ubiquitous	42	0.70	0.64	1.20	1.08	0.72	0.95733781	0.227
331	31.89	9.78	D3ZC84	Ubiquitin specific peptidase 9, X chromosome	23	1.21	1.17	1.33	1.46	1.56	1.20748059	0.0268
332	31.81	51.42	A0A0G2K9E0	Ribonuclease inhibitor	18	0.57	0.38	1.18	0.54	1.00	0.97231791	0.475
333	31.77	23.85	G3V6S2	Aconitate hydratase	18	0.90	0.65	1.02	1.12	0.90	0.98828565	0.658
334	31.74	38.97	P21396	Amine oxidase [flavin-containing] A	17	1.00	0.77	1.24	1.25	0.93	0.97016363	0.376
335	31.67	32.03	F1LV13	Heterogeneous nuclear ribonucleoprotein M	27	0.99	0.81	1.19	0.88	1.20	1.1320989	0.00661
336	31.65	50.60	Q63092	CaM kinase-like vesicle-associated protein	20	1.09	0.64	0.56	0.89	0.39	0.91573369	0.00714
337	31.62	62.31	G3V804	Sideroflexin	28	1.08	0.70	0.72	1.32	1.06	0.95138422	0.00065
338	31.59	48.85	Q01986	Dual specificity mitogen-activated protein kinase kinase 1	24	1.16	1.18	1.12	1.00	1.45	0.95184595	0.0724
339	31.58	19.90	A0A0G2K0X9	Protein transport protein Sec31A	20	1.02	1.01	0.99	1.01	1.02	1.02143684	0.527
340	31.51	42.51	D3ZCV0	Actinin alpha 2	35	0.75	1.21	0.82	0.89	0.55	0.91383145	0.0202
341	31.48	53.28	Q5M7T6	ATPase H+-transporting V0 subunit D1	24	0.91	1.03	1.11	1.20	1.18	0.94317671	0.0425
342	31.45	48.77	P62198	26S protease regulatory subunit 8	20	0.97	0.89	0.74	1.13	1.00	0.99314068	0.35
343	31.4	16.62	F1LQT3	Rho-associated protein	24	1.36	1.33	0.86	1.26	0.95	0.97488233	0.242
344	31.32	27.26	A0A0G2K7J0	Long-chain-fatty-acid--CoA ligase ACSBG1	20	0.97	0.96	1.84	0.76	1.24	0.98910803	0.639
345	31.2	53.30	A0A0G2K654	Histone cluster 1 H1 family member c	18	1.00	1.22	1.42	1.02	1.36	1.03469101	0.507
346	31.13	32.69	D3ZCH7	Adducin 3 (Gamma), isoform CRA_b	19	1.17	0.88	1.28	1.18	1.28	0.97779233	0.176
347	30.97	20.73	G3V8A5	VPS35 retromer complex component	16	0.56	0.50	0.74	0.84	0.72	1.01143247	0.623
348	30.94	51.39	A0A0G2K5L2	Mitochondrial glutamate carrier 1-like	28	1.21	0.54	1.08	1.08	1.07	0.89564567	0.0587
349	30.93	42.69	O35077	Glycerol-3-phosphate dehydrogenase [NAD(+)], cytoplasmic	23	0.91	0.51	1.56	1.56	2.01	1.00758392	0.827
350	30.88	22.58	P13383	Nucleolin	13	0.88	1.82	2.29	0.33	1.72	1.03075396	0.52
351	30.78	36.04	A0A0H2UHQ0	4F2 cell-surface antigen heavy chain	27	0.83	1.45	1.74	1.87	1.43	1.09277774	0.133
352	30.75	33.62	O35112	CD166 antigen	19	1.98	0.82	0.74	1.39	0.92	0.8944049	0.162
353	30.74	30.68	P00388	NADPH--cytochrome P450 reductase	18	1.21	1.05	1.01	1.13	1.36	0.94822412	0.32
354	30.71	35.71	F1M907	Disks large homolog 2	21	0.86	1.58	1.06	0.99	1.43	0.97752127	0.191
355	30.69	19.25	D3Z981	Plexin A1	26	0.84	0.52	0.86	1.18	0.90	1.02030466	0.104
356	30.67	24.10	Q68FP1	Gelsolin	20	0.77	0.89	1.32	1.06	1.34	1.08447741	0.017
357	30.59	51.44	Q6P6G9	Heterogeneous nuclear ribonucleoprotein A1	19	1.06	1.06	1.02	1.03	0.99	1.11265012	0.0993
358	30.51	44.00	A0A0G2K9P4	Endophilin-B2	20	1.04	0.77	0.73	0.54	0.49	0.87721355	0.0929
359	30.44	61.47	P54311	Guanine nucleotide-binding protein G(I)/G(S)/G(T) subunit beta-1	38	1.38	0.04	1.07	0.87	1.04	0.94986869	0.705
360	30.38	20.73	Q04462	Valine--tRNA ligase	20	1.12	1.32	1.27	1.24	1.04	1.01255481	0.629
361	30.33	81.88	P62161	Calmodulin	59	1.36	1.84	1.79	0.12	1.66	0.7494998	0.297
362	30.27	40.63	Q64057	Alpha-aminoadipic semialdehyde	21	1.02	1.01	1.01	0.99	0.97	0.92787348	0.176
363	30.2	55.91	Q68FS2	C	20	0.72	0.98	0.87	0.85	0.65	0.9666731	0.067
364	30.13	19.87	Q5M7W5	Microtubule-associated protein 4	21	null	null	null	null	null	1.08522937	0.399
365	30.08	15.99	Q9JLA3	UDP-glucose:glycoprotein glucosyltransferase 1	19	1.08	1.33	1.37	1.29	0.92	0.99759767	0.921
366	30.07	44.87	A0A0G2K350	NSF attachment protein gamma	21	1.06	1.42	0.97	0.90	1.33	1.03440417	0.48
367	30.06	67.46	P81155	Voltage-dependent anion-selective channel protein 2	24	0.70	0.70	0.93	0.82	0.47	0.89254697	0.133
368	30.03	53.67	P04905	Glutathione S-transferase Mu 1	23	0.82	1.22	1.24	1.18	1.26	0.96633813	0.32
369	29.98	32.18	A0A0G2K719	DEAD-box helicase 3, X-linked	19	0.83	0.93	0.69	0.82	0.80	0.99873235	0.843
370	29.88	42.78	Q5HZV9	Protein phosphatase 1 regulatory subunit 7	20	1.27	1.61	1.00	0.80	1.34	0.94697615	0.214
371	29.78	43.52	P10760	Adenosylhomocysteinase	20	1.09	0.97	1.60	1.12	1.27	1.00926148	0.81
372	29.78	61.36	M0R416	Pyridoxal phosphate phosphatase	18	1.27	0.86	0.79	0.72	0.67	0.95528294	0.46
373	29.77	55.00	A0A0G2K9A2	Arp2/3 complex 34 kDa subunit	20	1.26	0.98	1.29	1.61	2.00	1.0179735	0.486
374	29.76	21.72	D4A1Q2	Microtubule-associated protein	18	1.20	1.91	0.98	1.00	1.71	0.92146419	0.26
375	29.72	37.89	Q68FZ8	Propionyl coenzyme A carboxylase, beta	16	1.21	1.39	0.99	1.06	1.00	0.91827616	0.0353

376	29.68	16.61	B5DFK6	Adaptor-related protein complex 3, delta 1 subunit	16	1.07	0.56	0.65	0.74	0.95	1.04008397	0.126
377	29.62	32.91	H1UBN0	Copine-7	17	0.78	1.09	0.48	0.60	0.42	0.88209137	0.0522
378	29.62	40.00	Q6Q0N1	Cytosolic non-specific dipeptidase	17	0.60	1.07	1.50	1.20	1.64	1.00870198	0.616
379	29.61	22.73	F1LML7	Huntingtin-interacting protein 1-related	19	0.84	1.11	1.07	0.97	1.13	1.02733023	0.0111
380	29.61	25.55	Q4FZT9	26S proteasome non-ATPase regulatory subunit 2	19	1.03	1.17	1.21	1.20	1.13	0.93109482	0.052
381	29.52	15.35	F1M4A4	Kinesin family member 1A	21	0.98	0.95	0.96	0.95	0.95	0.99151053	0.656
382	29.47	20.96	P41542	General vesicular transport factor p115	17	1.06	1.27	1.10	1.20	1.39	1.02044611	0.26
383	29.45	37.08	G3V741	Phosphate carrier protein, mitochondrial	24	0.49	1.17	1.21	1.96	1.43	0.98261625	0.606
384	29.41	14.18	Q63372	Neurexin-1	18	1.09	1.06	1.21	0.95	0.95	1.02391787	0.294
385	29.39	72.54	A0A0G2JSV6	Globin c2	63	1.13	1.29	1.39	1.79	2.86	1.29953906	0.184
386	29.26	14.76	D3ZDX5	Plexin B1	21	1.03	0.99	1.04	0.99	0.99	0.96392981	0.165
387	29.17	45.13	G3V7L8	ATPase, H+ transporting, V1 subunit E isoform 1, isoform CRA_a	33	1.45	1.57	1.28	0.90	2.07	0.9554816	0.0366
388	29.15	32.15	P10362	Secretogranin-2	20	0.75	0.74	0.53	0.16	0.62	0.92338231	0.0647
389	28.96	43.62	Q6PCU0	ATP synthase subunit	26	0.64	1.13	1.21	1.47	0.74	0.99992376	0.998
390	28.88	23.31	A0A0G2JSR7	Matrin-3	24	1.08	1.25	1.31	0.98	1.07	1.0412381	0.218
391	28.84	45.64	A0A0G2K9L2	Target of myb1-like 2 membrane-trafficking	21	1.14	1.56	0.79	0.64	0.79	0.8962667	0.00107
392	28.83	15.54	Q3KRF2	High density lipoprotein binding protein (Vigilin)	17	0.71	1.29	1.26	1.25	1.07	0.98842267	0.271
393	28.81	70.08	P25113	Phosphoglycerate mutase 1	34	1.33	1.15	2.58	2.03	2.40	0.98698496	0.381
394	28.78	18.30	A0A0G2JUG7	IQ motif and Sec7 domain 1	16	0.80	1.19	1.11	1.17	1.47	1.02405983	0.416
395	28.71	30.99	E9PSS1	Serine/threonine-protein kinase DCLK2	18	0.97	0.82	1.08	1.50	1.57	1.00863207	0.825
396	28.68	30.49	Q5FVJ0	Protein RUFY3	15	1.10	1.24	1.01	0.97	1.06	0.96781285	0.0499
397	28.65	71.86	P37805	Transgelin-3	26	0.90	0.58	1.89	0.98	1.77	1.00779346	0.901
398	28.49	29.59	F1M5N4	Malic enzyme	16	1.80	2.23	1.69	2.73	2.86	1.00849225	0.666
399	28.48	50.82	A0A0G2K5U9	Alpha-actinin-4	46	1.06	1.10	1.11	1.08	1.03	0.91763988	0.122
400	28.44	23.07	F1LN92	AFG3-like matrix AAA peptidase subunit 2	17	0.77	0.89	1.16	1.10	0.93	0.98986247	0.259
401	28.37	30.95	Q60587	Trifunctional enzyme subunit beta, mitochondrial	16	0.28	0.90	1.09	1.02	1.12	1.13997327	0.0891
402	28.32	39.75	A0A0G2K9J2	ATPase H+-transporting V1 subunit H	27	0.62	0.96	0.88	1.45	1.13	0.97948819	0.289
403	28.31	42.06	D3ZDH8	Platelet glycoprotein Ib beta chain	25	1.17	1.21	0.46	0.88	0.87	0.96821544	0.446
404	28.25	45.41	G3V6W6	Proteasome 26S subunit, ATPase 6	21	1.04	1.01	1.01	1.00	1.05	0.97211574	0.324
405	28.23	11.34	A0A0G2KAH9	Inositol 1,4,5-trisphosphate receptor type 1	23	1.19	1.18	0.95	1.54	1.47	0.97576119	0.314
406	28.2	26.92	D3ZAI6	5'-nucleotidase domain-containing 3	16	0.58	1.75	0.64	1.38	1.05	0.9418701	0.332
407	28.16	43.26	Q5XIJ3	Isocitrate dehydrogenase [NAD] subunit,	25	0.64	0.97	1.07	1.39	1.17	0.94245785	0.0531
408	28.14	53.24	Q561S0	NADH dehydrogenase [ubiquinone] 1 alpha subcomplex subunit 10, mitochondrial	18	0.82	1.13	1.20	0.90	0.90	0.93316235	0.0305
409	28.13	36.96	P20059	Hemopexin	16	3.31	0.90	1.07	0.90	1.22	0.83508792	0.125
410	28.12	36.06	P18418	Calreticulin	17	0.95	0.95	1.45	0.58	1.25	0.98397939	0.879
411	27.98	16.97	F1M2K6	Plakophilin 4	19	1.20	1.58	1.16	1.60	0.92	1.0179735	0.359
412	27.98	43.15	Q63610	Tropomyosin alpha-3 chain	19	1.24	1.07	0.98	0.20	0.75	0.90563398	0.327
413	27.97	26.63	M0R961	Far upstream element-binding protein 2	15	1.01	1.05	1.27	0.87	1.18	0.9641971	0.119
414	27.93	63.97	P67779	Prohibitin	21	0.87	1.64	1.27	1.43	1.27	0.97779233	0.267
415	27.93	14.29	O08873	MAP kinase-activating death domain protein	18	0.90	0.96	0.77	0.63	0.61	0.97779233	0.397
416	27.87	22.82	F1M8H6	Phosphatidylinositol 4-phosphate 5-kinase type-1 gamma	18	1.01	0.77	0.68	1.03	1.26	0.92787348	0.0569
417	27.82	31.40	F7EN52	Cyp46a1 protein	15	0.94	0.91	0.68	0.75	0.75	1.0435502	0.667
418	27.81	49.19	Q99PD4	Actin-related protein 2/3 complex subunit 1A	18	1.46	0.54	1.54	2.17	2.07	0.97097092	0.415
419	27.74	21.54	F1LMR7	Dipeptidyl aminopeptidase-like protein 6	17	1.01	0.82	1.16	1.34	1.19	1.03118273	0.604
420	27.7	27.54	O08629	Transcription intermediary factor 1-beta	15	1.19	1.13	1.18	1.01	1.32	1.00653686	0.637
421	27.68	67.88	P84076	Neuron-specific calcium-binding protein hippocalcin	20	1.33	0.47	0.51	0.70	2.03	0.96052828	0.77
422	27.67	51.25	Q91Y81	Septin-2	23	1.17	0.97	1.69	0.89	1.11	0.99240438	0.825
423	27.64	19.82	A0A0G2JTN4	Uncharacterized protein	17	1.21	1.09	1.22	1.42	0.88	0.97366676	0.428
424	27.63	53.03	P62138	Serine/threonine-protein phosphatase PP1-alpha catalytic subunit	16	0.90	1.12	0.98	0.93	1.02	0.97928453	0.713

425	27.57	28.77	Q9EPH8	Polyadenylate-binding protein 1	18	1.00	1.01	1.01	0.99	1.04	0.91573369	0.0356
426	27.54	80.72	P45592	Cofilin-1	20	0.83	0.22	2.05	1.19	2.47	1.20163605	0.0594
427	27.53	24.80	D3Z955	Phosphoglucomutase 2-like	15	0.86	0.91	1.17	1.25	1.21	1.0369167	0.0454
428	27.5	24.88	Q499N5	Acyl-CoA synthetase family member 2, mitochondrial	15	1.36	0.85	0.89	1.02	0.96	0.92146419	0.0146
429	27.44	48.13	G3V8Q6	Protein kinase, cAMP-dependent, regulatory, type 2, alpha, isoform CRA_a	18	0.83	0.74	0.87	0.49	0.58	0.92466328	0.0914
430	27.42	36.24	G3V836	Clusterin	17	0.91	1.19	6.55	1.42	3.25	1.20080343	0.131
431	27.33	18.20	P24155	Thimet oligopeptidase	12	1.16	1.26	1.14	0.99	1.08	0.97204836	0.143
432	27.29	52.35	P14668	Annexin A5	17	1.36	1.96	4.02	1.54	1.89	1.0807254	0.515
433	27.28	29.65	P41499	Tyrosine-protein phosphatase non-receptor	15	1.58	1.91	1.25	1.19	1.33	1.00702535	0.657
434	27.28	23.01	P35565	Calnexin	16	1.05	1.34	1.54	1.24	1.32	1.00926148	0.277
435	27.23	38.63	A0A0G2JSI1	4-trimethylaminobutyraldehyde dehydrogenase	20	0.81	1.28	1.63	2.05	1.08	1.02235766	0.504
436	27.21	52.56	O35331	Pyridoxal kinase	19	0.74	0.82	0.93	0.59	0.53	0.96346222	0.331
437	27.2	36.88	Q91ZN1	Coronin-1A	18	1.27	1.45	1.66	1.25	1.58	0.97494991	0.282
438	27.17	30.22	Q5XI77	Annexin	16	1.13	0.49	0.98	1.15	1.21	1.03426078	0.0127
439	27.17	20.50	G3V864	Phospholipid phosphatase-related protein type 4	20	0.95	1.37	0.92	1.29	1.16	0.98910803	0.769
440	27.11	32.38	Q5BJU7	Wiskott-Aldrich syndrome protein family member 1	18	2.19	1.66	1.46	1.27	2.61	0.92018765	0.154
441	27.04	33.10	A0A0G2JUL7	Septin 6 (Predicted), isoform CRA_b	18	1.41	1.43	1.39	1.34	1.47	1.01494391	0.53
442	26.98	33.55	P07340	Sodium/potassium-transporting ATPase subunit beta-1	33	0.40	0.45	2.00	1.89	1.67	1.03340087	0.526
443	26.9	48.40	Q5RKI1	Eukaryotic initiation factor 4A-II	22	2.21	1.17	1.19	2.47	1.13	0.98452517	0.665
444	26.84	12.46	Q9WV48	SH3 and multiple ankyrin repeat domains protein 1	19	1.02	1.03	1.00	1.02	0.99	1.00135255	0.934
445	26.73	43.84	A0A0G2K2V2	AP complex subunit beta	46	1.00	1.02	1.02	0.99	1.04	0.98275248	0.478
446	26.71	16.43	G3V7K3	Ceruloplasmin	15	1.45	0.96	1.57	1.19	1.10	0.96989468	0.624
447	26.68	57.75	P04897	Guanine nucleotide-binding protein G(i) subunit alpha-2	34	0.79	0.46	1.26	0.95	1.11	0.96426394	0.306
448	26.61	59.79	Q3T1K5	F-actin-capping protein subunit alpha-2	20	1.33	1.45	1.22	1.46	1.89	1.03018254	0.222
449	26.54	51.68	Q5I0D1	Glyoxalase domain-containing protein 4	16	1.00	1.14	1.18	0.85	1.10	0.93698639	0.25
450	26.49	24.04	G3V7G8	Glycine--tRNA ligase	15	0.76	0.84	0.71	0.74	0.95	0.93653187	0.0922
451	26.48	22.89	D3ZIE9	Aldehyde dehydrogenase 18 family, member A1	14	1.01	0.98	0.97	0.98	0.99	1.10266916	0.477
452	26.44	34.84	Q4QQV4	Dead end homolog 1 (Zebrafish)	15	0.94	0.65	1.17	0.97	0.99	1.01966836	0.217
453	26.44	63.40	P39069	Adenylate kinase isoenzyme 1	17	0.93	0.74	1.64	1.21	1.71	1.01762076	0.613
454	26.42	26.77	Q68FR6	Elongation factor 1-gamma	13	1.00	1.63	1.54	1.46	1.26	0.98071102	0.586
455	26.33	21.99	G3V982	Engulfment and cell motility 2	13	1.03	1.31	1.14	1.58	1.24	0.99446328	0.843
456	26.33	50.71	P21913	Succinate dehydrogenase [ubiquinone] iron-sulfur subunit, mitochondrial	17	0.82	1.21	0.98	1.39	1.21	1.01213379	0.741
457	26.33	45.79	Q9Z1E1	Flotillin-1	18	0.99	0.94	0.89	0.95	0.97	1.02733023	0.464
458	26.32	28.57	Q64548	Reticulon-1	21	1.64	1.04	0.95	0.60	1.39	0.92851685	0.043
459	26.3	24.31	D3ZJR1	Epidermal growth factor receptor pathway substrate 15-like 1	15	0.84	0.79	0.90	0.70	0.72	1.00891176	0.752
460	26.27	85.45	Q05175	Brain acid soluble protein 1	17	0.46	1.09	0.82	1.05	0.78	0.97124017	0.517
461	26.12	25.72	B2GV74	Kinesin light chain 2	13	1.03	0.97	1.03	1.04	1.00	0.94723875	0.0797
462	26.11	70.42	Q06647	ATP synthase subunit	30	0.72	1.25	1.18	1.37	1.14	0.9665391	0.338
463	26.08	31.19	Q499T3	Sirpa protein	16	1.24	0.64	0.57	1.31	0.96	0.95740417	0.147
464	26.07	46.65	Q9QYU4	Ketimine reductase mu-crystallin	21	1.31	2.17	0.79	0.94	0.60	0.63993621	0.0737
465	26.05	38.38	Q6DGG0	Peptidyl-prolyl cis-trans isomerase D	13	1.36	1.36	1.32	1.16	1.13	0.93952275	0.0217
466	26.03	42.74	A0A0G2JXA8	Flotillin 2, isoform CRA_d	15	1.46	1.29	1.27	0.83	1.01	0.91891288	0.0198
467	25.98	19.28	D3ZBL5	Type I inositol 3,4-bisphosphate 4-phosphatase	15	0.98	1.24	0.97	0.85	0.96	0.98862823	0.482
468	25.97	40.96	P17105	Inositol-trisphosphate 3-kinase A	14	1.31	1.27	0.89	1.41	1.12	0.99233559	0.813
469	25.92	42.34	P82471	Guanine nucleotide-binding protein G(q) subunit alpha	14	1.22	0.80	0.90	0.90	1.15	0.95019795	0.103
470	25.88	23.52	Q9Z327	Synaptopodin	15	1.16	1.28	0.82	0.85	0.78	0.93174043	0.0629
471	25.84	18.57	G3V8B6	26S proteasome non-ATPase regulatory subunit 1	16	1.07	0.99	1.27	0.94	1.06	1.02768634	0.179
472	25.83	53.46	B2RYJ7	ARP1 actin-related protein 1 homolog B (Yeast)	18	0.88	0.95	0.80	0.71	0.95	0.95237391	0.329
473	25.83	61.43	P37377	Alpha-synuclein	21	1.21	2.33	1.85	0.33	1.75	0.75838377	0.254

474	25.82	55.25	P14669	Annexin A3	17	0.87	1.19	4.37	1.02	0.97	1.04015606	0.534
475	25.8	32.51	Q64568	Plasma membrane calcium-transporting ATPase 3	51	0.74	0.52	0.63	0.82	0.65	0.94520555	0.215
476	25.78	36.32	P12007	Isovaleryl-CoA dehydrogenase,	20	0.68	1.05	1.11	1.12	1.03	0.98732708	0.455
477	25.73	21.90	P97852	Peroxisomal multifunctional enzyme type 2	14	0.75	1.05	1.63	1.10	1.15	0.97867381	0.387
478	25.63	31.97	Q5XIT9	Methylcrotonoyl-CoA carboxylase beta chain, mitochondrial	15	0.67	0.77	0.52	0.82	0.65	0.95296821	0.219
479	25.61	35.18	Q6AXV4	Sorting and assembly machinery component 50 homolog	16	0.90	0.73	0.79	1.10	0.68	0.99360201	0.704
480	25.6	28.48	Q6GMM8	Long-chain fatty acid transport protein 1	15	1.04	0.72	1.07	1.14	0.63	0.9694914	0.291
481	25.59	44.48	P14604	Enoyl-CoA hydratase, mitochondrial	19	1.04	1.18	1.34	1.53	1.84	1.02647607	0.0692
482	25.58	21.54	F7EPE0	Prosaposin	13	0.79	0.04	1.31	0.52	0.88	1.07326029	0.468
483	25.56	28.87	F1LS42	Protein kinase C	16	0.72	0.74	1.07	1.58	1.39	1.11265012	0.19
484	25.52	33.49	Q5U2X8	Acyl-CoA thioesterase 9	14	0.71	1.17	1.12	1.42	1.33	1.02292474	0.43
485	25.5	41.41	P19511	ATP synthase F(0) complex subunit B1, mitochondrial	22	0.99	1.08	1.00	1.56	0.96	0.95740417	0.29
486	25.49	28.25	Q2PQA9	Kinesin-1 heavy chain	26	1.00	0.97	1.01	0.99	1.00	1.00398659	0.851
487	25.44	27.14	F1LPK7	Plastin 3 (T-isoform), isoform CRA_a	13	0.76	1.03	1.29	1.07	1.41	0.98931373	0.504
488	25.41	36.43	Q6P6U2	26S protease regulatory subunit 6A	18	0.83	0.97	0.77	0.50	0.95	0.90187538	0.0541
489	25.39	24.23	P70483	Striatin	15	1.08	1.08	1.01	1.01	1.01	0.91636865	0.0734
490	25.39	60.68	P60881	Synaptosomal-associated protein 25	35	1.02	1.38	0.88	1.01	1.20	0.88515377	0.0196
491	25.27	13.85	A0A0G2K6T9	Protocadherin 1	15	0.99	1.09	0.87	1.01	1.38	0.86934456	0.192
492	25.22	30.69	P60203	Myelin proteolipid protein	47	2.05	0.22	2.25	2.83	4.02	1.22518533	0.274
493	25.2	58.56	P13803	Electron transfer flavoprotein subunit alpha,	15	0.46	0.73	1.03	0.76	1.12	1.06149678	0.269
494	25.2	71.01	P09527	Ras-related protein Rab-7a	22	0.92	0.96	1.15	1.07	1.01	0.98268436	0.566
495	25.11	35.52	F1LT49	Leucine-rich repeat-containing 47	14	0.63	0.69	0.84	0.84	0.78	1.05914492	0.245
496	25.09	38.41	P41562	Isocitrate dehydrogenase [NADP] cytoplasmic	16	0.66	0.77	1.61	1.18	1.36	1.1188371	0.0257
497	25.06	51.01	Q6P503	ATPase H+-transporting V1 subunit D	18	0.93	1.25	0.81	0.84	0.85	1.10880064	0.132
498	25.02	27.20	A0A0G2JWX1	26S proteasome non-ATPase regulatory subunit	13	0.97	0.98	0.96	1.01	0.95	0.99928631	0.967
499	24.94	75.11	Q4QRB4	Tubulin beta-3 chain	279	1.28	0.19	0.93	0.69	0.77	0.9666061	0.61
500	24.87	84.66	O88767	Protein DJ-1	19	1.04	0.74	1.60	0.68	1.75	1.0546761	0.101
501	24.83	43.89	X1WI37	40S ribosomal protein S4	13	1.15	1.51	1.74	1.47	1.64	0.99144181	0.669
502	24.78	25.16	A0A0G2K7K2	Apoptosis-inducing factor 1, mitochondrial	15	0.98	1.04	0.98	0.99	1.02	1.03504967	0.0473
503	24.77	37.68	Q4V8H5	Aspartyl aminopeptidase	16	1.01	1.00	0.98	0.96	1.00	0.94350365	0.0574
504	24.74	25.95	O35274	Neurabin-2	15	0.86	1.26	1.26	0.74	1.42	0.97569355	0.215
505	24.73	61.54	P27139	Carbonic anhydrase 2	19	0.58	0.65	1.42	2.47	2.27	1.11265012	0.0732
506	24.71	19.18	A0A0G2JZ83	ArfGAP with GTPase domain, ankyrin repeat and PH domain 3	15	1.13	1.12	0.89	1.31	0.88	0.97969189	0.439
507	24.65	38.64	Q07936	Annexin A2	12	1.04	1.87	1.49	3.50	2.75	1.06223281	0.0448
508	24.65	20.90	P26817	Beta-adrenergic receptor kinase 1	13	0.82	0.90	0.92	0.79	0.72	1.02733023	0.452
509	24.64	27.18	A0A0G2JV31	X-prolyl aminopeptidase (Aminopeptidase P) 1, soluble, isoform CRA_a	15	1.02	1.13	1.29	0.99	1.21	0.90312651	0.0341
510	24.58	61.73	A0A0G2JSV0	N-myc downstream regulated gene 2, isoform	16	1.61	1.50	1.92	1.17	1.32	0.97434189	0.627
511	24.56	21.98	D3ZGE6	Cortactin, isoform CRA_c	15	0.89	1.32	1.05	0.90	1.38	0.99656789	0.908
512	24.56	45.28	A0A0H2UHE1	Succinate--CoA ligase [ADP/GDP-forming] subunit alpha, mitochondrial	19	0.48	0.52	0.60	0.97	0.96	0.981255	0.821
513	24.55	61.63	P68255	14-3-3 protein theta	36	1.32	0.98	1.36	1.45	1.69	0.97252012	0.526
514	24.5	33.08	P97546	Neuroplastin	23	1.38	0.72	0.99	1.41	1.24	1.00842235	0.91
515	24.46	24.79	Q05683	Glutamate decarboxylase 2	23	0.56	0.07	0.74	0.56	0.88	1.01515498	0.817
516	24.45	17.09	B2GUZ3	Methylenetetrahydrofolate dehydrogenase (NADP+-dependent) 1-like	16	0.87	1.13	0.69	0.91	0.74	0.96433078	0.317
517	24.43	29.72	M0R735	Heterogeneous nuclear ribonucleoprotein Q	16	1.17	1.45	1.47	1.51	1.22	0.98336574	0.366
518	24.41	42.42	Q568Z9	Phytanoyl-CoA hydroxylase-interacting	13	1.49	0.33	0.87	1.27	1.58	1.04514275	0.311
519	24.38	76.63	Q62636	Ras-related protein Rap-1b	21	1.31	0.21	1.74	1.22	1.71	1.03146867	0.422
520	24.38	18.13	A0A0G2JZZ7	Active BCR-related	13	1.01	1.00	0.98	1.03	1.02	0.9815271	0.387
521	24.31	18.75	D4A8H8	Cytoplasmic FMR1 interacting protein 1 (Predicted)	29	0.88	1.46	1.18	0.95	1.53	0.96848392	0.281

522	24.3	23.36	P13676	Acylamino-acid-releasing enzyme	12	0.88	1.26	1.07	1.13	1.15	0.97684394	0.578
523	24.24	21.70	A0A0G2K8K(	Uncharacterized protein	15	1.69	1.36	1.45	0.73	1.37	0.94376528	0.223
524	24.24	57.40	P47727	Carbonyl reductase [NADPH] 1	13	0.63	0.69	0.88	0.52	1.04	0.95422408	0.473
525	24.17	66.44	P85973	Purine nucleoside phosphorylase	16	1.07	0.69	1.75	1.10	1.58	1.07997656	0.0792
526	24.13	14.93	D4AE96	Importin 7	15	0.86	0.96	0.65	0.63	0.77	1.02498301	0.188
527	24.03	36.89	Q9Z214	Homer protein homolog 1	14	0.89	1.58	1.43	0.89	1.38	0.93044966	0.163
528	24	23.17	G3V7U4	Lamin-B1	14	1.11	1.45	1.29	0.91	0.86	1.00352044	0.721
529	23.97	13.77	Q5U2N3	Membrane-associated phosphatidylinositol transfer protein 1	15	0.84	0.78	0.86	0.71	0.59	0.96902111	0.152
530	23.96	61.88	P84079	ADP-ribosylation factor 1	32	1.16	1.31	1.12	1.04	1.15	0.91573369	0.087
531	23.88	12.00	D4A4Z9	Kinectin 1	13	1.19	1.28	1.56	0.97	1.28	1.01522535	0.539
532	23.87	50.79	A0A0G2JUX5	Transcriptional activator protein Pur-beta	14	1.56	1.16	1.53	1.27	1.54	0.9379611	0.0841
533	23.84	74.39	P10111	Peptidyl-prolyl cis-trans isomerase A	25	0.90	0.26	1.38	1.08	1.89	1.20246925	0.242
534	23.77	67.96	F1LP82	Ras-related protein Rab-2A	19	0.85	1.05	0.81	1.17	0.96	0.94284989	0.148
535	23.64	20.46	P18163	Long-chain-fatty-acid--CoA ligase 1	14	1.09	0.61	1.16	0.88	1.32	0.99417381	0.727
536	23.56	7.68	F1M0Z1	Triple functional domain protein	19	0.99	0.94	1.04	1.28	1.27	1.04804452	0.0365
537	23.54	50.31	A0A0G2JSV3	Ribose-phosphate pyrophosphokinase I -like	14	1.82	0.65	1.72	1.13	1.47	0.9583337	0.382
538	23.44	20.31	M0R3V7	Sodium/calcium exchanger	16	0.75	0.90	0.76	1.20	1.37	1.00176909	0.639
539	23.42	40.00	D3Z9H2	Hyaluronan and proteoglycan link protein 4	14	0.64	0.46	0.78	0.83	1.50	1.00177603	0.976
540	23.42	44.32	P49242	40S ribosomal protein S3a	14	1.05	0.84	1.43	1.10	1.43	0.97603176	0.43
541	23.37	38.65	A0A0G2JSZ5	Protein disulfide-isomerase A6	17	1.10	0.91	1.14	1.01	1.20	0.97258753	0.0819
542	23.35	36.91	Q6AYI1	DEAD (Asp-Glu-Ala-Asp) box polypeptide 5	22	0.66	0.66	1.19	0.96	1.09	0.9975631	0.881
543	23.35	49.53	P63245	Receptor of activated protein C kinase 1	12	0.80	1.01	1.36	1.34	1.26	1.06319041	0.0352
544	23.3	25.56	G3V6Z5	Glutamate receptor 3	19	1.24	1.00	0.96	1.75	1.71	1.029112	0.573
545	23.25	36.36	A0JPJ7		15	0.80	1.26	0.99	1.22	1.32	1.12974722	0.0388
546	23.23	38.27	P04182		14	0.82	1.05	0.74	0.92	0.75	1.01374866	0.717
547	23.2	34.15	P63004	Platelet-activating factor acetylhydrolase IB subunit alpha	15	1.16	0.90	1.60	1.67	1.50	1.00411882	0.269
548	23.08	27.41	Q3KRE0	ATPase family AAA domain-containing protein 3	17	1.17	1.34	0.90	1.25	1.33	0.98636944	0.514
549	23.05	23.12	Q6PST4	Atlastin-1	14	0.90	1.10	0.80	1.15	0.90	0.9507909	0.0853
550	23.01	38.71	F7FG31	C-terminal-binding protein	16	1.19	1.00	0.64	0.69	0.77	0.94311134	0.18
551	22.96	37.84	Q8VI04	Isoaspartyl peptidase/L-asparaginase	15	1.47	0.84	2.61	1.79	1.66	1.05482232	0.107
552	22.94	21.39	G3V918	Trifunctional purine biosynthetic protein adenosine-3	13	1.05	1.04	0.98	0.90	1.03	1.05372617	0.0433
553	22.94	23.07	G3V803	Cadherin-2	15	0.95	1.04	1.12	1.28	1.18	0.97589646	0.255
554	22.91	12.31	P97846	Contactin-associated protein 1	16	1.12	0.72	0.97	1.05	1.07	0.95019795	0.185
555	22.89	38.53	P0DMW1	Heat shock 70 kDa protein 1B	43	0.95	1.14	0.75	1.16	1.14	1.01656326	0.713
556	22.89	32.68	Q6P6T6	Cathepsin D	14	0.55	0.19	1.32	0.81	1.20	1.10266916	0.271
557	22.84	43.30	F1LMH0	Neuronal-specific septin-3	13	0.91	0.85	0.96	1.21	1.36	0.97623474	0.473
558	22.79	35.80	B2GUZ9	Fam49b protein	19	1.26	0.78	1.29	0.81	1.22	1.00364565	0.9
559	22.76	66.37	Q00981	Ubiquitin carboxyl-terminal hydrolase isozyme L1	27	1.16	0.72	2.00	0.24	1.87	1.00961133	0.933
560	22.76	7.04	Q62656	Receptor-type tyrosine-protein phosphatase zeta	21	1.19	0.47	2.73	1.42	2.13	1.07251662	0.034
561	22.7	41.99	Q5XIT1	Microtubule-associated protein RP/EB family member 3	18	1.19	1.07	0.95	1.19	0.98	0.97231791	0.398
562	22.65	31.11	F1LU71	AU RNA binding protein/enoyl-coenzyme A hydratase (Predicted), isoform CRA_a	13	0.69	0.79	0.61	0.82	0.97	0.93109482	0.0174
563	22.61	13.00	A0A0H2UHR'	Filamin-C	24	1.12	0.90	2.29	1.84	1.96	1.02747266	0.0277
564	22.61	92.51	P31044	Phosphatidylethanolamine-binding protein 1	26	0.95	0.90	1.07	0.31	1.46	0.85263489	0.126
565	22.56	34.70	G3V6U3	ALG2, alpha-1,3/1,6-mannosyltransferase	13	0.72	0.74	0.74	0.78	0.97	0.96600329	0.286
566	22.55	28.65	P19643	Amine oxidase [flavin-containing] B	15	1.49	1.57	4.02	2.68	3.56	1.08372597	0.117
567	22.46	42.31	B0K031	60S ribosomal protein L7	12	0.78	1.33	1.29	1.43	1.43	1.01860874	0.118
568	22.43	52.27	F1LNF1	Heterogeneous nuclear ribonucleoproteins A2/B1	19	0.95	1.89	1.66	1.28	1.54	1.09050773	0.107

569	22.43	52.45	Q5XI73	Rho GDP-dissociation inhibitor 1	16	2.54	1.75	3.80	1.89	3.34	0.97609942	0.502
570	22.42	13.61	Q02563	Synaptic vesicle glycoprotein 2A	18	1.03	0.98	1.03	0.98	0.99	0.94875008	0.104
571	22.4	39.77	F1LQY6	EF hand calcium binding protein 2	11	1.20	1.01	0.77	0.60	0.81	0.88576752	0.0142
572	22.34	18.67	Q80U96	Exportin-1	15	1.03	0.94	0.96	0.95	0.96	0.99733494	0.906
573	22.34	31.71	P47971	Neuronal pentraxin-1	13	1.13	1.08	0.63	0.82	1.00	0.90751916	0.378
574	22.31	7.31	B0LPN4	Ryanodine receptor 2	28	1.13	1.33	1.29	1.26	1.63	0.99602924	0.602
575	22.29	16.72	P28818	Ras-specific guanine nucleotide-releasing factor 1	20	0.91	0.77	0.78	1.00	0.69	0.99212926	0.561
576	22.27	10.78	Q1JU68	Eukaryotic translation initiation factor 3 subunit A	13	0.95	0.99	0.95	1.02	0.98	1.07400447	0.25
577	22.25	28.92	Q08602	Geranylgeranyl transferase type-2 subunit alpha	15	1.14	0.49	0.90	0.54	0.79	0.97231791	0.0869
578	22.21	14.01	Q5PPJ6	Leucyl-tRNA synthetase	13	1.17	1.28	0.82	1.19	0.85	1.03562378	0.245
579	22.2	59.27	P19234	NADH dehydrogenase [ubiquinone] flavoprotein 2, mitochondrial	18	0.69	0.78	0.72	0.44	0.84	0.94004388	0.166
580	22.2	40.05	F8WG67	Acyl-CoA thioesterase 7, isoform CRA_a	15	0.98	0.90	0.92	0.82	1.02	0.97090362	0.0582
581	22.12	36.88	Q3MIE4	Synaptic vesicle membrane protein VAT-1 homolog	15	0.74	0.35	0.71	0.61	0.58	0.94010904	0.401
582	22.08	18.68	G3V9M3	RCG28460	11	1.16	0.84	0.63	0.91	1.08	1.03082541	0.198
583	22.05	27.51	Q64542	Plasma membrane calcium-transporting ATPase 4	42	1.74	0.65	0.71	0.25	0.60	0.91955005	0.342
584	22.04	20.29	D3Z941	Methionyl-tRNA synthetase	13	0.97	0.90	0.83	0.95	0.75	1.02087059	0.152
585	22.03	45.74	P19945	60S acidic ribosomal protein P0	12	0.98	1.02	1.34	1.27	1.12	0.99116696	0.57
586	22.03	62.45	B0BMW2	3-hydroxyacyl-CoA dehydrogenase type-2	14	0.63	1.25	0.75	0.60	0.55	0.93867654	0.138
587	22.02	42.78	P62483	Voltage-gated potassium channel subunit beta-2	14	1.11	0.87	0.72	1.12	1.09	0.93303299	0.0964
588	22.01	61.16	O35244	Peroxiredoxin-6	18	1.20	1.74	2.88	0.95	1.94	1.07922824	0.502
589	21.97	86.18	Q05982	Nucleoside diphosphate kinase A	15	1.36	0.86	1.15	0.65	1.16	1.09202055	0.27
590	21.93	24.83	A0A140TAC3	Epsin-1	14	1.21	0.90	0.89	0.79	0.94	1.16877725	0.238
591	21.92	31.49	Q6AYT3	tRNA-splicing ligase RtcB homolog	14	0.94	0.86	1.05	0.69	1.24	1.02207425	0.466
592	21.89	68.12	A0A0H2UHV	Calcineurin subunit B type 1	21	1.67	0.52	0.97	0.72	2.51	0.97393676	0.79
593	21.86	43.24	Q6AY84	Secernin-1	16	0.82	0.67	0.95	0.52	1.09	0.9382212	0.245
594	21.85	40.27	D3ZC63	Cytidine/uridine monophosphate kinase 2	13	0.82	0.70	1.32	0.70	0.86	0.93685651	0.181
595	21.82	23.73	A0A140TAA1	Immunoglobulin superfamily, member 8	17	0.96	0.58	1.12	1.43	1.53	1.02562263	0.581
596	21.8	26.16	G3V624	Coronin	14	1.74	1.25	2.54	1.37	1.92	1.01726814	0.502
597	21.8	37.37	P24329	Thiosulfate sulfurtransferase	17	0.98	0.89	1.67	1.53	1.14	1.03806732	0.22
598	21.78	24.84	Q6IRJ7	Annexin	12	0.99	0.97	0.97	0.99	0.95	0.94284989	0.129
599	21.76	12.04	M0R5U4	Adenylate cyclase 9	12	1.10	1.06	0.79	1.21	1.24	1.00310317	0.794
600	21.72	60.57	P68511	14-3-3 protein eta	35	1.02	1.66	1.39	1.92	1.31	0.97589646	0.593
601	21.71	17.73	D3ZWX4	NCK-interacting protein with SH3 domain	13	1.29	1.08	0.75	0.96	0.99	0.97339684	0.0921
602	21.69	91.43	P62963	Profilin-1	18	1.19	0.58	1.53	1.46	2.05	1.09126388	0.137
603	21.66	28.48	P07943	Aldose reductase	11	0.86	1.27	1.74	1.32	1.54	1.04601243	0.0969
604	21.65	50.53	Q9R1Z0	Voltage-dependent anion-selective channel protein 3	20	0.80	0.70	0.72	0.78	0.73	0.90437938	0.0838
605	21.63	26.45	A0A0G2JYN7	Paraspeckle component 1	11	1.05	1.03	1.05	0.97	1.04	0.98993109	0.744
606	21.59	41.14	P62193	26S protease regulatory subunit 4	19	0.97	0.98	0.98	0.95	0.97	0.98677974	0.519
607	21.58	9.36	F1M949	Cytoskeleton-associated protein 5	14	1.04	1.04	1.05	1.05	1.06	1.03289958	0.19
608	21.54	64.60	Q63965	Sideroflexin-1	25	1.00	1.21	0.58	0.83	0.75	0.87539133	0.038
609	21.53	49.64	P20788	Cytochrome b-c1 complex subunit Rieske,	12	1.05	1.72	1.20	1.51	1.17	0.91066983	0.0177
610	21.53	26.50	A0A0G2JTG7	Heterogeneous nuclear ribonucleoprotein H	17	0.93	1.06	0.85	1.05	1.02	0.98890237	0.452
611	21.51	24.70	F1LSY2	Neuronal pentraxin receptor	15	1.25	0.66	1.37	0.81	1.33	0.98071102	0.449
612	21.51	14.63	Q9Z1T4	Connector enhancer of kinase suppressor of ras 2	12	1.15	1.29	0.91	1.28	1.45	0.99671987	0.917
613	21.49	77.27	P07632	Superoxide dismutase [Cu-Zn]	15	0.97	0.58	1.26	0.14	1.07	0.89254697	0.471
614	21.46	50.46	P51635	Alcohol dehydrogenase [NADP(+)]	16	0.62	0.97	0.61	0.85	0.88	0.92018765	0.0363
615	21.46	49.75	A0A0G2K3Z9	Uncharacterized protein	16	0.75	1.14	1.64	1.01	1.25	1.11651296	0.157
616	21.39	44.09	O35180	Endophilin-A3	12	0.95	1.26	0.57	0.74	0.75	0.97718255	0.328
617	21.37	21.30	O55166	Vacuolar protein sorting-associated protein 52 homolog	12	0.67	0.58	0.29	0.49	0.58	0.95382731	0.0623
618	21.31	22.32	Q9ES21	Phosphatidylinositide phosphatase SAC1	15	1.08	0.98	0.90	1.20	1.16	0.95217589	0.0601
619	21.3	18.76	Q4AEF8	Coatomer subunit gamma-1	12	0.70	0.72	0.95	0.70	0.72	0.97745352	0.278

620	21.24	11.84	Q9JLU4	SH3 and multiple ankyrin repeat domains protein 3	14	0.94	0.95	0.94	0.94	0.98	1.03993979	0.293
621	21.24	19.61	B5DFC3	SEC23A (S. cerevisiae) (Predicted)	14	0.99	1.03	1.01	0.98	1.02	0.94514003	0.18
622	21.24	25.16	P49803	Regulator of G-protein signaling 7	10	0.65	1.11	0.65	1.16	0.93	0.97731802	0.534
623	21.22	12.73	D3ZV52	Intersectin 1	17	1.03	1.04	0.98	0.98	1.01	1.02491197	0.242
624	21.14	15.51	E9PT53	Wolfram syndrome 1 homolog (Human)	12	1.80	0.54	0.90	1.32	1.71	0.99906469	0.973
625	21.13	39.91	F1LPV8	Succinate--CoA ligase [GDP-forming] subunit beta, mitochondrial	12	0.72	0.95	1.11	0.87	0.90	1.04564998	0.391
626	21.12	20.61	F1LPB9	Rabphilin-3A	11	1.74	0.49	1.31	1.51	1.27	1.06009973	0.235
627	21.09	47.45	G3V6H5	Mitochondrial 2-oxoglutarate/malate carrier protein	15	1.16	0.97	1.07	1.16	0.96	0.95674078	0.0527
628	21.09	11.06	D4A144	Adhesion G protein-coupled receptor L1	13	0.99	0.94	0.99	0.95	1.00	0.93044966	0.00375
629	21.08	14.78	D3ZTF0	Similar to Gene model 996	12	0.93	0.98	0.95	0.97	0.94	0.93174043	0.174
630	21.07	51.63	Q6P7Q4	Lactoylglutathione lyase	13	0.96	1.33	1.47	0.88	1.67	0.99075483	0.873
631	20.99	12.29	B5DFC8	Eukaryotic translation initiation factor 3 subunit C	11	1.04	1.08	1.19	1.04	1.10	1.03368743	0.0789
632	20.99	14.90	F1LRN8	Neural precursor cell-expressed, developmentally down-regulated 4-like, E3 ubiquitin protein ligase	14	1.07	0.99	1.02	1.02	1.05	1.03032537	0.145
633	20.96	26.11	B5DFE0	Membrane palmitoylated protein 6	15	1.16	1.64	0.90	0.86	1.04	0.96513322	0.218
634	20.93	42.61	G3V728	4-nitrophenylphosphatase domain and non-neuronal SNAP25-like protein homolog 1 (C. elegans), isoform CRA_b	16	1.82	0.95	1.25	1.57	1.43	0.9568071	0.0497
635	20.93	24.66	P05370	Glucose-6-phosphate 1-dehydrogenase	12	0.81	1.17	1.66	1.41	0.99	1.1671581	0.0832
636	20.9	28.05	F1M471	EPM2A-interacting protein	14	0.79	0.77	0.76	0.79	0.77	0.97225052	0.139
637	20.87	13.75	A0A0G2K261	Isoleucyl-tRNA synthetase 2, mitochondrial	13	0.86	1.12	1.03	1.33	1.12	1.00101945	0.964
638	20.85	17.99	G3V6I1	Lethal(2) giant larvae protein homolog 1	15	0.95	1.04	1.25	1.03	1.19	0.99630544	0.896
639	20.83	19.97	O54924	Exocyst complex component 8	14	1.13	1.33	0.98	1.25	1.18	0.97982771	0.348
640	20.81	28.25	D3ZJH9	Malic enzyme	13	0.90	0.92	0.94	0.93	0.94	1.00849225	0.539
641	20.81	24.32	D4A404	Pleckstrin and Sec7 domain-containing 3	11	1.12	1.47	1.07	1.03	1.06	0.96379619	0.417
642	20.81	53.94	P11348	Dihydropteridine reductase	19	0.82	0.79	1.15	1.42	1.50	1.07549439	0.0961
643	20.8	20.00	G3V881	Leucine rich repeat neuronal 6A, isoform CRA_a	11	0.95	0.65	0.93	0.83	0.77	0.91763988	0.0178
644	20.79	9.48	F1M378	Protein unc-13 homolog A	13	1.46	1.19	0.84	1.15	1.07	0.97589646	0.394
645	20.74	13.11	A0A0G2JX93	Signal transducer and activator of transcription	11	0.58	1.57	1.05	0.72	0.48	1.00037784	0.979
646	20.69	41.42	P10888	Cytochrome c oxidase subunit 4 isoform 1, mitochondrial	22	0.85	1.36	1.11	1.33	0.73	0.91446509	0.155
647	20.67	25.55	F1MAA3	Serine/threonine-protein phosphatase 2A 56 kDa regulatory subunit delta isoform-like	12	0.72	1.45	1.19	1.13	1.19	0.99701699	0.918
648	20.6	21.21	Q4V7C6	GMP synthase [glutamine-hydrolyzing]	12	1.06	1.05	1.05	1.04	1.05	1.04840781	0.135
649	20.59	19.98	D3ZBU7	Similar to putative protein (5S487)	13	1.56	0.81	1.39	1.75	2.09	1.0324701	0.235
650	20.56	18.00	G3V6V1	Aminopeptidase B	10	1.00	0.98	0.96	1.01	0.98	1.02633378	0.312
651	20.51	25.37	Q9WUD9	Proto-oncogene tyrosine-protein kinase Src	12	0.79	0.62	0.59	0.81	0.75	0.99212926	0.83
652	20.44	30.96	Q9JHL4	Drebrin-like protein	14	1.00	1.01	1.01	0.99	1.01	0.93516964	0.0487
653	20.41	16.71	G3V9J7	Rab GTPase-binding effector protein 1	13	1.31	1.39	0.99	0.89	0.89	1.04340554	0.354
654	20.39	28.04	F1M0Z3	Copine 4	15	0.80	0.44	0.42	0.83	1.43	0.94422331	0.217
655	20.37	25.78	Q68FQ9	LanC lantibiotic synthetase component C-like 2 (Bacterial)	12	1.27	1.17	1.14	1.60	1.42	0.97204836	0.328
656	20.35	30.69	Q5M9H4	Sodium/potassium-transporting ATPase subunit beta	17	1.25	0.37	1.69	1.07	1.33	1.07147635	0.286
657	20.31	13.34	Q80ZA5	Sodium-driven chloride bicarbonate exchanger	13	0.67	0.97	0.79	0.74	0.99	0.98910803	0.713
658	20.31	39.47	Q8CFD0	Sideroflexin-5	11	0.99	1.21	1.51	1.49	1.46	1.05672501	0.237
659	20.28	26.01	G3V7I5	Aldehyde dehydrogenase X, mitochondrial	15	1.19	1.45	1.04	1.39	1.45	0.97157684	0.384

660	20.27	24.02	P85968	6-phosphogluconate dehydrogenase, decarboxylating	12	1.17	1.34	1.36	0.93	1.60	1.00213023	0.899
661	20.27	31.96	A0A0G2K1U9	Immunity-related GTPase Q	12	1.24	1.02	0.94	1.10	0.92	0.95362899	0.153
662	20.24	34.03	P29266	3-hydroxyisobutyrate dehydrogenase,	13	0.65	1.01	1.08	0.94	1.21	1.02619151	0.594
663	20.19	30.58	Q5M9H7	DnaJ (Hsp40) homolog, subfamily A, member 2	11	0.86	0.48	1.06	0.97	1.13	1.04362253	0.00674
664	20.18	20.75	F1LPZ8	Neuroligin 3, isoform	12	0.84	0.71	0.78	0.78	0.67	0.99316133	0.849
665	20.18	40.99	P20760	Ig gamma-2A chain C	12	0.68	0.63	0.60	0.35	0.77	0.83856818	0.0284
666	20.16	13.58	D4A781	Importin 5	12	0.99	0.98	1.07	1.06	1.00	1.0243438	0.235
667	20.14	29.53	P15650	Long-chain specific acyl-CoA dehydrogenase,	14	0.79	1.11	1.16	1.12	1.08	0.96821544	0.0832
668	20.14	30.04	D4AEP0	Adenylosuccinate synthetase isozyme 2	10	0.86	0.54	0.77	0.95	0.98	1.13602927	0.104
669	20.11	20.75	P54319	Phospholipase A-2-activating protein	10	0.97	1.03	1.07	1.03	0.99	1.03146867	0.0625
670	20.06	51.19	P54921	Alpha-soluble NSF attachment protein	21	0.94	0.42	0.61	0.56	0.95	0.93971814	0.299
671	20.06	47.19	A0A0G2K459	Mitochondrial carrier 2	14	0.89	0.22	0.77	0.74	0.74	0.99254196	0.917
672	20.05	31.94	Q99ML5	Prenylcysteine oxidase	12	1.39	1.32	1.03	1.25	1.12	0.93516964	0.0123
673	20.03	62.54	A0A0G2KAL9	RCG23940, isoform CRA_g	13	1.02	1.10	1.01	0.99	1.09	1.00185936	0.941
674	20.02	18.47	D3ZDT1	Erythrocyte membrane protein band 4.1-like 2	14	1.17	1.25	1.58	1.33	1.38	1.0554074	0.26
675	19.99	49.31	P32851	Syntaxin-1A	20	1.43	1.67	0.87	1.27	1.43	0.91955005	0.0645
676	19.99	39.55	P03994	Hyaluronan and proteoglycan link protein 1	14	0.87	0.28	1.89	1.26	1.75	1.17772279	0.0429
677	19.98	15.82	A0A0G2JSP3	Cullin-3	12	0.99	0.95	1.00	1.01	0.97	1.00054011	0.968
678	19.95	54.46	A0A0G2JSS8	Peroxiredoxin 5, isoform CRA_c	13	1.11	0.64	1.15	0.42	1.53	0.99261076	0.876
679	19.94	32.62	Q32Q06	AP-1 complex subunit mu-1	12	0.66	0.89	0.63	0.75	0.75	0.92916067	0.0771
680	19.94	37.88	D3ZG43	NADH dehydrogenase (Ubiquinone) Fe-S protein 3 (Predicted), isoform CRA_c	13	0.67	1.18	0.85	0.74	0.60	0.85559503	0.0254
681	19.9	9.70	Q9QX74	SH3 and multiple ankyrin repeat domains protein 2	13	1.18	1.17	1.04	0.79	1.11	1.0324701	0.443
682	19.88	23.14	Q9QXU8	Cytoplasmic dynein 1 light intermediate chain 1	11	0.89	1.28	1.19	1.00	1.21	0.99151053	0.598
683	19.88	16.56	D3ZAN3	Alpha glucosidase 2 alpha neutral subunit (Predicted)	12	0.99	0.80	1.17	1.13	0.91	0.98459342	0.576
684	19.85	15.59	A0A0G2K490	TRAF2 and NCK-interacting kinase	22	1.15	1.24	1.20	1.04	0.95	0.94069569	0.141
685	19.85	17.82	G3V7S0	Folate hydrolase	11	0.96	0.99	1.03	0.98	1.00	0.98999971	0.822
686	19.85	24.23	G3V9B3	Myelin-associated glycoprotein	19	1.28	0.39	0.95	1.38	1.37	1.02654723	0.751
687	19.81	7.66	A0A0G2JX74	Serine/threonine-protein kinase mT	13	0.78	0.79	0.72	0.88	0.86	1.08147476	0.105
688	19.8	45.10	Q68FU3	Electron transfer flavoprotein subunit beta	11	0.46	0.73	0.85	0.64	1.02	1.01473288	0.724
689	19.75	58.47	A0A0G2JTW1	RCG36946	14	1.13	1.13	1.04	1.12	1.00	0.99233559	0.822
690	19.75	50.54	F1M978	Inositol monophosphatase 1	16	0.92	1.11	1.02	1.04	1.08	1.01762076	0.164
691	19.7	63.59	Q5EB77	Ras-related protein Rab-18	11	0.86	1.00	1.66	1.09	1.51	0.96774577	0.288
692	19.68	12.72	E9PTX9	Solute carrier family 12 member 2	11	0.55	0.63	0.61	0.61	0.73	1.033186	0.135
693	19.65	27.68	F1LVR4	Calcium/calmodulin-dependent protein kinase ID	11	1.20	1.22	1.01	1.50	1.89	0.79940583	0.35
694	19.62	18.38	B2DD29	Serine/threonine-protein kinase BRSK1	11	1.18	0.94	0.74	0.78	1.08	1.02704543	0.456
695	19.62	33.58	Q6AYS7	Aminoacylase-1A	12	1.54	1.36	0.88	1.18	1.16	0.89937831	0.0854
696	19.54	37.53	Q6AY30	Saccharopine dehydrogenase-like	13	0.93	0.55	0.86	0.90	0.98	1.03490619	0.564
697	19.54	9.03	Q00657	Chondroitin sulfate proteoglycan 4	19	1.10	1.46	0.76	0.76	1.10	0.96132756	0.5
698	19.52	19.86	Q07009	Calpain-2 catalytic subunit	10	1.19	0.64	1.19	0.90	1.16	1.00290156	0.92
699	19.52	29.64	B1H2A2	Fibronectin type III and SPRY domain-containing 1	10	1.32	1.28	1.19	0.69	0.61	1.03741994	0.105
700	19.37	40.46	P70566	Tropomodulin-2	15	1.34	1.16	0.99	0.38	1.07	0.92980494	0.0918
701	19.37	43.51	Q4FZY0	EF-hand domain-containing protein D2	12	1.39	1.12	1.50	1.01	1.49	0.9976115	0.961
702	19.35	15.02	Q5XIM7	Lysine--tRNA ligase	9	0.98	0.98	0.99	0.95	0.97	0.95899819	0.166
703	19.32	24.82	A0A0H2UHG0	Tyrosine--tRNA ligase	12	0.95	1.01	0.96	0.98	0.98	0.95753691	0.105
704	19.32	15.96	G3V758	Contactin 2	11	0.95	0.91	0.78	1.08	1.08	1.01402977	0.421
705	19.32	26.91	R9PXU4	Thioredoxin reductase 1, cytoplasmic	12	0.97	0.69	1.21	0.57	0.65	0.93303299	0.0889
706	19.31	33.97	Q63570	26S protease regulatory subunit 6B	12	1.04	1.22	1.14	0.78	1.20	1.10496449	0.12
707	19.31	25.92	P08050	Gap junction alpha-1	18	1.08	1.41	2.49	1.77	1.39	1.11033883	0.139
708	19.23	34.30	A2VD09	Abi1 protein	14	0.98	0.95	1.02	1.00	1.05	0.9935538	0.731
709	19.23	13.02	A0A0H2UHQ2	Microtubule-associated protein 1S	11	0.94	0.96	0.99	0.96	0.96	0.99027423	0.757

710	19.22	35.53	Q3S4A4	ADP-ribosylation factor GTPase activating protein 1 heart isoform	12	1.04	1.09	1.03	1.03	1.06	0.92980494	0.0566
711	19.2	38.01	P16446	Phosphatidylinositol transfer protein alpha	11	1.34	1.49	1.34	1.15	1.20	0.981255	0.441
712	19.2	32.92	F1LMM8	[Pyruvate dehydrogenase (acetyl-transferring)] kinase isozyme 2, mitochondrial	11	0.99	1.04	0.94	0.99	0.95	1.00891176	0.67
713	19.14	11.39	D4A022	GTPase-activating protein and VPS9 domains 1	15	1.03	0.93	0.95	0.96	0.97	0.94756709	0.231
714	19.12	15.18	Q5U329	Anion exchange protein	10	0.82	0.98	0.72	1.79	2.51	1.05350707	0.134
715	19.1	72.56	B0BMW0	RAB14, member RAS oncogene family	20	0.85	0.86	0.95	0.88	0.94	0.96580243	0.392
716	19.1	16.13	D4A9D8		10	1.01	0.96	0.98	1.02	1.03	0.99678896	0.805
717	19.09	11.04	Q02356	AMP deaminase 2	10	0.81	0.53	0.75	0.99	1.22	1.00323529	0.912
718	19.09	32.84	B2RYG6	Ubiquitin thioesterase	10	0.98	1.02	0.99	1.01	1.02	0.95826727	0.0638
719	19.05	17.14	Q62634	Vesicular glutamate transporter 1	11	0.99	1.49	0.97	1.39	1.25	0.95409181	0.492
720	19.02	17.14	Q5U2P5	C2CD2-like	11	1.63	1.24	1.42	1.21	1.33	0.98602764	0.416
721	18.98	15.45	Q9WU70	Syntaxin-binding protein 5	14	0.97	0.94	0.98	1.08	1.00	1.02193257	0.369
722	18.9	63.01	P11240	Cytochrome c oxidase subunit 5A, mitochondrial	13	0.79	1.60	1.14	0.56	1.00	0.86753869	0.1
723	18.86	32.62	Q68FW9	C	12	0.98	1.01	1.02	0.95	0.96	1.01395948	0.667
724	18.86	18.58	F1M6V8	Striatin 4	13	0.96	1.01	0.98	0.99	0.97	0.9520439	0.143
725	18.81	37.45	P62909	40S ribosomal protein S3	10	0.92	1.33	1.25	1.34	1.17	1.00940141	0.705
726	18.8	9.93	F1LZX9	Integrin subunit alpha V	9	1.04	0.62	1.18	1.05	0.99	0.97711482	0.14
727	18.79	24.94	P51583	Multifunctional protein ADE2	11	0.55	1.00	1.32	0.97	1.15	1.06984367	0.068
728	18.79	25.49	G3V8T5	RuvB-like helicase	11	1.00	1.21	1.02	0.91	0.99	0.92210312	0.00161
729	18.77	32.89	O88768	ArfGAP with dual PH domains 1	10	1.36	1.45	1.07	1.75	1.53	0.90626194	0.119
730	18.76	36.62	P19627	Guanine nucleotide-binding protein G(z) subunit alpha	13	0.86	0.44	0.65	0.72	0.63	0.92146419	0.0121
731	18.74	46.59	D4A249	Metallo-beta-lactamase domain-containing 2	10	1.33	0.73	1.00	0.98	0.77	0.90689033	0.0809
732	18.73	10.78	P31421	Metabotropic glutamate receptor 2	11	1.36	0.61	1.54	1.69	0.82	0.95224189	0.122
733	18.69	13.58	M0RB22	Protein tyrosine phosphatase, receptor type,	21	1.21	0.90	1.29	0.81	1.10	1.02172008	0.616
734	18.69	36.31	O08618	Phosphoribosyl pyrophosphate synthase-associated protein 2	11	0.58	0.78	1.02	0.89	0.95	0.9977913	0.941
735	18.68	46.63	Q641Z6	EH domain-containing protein 1	24	1.15	1.14	1.04	0.70	1.18	0.98897092	0.638
736	18.68	8.85	E9PSK7	Mitogen-activated protein kinase 8-interacting protein	10	0.99	1.02	1.01	0.97	0.99	1.15268635	0.0743
737	18.61	21.53	M0R7U1	Adenylate kinase 5	11	1.03	1.27	0.95	1.06	0.61	0.95515052	0.0486
738	18.61	34.40	Q5RJN0	NADH dehydrogenase (Ubiquinone) Fe-S protein 7	15	0.90	1.39	1.09	1.32	1.14	0.9695586	0.406
739	18.56	15.59	F1LP01	Diacylglycerol kinase	13	0.95	0.68	0.59	1.02	1.15	0.99547708	0.901
740	18.55	27.62	P19139	Casein kinase II subunit	10	1.12	1.25	1.19	1.25	1.13	0.9208257	0.169
741	18.55	18.48	D3ZWA8	Adaptor protein, phosphotyrosine-interacting with PH domain and leucine zipper 1	10	0.89	0.93	1.04	0.92	0.80	1.01094184	0.462
742	18.55	59.03	P22062	Protein-L-isoaspartate(D-aspartate)	11	0.97	0.83	1.01	0.88	1.16	0.96888678	0.552
743	18.53	21.58	A0A0G2K7R3	Neural cell adhesion molecule 2	14	1.24	0.95	0.94	1.01	0.60	1.36604026	0.032
744	18.53	23.74	P84850	D-2-hydroxyglutarate dehydrogenase,	9	1.01	1.18	0.65	1.18	1.28	1.01952701	0.581
745	18.49	14.43	D3ZBS2	Inter-alpha-trypsin inhibitor heavy chain H3	11	0.60	0.59	0.61	0.55	0.50	0.99226681	0.594
746	18.47	19.73	Q99N27	Sorting nexin-1	10	1.04	1.79	1.32	1.33	1.58	1.02462784	0.642
747	18.46	10.39	F1M853	Ribosome-binding protein 1	15	0.59	0.85	1.15	0.99	1.01	1.09126388	0.176
748	18.43	35.90	D3ZAP9	Glycerol-3-phosphate dehydrogenase [NAD(+)]	16	1.31	1.39	0.95	1.09	1.01	0.95369509	0.127
749	18.4	18.93	Q80Z30	Protein phosphatase 1E	11	0.81	0.08	1.21	1.02	1.26	1.05863114	0.261
750	18.4	35.71	A0A0G2JSH4	Glycogen synthase kinase 3 beta, isoform CRA_b	10	1.02	0.95	0.99	0.98	0.99	0.98924516	0.635
751	18.38	66.67	P20171	GTPase HRas	15	0.95	0.95	1.01	0.95	0.90	0.99392576	0.865
752	18.38	58.47	A0A0G2K0W9	Proteasome subunit alpha type	13	1.09	1.43	1.32	1.36	1.41	1.04789924	0.304
753	18.35	30.65	A0A0G2K0V3	3'(2'),5'-bisphosphate nucleotidase 1	10	0.63	0.79	0.93	0.97	0.98	0.99164799	0.759
754	18.33	8.40	D4A5I9	Myosin VI	9	0.97	0.95	1.31	1.09	1.18	0.99740407	0.872
755	18.32	56.42	O35509	Ras-related protein Rab-11B	12	1.03	1.74	1.17	0.92	1.00	0.94481253	0.098
756	18.31	42.01	Q6AY48	Poly(RC) binding protein 3	15	1.43	0.77	0.95	0.80	0.94	1.03648555	0.419
757	18.3	18.55	Q6AYD5	G1 to S phase transition 1	9	0.92	0.97	0.95	1.02	1.01	0.99247317	0.84

758	18.28	46.88	P12369	cAMP-dependent protein kinase type II-beta regulatory subunit	19	0.85	0.97	0.99	0.36	0.63	0.94173953	0.128
759	18.28	41.69	P38983	40S ribosomal protein SA	14	0.99	0.64	1.36	0.53	0.93	0.95541538	0.016
760	18.27	27.68	P05696	Protein kinase C alpha type	19	0.83	0.86	0.77	1.33	1.61	1.06813944	0.407
761	18.26	43.25	F1LPS8	Transcriptional activator protein Pur-alpha	13	0.82	0.92	1.25	0.76	1.16	0.94369986	0.223
762	18.25	46.33	P27605	Hypoxanthine-guanine phosphoribosyltransferase	13	0.99	0.86	0.79	0.70	1.14	0.94441967	0.0125
763	18.16	46.15	P62982	Ubiquitin-40S ribosomal protein S27a	20	1.10	1.38	1.71	1.66	1.42	1.09050773	0.0251
764	18.15	22.46	D4A4M0	Shisa family member 6	14	1.04	1.06	0.65	1.01	1.11	1.02484093	0.937
765	18.11	7.03	F1MA98	Nucleoprotein TPR	13	0.81	1.31	1.28	0.64	0.95	1.00539331	0.87
766	18.09	28.10	Q62733	Lamina-associated polypeptide 2, isoform beta	10	1.07	1.06	1.03	1.01	1.04	1.11342162	0.0416
767	18.06	57.27	P63012	Ras-related protein Rab-3A	27	0.79	0.31	0.84	0.86	0.79	1.0455775	0.683
768	18.04	65.18	P61983	14-3-3 protein gamma	32	1.01	1.02	1.04	0.97	1.05	0.96888678	0.539
769	18.03	8.53	O35889	Afadin	12	1.42	1.41	1.01	1.18	1.27	1.03548022	0.0991
770	18.02	10.23	D4A6L0	Probable G-protein coupled receptor 158	9	0.54	0.65	1.05	0.90	1.21	1.01945635	0.508
771	18.01	40.97	D4A6H8	Catenin alpha 2	17	0.90	1.06	0.86	1.26	0.94	0.97177889	0.523
772	18	53.41	D4A0T0	NADH:ubiquinone oxidoreductase subunit B10	10	0.69	0.41	0.97	0.69	0.69	0.95773604	0.541
773	18	25.39	F1M7V6	Cell adhesion molecule 4	12	1.08	0.77	1.38	1.47	1.33	1.00057756	0.993
774	17.94	30.94	F1M013	60S ribosomal protein L7a	10	0.94	1.16	1.28	1.50	1.32	1.08147476	0.00735
775	17.93	16.67	G3V9D1	Phosphoinositide phospholipase C	10	1.28	1.34	1.54	1.54	1.54	1.05263116	0.0398
776	17.89	42.11	P21708	Mitogen-activated protein kinase 3	18	1.10	1.41	0.94	1.04	0.79	0.93867654	0.0807
777	17.85	30.18	Q62813	Limbic system-associated membrane protein	9	1.00	0.97	0.81	0.91	0.92	0.92338231	0.00026
778	17.84	37.30	Q5U362	Annexin	10	1.50	0.51	1.74	1.04	1.16	1.06888008	0.35
779	17.84	33.78	O35987	NSFL1 cofactor p47	10	1.14	1.11	1.12	0.47	0.84	0.99116696	0.682
780	17.83	22.58	Q66H61	Glutaminyl-tRNA	14	0.97	0.99	0.95	1.00	0.91	0.97353179	0.597
781	17.83	16.82	Q6P747	Heterochromatin protein 1-binding protein 3	10	1.05	0.97	1.77	1.41	1.79	1.07848043	0.0361
782	17.83	71.13	B0BNA5	Coactosin-like protein	11	1.03	1.22	2.42	1.89	3.08	1.31494276	0.0027
783	17.81	8.15	D3Z9N6	Versican core protein	13	1.08	0.27	2.49	1.79	2.27	1.01924438	0.673
784	17.79	56.06	Q5EGY4	Synaptobrevin homolog YKT6	12	0.80	1.03	1.07	1.02	0.59	0.98561765	0.443
785	17.79	52.26	P56571	ES1 protein homolog, mitochondrial	13	0.77	1.22	1.34	1.31	1.25	0.96895395	0.168
786	17.71	49.52	P62243	40S ribosomal protein S8	11	1.28	0.63	1.63	1.36	1.53	0.99013696	0.76
787	17.69	48.92	Q66HA6	ADP-ribosylation factor-like protein 8B	13	0.94	0.94	1.04	1.05	1.21	1.02647607	0.174
788	17.66	15.11	F1M4I4	Uncharacterized protein	10	1.49	1.56	1.04	1.11	0.87	0.99396021	0.881
789	17.65	46.67	Q6MG60	N(G),N(G)-dimethylarginine	11	1.21	1.33	1.46	1.28	1.74	0.99185422	0.792
790	17.65	17.03	O35828	Coronin-7	10	1.53	1.43	1.54	1.05	1.74	0.97616708	0.404
791	17.65	22.83	P50503	Hsc70-interacting protein	9	1.16	1.17	1.72	1.06	1.37	1.03440417	0.0841
792	17.64	17.77	Q8K4Y5	Leucine-rich glioma-inactivated protein 1	10	0.93	0.79	0.70	0.99	1.21	0.95959663	0.518
793	17.61	27.65	P27952	40S ribosomal protein S2	8	1.13	1.04	1.71	1.38	1.47	1.0396515	0.271
794	17.59	44.83	P07171	Calbindin	13	1.45	1.77	0.66	0.48	0.61	0.85618829	0.0247
795	17.59	41.46	Q4V7D2	Protein rogdi homolog	10	0.65	0.53	0.58	0.76	0.84	0.96019545	0.336
796	17.56	21.71	A0A0G2K014	Lymphocyte cytosolic protein 1	13	0.79	1.27	1.79	0.88	0.77	0.96600329	0.486
797	17.54	70.20	A0A0G2JSH9	Peroxiredoxin-2	16	0.87	1.34	1.71	0.72	1.50	1.06888008	0.364
798	17.49	25.05	F1LML0	Asparaginyl-tRNA	14	0.85	0.72	0.72	0.52	0.67	0.96674011	0.00131
799	17.48	56.59	P12075	Cytochrome c oxidase subunit 5B, mitochondrial	12	1.28	1.51	1.27	0.77	1.57	0.87903956	0.0629
800	17.44	19.97	D3ZLC1	Lamin B2	10	1.27	1.01	0.77	0.56	0.84	0.96299486	0.226
801	17.39	25.10	A0A0G2JZH8	Dihydrolipoamide acetyltransferase component of pyruvate dehydrogenase complex	16	0.87	1.61	1.14	0.95	1.13	1.01066159	0.816
802	17.38	16.34	A0A0H2UHH	ATP-binding cassette sub-family B member 8, mitochondrial	10	0.97	1.00	0.99	0.96	0.98	1.03397407	0.453
803	17.36	63.85	P62630	Elongation factor 1-alpha 1	37	0.89	0.35	2.40	1.77	2.23	1.08372597	0.0562
804	17.36	16.97	A0A0G2JVB0	Solute carrier family 2, facilitated glucose transporter member 3-like	13	1.17	1.69	1.26	1.42	1.16	0.98520783	0.632
805	17.34	19.84	Q5U3Z7	Serine hydroxymethyltransferase	8	0.77	0.93	0.98	1.22	1.18	0.98350207	0.643
806	17.34	24.73	Q6P7Q2	Glypican-1	9	1.16	0.67	0.98	1.29	1.47	0.99533908	0.952
807	17.32	19.02	F1LM05	Serine protease inhibitor	9	0.74	1.05	1.02	0.86	1.16	1.01122217	0.744
808	17.3	13.97	Q8VIJ5	Protein	12	0.72	0.70	1.21	0.92	1.21	1.01073164	0.615
809	17.27	12.39	F1M403	Ubiquitin-conjugating enzyme E2	10	1.16	1.25	1.03	1.04	1.08	1.02790006	0.104
810	17.22	20.74	Q66H80	Coatomer subunit delta	9	0.83	0.65	1.03	0.74	0.68	0.91636865	0.0557

811	17.19	18.66	A0A0G2JY43	Fatty aldehyde dehydrogenase	9	0.83	1.25	0.90	1.10	1.18	0.98500298	0.612
812	17.19	12.56	M0RC17	Cell adhesion molecule L1-like	12	1.07	1.04	1.80	0.89	1.32	1.06348523	0.0739
813	17.18	15.49	P20717	Protein-arginine deiminase type-2	9	1.42	0.91	1.77	1.38	1.47	1.0285415	0.251
814	17.17	10.39	Q07310	Neurexin-3	12	0.95	1.01	0.98	1.02	0.98	0.97029813	0.0487
815	17.17	23.13	Q63413	Spliceosome RNA helicase Ddx39b	13	1.00	0.75	1.04	0.91	0.77	0.98684814	0.3
816	17.16	11.93	F1LNR1	Cytoplasmic linker-associated protein 1	13	0.94	0.99	0.95	0.97	0.95	1.06888008	0.257
817	17.14	15.48	F1LQX4	Rho GTPase-activating protein 44	9	0.90	0.99	0.80	0.97	1.28	1.06459154	0.0265
818	17.13	14.84	M0R5P8	ADAM metallopeptidase domain 22	10	0.79	0.41	0.87	0.51	0.74	1.02349212	0.136
819	17.11	22.26	Q5U2S7	Proteasome (Prosome, macropain) 26S subunit, non-ATPase, 3	12	0.82	1.07	0.81	1.32	0.96	0.98924516	0.331
820	17.11	42.23	P63322	Ras-related protein Ral-A	13	1.21	1.36	1.32	1.66	1.53	1.01087177	0.766
821	17.08	18.08	B2RYI2	Signal recognition particle subunit SRP68	8	0.96	0.80	0.94	1.00	0.84	1.00297803	0.791
822	17.08	8.58	Q7TT49	Serine/threonine-protein kinase MRCK beta	13	1.26	1.14	0.93	1.20	1.15	0.99048017	0.593
823	17.06	27.85	A0A0G2K1S6	Malic enzyme	10	0.77	1.03	0.72	0.80	0.95	0.94861856	0.0256
824	17.06	39.48	P63331	Serine/threonine-protein phosphatase 2A catalytic subunit alpha isoform	16	1.58	0.62	0.95	1.14	1.04	0.95893172	0.00438
825	17.04	45.33	Q6PDV7	60S ribosomal protein L10	13	1.09	0.74	1.64	1.15	1.61	1.00317966	0.935
826	17.02	31.59	Q920Q0	Paralemmin-1	12	1.13	1.36	0.86	0.93	0.83	0.85027416	0.0424
827	17	24.40	G3V8G2	Proteasome (Prosome, macropain) 26S subunit, non-ATPase, 5 (Predicted), isoform CRA_a	13	0.86	1.20	1.21	1.03	1.31	1.0153661	0.867
828	16.99	17.77	D3ZKG1	Methylmalonyl CoA mutase	10	0.65	1.08	0.69	0.70	0.72	1.00758392	0.878
829	16.96	34.30	Q6URK4	Heterogeneous nuclear ribonucleoprotein A3	13	1.03	1.05	1.04	1.00	1.05	1.00258184	0.916
830	16.96	23.29	A0A0G2KAS7	Amino acid transporter	18	0.95	0.47	1.33	1.08	0.98	1.03411742	0.386
831	16.95	22.55	Q9EQS0	Transaldolase	10	1.45	1.26	2.25	1.96	2.23	1.08147476	0.0249
832	16.94	16.70	F7EYB9		8	1.02	0.99	1.02	1.01	1.02	0.99529079	0.855
833	16.89	11.31	F1LMT8	Rab3 GTPase-activating protein non-catalytic subunit	14	0.97	1.03	0.94	1.00	0.99	0.99219803	0.228
834	16.89	44.04	P61589	Transforming protein RhoA	16	1.50	0.39	1.32	1.13	1.38	1.0163519	0.884
835	16.89	31.71	M0RDI5	Mitochondrial calcium uniporter	10	0.93	0.41	0.46	0.97	0.64	0.85797053	0.131
836	16.81	26.45	A0A0G2JSH2	3-hydroxybutyrate dehydrogenase, type 1, isoform CRA_a	12	0.82	1.05	1.18	0.74	1.04	1.02413081	0.361
837	16.79	37.50	P24368	Peptidyl-prolyl cis-trans isomerase B	9	1.03	0.82	1.57	1.57	2.27	1.11651296	0.11
838	16.78	27.44	Q5GFD9	Protein IMPACT	10	0.87	1.20	0.90	0.50	0.69	1.10266916	0.0868
839	16.77	41.89	P07895	Superoxide dismutase [Mn], mitochondrial	12	0.79	1.26	0.76	0.52	0.94	0.87539133	0.0618
840	16.74	60.29	P54313	Guanine nucleotide-binding protein G(I)/G(S)/G(T) subunit beta-2	27	1.36	0.10	1.25	1.15	1.29	0.95468719	0.661
841	16.72	46.61	P04904	Glutathione S-transferase alpha-3	13	1.27	1.02	3.13	1.63	2.42	1.27809936	0.0521
842	16.71	27.82	P09456	cAMP-dependent protein kinase type I-alpha regulatory subunit	10	1.54	1.42	1.60	1.60	1.60	1.02533831	0.287
843	16.71	14.05	Q62824	Exocyst complex component 4	9	0.69	1.14	0.86	1.13	0.96	1.12896441	0.108
844	16.67	27.99	P61203	C	12	1.09	1.16	0.89	1.02	1.11	1.02946872	0.218
845	16.67	15.11	Q6P7A9	Lysosomal alpha-	9	0.79	0.80	0.86	0.63	0.79	0.97319445	0.514
846	16.65	64.09	Q6P9Y4	ADP/ATP translocase 1	39	1.10	0.69	1.33	1.31	1.46	1.03426078	0.382
847	16.62	21.05	Q5M821	Protein phosphatase 1H	9	0.86	1.03	0.85	0.80	0.84	0.98999971	0.505
848	16.61	55.26	P62168	Neuronal calcium sensor 1	9	1.37	1.45	0.80	0.68	1.34	0.90500646	0.00513
849	16.61	14.69	P23514	Coatomer subunit beta	11	0.72	0.88	0.99	0.86	1.03	1.15188764	0.0435
850	16.59	13.56	P97878	Exocyst complex component 5	9	1.21	0.65	1.03	1.28	1.25	1.0049892	0.899
851	16.58	30.94	D4AEH3	Proteasome (Prosome, macropain) 26S subunit, non-ATPase, 7 (Predicted)	9	0.96	0.99	1.00	0.95	0.95	0.94147847	0.292
852	16.58	11.61	G3V8G5	Golgi apparatus protein 1	10	0.99	0.60	1.07	0.85	1.26	0.97657314	0.272
853	16.58	20.77	O08773	Regulator of G-protein signaling 14	8	0.67	0.58	0.78	1.13	1.42	1.054603	0.117
854	16.57	12.30	B2GV87	Receptor-type tyrosine-protein phosphatase epsilon	9	1.06	0.99	1.05	1.04	0.95	0.91130128	0.177
855	16.56	29.60	G3V9T7	ATPase Asna1	9	1.05	1.03	1.00	1.05	1.01	0.99171673	0.768
856	16.55	50.26	P62762	Visinin-like protein 1	14	0.88	0.47	1.11	0.27	0.85	0.94612323	0.479

857	16.54	10.31	F1M820	Sorbin and SH3 domain-containing protein 1	14	0.94	1.00	1.00	0.98	0.95	0.96026201	0.169
858	16.53	25.05	Q9QUR2	Dynactin subunit 4	9	0.95	1.03	1.00	1.03	0.98	0.97292466	0.341
859	16.53	18.82	P86182	Coiled-coil domain-containing protein 22	8	0.96	1.01	1.01	0.94	0.94	0.93303299	0.121
860	16.51	16.25	Q07803	Elongation factor G, mitochondrial	10	0.69	0.86	0.76	0.56	0.79	0.90563398	0.0729
861	16.5	13.83	F1M8A4	PTPRF-interacting protein alpha 2	13	1.28	1.38	0.93	0.87	0.78	1.00583942	0.728
862	16.49	33.03	P37397	Calponin-3	10	1.16	1.32	2.23	1.47	2.19	1.01017133	0.781
863	16.49	20.75	D3ZSY8	TBC1 domain family, member 10b	11	0.98	0.90	0.39	0.74	1.08	0.92916067	0.0128
864	16.49	53.64	P62278	40S ribosomal protein S13	10	1.16	1.29	1.49	1.18	1.24	1.03576736	0.151
865	16.49	54.79	P62250	40S ribosomal protein S16	9	0.88	0.72	1.22	0.95	1.07	1.01101192	0.734
866	16.45	24.95	Q6P7B0	Tryptophan--tRNA ligase, cytoplasmic	10	1.29	1.03	1.07	0.98	1.16	0.99871158	0.895
867	16.45	42.13	A0A0G2K2D6	Tubulin polymerization-promoting protein	14	0.76	1.42	0.66	0.70	1.11	0.92466328	0.0694
868	16.43	32.64	Q9JLZ1	Glutaredoxin-3	10	0.72	1.12	0.82	0.73	0.74	0.98193539	0.64
869	16.42	53.02	M0RC99	Ras-related protein Rab-5A	16	1.06	1.32	0.79	0.96	0.98	1.04405665	0.161
870	16.41	15.41	Q6MG55	Protein ABHD16A	10	0.96	0.90	0.97	0.98	0.93	0.99082351	0.531
871	16.41	39.58	Q6RUV5	Ras-related C3 botulinum toxin substrate 1	11	0.97	1.29	1.47	1.94	1.94	1.00199131	0.98
872	16.34	9.03	A0A0G2QC41	Histone deacetylase 6	10	1.11	0.79	0.46	0.55	0.71	1.1352421	0.135
873	16.32	11.18	P97608	5-oxoprolinase	9	0.92	0.65	1.07	0.74	0.79	0.96079464	0.362
874	16.32	17.50	F1LQ48	Heterogeneous nuclear ribonucleoprotein L	8	1.02	0.79	1.09	0.56	0.79	0.85263489	0.101
875	16.28	21.53	Q5M963	Cytidine monophosphate N-acetylneuraminic acid synthetase	9	1.39	0.59	1.45	1.24	1.66	1.03261324	0.221
876	16.22	18.02	Q6UPE1	Electron transfer flavoprotein-ubiquinone oxidoreductase, mitochondrial	9	0.92	0.95	0.94	1.02	0.96	0.98698496	0.419
877	16.21	20.93	Q5XI21	Target of myb1 homolog (Chicken)	10	0.82	1.41	1.16	1.01	1.03	0.86453723	0.00562
878	16.19	52.43	P62804	Histone H4	13	1.53	1.02	0.44	0.29	0.33	0.51050606	0.00567
879	16.17	38.61	Q8R431	Monoglyceride lipase	10	1.24	0.32	0.66	0.89	0.90	1.02150764	0.768
880	16.16	56.25	A0A0G2JT78	RAB6B, member RAS oncogene family	16	1.03	1.50	1.07	1.25	1.24	0.9534968	0.0506
881	16.15	10.06	Q5YLM1	Sn1-specific diacylglycerol lipase alpha	9	0.98	0.74	0.68	0.90	1.08	0.91763988	0.00444
882	16.15	54.75	B2RYW3	NADH dehydrogenase (Ubiquinone) 1 beta subcomplex, 9	11	0.70	0.88	0.79	0.77	0.77	0.99909932	0.995
883	16.13	35.81	Q6Q7Y5	Guanine nucleotide-binding protein subunit alpha-13	15	0.91	1.18	1.39	1.46	1.38	0.95674078	0.211
884	16.12	18.49	D3ZQ02	WD repeat domain 37	9	1.20	0.79	0.97	1.04	0.97	1.00526091	0.817
885	16.12	21.34	M0RBS9	Sorting nexin family member 27, isoform CRA_a	9	0.98	0.99	0.95	0.97	0.91	0.94671363	0.174
886	16.11	18.61	D4A3E1	Heterogeneous nuclear ribonucleoprotein L-like	9	1.01	0.95	0.96	0.96	0.99	0.96640512	0.354
887	16.11	22.24	P53042	Serine/threonine-protein phosphatase 5	11	1.26	0.59	1.13	0.76	0.82	0.90877812	0.0321
888	16.1	5.96	A0A0G2JVW5	E3 ubiquitin-protein ligase HUWE1	17	0.96	0.96	0.95	0.96	0.95	0.96299486	0.025
889	16.1	24.01	Q5MJ12	F-box/LRR-repeat protein	9	1.02	0.62	0.69	0.73	0.72	0.93698639	0.201
890	16.09	16.67	F1M3P6	Protein SCAI	9	1.00	0.90	0.76	1.09	0.77	1.05059018	0.353
891	16.08	21.19	A0A0H2UHE5	Protein phosphatase 1A	10	0.74	0.72	0.72	0.77	0.93	0.97184625	0.343
892	16.06	10.76	A0A0G2JWA3	Exportin 7	9	1.74	0.95	1.25	1.60	1.33	0.98554934	0.057
893	16.03	12.60	F1LM09	Ubiquitin carboxyl-terminal hydrolase 7	11	0.78	0.79	0.62	0.60	0.90	0.99693407	0.951
894	16.02	37.05	F1M7K7	Voltage-dependent calcium channel gamma subunit	10	1.34	1.27	1.29	1.58	1.79	1.00367348	0.933
895	16	67.08	P31399	ATP synthase subunit d, mitochondrial	12	0.46	1.05	1.15	1.25	1.13	1.04586744	0.321
896	15.99	34.01	P97532	3-mercaptopyruvate sulfurtransferase	8	0.97	0.95	0.94	0.94	0.96	1.04898933	0.455
897	15.96	23.90	P60123	RuvB-like 1	8	0.92	0.98	0.95	0.93	0.93	1.02398885	0.462
898	15.95	17.01	Q62696	Disks large homolog 1	13	1.24	1.43	1.14	1.04	1.14	1.04066087	0.0774
899	15.93	13.90	F1LM66	Elongation factor Tu GTP-binding domain-containing	11	0.79	0.95	0.66	0.70	0.73	0.95740417	0.053
900	15.93	21.09	P14480	Fibrinogen beta chain	10	1.42	1.13	0.62	1.11	0.60	0.91193317	0.14
901	15.91	47.39	A0A0G2K3I9	Dual-specificity phosphatase 3	11	1.19	1.29	1.13	1.13	1.34	0.93744113	0.129
902	15.9	12.00	F1LTW9	EFR3 homolog B	8	0.96	0.94	0.93	0.92	0.91	1.04593993	0.248
903	15.88	21.04	F1LMV9	Coronin	10	0.98	0.70	0.86	1.08	1.08	1.00220664	0.958
904	15.87	35.86	A0A0G2K1I6	Secretory carrier-associated membrane protein	13	1.06	1.18	0.80	1.00	1.02	0.89564567	0.00957
905	15.84	17.69	D3ZQN3	Paraneoplastic Ma antigen family-like 2	9	1.28	1.17	0.58	0.28	1.03	1.00807292	0.631

906	15.82	19.93	A0A0G2JVM(	Delta-1-pyrroline-5-carboxylate dehydrogenase, mitochondrial	9	1.27	1.67	1.08	1.17	1.14	0.98657457	0.675
907	15.82	23.53	Q63083	Nucleobindin-1	10	1.00	0.93	1.07	0.50	1.00	0.93044966	0.00703
908	15.8	21.90	P17475	Alpha-1-antiproteinase	8	2.17	2.86	2.21	1.26	2.40	0.89813237	0.0731
909	15.79	23.72	A0A0G2K872	Cell adhesion molecule 3	12	0.49	0.49	0.76	1.15	1.15	0.99435989	0.93
910	15.73	13.71	G3V8D6	Tripartite motif protein 3, isoform CRA_a	10	1.04	1.01	0.94	0.98	1.01	0.87964908	0.128
911	15.71	32.03	Q5RK08	Glioblastoma amplified sequence	10	0.65	0.64	1.03	0.85	1.19	0.97117285	0.387
912	15.71	18.84	D3ZFX4	Phosphoglucomutase 3	9	1.17	1.07	1.11	1.26	1.02	1.03979563	0.181
913	15.7	16.83	A0A0G2K757	Dolichyl-diphosphooligosaccharide--protein glycosyltransferase subunit 2	9	0.63	0.87	0.80	1.04	0.74	0.9932646	0.648
914	15.67	19.90	D3ZGN2	Copine 5	11	0.89	0.67	0.47	0.26	0.65	1.02555154	0.774
915	15.67	35.27	P13084	Nucleophosmin	11	0.76	1.24	1.38	0.47	0.88	0.92851685	0.406
916	15.64	14.09	D4ABT8	Heterogeneous nuclear ribonucleoprotein U-like 2	11	1.08	0.54	1.07	0.68	0.88	1.16877725	0.268
917	15.62	20.31	A0A0G2K2K1	Phosphodiesterase	9	1.02	0.99	0.72	0.78	0.38	0.88945899	0.0362
918	15.58	51.90	P04906	Glutathione S-transferase P	14	0.99	1.01	1.75	1.91	1.64	1.07474917	0.115
919	15.57	36.42	B2RYW9	Fumarylacetoacetate hydrolase domain-containing protein 2	8	0.80	0.78	0.85	0.64	0.74	0.97806347	0.124
920	15.55	19.60	Q5EBD0	SEC14-like 2 (S. cerevisiae)	10	1.19	1.43	0.77	1.16	1.09	0.9694242	0.0749
921	15.54	40.70	A0A0G2JVL6	NADH dehydrogenase [ubiquinone] 1 alpha subcomplex subunit 8	9	0.84	0.74	1.03	1.16	1.10	0.93355052	0.0319
922	15.51	36.39	B0BN63	Activator of Hsp90 ATPase activity 1	11	1.02	0.97	0.73	0.90	1.16	0.91827616	0.0183
923	15.49	17.78	O88954	MAGUK p55 subfamily member 3	8	0.73	0.90	1.02	1.20	1.05	0.9621942	0.0974
924	15.49	18.83	M0R9Q1	RCG48334, isoform CRA_e	9	0.95	1.27	0.90	1.24	1.03	0.89751005	0.0753
925	15.46	29.53	F1LQS3	60S ribosomal protein L6	9	0.95	1.25	1.37	1.43	1.28	1.08522937	0.00047
926	15.45	10.99	A0A0G2JTL4	KIT proto-oncogene receptor tyrosine kinase	13	0.72	1.47	0.74	1.00	0.98	0.94089132	0.0224
927	15.44	31.82	P12711	Alcohol dehydrogenase class-3	9	1.12	0.55	1.10	1.08	1.27	0.95409181	0.391
928	15.39	7.48	P04775	Sodium channel protein type 2 subunit alpha	11	0.77	0.70	0.72	0.87	0.97	1.02548046	0.135
929	15.34	36.53	B5DFG5	RCG53214, isoform CRA_d	17	1.38	1.25	1.26	1.28	1.41	0.97833469	0.287
930	15.34	14.29	Q5M9G3	Caprin-1	9	0.96	1.20	1.50	0.95	1.34	1.03383074	0.343
931	15.33	30.20	P63095	Guanine nucleotide-binding protein G(s) subunit alpha isoforms short	15	0.87	0.40	0.86	0.85	0.76	0.96299486	0.254
932	15.31	6.13	M3ZCQ2	U5 small nuclear ribonucleoprotein 200 kDa helicase	10	1.06	1.03	1.03	0.95	0.95	1.01283559	0.49
933	15.3	28.72	P63036	DnaJ homolog subfamily A member 1	10	1.28	1.28	1.51	1.06	1.64	1.0051146	0.423
934	15.3	42.74	Q6P9V6	Proteasome subunit alpha type	9	0.87	0.77	1.45	0.78	1.45	0.99716213	0.892
935	15.29	44.93	A0A0G2JSJ2	UMP-CMP kinase	8	0.94	1.09	1.04	0.81	1.43	1.01712712	0.529
936	15.29	41.20	P62828	GTP-binding nuclear protein Ran	10	1.41	0.71	1.38	1.00	1.36	1.01381893	0.773
937	15.29	23.13	P07825	Synaptophysin	24	1.41	0.20	0.91	0.99	1.61	0.96520012	0.709
938	15.25	33.97	O35964	Endophilin-A2	16	1.20	1.06	1.17	0.94	1.37	0.87964908	0.0381
939	15.24	13.97	O88831	Calcium/calmodulin-dependent protein kinase kinase 2	7	1.14	1.04	0.72	0.84	1.06	0.99417381	0.772
940	15.22	22.80	Q68FX3	DEAD (Asp-Glu-Ala-Asp) box polypeptide 19a	9	1.05	0.97	1.01	1.04	1.02	1.02775757	0.319
941	15.22	21.31	G3V792	Cytoplasmic dynein 1 intermediate chain 1	8	1.58	1.77	1.53	0.73	1.25	0.87055056	0.00688
942	15.22	27.83	A0A0G2K657		11	0.95	0.87	0.86	1.42	1.43	0.95409181	0.361
943	15.21	15.79	Q68FT7	Phenylalanyl-tRNA synthetase, beta subunit	8	1.10	1.11	1.06	0.69	0.86	1.25788497	0.05
944	15.21	26.42	P01015	Angiotensinogen	10	0.75	0.67	1.34	0.79	1.28	0.98507126	0.744
945	15.17	35.24	P20651	Serine/threonine-protein phosphatase 2B catalytic subunit beta isoform	28	1.06	0.92	0.67	0.81	0.78	1.01417035	0.872
946	15.16	7.46	P35439	Glutamate receptor ionotropic, NMDA 1	8	0.82	1.17	1.06	1.80	1.49	1.02377594	0.189
947	15.16	21.63	O88377	Phosphatidylinositol 5-phosphate 4-kinase type-2 beta	10	1.03	0.95	0.76	0.82	0.83	0.96413027	0.299
948	15.15	28.77	D3ZLK9	ATP-dependent (S)-NAD(P)H-hydrate dehydratase	9	1.10	1.04	0.98	1.04	1.04	0.97467963	0.343
949	15.14	17.67	P08932	T-kininogen 2	9	3.13	1.38	0.81	0.84	1.02	0.81960461	0.251

950	15.11	8.20	Q6MG49	Large proline-rich protein BAG6	9	1.15	1.11	0.96	1.12	0.89	0.97874165	0.436
951	15.1	12.45	Q63564	Synaptic vesicle glycoprotein 2B	8	1.28	0.38	1.27	1.12	1.24	1.0002981	0.989
952	15.1	34.90	Q63635	Syntaxin-6	8	1.04	1.01	0.99	1.00	1.02	1.01445158	0.715
953	15.07	13.99	P35400	Metabotropic glutamate receptor 7	10	0.89	0.92	1.11	1.09	1.25	0.99579453	0.902
954	15.07	13.53	A0A0G2JSK5	Integrin beta	9	0.97	1.37	1.28	1.58	1.07	1.07848043	0.0218
955	15.05	43.15	P15791	Calcium/calmodulin-dependent protein kinase type II subunit delta	42	0.97	1.11	2.33	1.33	1.69	1.72189838	0.01208
956	15.05	19.76	Q8K1Q0	Glycylpeptide N-tetradecanoyltransferase 1	9	1.47	1.18	0.98	1.57	1.34	0.98057508	0.639
957	15.02	26.62	P36876	Serine/threonine-protein phosphatase 2A 55 kDa regulatory subunit B alpha isoform	8	0.75	1.54	0.95	0.72	1.27	0.92530443	0.187
958	15.01	39.63	P62994	Growth factor receptor-bound protein 2	9	2.36	1.69	2.11	1.04	1.91	0.93991357	0.0238
959	14.93	15.94	Q505I9	Epsin 2	10	1.32	1.04	1.41	0.81	1.21	1.04311629	0.248
960	14.93	25.32	F1LRB8	S-adenosylmethionine synthase	8	0.98	0.91	0.95	0.92	0.96	0.90375273	0.0116
961	14.92	23.14	Q5RJR2	Twinfilin-1	8	1.14	1.07	1.33	1.03	1.47	0.97542307	0.184
962	14.87	14.45	A0A0G2K0B4	E3 ubiquitin-protein ligase NEDD4	12	0.77	1.11	0.98	1.17	1.04	1.01073164	0.764
963	14.87	46.40	Q00715	Histone H2B type 1	27	1.27	1.15	1.08	0.79	0.72	0.61813763	0.00991
964	14.86	31.84	Q63345	Myelin-oligodendrocyte glycoprotein	9	0.94	0.29	1.15	1.67	2.11	1.14155471	0.22
965	14.85	17.06	D4A5J1	Kelch repeat and BTB domain-containing 11	9	1.60	1.16	1.28	1.28	1.09	1.02002181	0.717
966	14.82	9.78	Q6IRL2	Mitofusin 2	9	0.95	1.01	0.97	1.02	1.00	1.01164281	0.619
967	14.81	19.62	O35458	Vesicular inhibitory amino acid transporter	11	0.81	0.84	1.07	0.58	0.95	0.96206082	0.421
968	14.8	8.87	A0A0G2KB46	Rho GTPase-activating protein 35	10	0.96	0.68	0.77	0.71	0.79	0.9885597	0.564
969	14.79	11.79	Q8CF97	Deubiquitinating protein VCIP135	10	0.82	0.90	0.86	1.10	1.15	1.01297601	0.682
970	14.77	16.55	Q03351	NT-3 growth factor receptor	12	0.98	1.02	1.06	1.05	1.02	1.05168307	0.171
971	14.74	35.07	P14659	Heat shock-related 70 kDa protein 2	52	1.32	1.01	1.85	1.25	1.43	1.04928021	0.343
972	14.73	10.00	Q9JJ31	Cullin-5	7	1.02	1.08	1.45	0.70	1.15	1.05285007	0.293
973	14.69	9.53	F1LPM3	Sorbin and SH3 domain-containing protein 2	9	0.64	1.25	0.79	1.63	1.13	0.95257197	0.554
974	14.68	59.03	D3ZE15	NADH dehydrogenase [ubiquinone] 1 alpha subcomplex subunit 13-like	9	0.69	1.32	0.92	0.65	0.81	0.8735729	0.0465
975	14.65	8.84	F1LW74	IQ motif-containing GTPase-activating protein 2	11	1.24	1.11	1.01	1.29	1.29	1.0034787	0.609
976	14.65	10.40	P18395	Cold shock domain-containing protein E1	9	0.88	1.29	1.14	1.10	1.11	1.08748839	0.037
977	14.64	31.88	A0A0G2K7G7	Septin-8	16	1.16	1.43	1.27	1.39	1.45	1.01571806	0.292
978	14.63	30.97	G3V7P8	N-myc downstream regulated gene 4, isoform	9	0.79	0.95	0.97	1.21	0.99	0.96346222	0.517
979	14.63	9.98	F1LS86	Isoleucine-tRNA synthetase (Predicted)	10	0.97	1.05	1.03	1.02	0.97	1.02847021	0.102
980	14.61	46.15	P17220	Proteasome subunit alpha type-2	9	0.76	1.25	1.24	1.20	1.02	0.9623943	0.443
981	14.58	45.74	Q9WUS0	Adenylate kinase 4, mitochondrial	8	1.18	1.41	1.21	1.46	0.95	0.93238649	0.0871
982	14.47	38.13	P62919	60S ribosomal protein L8	8	1.21	1.29	1.94	1.58	1.63	1.05394531	0.183
983	14.47	33.56	Q6AZ33	Biliverdin reductase A	8	1.01	1.07	0.95	0.80	1.17	0.95131828	0.291
984	14.46	11.12	D4AB03	Family with sequence similarity 120A	9	1.05	1.04	1.06	1.05	1.06	1.03447588	0.135
985	14.44	56.49	O08557	N(G),N(G)-dimethylarginine	11	0.44	0.41	1.36	0.36	1.26	0.91383145	0.196
986	14.43	9.03	F1LQL1	Nitric oxide synthase	12	1.20	1.12	0.99	0.81	0.79	0.9952494	0.811
987	14.43	27.25	M0R3N4	Vesicle amine transport 1-	8	0.44	0.31	0.52	0.35	0.72	0.95594532	0.52
988	14.41	20.89	P37285	Kinesin light chain 1	10	0.96	0.98	1.00	1.01	1.01	1.00025719	0.987
989	14.4	19.43	Q9QVC8	Peptidyl-prolyl cis-trans isomerase FKBP4	8	0.95	0.91	0.95	0.93	0.93	0.94500902	0.419
990	14.38	15.15	B2GV15	Dihydrolipoamide acetyltransferase component of pyruvate dehydrogenase complex	8	1.03	1.02	0.98	1.03	1.03	0.99185422	0.695
991	14.37	61.01	P08009	Glutathione S-transferase Yb-3	18	0.95	1.49	0.69	0.89	1.19	0.87539133	0.102
992	14.36	16.08	Q7TSA0	Mitochondrial Rho GTPase	9	0.92	0.89	0.80	0.82	0.70	1.03612639	0.356
993	14.34	10.51	A0A0G2JZF0	SEC24 homolog C, C	10	1.05	0.95	0.97	0.96	0.96	1.00132479	0.96
994	14.32	22.51	G3V6H9	Nucleosome assembly protein 1-like 1	9	0.99	1.56	1.60	0.40	1.25	1.08899702	0.0388

995	14.32	13.41	D3ZKQ4	Putative uncharacterized protein RGD1307615_predicted	8	1.06	1.36	1.20	0.50	1.14	0.95429023	0.226
996	14.3	47.46	P10824	Guanine nucleotide-binding protein G(i) subunit alpha-1	30	1.18	0.70	0.43	1.02	0.63	0.95508431	0.295
997	14.28	55.17	G3V8U9	Proteasome subunit beta	9	0.81	0.76	1.02	0.53	0.49	0.9666731	0.566
998	14.25	25.80	B0BN93	26S proteasome non-ATPase regulatory subunit	8	1.53	1.36	1.46	0.90	1.01	0.9840476	0.635
999	14.25	8.95	D4A1G8	Centrosomal protein 170B	10	0.88	1.03	1.15	0.69	1.18	0.98999971	0.122
1000	14.23	22.55	D4A6C5	Rho GTPase-activating protein 1	11	0.85	1.31	0.87	0.97	0.92	0.95323247	0.145
1001	14.22	15.99	D4AA63	Ubiquilin 2	16	1.25	1.41	1.19	0.36	1.18	1.07177346	0.0266
1002	14.19	56.19	D4A5L9	Uncharacterized protein	11	0.36	1.13	0.63	0.79	0.38	0.78132788	0.172
1003	14.15	14.41	A0A0H2UHD	Nicalin	7	1.06	1.06	0.96	1.00	1.05	0.95959663	0.0285
1004	14.13	10.58	D3ZI07	Kinesin-like protein	7	0.97	0.95	1.01	0.69	0.95	0.99994178	0.999
1005	14.12	27.41	Q63525	Nuclear migration protein nudC	9	1.53	1.36	1.18	0.60	1.29	1.83782577	0.01798
1006	14.12	14.39	A0A0G2K1M	Prolyl endopeptidase	8	0.91	0.83	0.97	0.91	0.93	1.01917373	0.658
1007	14.1	17.91	Q9QZR6	Septin-9	11	0.94	0.97	0.72	0.87	0.78	0.93303299	0.197
1008	14.1	54.90	P26772	10 kDa heat shock protein, mitochondrial	7	1.00	1.53	1.27	1.28	1.28	0.9347808	0.00035
1009	14.09	19.25	B5DF41	Syntaphilin	10	0.83	0.82	0.87	0.51	0.69	1.03849913	0.275
1010	14.09	50.79	Q32PX6	Ras homolog family member G	10	1.02	0.83	1.38	1.67	1.98	1.08147476	0.359
1011	14.07	19.82	Q6AYG3	Protein prune homolog	9	0.91	1.26	0.52	1.08	1.02	1.01804406	0.716
1012	14.06	15.99	P49620	Diacylglycerol kinase	8	0.90	1.26	0.94	0.67	0.63	0.98712179	0.401
1013	14.06	42.33	Q4KM74	Vesicle-trafficking protein SEC22b	10	0.67	0.79	1.05	0.79	0.98	0.93044966	0.0765
1014	14.04	34.54	P29314	40S ribosomal protein S9	10	0.90	1.20	1.24	1.42	1.27	0.99584285	0.896
1015	14.03	15.38	Q499P2	Leukotriene A(4) hydrolase	8	1.00	0.95	0.97	0.97	0.95	0.99504936	0.781
1016	14.03	27.74	Q03114	Cyclin-dependent-like kinase 5	7	0.95	1.01	1.06	1.12	1.05	0.99096087	0.513
1017	14.02	29.55	F7EL36	Acidic leucine-rich nuclear phosphoprotein 32 family member A	11	1.46	0.87	1.80	0.88	1.24	1.00056993	0.995
1018	14.01	20.11	D3ZJX5	RCG54610, isoform CRA_a	8	0.95	0.95	0.99	1.00	0.99	0.96286137	0.248
1019	13.98	13.46	A0A0G2QC21	Rho guanine nucleotide exchange factor 7	9	0.95	0.99	0.99	1.03	1.08	1.0287554	0.171
1020	13.97	16.76	P49088	Asparagine synthetase [glutamine-hydrolyzing]	8	0.98	1.02	0.97	0.99	0.97	0.99578073	0.864
1021	13.95	26.35	P68101	Eukaryotic translation initiation factor 2 subunit 1	9	0.86	1.25	1.32	1.31	1.29	1.08824244	0.126
1022	13.94	68.10	P63045	Vesicle-associated membrane protein 2	22	1.21	1.58	0.89	0.94	0.77	0.83450928	0.0374
1023	13.93	4.19	A0A0G2K9M	WD repeat and FYVE domain-containing 3	12	0.95	0.92	0.95	0.99	1.01	1.01122217	0.0886
1024	13.92	14.55	D4AEI5	Myelin expression factor 2	10	1.69	1.60	1.37	1.29	1.17	0.97914878	0.405
1025	13.92	22.53	Q1WIM2	Cell adhesion molecule 2	9	1.41	0.31	2.23	2.63	2.99	1.13602927	0.1
1026	13.87	24.81	Q9QX69	LanC-like protein 1	9	1.05	1.04	1.07	1.03	1.08	1.04782661	0.436
1027	13.87	26.57	G3V734	2,4-dienoyl CoA reductase 1, mitochondrial, isoform CRA_a	8	1.08	1.87	2.33	1.37	1.91	1.08222465	0.115
1028	13.86	25.93	A0A140TAG	Hsp90 co-chaperone Cdc37	9	1.22	0.98	0.98	0.74	0.99	0.96895395	0.3
1029	13.84	22.09	P97610	Synaptotagmin-12	8	0.41	0.79	0.47	0.49	0.61	0.83335321	0.106
1030	13.81	14.45	Q63259	Receptor-type tyrosine-protein phosphatase-like N	11	0.67	0.67	0.66	0.62	0.51	1.04058874	0.169
1031	13.8	46.10	Q5RJL0	Ermin	8	1.36	0.80	1.64	0.75	1.82	1.0565053	0.388
1032	13.79	25.46	P47728	Calretinin	12	0.95	0.53	0.51	0.22	0.64	0.89564567	0.0272
1033	13.79	21.81	Q9JJ54	Heterogeneous nuclear ribonucleoprotein D0	9	1.02	0.99	1.00	0.98	1.03	1.06688154	0.313
1034	13.78	11.34	B1WBY1	Cul1 protein	7	1.01	0.97	0.98	0.94	1.00	1.03749185	0.0714
1035	13.73	21.37	A1L1M0	Protein kinase, cAMP-dependent, catalytic, alpha	7	0.47	1.11	0.96	0.65	0.99	0.98760086	0.194
1036	13.71	30.67	A0A0G2JSL0	Proteasome subunit beta	7	0.99	0.91	0.96	0.93	1.01	0.93971814	0.371
1037	13.71	32.50	Q5PQZ9	NADH dehydrogenase [ubiquinone] 1 subunit C2	8	0.89	0.90	0.79	0.96	0.68	0.88025901	0.0323
1038	13.7	32.18	P21670	Proteasome subunit alpha type-4	8	0.90	1.38	1.07	1.42	1.54	1.02576482	0.325
1039	13.69	11.19	D3ZU84	Syntaxin-binding protein 5-like	7	0.61	0.38	0.71	0.87	0.96	1.03792342	0.0206
1040	13.67	8.10	A0A0G2JUR	Myosin phosphatase Rho-interacting protein	11	1.13	1.10	0.83	1.11	1.29	0.99694098	0.836
1041	13.67	54.07	P55053	Fatty acid-binding protein, epidermal	10	1.18	0.17	3.19	0.96	2.42	1.20999409	0.0948
1042	13.64	22.71	B5DEH2	Erlin-2	8	0.54	0.54	0.70	0.81	0.87	0.94415786	0.506
1043	13.63	44.64	B2RZ72	Actin-related protein 2/3 complex subunit 4	9	1.00	1.47	1.31	2.11	1.82	1.06451775	0.519
1044	13.62	54.63	P57113	Maleylacetoacetate	8	1.31	0.67	0.91	1.04	0.95	0.98554934	0.534
1045	13.57	11.50	Q62688	Inactive phospholipase C-like protein 1	10	1.02	0.98	0.96	1.03	1.09	1.05029893	0.0126
1046	13.53	17.89	A0A0G2JZH0	Calcium-binding protein 39	7	0.84	1.51	0.52	0.69	0.93	0.99144181	0.565

1047	13.5	8.81	P31424	Metabotropic glutamate receptor 5	10	1.01	1.01	1.02	1.10	1.06	1.04456336	0.23
1048	13.47	7.58	D4A9D6	DEAH (Asp-Glu-Ala-His) box polypeptide 9	10	0.94	0.86	0.75	0.77	0.74	0.98684814	0.247
1049	13.47	15.57	P14925	Peptidyl-glycine alpha-amidating monooxygenase	9	0.67	0.65	0.72	0.40	0.50	0.99686497	0.881
1050	13.47	15.41	D3ZPJ0	Shisa family member 7	7	1.02	0.98	0.94	0.95	0.94	0.93919719	0.231
1051	13.46	36.89	B5DEN5	Eukaryotic translation elongation factor 1 beta 2	8	1.02	1.54	1.66	0.75	1.33	0.9208257	0.446
1052	13.43	19.44	B5DFL0	Snta1 protein	8	0.78	1.04	1.19	1.10	0.94	1.01550687	0.475
1053	13.43	28.90	P18420	Proteasome subunit alpha type-1	8	0.73	1.10	1.06	1.31	1.01	0.9970861	0.934
1054	13.41	21.21	Q5RJK6	Inositol polyphosphate-1-phosphatase	9	1.01	0.95	0.95	0.94	0.94	0.90563398	0.024
1055	13.41	44.00	F1M695	YjeF N-terminal domain-containing 3	9	0.95	0.91	0.99	0.90	0.93	0.98821715	0.554
1056	13.4	75.63	P50408	V-type proton ATPase subunit F	7	1.15	1.27	1.03	0.99	1.33	0.90626194	0.0522
1057	13.39	53.05	P51146	Ras-related protein Rab-4B	12	0.94	0.93	0.86	0.95	0.90	0.99151053	0.766
1058	13.37	45.73	Q5M7A7	CB1 cannabinoid receptor-interacting protein 1	7	1.20	1.13	1.07	1.16	1.10	0.90312651	0.0275
1059	13.35	15.52	Q5I0C3	Methylcrotonoyl-CoA carboxylase subunit alpha, mitochondrial	8	1.04	1.05	1.01	1.02	1.03	1.0159293	0.483
1060	13.34	7.54	D4AD15	Eukaryotic translation initiation factor 4 gamma, 1	9	0.95	1.15	1.11	1.01	1.04	1.05051736	0.0999
1061	13.34	6.86	D3ZF26	Tankyrase 1-binding protein	8	1.84	0.74	1.28	0.90	1.29	1.05782428	0.0186
1062	13.32	8.17	F1LW91	Nuclear mitotic apparatus protein 1	14	0.99	0.95	1.03	1.04	1.04	1.03068251	0.351
1063	13.32	9.27	F1LN59	Eukaryotic translation initiation factor 4, gamma 2	7	1.13	1.12	1.17	0.99	1.27	1.02868409	0.129
1064	13.32	23.74	Q5RJR9	Serine (	7	0.74	0.92	0.99	0.97	1.15	0.95112048	0.353
1065	13.32	17.54	Q4V7D4	Family with sequence similarity 126, member B	8	1.04	1.05	0.90	0.77	0.91	1.15668818	0.116
1066	13.3	22.63	P97612	Fatty-acid amide hydrolase 1	8	0.82	0.83	0.34	1.00	1.24	1.11342162	0.364
1067	13.29	30.67	Q6AYP5	Cell adhesion molecule 1	14	1.22	0.13	0.94	0.74	0.65	1.55078641	0.0116
1068	13.29	13.90	B1WC34	Protein kinase C substrate 80K-H	7	0.80	0.78	1.36	0.48	1.26	0.9989262	0.98
1069	13.26	37.88	G3V6Q6	Guanine nucleotide binding protein, alpha 11	12	0.97	1.01	1.01	0.95	0.95	0.94350365	0.0178
1070	13.26	56.03	A0A0G2JY03	Uncharacterized protein	17	1.16	1.42	0.96	0.93	1.05	0.91256549	0.221
1071	13.25	9.02	Q9JJ50	Hepatocyte growth factor-regulated tyrosine kinase substrate	11	1.08	1.19	1.21	0.88	1.28	0.88025901	0.0274
1072	13.24	29.22	G3V9U2	3-ketoacyl-CoA thiolase, mitochondrial	8	0.79	0.55	0.53	0.62	1.26	0.99511144	0.838
1073	13.24	27.13	A0A0G2K865	Tumor protein D52	7	1.00	1.00	1.05	0.98	1.04	1.00030087	0.996
1074	13.2	20.66	Q5EB81	NADH-cytochrome b5 reductase 1	7	1.03	1.41	1.20	1.19	1.14	1.04782661	0.252
1075	13.2	38.46	D3ZXP3	Histone H2A	11	1.67	1.34	0.50	1.15	0.86	0.47896761	0.01908
1076	13.19	18.73	D3ZB78	PHD finger protein 24	7	1.69	0.91	0.79	1.04	1.09	0.88209137	0.123
1077	13.19	20.86	Q641Y0	Dolichyl-diphosphooligosaccharide--protein glycosyltransferase 48 kDa subunit	7	0.85	0.89	1.19	1.25	0.95	0.99157926	0.305
1078	13.16	13.54	D3ZUM2	Sterile alpha and TIR motif containing 1 (Predicted)	8	0.83	1.01	0.90	0.92	0.82	1.00347175	0.843
1079	13.15	13.62	G3V7I7	Leucine-rich repeat-containing 40	9	0.82	0.48	0.60	0.46	0.55	0.99020559	0.921
1080	13.15	14.60	Q6P6Q5	Amyloid beta A4 protein	9	1.26	0.92	1.92	1.28	1.31	1.01938569	0.771
1081	13.15	28.68	B2GV54	Neutral cholesterol ester hydrolase 1	7	0.63	1.13	1.17	0.82	1.01	1.03132569	0.328
1082	13.14	6.79	Q4G017	Nischarin	9	0.98	1.01	0.97	0.99	0.96	1.05131864	0.221
1083	13.13	23.99	Q02874	Core histone macro-H2A.1	7	0.82	0.65	0.52	0.39	0.25	0.973127	0.223
1084	13.13	26.99	B5DF91	ELAV-like protein 1	7	0.70	0.86	1.28	0.98	1.16	1.00651593	0.792
1085	13.12	48.98	D3ZFY8	Ubiquitin-conjugating enzyme E2 variant 1-like	9	0.91	1.02	1.13	0.96	1.10	0.98527612	0.683
1086	13.11	45.21	A0A0G2K737	Thioredoxin-like protein 1	9	1.00	0.95	1.05	0.46	1.03	0.93581808	0.18
1087	13.1	28.30	P32089	Tricarboxylate transport protein, mitochondrial	7	0.86	0.55	1.26	1.33	1.12	1.01178307	0.456
1088	13.09	49.64	Q63754	Beta-synuclein	18	1.28	1.82	1.38	0.63	1.20	0.77217513	0.244
1089	13.09	16.11	F1LMT5	PEX5-related protein	7	1.15	1.87	0.95	1.43	1.00	0.97366676	0.104
1090	13.08	13.77	P15178	Aspartate--tRNA ligase, cytoplasmic	8	0.96	0.59	0.97	0.85	0.96	0.96526703	0.324
1091	13.06	8.79	D4A0A1	Uncharacterized protein	8	0.81	0.86	0.79	0.90	0.90	0.96714225	0.0165
1092	13.06	10.14	F1M392	LIM and calponin homology domains 1	9	1.01	1.03	1.07	1.07	1.10	1.0382832	0.255
1093	13.04	16.32	Q5XI19	Fermitin family homolog 2 (Drosophila)	10	0.82	1.36	1.17	1.08	1.07	1.01988042	0.412
1094	13.04	14.69	Q07205	Eukaryotic translation initiation factor 5	7	0.90	0.87	0.98	0.79	0.85	1.01755022	0.176

1095	13.04	19.17	Q99068	Alpha-2-macroglobulin receptor-associated protein	6	1.16	1.28	1.06	0.81	1.28	1.02150764	0.567
1096	13.03	16.38	A0A0G2K9C5	Numb-like protein	8	1.02	1.00	0.98	1.02	1.02	0.99013696	0.373
1097	13.03	47.76	P84087	Complexin-2	11	1.28	0.05	1.42	1.01	1.63	0.85027416	0.322
1098	13.03	37.50	G3V7Y3	ATP synthase subunit delta, mitochondrial	12	0.99	1.33	1.29	0.55	0.93	0.82188019	0.0568
1099	13.01	82.14	Q5XIF6	Tubulin alpha-4A chain	261	1.56	0.54	1.53	1.77	2.11	1.00336047	0.947
1100	13	12.84	Q5BKC9	Ephexin-1	7	0.98	0.65	0.37	0.35	0.51	0.94265385	0.011
1101	12.98	13.75	Q641Y5	Ubiquitin-like modifier-activating enzyme ATG7	8	0.97	0.97	0.92	1.05	0.94	1.11265012	0.0453
1102	12.96	42.13	P11730	Calcium/calmodulin-dependent protein kinase type II subunit gamma	39	0.59	1.04	1.89	1.84	2.25	1.66786209	0.01197
1103	12.96	12.56	P19468	Glutamate--cysteine ligase catalytic subunit	7	0.68	0.78	0.98	0.79	1.04	1.07848043	0.00583
1104	12.95	32.86	F1M2E9	60S ribosomal protein L13	9	1.07	1.22	1.37	1.12	1.34	0.97238531	0.324
1105	12.95	21.84	O88321	Antisecretory factor	8	1.47	1.32	1.64	1.39	1.36	1.04434617	0.0113
1106	12.94	15.69	P32232	Cystathionine beta-synthase	8	1.00	1.16	0.59	0.76	0.72	0.96976023	0.261
1107	12.94	7.24	F1M2J2	Ankyrin repeat and sterile alpha motif domain-containing protein 1B	7	0.72	0.79	1.06	1.17	1.25	1.08297505	0.0558
1108	12.94	34.25	Q63584	Transmembrane emp24 domain-containing protein	8	0.72	0.82	1.14	1.06	1.12	0.99757001	0.913
1109	12.91	5.09	Q2IBD4	Cortactin-binding protein 2	9	0.95	0.98	1.03	1.03	1.02	1.06873191	0.162
1110	12.91	36.68	Q6AXX6	Redox-regulatory protein FAM213A	8	0.87	1.27	1.17	1.42	1.31	0.96332867	0.154
1111	12.9	25.93	P09895	60S ribosomal protein L5	10	0.97	1.28	1.63	1.50	1.49	0.96252772	0.0626
1112	12.89	10.78	D4A994	ER membrane protein complex subunit 1	8	0.98	1.00	1.03	1.01	1.06	1.01557726	0.206
1113	12.89	20.13	Q6IRK9	Carboxypeptidase Q	8	0.92	1.01	1.02	1.06	0.98	1.0603202	0.18
1114	12.89	51.74	P13832	Myosin regulatory light chain RLC-A	8	1.72	2.09	1.85	0.65	1.75	0.92530443	0.197
1115	12.88	18.64	B0BND0	Ectonucleotide pyrophosphatase/phosphodiesterase family member 6	6	0.92	0.81	1.00	1.31	1.47	0.97474719	0.529
1116	12.86	18.79	D3ZVS2	L-2-hydroxyglutarate dehydrogenase	8	1.12	1.13	1.12	1.01	1.04	1.10880064	0.0533
1117	12.86	22.84	B1WC26	N-acetylneuraminate synthase	7	0.96	1.06	1.10	1.05	1.09	0.99991683	0.998
1118	12.84	62.71	P80254	D-dopachrome	13	1.36	1.29	0.86	0.12	1.00	0.81168958	0.0651
1119	12.82	10.83	D4A8V2	Coiled-coil domain-containing 177	7	0.96	1.01	0.97	1.04	1.00	0.86994735	0.192
1120	12.82	17.83	Q4KMA2	UV excision repair protein RAD23 homolog B	9	1.27	1.92	1.12	0.60	1.15	1.03619821	0.609
1121	12.81	63.01	P35213	14-3-3 protein beta/alpha	41	1.14	1.46	1.66	1.36	1.37	0.99219803	0.799
1122	12.81	14.46	G3V6T7	Protein disulfide-isomerase A4	12	1.04	1.02	1.00	1.01	0.98	1.00870198	0.815
1123	12.78	53.40	Q6PDU7	ATP synthase subunit g, mitochondrial	11	1.15	1.51	1.17	1.20	1.21	0.94278454	0.0126
1124	12.77	32.18	F7EPH4	Pyrophosphatase (inorganic) 1	9	0.84	1.41	1.33	0.99	1.21	0.99267957	0.705
1125	12.76	11.57	A0A0G2JZG7	Serine--tRNA ligase, cytoplasmic	6	0.95	1.01	1.01	1.03	0.99	0.96259444	0.295
1126	12.75	10.49	B5DEJ1	SLIT-R	9	0.76	0.52	0.64	0.64	0.68	0.99903007	0.969
1127	12.74	9.39	D3ZSA9	Nodal modulator 1	9	1.00	0.99	0.99	1.04	1.03	0.95966315	0.0454
1128	12.74	18.45	B1WBS4	Similar to Vacuolar protein sorting 26 homolog (VPS26 protein homolog), isoform CRA_a	8	0.79	0.99	0.80	0.80	0.86	1.01966836	0.211
1129	12.71	18.63	A0A0G2JTA1	Protein phosphatase 2 regulatory subunit B',	7	0.75	0.46	0.95	1.09	0.70	0.97643777	0.349
1130	12.71	38.49	G3V8D5	6-phosphogluconolactonase	10	0.84	0.82	1.29	0.95	1.08	1.02619151	0.241
1131	12.69	13.20	P31662	Sodium-dependent neutral amino acid transporter SLC6A17	12	1.04	1.02	1.00	1.00	1.01	0.98336574	0.299
1132	12.67	5.98	F1LXQ7	Rho GTPase-activating protein 21	9	0.97	1.12	0.72	1.10	0.97	1.04717314	0.0646
1133	12.66	10.19	D3Z9Z0	Ankyrin 1	17	0.40	0.64	0.50	0.54	0.77	1.02342118	0.0513
1134	12.66	35.53	Q792I0	Protein lin-7 homolog C	8	1.15	0.99	1.02	1.14	1.02	0.97582882	0.104
1135	12.65	26.84	F1LU48	Endoplasmic reticulum-golgi intermediate	8	1.04	1.06	1.03	1.02	0.98	0.99533908	0.848
1136	12.64	17.98	G3V940	Coronin	8	1.14	1.06	1.37	1.34	1.57	1.02754388	0.165
1137	12.61	17.51	Q6AYD3	Proliferation-associated protein 2G4	7	1.38	1.41	1.58	1.32	1.41	0.96908828	0.366
1138	12.6	27.80	P69682	Adaptin ear-binding coat-associated protein 1	7	0.97	1.38	1.02	0.74	1.19	0.93238649	0.108
1139	12.6	36.55	Q9Z270	Vesicle-associated membrane protein-	11	1.04	1.01	1.39	1.29	1.29	0.95151612	0.253
1140	12.59	17.66	H9KVF3	Serine/threonine-protein kinase 24	6	0.83	1.18	0.87	1.14	0.96	1.06112896	0.335

1141	12.59	19.19	P10499	Potassium voltage-gated channel subfamily A member 1	7	1.03	1.04	0.98	1.04	1.15	0.93044966	0.151
1142	12.54	66.31	P68035	Actin, alpha cardiac muscle	132	1.08	2.31	0.69	2.38	0.98	1.09809281	0.00471
1143	12.54	18.75	A0A0G2K5E4	DnaJ heat shock protein family (Hsp40) member A3	8	0.85	1.00	0.64	0.56	0.64	0.97813127	0.519
1144	12.52	29.50	D3ZEI4	Uncharacterized protein	12	1.50	1.09	2.56	1.69	1.46	1.16070391	0.13
1145	12.52	36.95	D4A2G9	RAN-binding protein 1	7	0.89	0.58	0.97	0.64	0.88	0.97238531	0.231
1146	12.52	26.60	B0BNM1	NAD(P)H-hydrate	7	1.05	1.00	1.03	0.99	1.07	1.07088236	0.207
1147	12.51	20.66	A1A5L1	Bleomycin hydrolase	7	1.84	1.37	1.47	1.60	1.45	1.02016323	0.399
1148	12.51	35.10	A0A0H2UHT6	40S ribosomal protein S18	8	1.17	1.20	1.32	1.13	1.20	0.98432047	0.742
1149	12.49	28.30	P21775	3-ketoacyl-CoA thiolase A, peroxisomal	6	1.03	0.96	1.09	1.01	0.96	0.9949735	0.886
1150	12.49	5.20	Q2TL32	E3 ubiquitin-protein ligase UBR4	21	1.03	1.00	0.98	1.01	0.99	1.00716496	0.453
1151	12.47	9.79	A0A0U1RS25	UPF1, RNA helicase and ATPase	7	0.88	0.55	0.84	1.09	0.82	1.03003974	0.0276
1152	12.47	8.64	Q924M0	GABA type B receptor 1f	7	0.94	0.97	0.99	0.95	0.97	1.08372597	0.198
1153	12.46	18.55	D3ZWC6	Syntrophin, basic 1 (Predicted)	7	1.85	1.39	1.66	1.56	1.67	1.02037538	0.484
1154	12.46	19.00	Q56R18	Importin subunit alpha	6	2.51	1.57	0.89	1.46	2.19	1.07251662	0.422
1155	12.45	8.08	A0A0G2KAW[illegible]	Signal-induced proliferation-associated 1 like 1, isoform CRA_b	12	1.14	0.81	1.12	1.54	1.04	1.08824244	0.145
1156	12.45	23.68	P53678	AP-3 complex subunit mu-2	10	0.92	0.58	0.65	0.76	0.77	0.98411581	0.64
1157	12.44	34.96	P60901	Proteasome subunit alpha type-6	8	1.21	0.75	1.29	0.84	1.34	1.00242896	0.957
1158	12.41	11.05	A0A0G2K9A9	F-box protein 41	7	0.93	0.89	0.65	0.85	0.77	1.02079983	0.34
1159	12.39	11.74	F1LRS5	Amyloid beta precursor-like protein 1	9	0.92	0.98	1.28	0.76	1.12	0.90940825	0.00473
1160	12.36	5.99	D3ZZB0	Disco-interacting protein 2 homolog C	8	1.07	1.04	0.73	0.99	0.99	0.83682624	0.18
1161	12.34	5.00	D3ZQ57	Plexin B2	7	0.86	0.82	1.21	1.05	1.05	1.06466533	0.614
1162	12.32	30.39	B1WBW4	Armadillo repeat-containing protein 10	7	0.98	1.02	1.00	1.07	0.99	1.00884183	0.514
1163	12.3	26.27	P18422	Proteasome subunit alpha type-3	9	1.01	1.01	0.97	0.98	0.94	0.91700404	0.0212
1164	12.3	22.29	B2RYQ2	Serine/threonine-protein phosphatase 2A activator	8	1.00	1.07	0.93	0.92	0.93	0.97380175	0.778
1165	12.28	7.36	P97874	Cyclin-G-associated kinase	9	1.10	1.32	1.19	0.82	0.85	1.01656326	0.514
1166	12.27	13.33	F1LYZ8	Protein phosphatase 1, regulatory subunit 21	7	0.90	1.05	0.97	0.63	0.95	1.03634187	0.315
1167	12.22	13.82	F1MAA5	RAN GTPase-activating protein 1	8	1.09	1.09	1.39	1.00	1.21	0.99048017	0.868
1168	12.22	8.22	D3ZAZ1	Formin-like 1	6	0.67	0.96	0.89	0.90	0.79	1.04145464	0.218
1169	12.22	33.08	G3V6X7	ProSAAS	8	0.69	0.63	0.69	0.28	0.58	0.76684133	0.107
1170	12.21	13.45	A0A0H2UHV9	Coatomer subunit gamma	9	0.79	0.75	1.46	0.95	1.38	0.99798498	0.817
1171	12.21	70.93	D3ZD09	Cytochrome c oxidase subunit	9	0.53	0.45	0.90	0.81	0.86	0.95138422	0.431
1172	12.19	39.07	Q6PDV6	40S ribosomal protein S14-like	6	0.90	0.95	1.20	1.13	1.15	1.01452189	0.599
1173	12.17	6.48	M0R6K4	Dedicator of cytokinesis 4	10	0.50	0.96	1.14	1.06	1.79	1.01220394	0.622
1174	12.14	15.56	P0C5X8	Protein tweety homolog 1	8	1.06	0.93	1.08	0.93	0.85	0.94115223	0.166
1175	12.13	7.29	P23347	Anion exchange protein 2	7	0.38	0.26	0.30	0.47	0.67	0.98582263	0.498
1176	12.12	18.54	O55171	Acyl-coenzyme A thioesterase 2,	8	0.73	0.76	0.64	0.61	0.52	1.0097513	0.802
1177	12.11	8.33	A0A0G2K5D7	Sperm antigen with calponin homology and coiled-coil domains 1	9	1.42	1.47	1.56	1.32	1.74	1.03662925	0.261
1178	12.11	23.55	D3ZI16	C	9	0.89	0.29	1.18	1.05	0.83	1.00219969	0.935
1179	12.1	18.11	P21531	60S ribosomal protein L3	7	0.95	1.17	1.39	1.32	1.41	0.99958143	0.978
1180	12.09	25.42	Q5FVM4	Non-P	13	1.32	1.13	1.34	0.98	1.66	1.0442014	0.159
1181	12.09	20.10	Q6AYT7	Monoacylglycerol lipase ABHD12	7	0.96	0.86	0.96	1.33	0.79	0.97393676	0.411
1182	12.08	16.45	Q01066	Calcium/calmodulin-dependent 3',5'-cyclic nucleotide phosphodiesterase 1B	7	0.90	0.82	0.97	0.41	0.46	0.98664295	0.693
1183	12.07	29.74	B2RZC6	Ilf2 protein	7	1.13	1.07	0.96	0.90	0.98	0.97400427	0.0303
1184	12.04	47.27	P23358	60S ribosomal protein L12	8	0.87	0.62	1.16	1.13	1.02	1.08598186	0.0145
1185	12.03	31.80	O70257	Syntaxin-7	6	1.18	1.14	1.00	0.65	1.09	0.90187538	0.111
1186	12.02	25.16	A0A0G2K7W7	Glycogen synthase kinase-3 alpha	8	1.06	0.62	0.86	1.45	1.18	0.99578763	0.858
1187	12.01	27.67	A0A0A0MY48	Dynamin-2	35	1.91	1.31	1.57	1.50	1.63	0.89502507	0.0565
1188	12.01	30.06	Q9JJ19	Na(+)/H(+) exchange regulatory cofactor NHE-	10	1.04	1.02	1.74	0.72	1.38	0.98979386	0.91
1189	12	23.22	A0A096MK30	Moesin	14	0.51	1.47	1.03	1.09	0.82	1.09505247	0.0967
1190	12	47.37	B2GV99	Myl6 protein	7	1.54	0.90	1.54	0.46	1.38	0.90940825	0.321
1191	12	29.93	A2RUW1	Toll-interacting protein	6	0.77	1.11	0.99	0.71	1.07	1.0341891	0.0655
1192	11.97	16.18	B2RYP4	Sorting nexin 2	7	0.98	1.01	0.99	0.99	1.00	1.05445681	0.0283

1193	11.97	22.29	O70593	Small glutamine-rich tetratricopeptide repeat-containing protein alpha	8	1.20	0.83	1.39	0.73	1.19	0.90062598	0.0587
1194	11.96	10.99	Q62825	Exocyst complex component 3	7	1.03	1.05	1.00	1.03	1.06	1.00240812	0.944
1195	11.96	27.30	Q63768	Adapter molecule crk	6	1.38	1.60	1.07	0.54	1.63	0.94631999	0.0833
1196	11.94	47.30	D3ZDU5	Profilin	10	0.93	2.44	2.03	2.73	2.94	1.12896441	0.0288
1197	11.93	19.28	C9WPN6	Eukaryotic translation initiation factor 2 subunit 3, Y-linked	7	1.03	0.98	1.08	1.07	0.94	1.01825578	0.309
1198	11.92	54.45	P35332	Hippocalcin-like protein 4	12	2.01	0.48	0.70	0.76	0.79	0.93958787	0.147
1199	11.91	27.51	F1LMW7	Myristoylated alanine-rich C-kinase substrate	7	1.29	1.38	1.42	0.80	0.89	0.72597914	0.123
1200	11.89	26.67	Q4G079	Aminoacyl tRNA synthetase complex-interacting multifunctional protein 1	8	1.11	1.06	1.04	0.74	1.13	1.03677296	0.205
1201	11.89	19.89	F1LP57	Mitogen-activated protein kinase kinase 4	7	0.99	0.99	1.00	0.97	1.05	0.99504936	0.765
1202	11.89	14.63	Q5I0D7	Xaa-Pro dipeptidase	5	1.11	1.16	1.51	1.31	1.15	1.02484093	0.624
1203	11.89	31.56	F7EMB2	Putative uncharacterized protein RGD1304704	7	0.99	1.50	1.24	0.65	1.11	0.93698639	0.239
1204	11.88	10.59	Q3ZAV8	Enhancer of mRNA-decapping protein 4	9	1.02	1.04	0.98	1.02	0.99	0.93828623	0.0735
1205	11.87	12.33	G3V743	Mannosyl-oligosaccharide glucosidase	7	0.91	0.86	0.90	0.81	0.74	1.02030466	0.0376
1206	11.86	7.58	F1LMB9	Exocyst complex component 2	7	0.90	0.95	0.90	0.92	0.89	1.0287554	0.215
1207	11.86	57.89	Q9EQX9	Ubiquitin-conjugating enzyme E2 N	7	0.98	1.16	0.99	0.76	1.26	0.94737007	0.127
1208	11.86	42.11	P84817	Mitochondrial fission 1 protein	8	0.79	0.86	1.50	1.18	1.31	0.95879879	0.483
1209	11.83	22.02	Q4FZT2	Protein phosphatase methylesterase 1	9	0.98	1.02	1.06	1.01	1.07	0.91700404	0.0287
1210	11.81	15.95	F7EV94	2-oxoisovalerate dehydrogenase subunit alpha, mitochondrial	8	0.90	0.96	1.00	0.87	0.91	0.96808122	0.368
1211	11.8	31.34	Q66HR2	Microtubule-associated protein RP/EB family member 1	8	0.82	1.51	1.38	1.53	1.31	1.04964393	0.00488
1212	11.77	19.78	Q8R3Z7	EH-domain containing 4	10	0.72	1.04	1.18	0.59	0.98	0.96553469	0.301
1213	11.77	11.94	Q63151	Long-chain-fatty-acid--CoA ligase 3	6	0.93	0.90	0.90	0.92	1.04	1.02391787	0.392
1214	11.76	18.08	B2GUY4	Dematin actin-binding	5	1.34	1.43	0.52	0.88	1.47	0.9599958	0.249
1215	11.75	24.71	Q99MZ8	LIM and SH3 domain protein 1	8	0.89	1.67	1.43	1.13	0.95	0.94953955	0.0707
1216	11.72	20.91	A0A096MKD[illegible]	Tripartite motif-containing 46 (Fragment)	6	0.90	0.91	0.94	0.93	0.93	0.91383145	0.0435
1217	11.71	34.23	B0BN85	Protein SGT1 homolog	9	0.96	0.96	1.09	1.01	1.01	1.08147476	0.00441
1218	11.69	21.72	P25093	Fumarylacetoacetase	7	0.90	0.52	1.19	0.98	0.86	1.02327932	0.701
1219	11.67	22.88	F7EUU4	C	6	1.18	0.93	0.95	0.92	1.05	1.02122446	0.186
1220	11.66	33.45	Q68FR9	Elongation factor 1-delta	8	0.93	1.24	1.34	0.77	0.99	0.9695586	0.566
1221	11.64	18.82	Q1HCL7	NAD kinase 2,	6	0.79	0.43	0.68	0.48	0.76	0.96152748	0.0447
1222	11.64	25.41	D3ZW08	Adenylosuccinate lyase	10	1.17	1.29	1.14	1.29	1.26	0.96486567	0.267
1223	11.63	19.01	Q498N4	Phosphatidylinositol binding clathrin assembly protein, isoform CRA_c	13	1.00	0.99	0.95	1.01	0.99	0.99027423	0.801
1224	11.63	48.45	D3ZS58	NADH dehydrogenase [ubiquinone] 1 alpha subcomplex subunit 2	9	0.53	1.38	1.06	1.07	0.83	0.92274249	0.0766
1225	11.6	22.54	Q5M9F7	ARP10 actin-related protein 10 homolog (S. cerevisiae)	8	1.24	0.57	1.04	0.97	1.12	0.88025901	0.029
1226	11.6	10.84	A0A0G2K707	Diacylglycerol kinase	6	1.03	1.01	0.92	0.98	1.00	0.94317671	0.4
1227	11.58	24.47	A0A096MIZ1	Paralemmin 2	7	1.00	1.04	1.04	0.99	1.03	0.96252772	0.0875
1228	11.57	20.13	D3ZT90	Glutaryl-CoA	9	0.79	0.95	1.50	2.38	1.49	0.97589646	0.308
1229	11.57	21.10	P07150	Annexin A1	7	0.81	1.21	2.96	0.99	1.21	0.99369155	0.843
1230	11.56	10.40	M0R6E0	Phospholipid-transporting ATPase	8	1.02	1.04	1.01	1.02	1.04	1.1188371	0.133
1231	11.55	14.49	A0A0G2K785	Glycerol kinase	6	0.90	0.91	1.09	0.94	0.67	1.0073046	0.89
1232	11.55	28.82	P08082	Clathrin light chain B	8	1.26	0.79	1.06	0.29	0.81	0.94036973	0.544
1233	11.54	17.89	Q5M7W7	Probable proline--tRNA ligase, mitochondrial	6	1.04	1.13	1.08	1.04	1.03	0.93044966	0.294
1234	11.53	7.49	G3V700	Nardilysin, N-arginine dibasic convertase 1	6	1.27	0.82	1.15	1.20	1.58	1.03799536	0.069
1235	11.53	20.39	B0BNG3	Lectin, mannose-binding 2	6	0.61	0.53	0.72	0.76	0.86	1.00542119	0.468
1236	11.53	36.08	P62083	40S ribosomal protein S7	6	0.99	1.69	1.91	0.84	1.80	0.99647119	0.95
1237	11.5	23.18	Q9Z2X5	Homer protein homolog 3	9	1.19	0.90	1.38	1.00	1.84	0.95356289	0.367
1238	11.5	12.61	F1LPP6	A-kinase anchor protein 5	7	0.95	1.02	0.90	0.76	0.72	0.98945088	0.606
1239	11.48	26.26	D4A5X7	Ganglioside-induced differentiation-associated-protein 1	9	0.80	1.24	0.90	1.03	1.06	0.94278454	0.0246

1240	11.47	22.86	Q5XIG8	Serine-threonine kinase receptor-associated protein	6	0.96	1.06	1.05	0.98	1.04	1.00905163	0.718
1241	11.46	15.40	G3V7V3	Solute carrier family 27 (Fatty acid transporter), member 4	8	0.59	0.76	0.67	0.36	0.72	1.07997656	0.182
1242	11.45	34.03	G3V7B5	Phosphoribosyl pyrophosphate synthase-associated protein 1	10	0.86	1.19	0.95	0.87	1.02	1.00457133	0.908
1243	11.43	24.62	Q64303	Serine/threonine-protein kinase PAK 2	14	0.94	0.92	1.20	1.01	1.01	1.06895417	0.0988
1244	11.43	9.39	A0A0G2JV52	Complement component 4A (Rodgers blood group) (Fragment)	8	2.01	2.38	2.36	1.80	1.63	1.07997656	0.306
1245	11.42	16.28	O55173	3-phosphoinositide-dependent protein kinase 1	7	1.01	0.97	1.04	1.00	1.03	1.05401836	0.136
1246	11.42	11.37	Q6MG06	Guanine nucleotide-binding protein-like 1	7	1.00	0.95	0.95	0.95	0.94	0.92658806	0.0438
1247	11.4	9.35	Q5FVQ8	NLR family member X1	7	1.08	0.95	1.11	1.11	0.87	0.93711629	0.00277
1248	11.39	59.82	A9UMV9	NADH:ubiquinone oxidoreductase subunit A7	9	1.10	1.05	1.47	1.84	1.89	0.98159513	0.529
1249	11.39	29.35	Q497B0		5	0.83	0.22	0.59	0.90	0.76	0.96700818	0.324
1250	11.36	33.81	B0BMY7	Protein tyrosine kinase 9-like (A6-related protein) (Predicted), isoform CRA_b	8	0.95	0.96	1.02	1.03	1.02	0.99824096	0.947
1251	11.34	16.88	A0A140TAF2	ELAV-like protein	6	0.99	1.00	0.85	0.94	0.80	0.96186078	0.221
1252	11.33	42.41	Q8CFN2	Cell division control protein 42 homolog	8	0.63	1.25	1.06	0.95	1.10	1.16473359	0.0916
1253	11.33	32.39	D3ZCR3	High-mobility group (nonhistone chromosomal) protein 1-like 1	8	1.19	1.69	2.03	1.79	2.05	1.15668818	0.0295
1254	11.3	10.95	F1LM55	Cell cycle and apoptosis regulator 2	7	0.99	0.95	0.94	0.90	0.95	0.93919719	0.157
1255	11.29	7.09	F1MAK3	Rho GTPase-activating protein 32	11	1.00	0.98	1.00	1.03	0.98	1.01143247	0.753
1256	11.28	46.98	Q5FVI4	Cell cycle exit and neuronal differentiation protein 1	10	0.77	1.49	1.31	1.57	1.14	0.99240438	0.945
1257	11.28	20.82	Q4FZU0	Acid phosphatase 6, lysophosphatidic	7	0.99	1.00	0.94	0.95	0.95	0.83973149	0.0399
1258	11.25	57.66	P52759	Ribonuclease UK114	6	1.39	1.17	2.56	0.47	2.19	1.01171294	0.874
1259	11.25	14.58	D3ZUX7	Acyl-CoA synthetase family member 3	6	0.41	0.48	0.53	0.67	0.17	1.02115368	0.209
1260	11.24	42.86	P07483	Fatty acid-binding protein, heart	6	1.43	1.17	1.46	0.60	0.83	0.99797806	0.959
1261	11.24	17.17	B1WBY5	DnaJ (Hsp40) homolog, subfamily C, member 11	8	0.66	0.41	0.40	0.40	0.33	0.91827616	0.00662
1262	11.24	45.14	G3V7I0	Peroxiredoxin 3	12	0.97	0.99	0.91	0.93	0.93	0.89316585	0.0139
1263	11.21	13.92	M0R4V4	Similar to solute carrier family 25 (Mitochondrial carrier; phosphate carrier), member 23, isoform CRA_b	6	1.02	0.73	0.71	0.70	0.50	0.91130128	0.0649
1264	11.21	35.78	B0BN81	40S ribosomal protein S5	9	0.72	0.15	1.09	0.77	0.90	0.97684394	0.748
1265	11.21	42.74	A0A140TA99	Uncharacterized protein	6	0.90	0.43	1.10	0.54	0.95	0.96486567	0.614
1266	11.19	14.98	A0A0G2JVK4	Neurolysin, mitochondrial	9	1.14	1.33	1.32	1.27	1.43	0.99477351	0.716
1267	11.18	29.24	P20070	NADH-cytochrome b5 reductase 3	11	0.77	0.65	0.79	1.03	0.95	0.97792789	0.537
1268	11.16	21.47	D3ZH41	Cytoskeleton-associated protein 4	6	0.92	0.92	1.04	0.99	0.92	1.0324701	0.235
1269	11.14	29.74	P45479	Palmitoyl-protein thioesterase 1	7	0.70	0.66	0.67	0.61	0.67	1.00046105	0.984
1270	11.14	8.08	D3ZX42	G protein-coupled receptor 21 (Predicted), isoform CRA_a	7	2.17	1.09	1.56	1.41	1.96	1.02612039	0.336
1271	11.13	10.25	Q4V8I7	Volume-regulated anion channel subunit LRRC8A	7	0.72	0.91	0.77	0.67	0.70	1.03182621	0.209
1272	11.13	58.62	F1LXA0	NADH dehydrogenase [ubiquinone] 1 alpha subcomplex subunit 12	6	1.05	1.07	1.01	1.03	1.02	0.93750611	0.0744
1273	11.11	8.09	D3ZT36	ADAM metallopeptidase domain 23	8	0.86	0.53	0.88	0.95	0.87	0.97928453	0.532
1274	11.1	16.43	Q5U2N2	Ubiquitin specific peptidase 14	6	1.00	1.20	0.84	0.71	1.09	1.00587428	0.845
1275	11.1	53.52	Q63228	Glia maturation factor beta	7	1.06	1.58	1.31	0.93	1.39	0.9354938	0.152
1276	11.08	25.60	Q6AYR2	Protein NDRG3	6	0.95	0.59	1.08	0.97	0.96	0.96006235	0.368
1277	11.06	8.06	D4ABM3	Dishevelled associated activator of morphogenesis 1 (Predicted)	9	0.72	0.65	0.42	0.77	1.05	1.01311644	0.459
1278	11.06	13.74	Q7TPH4	Dystrophin	7	null	null	null	null	null	1.04275483	0.0729
1279	11.05	26.73	P62907	60S ribosomal protein L10a	7	1.12	1.10	1.79	1.56	1.53	1.06252737	0.00983
1280	11.02	21.68	Q6AZ35	Cytoplasmic dynein 1 intermediate chain 2	7	1.51	1.10	1.09	0.88	0.98	0.90187538	0.0486

1281	11.02	8.37	Q63475	Receptor-type tyrosine-protein phosphatase N2	6	0.85	1.16	1.03	0.88	0.63	1.0387151	0.112
1282	11.01	15.69	M0R402	Thioredoxin-related transmembrane protein 3	8	0.91	1.18	1.01	0.95	0.70	0.9641971	0.0809
1283	11	17.37	Q6AY21	G3BP stress granule assembly factor 2	7	1.06	1.28	0.83	1.07	1.16	0.96092784	0.188
1284	10.98	11.93	O08700	Vacuolar protein sorting-associated protein 45	6	0.92	0.88	0.95	0.86	0.89	0.99819944	0.898
1285	10.98	11.46	D4A0E8	Protein arginine N-methyltransferase 5	6	0.65	0.79	0.65	0.64	0.54	0.92658806	0.0737
1286	10.97	15.24	A0A0G2JSL8	Adenosine kinase	5	0.92	0.79	0.97	1.16	1.13	1.06422264	0.0924
1287	10.96	23.52	Q03346	Mitochondrial-processing peptidase subunit beta	8	0.94	1.01	0.99	0.98	0.97	0.95164804	0.0478
1288	10.96	11.38	P62024	Phosphatase and actin regulator 1	5	0.99	1.03	0.97	0.96	0.98	0.93724622	0.0181
1289	10.96	7.88	D4A857	Importin 9	6	1.01	0.67	0.82	0.86	0.80	1.02519617	0.457
1290	10.96	50.58	B4F7A3	Galectin	6	0.96	1.01	0.82	0.58	0.85	0.90187538	0.0192
1291	10.94	21.73	F1LPE9	ELKS/Rab6-interacting/CAST family member 1	23	0.67	1.00	0.77	0.60	0.82	1.004446	0.798
1292	10.94	14.31	F7F1Y3	Vacuolar protein sorting-associated protein 4A	8	0.93	0.98	0.95	1.04	0.94	0.97752127	0.225
1293	10.94	23.22	D4ADE5	Histone-lysine N-methyltransferase SETD7	6	1.50	1.50	1.20	0.56	1.25	0.92851685	0.0224
1294	10.93	11.66	F1M7X3	Cadherin 13	6	1.50	1.04	0.40	0.60	0.56	1.07922824	0.356
1295	10.93	22.67	Q5XI22	Acetyl-CoA acetyltransferase, cytosolic	8	1.58	1.10	1.17	1.64	1.71	0.9465824	0.0867
1296	10.92	40.76	A0A0H2UI38	40S ribosomal protein S19-like	7	0.94	1.16	1.33	1.29	1.41	1.09202055	0.0122
1297	10.92	56.10	B2RZD6	NADH:ubiquinone oxidoreductase subunit A4	12	0.90	1.63	1.02	1.60	1.09	0.95554783	0.241
1298	10.91	9.44	P31422	Metabotropic glutamate receptor 3	9	0.77	0.84	1.19	0.86	1.00	0.97009639	0.146
1299	10.9	30.05	Q5RK10	60S ribosomal protein L13a	7	1.11	1.60	1.54	1.56	1.60	1.01318667	0.665
1300	10.89	22.36	P13234	Calcium/calmodulin-dependent protein kinase type IV	8	1.51	1.10	1.19	1.45	1.50	1.06304303	0.108
1301	10.89	34.63	P40112	Proteasome subunit beta type-3	5	0.94	1.03	1.00	1.01	0.93	0.98766932	0.646
1302	10.87	13.09	G3V7X5	SPARC-like 1 (Mast9, hevin), isoform CRA_a	6	1.31	1.31	1.54	0.91	1.15	0.91193317	0.0334
1303	10.87	59.78	P04631	Protein S100-B	16	1.19	1.54	2.19	0.27	1.02	0.86035263	0.485
1304	10.86	8.52	A0A0G2K104	Calcium-activated potassium channel subunit	7	1.29	1.20	1.51	1.69	1.60	1.03950738	0.129
1305	10.85	14.71	Q62888	Neuroligin-2	7	1.22	1.34	0.83	1.46	1.22	0.99983435	0.995
1306	10.85	41.40	F8WG91	ADP-ribosylation factor-like protein 3	8	1.72	1.58	1.18	0.72	1.45	0.882703	0.00275
1307	10.85	13.39	A0A0G2K0I3	Nicotinamide phosphoribosyltransferase	5	0.64	0.99	1.21	0.79	0.84	1.02101213	0.343
1308	10.81	33.33	A0A0G2JWK	Transgelin	11	1.04	5.81	1.10	2.78	1.91	1.02363402	0.678
1309	10.81	7.89	Q5M9I2	Alpha-mannosidase	6	0.99	0.65	0.94	0.90	0.49	0.97576119	0.509
1310	10.81	46.51	F1LPG5	NADH:ubiquinone oxidoreductase subunit B4	9	0.84	1.19	1.11	0.83	1.01	0.94239252	0.11
1311	10.79	10.86	Q704S8	Carnitine	6	0.60	1.06	0.89	1.17	1.29	1.01691564	0.538
1312	10.79	50.00	P62832	60S ribosomal protein L23	7	1.32	0.26	1.26	0.91	1.33	1.03146867	0.754
1313	10.78	6.69	Q5RKI5	FLII, actin-remodeling protein	9	0.92	0.92	0.97	0.92	0.92	1.04181564	0.402
1314	10.77	21.46	M0RD75	40S ribosomal protein S6	6	0.90	0.52	1.10	0.89	1.18	1.03806732	0.0141
1315	10.76	32.26	A0A0G2JSQ4	Tropomyosin 1, alpha, isoform CRA_p	11	1.19	1.87	1.26	0.90	1.31	0.89254697	0.398
1316	10.74	7.32	A0A0G2QC02	Ski2-like RNA helicase	8	0.90	0.69	0.85	0.80	1.02	1.12194348	0.042
1317	10.74	16.79	D3ZZC1	RCG43947	6	0.83	0.86	0.79	0.69	0.66	0.96915545	0.266
1318	10.74	17.86	P85125	Polymerase I and transcript release factor	6	1.03	1.56	1.51	1.34	1.64	1.005944	0.779
1319	10.74	61.84	P11951	Cytochrome c oxidase subunit 6C-2	11	0.88	1.50	0.94	1.14	0.64	0.86035263	0.113
1320	10.74	18.29	B1WC33	CDC42 effector protein (Rho GTPase binding) 4 (Predicted), isoform CRA_a	5	0.99	1.05	1.19	0.95	0.99	1.01705662	0.556
1321	10.73	26.67	Q9QX80	CArG-binding factor A	8	1.27	1.41	1.77	1.12	1.75	1.04710056	0.19
1322	10.73	69.01	P29419	ATP synthase subunit e, mitochondrial	6	0.94	1.04	1.10	0.93	0.86	0.94219658	0.392
1323	10.72	10.39	O35142	Coatomer subunit beta'	7	0.95	0.96	0.98	0.96	0.98	0.95753691	0.0231
1324	10.71	46.12	B5DF65	Biliverdin reductase B	7	0.86	1.33	0.86	0.91	1.11	0.89937831	0.0037
1325	10.7	25.27	A0A096MJN4	Septin 4	13	1.41	1.01	1.09	1.24	1.46	1.04869853	0.119
1326	10.7	18.58	G3V774	F-box only protein 2	5	0.95	0.50	1.33	0.86	1.31	1.02448581	0.56
1327	10.67	19.32	M0R7F3	WAS protein family, member 3	8	0.98	0.77	1.06	0.62	0.61	0.93238649	0.00778
1328	10.66	14.48	Q5PQK2	Fused in sarcoma RNA-binding protein	6	1.29	1.33	1.69	1.36	1.66	0.99894005	0.989

1329	10.64	11.89	G3V9X2	G-protein signalling modulator 1 (AGS3-like, C. elegans), isoform CRA_c	6	0.92	0.61	0.66	0.55	0.57	1.0242728	0.371
1330	10.64	20.91	G3V7Q6	Proteasome subunit beta	5	1.21	1.37	1.21	1.27	1.31	0.90375273	0.0171
1331	10.63	58.39	D3ZLT1	NADH dehydrogenase (Ubiquinone) 1 beta subcomplex, 7 (Predicted)	12	1.16	0.36	1.12	0.55	0.92	0.86035263	0.256
1332	10.62	10.88	Q64566	Calcium-transporting ATPase type 2C member 1	9	1.17	1.04	1.14	0.77	1.00	1.01213379	0.464
1333	10.62	8.22	F1M6X3	Potassium voltage-gated channel subfamily KQT member 2	5	1.14	1.38	0.71	0.81	0.90	1.07698638	0.0759
1334	10.62	4.01	F1LMS1	Voltage-dependent R-type calcium channel subunit alpha	7	0.94	0.91	0.90	1.04	0.90	1.05350707	0.0927
1335	10.61	18.47	Q5VLR5	BWK4	8	0.53	0.42	0.86	0.46	1.10	0.96761162	0.568
1336	10.59	18.83	Q566E4	Heterogeneous nuclear ribonucleoprotein R	12	0.97	1.16	0.93	0.92	0.81	0.93588294	0.121
1337	10.58	23.36	Q641W2	UPF0160 protein MYG1, mitochondrial	6	1.00	1.07	1.01	1.02	1.06	0.96132756	0.336
1338	10.57	17.76	P54690	Branched-chain-amino-acid aminotransferase, cytosolic	9	0.41	0.61	0.83	0.65	0.76	0.95660816	0.133
1339	10.56	22.65	A0A0G2K0H(	WD repeat domain 13	5	1.02	1.06	0.93	0.91	0.95	0.97218313	0.0198
1340	10.56	20.96	M0RAD5	ATP-dependent Clp protease proteolytic subunit	9	2.56	2.51	1.64	1.38	1.02	0.90187538	0.029
1341	10.53	27.61	D3ZFQ8	Cytochrome c-1	14	1.14	1.29	1.08	1.10	0.92	0.86814023	0.0223
1342	10.53	10.99	Q66H79	Tripartite motif protein 32	5	0.92	1.03	0.92	0.90	0.87	1.03433248	0.13
1343	10.51	11.33	D4A3S6	MTSS1L, I-BAR domain-containing	6	1.34	0.81	1.60	1.21	1.24	1.04159902	0.248
1344	10.51	24.92	Q3KRD5	Mitochondrial import receptor subunit T	7	1.05	0.65	0.60	0.73	0.70	1.08748839	0.177
1345	10.5	10.05	F1LPA3	IQ motif and SEC7 domain-containing protein 3	9	0.63	0.64	0.56	0.77	0.74	0.9998094	0.99
1346	10.45	18.18	A0A0G2K189	Secernin 3	6	0.98	1.20	0.99	1.03	0.98	1.06252737	0.0818
1347	10.44	11.09	D3ZVU6	AVL9 cell migration-associated	6	1.10	0.96	0.56	0.90	0.98	1.02030466	0.401
1348	10.44	9.80	F1M0J7	Prickle planar cell polarity protein 2	6	1.51	1.41	1.09	1.91	1.74	0.98418402	0.772
1349	10.42	17.08	A0A0G2JTA0	FAS-associated factor 2	6	0.35	0.59	0.65	0.54	0.64	0.97447697	0.321
1350	10.39	7.98	D4A8H3	Similar to RIKEN cDNA 5730469D23 (Predicted)	8	0.95	1.00	0.99	1.03	0.93	1.02832764	0.287
1351	10.39	25.20	Q5HZE4	Methylthioribose-1-phosphate isomerase	6	1.20	1.50	1.11	0.94	1.39	0.98561765	0.553
1352	10.38	13.09	A0A0G2JTH4	Leukocyte surface antigen CD47	8	0.85	1.02	1.03	1.41	0.96	1.08372597	0.0314
1353	10.36	14.21	A0A0G2QC17	Protein phosphatase 2C, magnesium dependent, catalytic subunit, isoform CRA_a	7	0.90	0.99	0.99	0.85	0.86	1.04376722	0.599
1354	10.36	14.95	Q99MZ4	Gamma-glutamyltransferase	7	0.88	1.20	1.16	1.00	1.04	1.04710056	0.513
1355	10.35	28.13	Q62651	Delta(3,5)-Delta(2,4)-dienoyl-CoA isomerase, mitochondrial	8	0.90	1.01	1.27	1.17	1.18	1.02768634	0.617
1356	10.35	38.33	P36972	Adenine phosphoribosyltransferase	5	0.81	0.95	1.14	0.99	0.87	0.9797598	0.349
1357	10.34	25.48	Q68FX9	NAD-dependent protein deacylase sirtuin-5, mitochondrial	7	1.10	0.32	0.87	1.15	0.80	1.01150258	0.635
1358	10.34	30.66	Q5PQJ6	Pyrroline-5-carboxylate reductase 3	7	1.17	1.10	0.98	0.98	0.90	0.90000193	0.0246
1359	10.31	16.19	P56522	NADPH:adrenodoxin oxidoreductase, mitochondrial	6	2.17	1.89	1.15	1.82	1.36	0.96861819	0.0757
1360	10.31	46.97	P55051	Fatty acid-binding protein, brain	7	1.47	1.02	6.25	1.57	3.08	1.43097652	0.0672
1361	10.3	13.10	B1WC49	Api5 protein	5	1.00	1.02	1.02	0.98	1.03	1.00551876	0.865
1362	10.3	31.67	Q6PDW4	Proteasome subunit beta	8	0.97	1.06	1.03	1.06	0.99	0.90062598	0.277
1363	10.3	38.52	Q5I0D5	Phospholysine phosphohistidine inorganic pyrophosphate phosphatase	7	0.51	0.42	0.86	0.72	0.75	0.99598091	0.904
1364	10.29	38.08	Q5FVI3	Leucine-rich repeat-containing protein 57	7	1.14	0.91	1.24	1.36	1.46	1.10419885	0.00557
1365	10.28	10.12	A0A0G2K6X(	Non-specific serine/threonine protein	7	1.07	1.14	1.15	1.07	1.12	1.01255481	0.637
1366	10.28	13.90	Q3B7U9	Peptidyl-prolyl cis-trans isomerase FKBP8	5	0.95	0.98	1.17	1.54	1.22	1.01410005	0.573
1367	10.26	19.31	A0A0G2JWK2	Methyl-CpG-binding protein 2	7	1.03	1.03	1.02	0.97	0.98	1.04304399	0.265
1368	10.26	27.87	Q9Z0J8	Neuronal growth regulator 1	7	1.64	0.82	0.49	0.52	0.38	0.79168587	0.0235
1369	10.25	6.52	F1M1D5	Tubulin folding cofactor D	7	0.99	0.97	0.95	0.97	0.96	0.98589096	0.634

1370	10.25	19.46	D3ZUY0	Retinol dehydrogenase 14 (all-trans/9-cis/11-cis)	5	0.67	1.57	1.31	1.53	1.18	1.03139718	0.615
1371	10.23	9.15	A0A1B0GWW	Ubiquitin protein ligase E3C (Fragment)	7	0.89	0.89	0.91	0.96	0.86	1.02954008	0.421
1372	10.22	26.55	Q66H20	Polypyrimidine tract-binding protein 2	8	0.95	1.01	0.92	0.89	0.89	1.00089456	0.95
1373	10.22	35.00	Q6PCT3	Tumor protein D54	7	0.97	0.95	1.06	0.76	1.03	0.97319445	0.257
1374	10.2	8.45	D4A644	MAP7 domain-containing 1	6	0.99	1.00	1.02	1.04	0.99	1.11961289	0.0329
1375	10.19	30.13	A0A0H2UH9	60S ribosomal protein L24	8	0.87	0.81	1.18	0.89	1.26	0.95753691	0.509
1376	10.18	15.35	B1H267	Sorting nexin-5	6	0.82	1.14	0.79	0.64	0.74	1.01431095	0.261
1377	10.17	27.20	Q6AYN4	Phytanoyl-CoA hydroxylase-interacting	12	0.98	0.84	1.45	1.10	1.19	1.06503438	0.0218
1378	10.17	15.16	Q5FVH2	Phospholipase D3	6	0.78	1.17	0.77	0.85	0.74	0.90877812	0.0036
1379	10.16	5.31	F1MAA1	Ubiquitin-specific peptidase 47	6	0.98	1.07	1.00	1.00	1.05	1.07848043	0.0931
1380	10.15	59.47	Q6AY56	Tubulin alpha-8 chain	175	0.50	1.18	0.27	0.70	0.86	0.96814833	0.411
1381	10.15	37.37	Q6AY18	SAR1 gene homolog A (S. cerevisiae), isoform CRA_b	7	0.77	1.34	1.05	1.21	0.99	0.9999643	0.999
1382	10.15	6.92	D3ZD97	DEAH (Asp-Glu-Ala-His) box polypeptide 15 (Predicted), isoform CRA_b	5	0.90	0.97	0.90	0.95	0.92	1.02718782	0.492
1383	10.14	6.26	Q63357	Unconventional myosin-Id	6	0.95	0.95	0.92	0.94	0.96	1.05299603	0.0876
1384	10.14	3.80	P11497	Acetyl-CoA carboxylase 1	8	0.74	0.86	0.60	0.69	0.70	1.02654723	0.487
1385	10.14	15.17	F1LSG0	Neural Wiskott-Aldrich syndrome protein	5	0.90	0.82	0.83	0.53	1.26	0.95488573	0.142
1386	10.14	18.02	Q76EQ0	Serine racemase	5	0.94	1.02	1.01	1.02	0.95	0.94769846	0.202
1387	10.12	82.59	G3V6H0	RAB1B, member RAS oncogene family-like	27	1.08	1.39	1.04	1.27	1.50	0.97880949	0.443
1388	10.12	14.25	A0A0G2JU49	Metaxin 1	7	1.31	0.80	1.07	0.75	0.79	0.94258851	0.0859
1389	10.12	17.21	Q68FS8	RNA terminal phosphate cyclase domain 1	6	1.25	0.47	0.72	0.52	0.95	0.90062598	0.184
1390	10.1	13.52	A0A0G2K4A	RAB11 family-interacting protein 5	6	0.90	0.99	1.07	0.97	1.04	0.94651679	0.038
1391	10.09	16.27	F1LQ55	RCG50466, isoform CRA_a	7	0.91	0.86	1.04	0.90	0.93	1.02654723	0.37
1392	10.08	30.53	P07936	Neuromodulin	6	1.09	1.31	1.00	0.63	0.82	0.70563792	0.00228
1393	10.07	15.44	Q91Z80	Liprin-alpha-4 (Fragment)	11	1.03	1.18	0.95	0.96	1.10	1.02242853	0.272
1394	10.06	22.67	Q62718	Neurotrimin	7	1.09	0.88	0.89	0.91	0.71	0.98609599	0.656
1395	10.05	30.84	P00406	Cytochrome c oxidase subunit 2	11	0.74	1.22	0.51	0.67	0.64	0.90437938	0.0165
1396	10.05	7.70	D4AE06	Peptidylprolyl isomerase	6	1.01	0.91	0.99	0.94	1.03	1.08824244	0.137
1397	10.05	14.50	B4F779	Adaptor protein, phosphotyrosine interaction, PH domain and leucine zipper containing 2	6	1.05	1.01	0.90	1.00	0.94	1.06157036	0.57
1398	10.04	10.68	A0A0G2KA6	ATP-binding cassette subfamily B member 1A	5	0.96	1.06	0.96	0.95	1.01	0.99192297	0.803
1399	10.03	81.76	P69897	Tubulin beta-5 chain	332	1.42	1.96	1.46	1.58	1.74	0.97664083	0.788
1400	10.02	81.33	G3V8K2	Guanine nucleotide-binding protein subunit gamma	12	2.56	0.33	1.02	1.33	1.85	0.90814842	0.574
1401	10	21.00	Q811X6	Lambda-crystallin homolog	7	0.94	1.92	0.90	1.21	1.10	0.9481584	0.169
1402	10	20.22	D4AC36	Eukaryotic translation initiation factor 3 subunit F	6	1.05	0.95	0.94	0.94	0.90	0.88761134	0.0772
1403	10	58.04	P83941	Elongin-C	6	1.42	1.43	1.60	1.26	1.31	1.01445158	0.804
1404	9.99	8.09	D3ZXA6	Pyruvate dehydrogenase phosphatase regulatory subunit	8	1.09	1.05	1.04	0.95	1.01	1.00772361	0.934
1405	9.99	14.92	G3V784	ADP-dependent glucokinase	7	0.76	0.75	1.25	0.75	1.56	1.07997656	0.326
1406	9.97	24.59	B4F774	Ganglioside-induced differentiation-associated protein 1-like 1	8	0.76	0.96	0.64	0.64	0.74	0.92466328	0.0157
1407	9.97	8.37	P10686	1-phosphatidylinositol 4,5-bisphosphate phosphodiesterase gamma-1	8	1.63	1.12	1.21	1.34	0.93	1.03634187	0.00836
1408	9.96	11.37	A0A140TAJ3	Far upstream element-binding protein 1	8	1.32	1.46	1.37	1.26	1.46	0.96976023	0.0169
1409	9.96	12.25	B5DF55	RCG55706	6	1.33	1.61	0.77	0.93	1.29	0.99967358	0.984
1410	9.95	6.52	Q7TQ70	Ac1873	5	0.91	0.86	0.42	0.36	0.52	0.95959663	0.201
1411	9.93	73.91	D4A4D5	Uncharacterized protein	8	0.79	1.46	1.37	0.13	1.12	0.93044966	0.52
1412	9.92	27.25	D3ZX63	Similar to D3Mm3e (Predicted), isoform CRA_a	5	1.00	0.89	0.83	0.81	0.88	0.92787348	0.104
1413	9.91	39.62	Z4YNF4	Low molecular weight phosphotyrosine protein phosphatase	5	1.13	1.80	1.60	1.16	1.79	0.96392981	0.59
1414	9.89	8.73	A0A0G2K9V	Threonine--tRNA ligase, cytoplasmic	6	1.00	1.05	1.02	1.01	1.01	1.02512512	0.7
1415	9.89	7.17	F1LP59	Rab3 GTPase-activating protein catalytic subunit	4	1.00	0.85	0.77	0.85	0.70	0.98787472	0.764
1416	9.89	18.57	P15431	Gamma-aminobutyric acid receptor subunit beta-1	6	0.97	0.92	0.76	0.85	0.91	0.9459265	0.303

1417	9.89	9.41	P31647	Sodium- and chloride-dependent GABA transporter 3	5	0.64	0.58	0.51	0.61	0.41	0.92658806	0.166
1418	9.87	23.75	Q4V8J5	DNA-damage inducible protein 2	5	1.24	1.33	0.91	1.02	1.12	0.9857543	0.604
1419	9.86	37.50	P09495	Tropomyosin alpha-4 chain	11	1.01	1.17	1.06	1.07	1.05	0.8906929	0.382
1420	9.84	13.86	P97756	Calcium/calmodulin-dependent protein kinase kinase 1	8	1.08	1.05	1.01	1.03	1.06	1.03082541	0.111
1421	9.82	11.98	D4A1J6	Ankyrin repeat and FYVE domain-containing 1	9	1.14	0.81	1.39	0.70	0.83	1.0435502	0.0355
1422	9.82	18.70	Q5D023	Cytoplasmic dynein 1 light intermediate chain 2	6	1.10	1.32	1.25	1.45	1.63	1.03154017	0.401
1423	9.81	8.95	D4A772	Dystrobrevin	7	1.11	0.99	1.01	1.10	1.03	0.90187538	0.0242
1424	9.8	29.73	D3ZBN0	Histone H1.5	8	1.19	1.42	1.98	1.17	1.50	1.02661838	0.485
1425	9.8	9.95	O54922	Exocyst complex component 7	6	0.82	1.14	0.89	1.20	1.26	1.07043708	0.161
1426	9.8	19.62	Q5EB62	Solute carrier family 25 member 46	7	0.98	0.98	0.70	1.03	1.22	1.04340554	0.0588
1427	9.8	22.28	P47875	Cysteine and glycine-rich protein 1	8	1.20	1.89	1.72	2.23	1.77	1.00636245	0.806
1428	9.79	34.27	P62268	40S ribosomal protein S23	6	1.17	1.39	1.36	1.53	1.46	1.1320989	0.086
1429	9.77	34.60	F1LY09	ARP3 actin-related protein 3 homolog B	13	2.36	1.66	1.58	2.00	1.43	1.02889802	0.366
1430	9.77	8.11	Q5FWU3	Autophagy-related protein	6	1.05	1.04	1.13	0.95	0.96	0.95913115	0.147
1431	9.77	29.35	D3ZTW9	Endonuclease G-like 1 (Predicted), isoform CRA_d	6	0.67	0.64	0.88	0.49	0.49	0.94343825	0.0824
1432	9.76	27.39	A0A0H2UHZ	Nucleosome assembly protein 1-like 4	12	0.97	0.93	1.00	0.22	0.69	0.96032857	0.285
1433	9.76	18.57	Q5QJC9	BAG family molecular chaperone regulator 5	7	0.92	0.96	0.89	0.84	0.88	0.97928453	0.418
1434	9.76	36.94	B2RYS2	Cytochrome b-c1 complex subunit 7	7	1.29	1.26	1.06	0.86	1.04	0.85916076	0.0109
1435	9.75	22.69	B0BN46	Glyoxylate and hydroxypyruvate reductase	8	0.93	1.02	1.00	1.01	0.99	1.00003909	0.999
1436	9.74	14.99	D3ZUC9		6	0.47	0.81	0.66	0.52	0.50	1.01227411	0.717
1437	9.74	9.51	D4ACM1	Elongator complex protein 3	5	1.08	0.82	0.70	0.53	0.61	0.85559503	0.0814
1438	9.74	34.93	Q6P4Z9	C	5	0.63	1.26	1.03	0.67	1.03	0.93238649	0.0529
1439	9.71	29.53	B0BNJ4	ETHE1, persulfide dioxygenase	6	1.26	1.16	1.08	0.97	1.22	1.00316575	0.93
1440	9.7	27.93	P14942	Glutathione S-transferase alpha-4	8	0.84	1.82	2.47	1.22	0.79	1.11806185	0.319
1441	9.7	29.60	P62853	40S ribosomal protein S25	6	0.89	0.79	1.17	1.21	0.71	1.00420931	0.887
1442	9.69	15.27	A0A0G2K07	Breast carcinoma-amplified sequence 1 homolog	7	1.24	1.08	1.22	1.41	1.57	1.00224137	0.957
1443	9.67	7.33	G3V9H0	Ras GTPase-activating protein 1	6	0.90	0.94	0.95	0.95	0.95	1.07474917	0.0285
1444	9.66	9.21	B5DF63	Sarcolemmal membrane-associated protein	5	0.63	0.65	0.48	0.70	0.69	1.02512512	0.301
1445	9.65	15.80	Q99MI7	NEDD8-activating enzyme E1 catalytic subunit	5	0.82	0.59	1.20	0.67	0.52	1.0807254	0.303
1446	9.64	21.07	Q6AYG5	Ethylmalonyl-CoA decarboxylase	5	1.06	0.97	1.06	1.09	1.11	1.15829281	0.0128
1447	9.63	17.11	B5DFI9	Pdk3 protein	6	0.94	0.98	0.96	0.97	0.90	0.99543568	0.807
1448	9.63	32.84	P40307	Proteasome subunit beta type-2	6	0.79	1.32	1.18	1.14	1.43	0.98609599	0.532
1449	9.63	26.55	Q6AXM8	Serum paraoxonase/arylesterase 2	6	0.63	0.63	0.70	0.79	0.60	0.96292811	0.201
1450	9.62	10.18	P20595	Guanylate cyclase soluble subunit beta-1	5	1.18	1.29	0.86	0.65	1.03	0.97083633	0.56
1451	9.6	10.93	Q5XIN4	Myotubularin related protein 9	6	0.93	1.03	0.95	0.96	0.87	1.10190512	0.261
1452	9.6	22.93	A8WCF8	Tumor protein p63-regulated gene 1-like protein	6	0.89	0.99	0.89	0.86	0.96	1.00709515	0.944
1453	9.59	7.29	A0A0G2JUZ	Myotubularin-related protein 1	5	1.37	1.12	0.89	1.45	1.24	0.99123566	0.714
1454	9.58	12.12	D3ZZZ9	Catenin (Cadherin associated protein), delta 1 (Predicted), isoform CRA_a	9	0.86	0.52	0.90	0.58	0.69	1.06488674	0.0341
1455	9.58	29.79	B0BNE5	S-formylglutathione hydrolase	7	0.90	0.24	0.78	0.82	0.83	1.01178307	0.859
1456	9.57	10.15	D4A6U1	A disintegrin and metallopeptidase domain 11 (Predicted), isoform CRA_a	5	0.95	0.95	0.90	0.90	0.97	0.93174043	0.0327
1457	9.56	18.57	B4F7A9	Casein kinase 2 alpha 2	9	0.98	1.02	0.95	0.99	0.95	0.89502507	0.034
1458	9.56	11.52	Q6IRH7	Caseinolytic peptidase B protein homolog	5	1.14	1.24	0.96	1.11	0.88	0.99758384	0.902
1459	9.56	16.89	Q8CG45	Aflatoxin B1 aldehyde reductase member 2	5	1.14	1.00	0.77	1.00	0.95	0.94901316	0.272
1460	9.55	33.33	A0A0G2JSZ1	Ras-related protein Rab-31	6	1.01	0.92	1.10	0.94	1.01	0.94881584	0.397
1461	9.54	7.75	F1LQP9	Transportin 1	8	1.11	1.01	1.43	0.95	1.16	1.05863114	0.0144

1462	9.54	16.48	F1M7W7	NEDD8-activating enzyme E1 regulatory subunit	6	1.08	1.51	0.56	0.95	1.09	1.06326411	0.146
1463	9.54	37.23	Q642E2	60S ribosomal protein L28	7	0.88	1.38	1.63	1.51	1.66	1.09050773	0.024
1464	9.53	16.03	D3ZC89	Uncharacterized protein	5	1.71	1.38	1.32	0.74	0.95	0.99247317	0.752
1465	9.52	14.60	Q64611	Cysteine sulfinic acid decarboxylase	7	1.04	0.89	1.04	0.95	0.95	0.96466505	0.271
1466	9.51	14.14	Q9QY17	Protein kinase C and casein kinase substrate in neurons 2 protein	6	1.26	1.20	1.03	0.76	0.65	1.04652009	0.211
1467	9.5	11.31	F1LSG8	Syndetin	8	0.92	0.95	0.96	0.97	0.89	1.00392396	0.787
1468	9.5	9.48	A0A0U1RVI2	Mitogen-activated protein kinase	5	1.21	1.07	0.78	0.61	1.13	1.14393097	0.093
1469	9.49	32.71	D3ZTN1	Tescalcin	7	1.02	1.51	1.15	1.25	1.37	0.97501749	0.416
1470	9.48	37.01	Q3T1J1	Eukaryotic translation initiation factor 5A-1	6	2.03	2.49	1.67	0.74	1.57	0.99391198	0.911
1471	9.48	18.78	B0BNB9	HtrA serine peptidase 2	7	1.49	1.45	0.85	0.79	1.33	0.79996013	0.0293
1472	9.47	24.53	E9PT65	Radixin	11	0.73	0.71	0.86	0.85	0.65	1.04152683	0.258
1473	9.47	16.41	P47863	Aquaporin-4	5	1.54	1.72	6.37	4.33	3.28	1.48761376	0.0053
1474	9.46	17.31	Q6P7R8	Very-long-chain 3-oxoacyl-CoA reductase	5	0.67	0.67	1.28	0.79	1.07	1.11496622	0.00096
1475	9.43	5.81	Q00959	Glutamate receptor ionotropic, NMDA 2A	7	1.31	1.37	1.39	1.69	1.84	1.09885422	0.0572
1476	9.43	13.95	P54645	5'-AMP-activated protein kinase catalytic subunit alpha-1	5	1.01	0.99	1.00	0.95	1.01	1.00751408	0.671
1477	9.43	24.29	Q6DGG1	Protein ABHD14B	5	0.98	0.93	0.98	1.04	0.99	0.91955005	0.176
1478	9.4	23.96	Q5XIU9	Membrane-associated progesterone receptor component 2	6	0.74	1.03	0.91	0.65	0.75	0.92594602	0.289
1479	9.39	19.37	Q9ER24	Ataxin-10	9	0.64	0.41	1.13	0.94	0.95	1.01438126	0.207
1480	9.37	5.26	G3V746	Glutamate receptor ionotropic, NMDA 2B	9	0.96	1.18	1.37	1.57	1.53	1.04869853	0.148
1481	9.37	16.41	Q5BJP4	RNA binding motif protein	6	1.37	1.07	1.80	1.25	1.17	0.95131828	0.215
1482	9.36	29.05	A0A0G2K7W(	Uncharacterized protein	6	1.42	0.92	1.19	0.83	1.27	1.03713234	0.0312
1483	9.35	7.97	Q66HA5	Coiled-coil and C2 domain-containing protein 1A	6	1.47	1.07	0.61	1.13	0.86	1.07177346	0.138
1484	9.34	9.61	Q99PV2	Syntaxin binding protein 3	7	1.09	1.28	1.77	2.03	1.79	0.98425224	0.41
1485	9.33	22.26	Q68FQ2	Junctional adhesion molecule C	5	1.09	1.09	1.13	1.41	1.42	1.0222868	0.19
1486	9.33	20.78	F1LNP8	Nectin cell adhesion molecule 1	8	1.47	1.18	0.95	1.21	1.17	0.98657457	0.488
1487	9.33	47.12	P36201	Cysteine-rich protein 2	7	1.98	1.72	1.79	2.47	2.21	1.0167042	0.821
1488	9.32	10.85	D3ZD23	ATP-binding cassette subfamily E member 1	5	1.21	0.66	1.33	1.17	1.00	1.04550503	0.201
1489	9.32	17.15	Q6AYK8	Eukaryotic translation initiation factor 3 subunit D	6	0.97	1.03	0.96	1.01	1.00	1.04942568	0.0225
1490	9.32	28.21	D3ZF13	Acyl carrier protein	7	0.73	0.47	1.14	0.66	1.20	0.74535519	0.283
1491	9.28	6.01	F1LXD6	Phosphatidylinositol transfer protein, membrane-associated 2	6	0.99	1.01	0.95	0.98	0.99	0.97948819	0.512
1492	9.26	10.59	A0A0G2JZV8	Synaptic functional regulator FMR1	6	0.97	1.05	0.96	0.98	0.98	0.92787348	0.0174
1493	9.25	10.08	P25409	Alanine aminotransferase 1	5	1.13	1.57	0.95	1.03	0.85	0.882703	0.0155
1494	9.25	37.41	Q6PEC0	Bis(5'-nucleosyl)-tetraphosphatase [asymmetrical]	5	0.96	0.97	1.08	0.97	0.98	0.93174043	0.109
1495	9.22	13.05	D3ZWW5	RCG64242	6	0.92	0.98	0.64	1.15	0.81	1.00772361	0.825
1496	9.22	16.38	Q62747	Synaptotagmin-7	5	1.46	0.94	1.22	1.60	1.22	1.03239854	0.339
1497	9.22	15.23	D4A3C2	Similar to RIKEN cDNA 6430548M08	7	1.26	1.25	1.41	1.49	1.14	0.95475336	0.123
1498	9.22	26.42	B0BNE6	NADH dehydrogenase (Ubiquinone) Fe-S protein 8 (Predicted), isoform CRA_a	7	0.79	0.42	0.82	0.42	0.97	0.92916067	0.154
1499	9.2	9.70	Q66HR5	Calcium-binding and coiled-coil domain-containing protein 1	6	1.16	1.36	0.82	0.72	0.82	1.01332714	0.598
1500	9.19	6.99	D4A4L4	MAP7 domain-containing 2	4	0.88	1.08	0.83	0.76	1.14	1.04470817	0.262
1501	9.18	24.24	A0A0G2K4X8	S-phase kinase-associated protein 1	5	1.26	1.10	1.67	0.61	1.43	0.97752127	0.543
1502	9.17	7.02	A0A0A0MXV	Gamma-aminobutyric acid type B receptor subunit 2	6	1.12	1.10	1.01	0.89	0.95	1.00968131	0.78
1503	9.17	12.68	P29066	Beta-arrestin-1	5	1.27	1.56	2.11	1.94	1.58	1.05562689	0.115
1504	9.16	22.11	A0A0G2JVB6	Syntaxin-12	9	1.22	1.32	0.96	0.93	0.87	0.94500902	0.368
1505	9.14	50.41	A0A0G2K0T6	Gamma-synuclein	5	1.03	2.42	1.42	0.68	2.13	0.94461608	0.322
1506	9.13	16.16	Q6AYE2	Endophilin-B1	5	0.98	1.04	1.00	1.00	0.99	1.04232125	0.149
1507	9.12	19.09	A0A0G2KAZ	Heterogeneous nuclear ribonucleoprotein D-like	7	0.54	0.71	0.70	0.74	0.69	0.99676824	0.741
1508	9.12	6.07	M0R8V0	UHRF1-binding protein 1-	6	1.15	1.09	1.18	1.10	1.09	1.04001187	0.158
1509	9.12	6.24	A0A0G2JZ27	Uncharacterized protein	6	1.09	0.98	1.01	1.07	1.01	1.0561392	0.078
1510	9.12	23.89	D4AA35	Acetylserotonin	5	1.15	1.13	1.21	1.05	1.04	0.9347808	0.46

1511	9.11	36.22	P62747	Rho-related GTP-binding protein RhoB	10	1.51	0.09	1.15	1.12	1.03	1.00473846	0.981
1512	9.1	57.56	P63029	Translationally-controlled tumor protein	7	0.84	0.88	1.50	0.33	1.63	0.99869081	0.992
1513	9.09	26.69	Q3B8Q0	Microtubule-associated protein RP/EB family member 2	6	1.17	1.56	1.02	1.18	1.38	0.99893312	0.94
1514	9.08	8.53	Q62780	Probable ATP-dependent RNA helicase DDX46	7	0.98	1.03	1.10	1.04	0.99	1.02448581	0.456
1515	9.07	15.54	G3V8W2	Phosphatidate cytidylyltransferase	6	1.07	1.21	0.61	1.13	1.16	0.95032969	0.0966
1516	9.06	9.85	G3V709	Nicotinate phosphoribosyltransferase	6	0.90	1.64	2.70	1.63	1.18	1.02455682	0.817
1517	9.02	4.01	F1LRI5	GCN1 eIF2 alpha kinase activator-like 1	8	0.95	0.96	0.98	1.03	0.95	1.06569899	0.0262
1518	8.99	20.86	E9PTW1	Secretory carrier-associated membrane protein	7	1.09	1.32	0.66	1.15	0.93	0.96339544	0.342
1519	8.98	10.07	D4A1R8	Copine-1	5	1.03	1.74	1.19	1.00	1.66	0.96006235	0.202
1520	8.98	25.74	M0RBX6	Histone H3	5	1.42	1.17	0.62	0.77	0.63	0.59254639	0.02398
1521	8.96	15.83	Q812E9	Neuronal membrane glycoprotein M6-a	7	1.63	0.11	1.39	1.49	1.09	1.06090833	0.789
1522	8.95	6.07	A0A0G2K132	Formin-like 2	6	1.07	1.10	1.12	1.05	1.08	0.91130128	0.0348
1523	8.95	7.44	F1LYQ8	FERM, RhoGEF and pleckstrin domain-containing protein 1	7	1.02	1.01	1.03	1.03	0.97	0.99274838	0.802
1524	8.95	18.16	B2RYL4	Armadillo repeat-containing	6	1.21	0.93	0.98	0.79	0.99	0.9797598	0.688
1525	8.92	57.94	P62959	Histidine triad nucleotide-binding protein 1	7	0.82	0.63	1.10	0.85	1.37	1.00450866	0.901
1526	8.9	11.55	P18886	Carnitine	5	0.71	0.52	0.74	0.85	1.01	0.97056719	0.673
1527	8.9	32.69	F1LT35	Uncharacterized protein	5	0.87	1.14	1.37	1.06	1.37	1.09202055	0.128
1528	8.9	42.50	D3ZYX8	Cytochrome c oxidase subunit 7A2-like	6	1.05	1.21	0.83	1.21	0.88	0.8962667	0.153
1529	8.88	24.71	A0JPM9	Eukaryotic translation initiation factor 3 subunit J	7	1.20	0.57	1.05	0.59	0.94	1.00919153	0.787
1530	8.88	5.24	F1LQ26	Rap guanine nucleotide exchange factor 4	5	0.99	1.09	0.99	1.03	0.95	1.10190512	0.0111
1531	8.87	4.92	Q7TP36	Protein Shroom2	7	0.65	0.55	0.29	0.39	0.62	1.03132569	0.0556
1532	8.87	16.99	Q68FT1	Ubiquinone biosynthesis protein C	7	1.36	1.20	1.22	0.65	1.01	0.93238649	0.0516
1533	8.87	7.77	D3ZZN3	Acetyl-coenzyme A synthetase	5	0.82	0.59	1.20	0.70	0.85	1.18345102	0.0138
1534	8.86	4.03	Q5QD51	A-kinase anchor protein 12	5	1.29	0.94	0.65	1.37	0.65	0.99604305	0.946
1535	8.86	28.66	Q0ZFS8	Putative uncharacterized protein	4	1.21	1.28	1.27	0.73	0.97	0.85559503	0.014
1536	8.85	8.54	Q6Q629	Inactive dipeptidyl peptidase 10	7	0.92	0.86	0.76	0.99	0.77	1.07400447	5.7E-05
1537	8.84	11.85	F1MAR6	Proline dehydrogenase 1	5	0.86	0.50	0.65	0.76	0.54	0.96868533	0.564
1538	8.83	14.57	Q66HJ7	Receptor-type tyrosine-protein phosphatase	11	2.25	2.07	0.99	1.74	0.77	0.95039556	0.117
1539	8.83	18.00	Q9WVR7	Protein phosphatase 1F	5	1.01	0.86	1.54	0.88	1.10	0.96346222	0.585
1540	8.83	19.34	D4ABI7	Very-long-chain (3R)-3-hydroxyacyl-CoA dehydratase	6	0.97	0.94	1.07	1.18	0.95	0.96039514	0.233
1541	8.83	9.30	F1LV89	Rap1 GTPase-activating protein	5	0.69	0.79	0.82	0.87	0.94	0.97887734	0.296
1542	8.81	21.09	Q6QI86	Haloacid dehalogenase-like hydrolase domain-containing protein 2	7	1.15	0.96	0.53	0.93	1.17	0.96620418	0.157
1543	8.81	20.29	D3ZXF9	Mitochondrial ribosomal protein L12	6	1.12	1.13	1.19	0.70	0.94	1.03986771	0.133
1544	8.81	25.26	I6L9G6	RCG31562, isoform CRA_c	4	1.27	0.31	0.82	1.12	1.26	1.05679826	0.173
1545	8.8	63.64	Q6PDW1	40S ribosomal protein S12	7	0.59	0.34	0.88	0.65	1.14	0.96526703	0.5
1546	8.78	20.63	Q6AXQ0	SUM	6	0.93	1.61	1.54	1.17	1.14	1.00737442	0.837
1547	8.77	19.94	D4A4T9	Cysteine and histidine-rich domain-containing protein 1	5	1.80	0.76	1.89	1.39	1.77	1.011082	0.786
1548	8.76	45.07	Q6P3V8	Eukaryotic translation initiation factor 4A1	21	1.06	1.51	1.63	1.64	1.80	0.98472992	0.536
1549	8.76	5.56	D4ACS0	Protein phosphatase 1 regulatory subunit 12A	5	1.00	1.03	1.00	0.97	1.06	1.02377594	0.392
1550	8.76	9.32	F1M6T6	Protein phosphatase 6, regulatory subunit 2	7	0.86	0.81	1.01	0.94	0.79	1.01621101	0.482
1551	8.76	16.33	Q5RKH2	Galactokinase 1	5	0.99	0.97	0.89	0.90	0.88	0.89937831	0.142
1552	8.75	10.42	Q99JE4	Ras-specific guanine nucleotide-releasing factor 2	10	1.01	0.70	0.81	1.13	1.09	0.98309313	0.569
1553	8.75	28.05	Q4FZT0	Stomatin-like protein 2, mitochondrial	8	0.52	0.67	0.47	0.33	0.44	0.99572551	0.947
1554	8.73	12.52	F1LSC3	Splicing factor 1	6	0.53	0.92	0.52	0.64	0.42	0.86035263	0.00987
1555	8.72	16.09	F1LR71	ASPSCR1, UBX domain-containing tether for	5	1.05	1.04	1.07	1.02	1.02	0.89564567	0.0783
1556	8.71	8.88	Q9ES54	Nuclear protein localization protein 4 homolog	6	1.01	1.03	0.97	1.04	1.05	0.97853815	0.565

1557	8.71	19.87	Q5RJR8	Leucine-rich repeat-containing protein 59	6	1.37	0.67	0.99	0.79	0.94	0.94782985	0.259
1558	8.69	20.89	Q9Z1B2	Glutathione S-transferase Mu 5	9	0.97	0.63	1.02	0.95	1.02	0.94317671	0.0216
1559	8.69	11.30	Q62991	Sec1 family domain-containing protein 1	5	1.05	1.01	0.98	1.02	0.96	1.06024671	0.307
1560	8.69	14.58	D4ACG2	IlvB (Bacterial acetolactate synthase)-like (Predicted), isoform CRA_c	5	0.93	0.99	1.15	0.97	0.95	1.06562512	0.174
1561	8.69	20.43	Q5I0E7	Transmembrane emp24 domain-containing protein 9	4	0.81	0.59	0.80	0.81	0.76	0.93659679	0.233
1562	8.69	29.14	Q5XIF3	NADH dehydrogenase [ubiquinone] iron-sulfur protein 4, mitochondrial	5	0.83	1.36	0.94	1.28	0.86	0.89192852	0.098
1563	8.68	13.02	G3V640	Mitochondrial import inner membrane translocase subunit TIM44	6	0.97	0.98	0.92	0.79	0.78	1.00019202	0.993
1564	8.68	17.69	P52555	Endoplasmic reticulum resident protein 29	4	1.19	1.12	1.43	0.73	1.18	0.98472992	0.596
1565	8.68	38.79	B0BNL2	Peptidyl-prolyl cis-trans isomerase	6	0.79	1.07	0.61	0.76	0.79	0.94082611	0.0067
1566	8.67	13.40	A0A0H2UHI7	ATPase family AAA domain-containing protein 1	4	1.13	0.58	0.92	0.88	1.00	1.01832636	0.479
1567	8.67	11.29	B5DFB2	RB-binding protein 4, chromatin-remodeling factor	5	1.09	0.97	1.12	0.33	1.17	1.00765376	0.769
1568	8.67	7.21	A0A0G2JYC6	RCG62582, isoform CRA_c	6	1.05	0.98	0.95	0.95	0.94	1.02363402	0.47
1569	8.66	10.12	E9PU28	Inosine-5'-monophosphate dehydrogenase 2	4	0.97	0.91	0.99	0.96	0.99	1.02847021	0.0447
1570	8.66	7.12	A0A0G2JXR9	Membrane-associated guanylate kinase, WW and PDZ domain-containing protein 2	5	1.06	1.03	0.96	1.05	1.01	1.056066	0.088
1571	8.66	3.13	A0A0G2JWL3	Neurofibromin	7	1.46	0.92	1.14	0.83	0.70	1.04282712	0.115
1572	8.65	6.89	G3V8Y8	Huntingtin interacting protein 1, isoform CRA_a	6	1.09	1.15	1.08	1.01	1.06	1.02044611	0.526
1573	8.65	7.62	D3ZPR0	Chromosome segregation 1-like	7	1.00	0.82	1.13	0.95	1.03	1.00821268	0.801
1574	8.64	12.06	Q63604	BDNF/NT-3 growth factors receptor	9	1.36	0.51	1.20	1.06	1.04	1.04196008	0.288
1575	8.63	26.71	P01830	Thy-1 membrane glycoprotein	10	1.46	0.40	0.77	1.03	0.72	0.89192852	0.135
1576	8.62	15.70	P54287	Voltage-dependent L-type calcium channel subunit beta-3	6	0.99	1.41	0.79	0.69	0.86	1.00256794	0.937
1577	8.62	9.82	D3ZG95	Mitochondrial calcium uptake family, member 3	4	0.70	1.96	1.02	1.41	1.10	0.95369509	0.283
1578	8.62	14.11	M0RDK9	Uncharacterized protein	5	0.90	1.24	0.76	1.05	0.84	0.95813444	0.276
1579	8.61	6.83	D4AAM0	Transportin 3	5	1.03	0.90	1.02	1.06	1.00	0.98507126	0.756
1580	8.59	25.63	D3ZHX3	DIRAS family GTPase 2	5	0.90	1.36	1.29	1.29	1.22	0.99777056	0.909
1581	8.59	7.28	Q4G061	Eukaryotic translation initiation factor 3 subunit B	5	1.07	0.99	0.96	1.03	1.05	1.01747969	0.643
1582	8.58	6.95	G3V8E2	Striatin-interacting protein 1	4	0.95	0.88	1.11	0.88	0.74	1.04804452	0.0402
1583	8.58	41.91	P61354	60S ribosomal protein L27	7	1.06	1.24	1.13	1.07	1.26	1.03361578	0.0627
1584	8.57	6.95	G3V9K0	Cysteinyl-tRNA synthetase	4	1.00	1.29	1.12	0.89	0.72	1.07698638	0.0202
1585	8.57	12.13	O88637	Ethanolamine-phosphate cytidylyltransferase	4	1.39	0.64	1.19	0.80	1.43	1.01733865	0.577
1586	8.57	10.13	A0A0G2KAW	WD repeat domain 48	5	1.32	1.41	1.94	1.11	1.60	0.97238531	0.474
1587	8.55	31.43	P11232	Thioredoxin	6	0.63	1.05	1.41	0.36	1.46	0.96908828	0.776
1588	8.53	12.36	A0A0G2JT06	C	5	1.06	1.07	1.06	1.08	1.06	1.01297601	0.618
1589	8.53	7.38	B4F795	Choline transporter-like protein 2	5	1.20	0.35	1.74	1.13	1.28	0.94782985	0.035
1590	8.53	22.12	Q5RKJ7	Muscle RAS oncogene homolog	4	1.05	1.20	1.11	1.19	0.93	0.91955005	0.0283
1591	8.52	15.01	P18088	Glutamate decarboxylase 1	9	0.90	0.70	0.65	0.80	0.67	0.9727898	0.388
1592	8.52	6.84	D3ZW15	SEC24 homolog B, C	5	1.28	0.77	1.17	0.91	0.94	0.98173122	0.449
1593	8.51	11.81	Q505J8	Phenylalanine--tRNA ligase alpha subunit	5	1.04	0.95	0.95	0.94	0.94	0.95893172	0.0314
1594	8.5	8.96	A0A0G2K536	Paraplegin	5	0.74	0.58	1.33	0.72	0.96	1.03936328	0.0823
1595	8.5	5.73	D3ZWQ0	Proline-rich transmembrane protein 3	4	0.88	0.95	0.47	0.44	0.84	0.63551585	0.01686
1596	8.49	16.99	A0A0H2UHG4	Cyclin-dependent kinase 18	8	1.13	0.85	0.82	0.91	0.87	1.01515498	0.596
1597	8.48	24.02	P61314	60S ribosomal protein L15	4	0.86	1.37	1.82	1.27	1.41	1.09277774	0.039
1598	8.47	17.30	Q641X8	Eukaryotic translation initiation factor 3 subunit E	6	0.90	1.12	0.92	1.10	1.11	0.99663006	0.926
1599	8.47	26.51	B2RYX0	Naca protein	6	0.90	1.31	1.39	0.58	1.31	0.96881963	0.41
1600	8.46	14.64	D4A1H2	Phosphatidylinositol-specific phospholipase C, X domain-containing 3	6	0.82	1.32	1.14	0.95	0.95	1.02129525	0.597
1601	8.45	9.50	G3V796	Acetyl-Coenzyme A dehydrogenase, medium chain	4	0.95	1.10	1.09	0.97	1.02	0.92466328	0.0326

1602	8.44	5.95	F1LR60	Amyloid beta (A4) protein-binding, family A, member 1, isoform CRA_a	4	1.03	0.95	0.80	0.68	0.68	1.0552611	0.0081
1603	8.43	14.91	Q6PCT9	Proteasome (Prosome, macropain) 26S subunit, non-ATPase, 6	5	1.16	0.88	1.64	1.21	1.29	0.91446509	0.00601
1604	8.43	24.34	A0A0H2UHS	60S ribosomal protein L18	4	0.88	1.22	1.17	1.32	1.18	1.1455179	0.11
1605	8.42	20.78	Q5XIE6	3-hydroxyisobutyryl-CoA hydrolase, mitochondrial	9	0.69	1.08	0.85	1.06	0.90	0.93491039	0.119
1606	8.42	28.41	Q5PPN5	Tubulin polymerization-promoting protein family member 3	5	0.69	0.89	1.04	0.78	1.24	0.97218313	0.398
1607	8.4	19.94	D3ZDK7	Glycerol-3-phosphate phosphatase	5	1.09	0.80	0.91	0.99	0.84	0.98657457	0.792
1608	8.4	15.76	D4ABB2	Megalencephalic leukoencephalopathy with subcortical cysts 1	7	0.99	0.62	2.58	2.13	1.77	1.15829281	0.174
1609	8.4	26.05	P67874	Casein kinase II subunit	4	1.19	0.90	1.11	0.77	1.11	0.87843047	0.21
1610	8.38	45.73	Q5XFX0	Transgelin-2	14	3.31	3.05	2.88	1.27	1.80	1.07088236	0.154
1611	8.38	7.99	D3ZZ32	Prolyl endopeptidase-like	6	0.95	0.92	0.95	0.99	1.03	1.06865783	0.0855
1612	8.37	24.00	G3V8Z9	C	4	0.92	0.98	0.95	1.02	0.93	0.9938362	0.808
1613	8.37	25.76	D3ZW55	Inosine triphosphate pyrophosphatase	6	0.93	1.31	0.99	1.01	1.12	0.94154373	0.0685
1614	8.36	27.50	D3ZXS8	Huntingtin interacting protein 2 (Predicted),	4	0.99	1.20	1.19	0.79	1.04	1.01867935	0.638
1615	8.36	29.26	A0A0G2JXD	RCG63708	5	1.29	1.07	1.41	1.77	1.60	0.97427436	0.243
1616	8.33	13.13	B2GUZ6	Reticulon 4 interacting protein 1	5	0.97	1.07	1.04	1.04	0.97	0.97076904	0.0979
1617	8.33	18.66	Q5XIM0	BCS1 homolog, ubiquinol-cytochrome c reductase complex chaperone	6	1.09	1.02	1.05	0.95	0.93	0.99089219	0.627
1618	8.33	18.02	P20761	Ig gamma-2B chain C	5	0.33	0.75	1.17	0.66	0.65	0.93374467	0.493
1619	8.32	17.49	Q5BJY9	Keratin, type I cytoskeletal 18	9	0.13	0.30	0.29	0.30	0.74	0.92274249	0.466
1620	8.31	13.95	F1M9Y7	T-complex 11-like 1	5	0.34	0.95	0.74	1.01	0.45	1.0510272	0.0448
1621	8.31	22.16	G3V8F5	Mitochondrial import receptor subunit T	7	0.77	0.95	0.79	0.82	0.90	0.90689033	0.175
1622	8.31	14.73	Q6IMX3	Acetyl-Coenzyme A dehydrogenase, short chain, isoform CRA_a	4	0.90	0.76	0.89	0.85	1.02	1.02122446	0.759
1623	8.3	6.27	Q8CFG5	Voltage-dependent calcium channel subunit alpha-2/delta-3	4	0.93	0.62	1.09	0.72	1.25	1.06577286	0.0379
1624	8.3	13.66	Q58FK9	Kynurenine--oxoglutarate transaminase 3	6	0.89	1.45	0.45	0.75	0.46	0.93594782	0.0815
1625	8.3	21.12	O35263	Platelet-activating factor acetylhydrolase IB subunit gamma	5	0.95	0.95	0.91	0.95	0.90	0.89813237	0.00798
1626	8.3	29.37	P83868	Prostaglandin E synthase 3	6	1.36	0.19	1.27	0.55	1.39	0.98445693	0.927
1627	8.29	7.43	F1LSH6	Potassium/sodium hyperpolarization-activated cyclic nucleotide-gated channel 1	6	0.99	1.04	0.97	1.03	0.98	1.10419885	0.182
1628	8.29	22.22	B0BNN3	Carbonic anhydrase 1	6	1.39	2.01	1.42	2.25	2.96	0.88454044	0.0174
1629	8.28	19.01	D4AE56	Prostaglandin E synthase 2	4	0.61	1.02	0.69	0.52	0.70	1.03821123	0.314
1630	8.28	16.82	G3V762	Tissue specific transplantation antigen P35B (Predicted), isoform	4	1.12	1.36	1.31	1.06	1.11	0.95409181	0.258
1631	8.28	14.04	P04762	Catalase	5	0.47	0.84	0.98	0.95	1.03	1.03325762	0.627
1632	8.27	15.46	Q6QIX3	Zinc transporter 3	5	1.43	1.16	0.82	1.03	0.93	0.86094919	0.058
1633	8.26	25.76	B0BNJ1	L	6	1.01	1.04	1.05	1.04	0.98	0.95693975	0.403
1634	8.25	40.09	P62824	Ras-related protein Rab-3C	13	0.98	1.00	1.29	1.17	0.68	0.9815271	0.747
1635	8.25	16.02	Q4V8H8	EH domain-containing protein 2	10	1.31	2.83	2.61	1.43	2.54	0.97731802	0.403
1636	8.25	9.44	E9PT82	Striatin-3	6	1.10	1.04	0.98	0.95	1.09	0.85618829	0.351
1637	8.25	18.78	G3V644	NADH dehydrogenase (Ubiquinone) flavoprotein 3-like, isoform CRA_a	4	0.90	1.20	1.08	0.47	0.61	0.97813127	0.372
1638	8.24	13.30	G3V9U1	ArfGAP with GTPase domain, ankyrin repeat and PH domain 1	8	0.66	0.95	1.09	0.75	0.90	1.0055327	0.923
1639	8.23	13.81	Q62609	Noelin	4	1.04	1.21	1.03	1.05	1.03	1.00856216	0.755
1640	8.23	39.88	D4AB01	Histidine triad nucleotide binding protein 2 (Predicted), isoform CRA_a	5	0.79	0.81	0.95	0.90	1.11	1.01853814	0.649
1641	8.23	11.50	Q5U2U7	mRNA cap guanine-N7 methyltransferase	4	0.64	0.95	0.92	0.89	0.90	1.19085685	0.108
1642	8.21	25.52	O89035	Mitochondrial dicarboxylate carrier	5	1.09	1.04	0.99	0.95	0.95	0.98193539	0.653
1643	8.21	32.28	P62282	40S ribosomal protein S11	7	1.05	0.88	1.47	1.28	1.38	1.02782881	0.389
1644	8.21	29.12	D4A7N1	MIC	7	0.97	1.06	1.02	0.94	1.02	0.97002915	0.705

1645	8.2	10.07	P84903	Stromal interaction molecule 1	7	1.39	0.49	0.81	0.97	1.07	1.07177346	0.184
1646	8.2	22.49	Q5XII9	Mitochondrial fission regulator 1-like	4	1.01	1.00	1.01	0.95	1.08	1.02904067	0.47
1647	8.2	24.87	D4A565	NADH dehydrogenase (Ubiquinone) 1 beta subcomplex, 5 (Predicted), isoform CRA_b	5	1.01	1.43	1.01	1.17	0.82	0.85916076	0.0683
1648	8.19	13.61	F1LP80	Neurosecretory protein VGF	4	0.80	0.72	1.60	0.67	1.29	0.97083633	0.454
1649	8.19	24.10	P61023	Calcineurin B homologous protein 1	4	0.93	0.88	0.92	0.90	0.91	0.89751005	0.135
1650	8.18	50.56	Q78P75	Dynein light chain 2, cytoplasmic	5	0.95	0.93	1.01	1.01	0.90	1.05255819	0.273
1651	8.17	15.98	Q6IRF8	ATPase, H+ transporting, lysosomal accessory protein	5	0.83	0.99	0.90	0.95	0.90	0.99942416	0.984
1652	8.17	16.13	Q5M7A4	Ubiquitin-like modifier-activating enzyme 5	5	1.09	1.02	1.09	0.96	1.02	0.96066145	0.231
1653	8.17	40.48	Q566C7	Diphosphoinositol polyphosphate phosphohydrolase 1	5	0.85	0.30	0.90	0.52	0.59	0.90626194	0.429
1654	8.16	4.83	M0R9N8	Anillin, actin-binding protein-like 1	5	1.06	1.33	0.78	1.17	1.84	0.9945391	0.874
1655	8.14	20.67	A0A0G2K7B3	Heterogeneous nuclear ribonucleoprotein C	7	1.03	1.10	1.08	0.96	0.95	1.04891662	0.238
1656	8.14	6.13	A0A096MJG4	Cartilage acidic protein 1	4	1.19	1.69	1.27	0.59	1.07	1.06888008	0.314
1657	8.13	18.48	G3V9N0	Polyadenylate-binding protein	11	1.09	1.00	1.12	0.97	1.06	1.01213379	0.746
1658	8.13	31.36	G3V816	Nucleoside diphosphate kinase	5	0.94	1.09	1.00	0.99	0.91	0.98582263	0.803
1659	8.13	8.84	A0A0G2K0P8	CDW92 antigen, isoform CRA_b	4	1.28	0.65	1.34	1.29	1.29	0.99416692	0.93
1660	8.13	16.77	O88775	Embigin	6	0.88	0.47	1.43	1.20	1.20	0.99941377	0.987
1661	8.13	10.60	Q99MI5	Spermidine synthase	4	0.99	1.00	1.08	1.04	1.00	0.99813717	0.935
1662	8.13	10.79	Q63881	Potassium voltage-gated channel subfamily D member 2	6	0.93	1.01	0.94	1.01	0.98	1.03526492	0.474
1663	8.12	7.74	B1H264	TBC1 domain family, member 17	4	0.97	0.94	0.97	0.98	0.95	0.98179927	0.209
1664	8.12	26.34	P18297	Sepiapterin reductase	5	0.94	0.80	0.62	0.62	0.68	0.87599832	0.244
1665	8.1	6.74	F1LVZ9	HECT domain E3 ubiquitin protein ligase 3	4	1.53	0.83	0.52	0.82	1.26	0.99439435	0.788
1666	8.08	28.06	Q4V8E2	Proteasome (Prosome, macropain) 26S subunit, non-ATPase, 14	6	0.95	1.01	1.12	1.16	1.07	0.99178547	0.762
1667	8.08	15.12	F1LRE1	Glutathione reductase	4	1.46	0.42	1.29	1.45	0.43	1.03569557	0.0932
1668	8.07	9.72	Q9JMJ4	Pre-mRNA-processing factor 19	4	0.95	0.91	0.99	1.03	1.02	0.97420683	0.246
1669	8.07	7.17	E9PST5	Apoptotic chromatin condensation inducer 1	7	1.07	0.47	0.89	0.38	0.72	1.02612039	0.486
1670	8.06	9.54	Q5BJT7	Coiled-coil domain-containing protein 93	5	0.91	0.93	0.99	0.90	0.90	0.99588427	0.839
1671	8.06	6.53	F1LPJ6	Ubiquitin carboxyl-terminal hydrolase CYLD	5	1.02	1.49	1.36	0.88	1.22	1.00328397	0.909
1672	8.06	37.60	D4ADF5	Programmed cell death protein 5-like	4	1.57	1.57	1.34	0.76	1.72	0.98486644	0.556
1673	8.06	22.22	O35796	Complement component 1 Q subcomponent-binding protein, mitochondrial	5	0.86	1.06	0.74	0.25	0.78	0.92980494	0.201
1674	8.05	25.59	G3V6M3	Synaptotagmin II	18	0.93	0.98	1.05	0.96	0.95	0.91383145	0.0726
1675	8.05	17.01	P26453	Basigin	8	1.89	0.38	1.56	1.64	1.63	1.08673486	0.509
1676	8.05	19.19	F2Z3T7	Isochorismatase domain-containing protein 1	6	0.88	1.10	1.13	0.93	1.54	1.02072908	0.306
1677	8.04	25.33	D3ZWL6	Adenosylhomocysteinase	20	0.39	0.38	0.79	0.65	0.42	1.00779346	0.918
1678	8.04	6.42	Q5FVM6	Myotubularin-related protein 12	4	1.01	0.95	1.03	1.01	1.05	1.00393788	0.852
1679	8.04	24.21	P0C546	Mitochondrial coenzyme A transporter SLC25A42	5	0.79	0.69	0.77	0.69	0.83	0.93900191	0.146
1680	8.04	19.08	Q5RK25	Phosphomannomutase	5	0.83	0.78	0.61	0.72	0.80	1.01832636	0.588
1681	8.04	26.11	Q5XI64	Monoacylglycerol lipase ABHD6	5	1.03	1.49	1.00	1.33	0.94	1.04528764	0.0495
1682	8.04	18.99	D3ZGY2		4	0.51	0.65	0.58	0.31	0.63	0.90689033	0.0659
1683	8.03	12.45	Q5HZY0	UBX domain-containing protein 4	4	0.91	1.42	1.27	1.26	1.11	1.02661838	0.682
1684	8.02	8.39	G3V8V8	Leucine zipper putative tumor suppressor 3	4	0.44	0.67	0.64	0.25	0.52	0.89564567	0.00532
1685	8.01	44.62	Q5U318	Astrocytic phosphoprotein PEA-15	5	0.77	0.86	1.06	0.33	0.77	0.87175824	0.0862
1686	8	93.88	P11517	Hemoglobin subunit beta-2	49	0.61	1.80	1.34	1.42	2.15	1.15268635	0.568
1687	8	50.46	B0BNK1	RAB5C, member RAS oncogene family	18	1.00	0.99	0.96	0.96	0.98	1.00547694	0.871

1688	8	32.31	O35264	Platelet-activating factor acetylhydrolase IB subunit beta	7	0.99	1.28	1.13	0.84	1.15	0.96600329	0.491
1689	7.98	42.68	Q7TQ16	Cytochrome b-c1 complex subunit 8	7	1.18	1.32	0.86	1.19	0.95	0.88392753	0.197
1690	7.96	20.00	Q99ND8	Ppm1b protein	11	1.49	1.02	0.95	0.74	1.01	0.95528294	0.236
1691	7.96	8.57	Q6AXR4	Beta-hexosaminidase subunit beta	5	0.94	0.96	1.01	0.98	0.94	0.98186733	0.407
1692	7.96	13.61	G3V7G9	Eukaryotic translation initiation factor 3, subunit 6 interacting protein	4	0.95	1.02	0.97	0.96	0.98	0.98801168	0.795
1693	7.94	13.55	Q68FX8	Mitochondrial-processing peptidase subunit alpha	4	0.90	0.94	0.94	0.90	0.90	0.9879432	0.769
1694	7.94	5.33	Q9Z1X1	Extended synaptotagmin-1	4	1.24	1.37	1.24	1.49	1.32	1.01143247	0.554
1695	7.93	17.74	D4A9L2	RCG34610, isoform CRA_c	4	1.36	1.16	1.03	0.92	0.91	0.90877812	0.0471
1696	7.93	7.93	D3ZEV8	Sushi domain containing 2 (Predicted), isoform CRA_a	4	0.98	0.90	0.95	0.67	0.50	0.90375273	0.215
1697	7.92	33.77	Q4KLF8	Actin-related protein 2/3 complex subunit 5	7	1.21	1.17	1.13	1.22	1.54	1.04978945	0.321
1698	7.92	12.10	Q6AYB5	Signal recognition particle 54 kDa protein	4	0.96	0.96	0.97	0.97	0.90	1.03655739	0.112
1699	7.92	4.10	D3ZN27	DnaJ heat shock protein family (Hsp40) member	7	0.38	0.76	0.55	0.40	0.50	1.09885422	0.0968
1700	7.9	18.05	D3Z900	Mitochondrial amidoxime reducing component 2	8	1.15	0.72	1.87	1.27	1.38	0.97407178	0.554
1701	7.9	21.31	D3ZNQ6	Ubiquitin-conjugating enzyme E2M	4	1.21	1.32	1.27	1.36	1.49	1.00411882	0.858
1702	7.89	14.25	Q3MHS7	GDP-mannose 4, 6-dehydratase	4	1.01	1.00	1.03	0.98	0.99	0.96727633	0.446
1703	7.87	36.30	D4AAE9	CDGSH iron sulfur domain	5	0.96	0.99	0.98	1.03	0.97	0.97528786	0.202
1704	7.84	19.35	Q6AYR1	RCG52996, isoform CRA_a	6	0.72	0.75	1.03	0.34	0.97	0.99934311	0.977
1705	7.84	8.38	D3ZRC4	Patatin-like phospholipase domain-containing 8	5	1.06	1.06	0.96	0.98	1.01	1.05995278	0.00143
1706	7.83	79.61	P19804	Nucleoside diphosphate kinase B	17	0.76	1.31	1.63	1.04	1.67	1.12583859	0.214
1707	7.83	9.17	F1LMW3	Adhesion G-protein-coupled receptor G1	4	1.00	1.00	1.02	1.04	0.97	1.03096832	0.297
1708	7.82	10.87	Z4YNP1	CUGBP Elav-like family member 2	7	0.79	0.38	0.77	0.57	0.85	1.00484989	0.794
1709	7.82	15.22	A0A0G2JW01	Actin-binding LIM protein 1	4	0.97	0.95	0.99	0.96	0.95	1.02825636	0.53
1710	7.81	7.30	F1LR18	Rho GTPase-activating protein 39	6	1.06	1.06	0.97	1.13	1.09	1.02292474	0.472
1711	7.81	13.32	A0A0G2K8T(	Acid ceramidase	5	0.52	0.41	0.77	0.35	0.96	0.94921052	0.155
1712	7.81	5.81	P97603	Neogenin (Fragment)	4	0.98	1.07	1.01	0.99	0.95	0.91319825	0.0586
1713	7.81	21.71	D4A830	Pyrophosphatase (inorganic) 2	6	1.13	1.18	0.77	1.02	0.81	0.83335321	0.0943
1714	7.79	5.64	Z4YNH5	Formin-binding protein 1	4	0.96	0.98	0.95	1.02	1.04	1.02335025	0.209
1715	7.78	24.25	F1M3H8	Hypothetical protein L	6	0.64	1.13	1.06	1.14	0.90	1.03569557	0.61
1716	7.77	7.64	E9PT66	Splicing factor 3b, subunit 3	7	0.78	0.62	0.66	0.54	0.74	0.99896774	0.967
1717	7.77	16.62	Q6IRE4	Tumor susceptibility gene 101 protein	5	0.97	1.01	0.99	0.99	1.07	1.10649735	0.0478
1718	7.76	5.23	G3V9G1	Adenylate cyclase 5	5	0.87	0.97	0.98	1.16	0.90	1.05584642	0.508
1719	7.76	24.44	Q5U1Z2	Trafficking protein particle complex subunit 3	4	1.07	1.06	1.08	1.07	1.07	1.04434617	0.385
1720	7.74	29.72	B0BN65	Family with sequence similarity 49, member A	11	1.21	0.80	0.82	1.10	0.88	0.98241194	0.68
1721	7.74	19.69	F1LQZ0	Transmembrane protein 65	6	0.95	1.07	1.04	1.04	1.03	0.98527612	0.837
1722	7.73	35.92	P62744	AP-2 complex subunit	6	0.72	1.19	0.59	0.99	0.94	0.93174043	0.191
1723	7.72	38.96	B5DFN4	Prefoldin 5 (Predicted), isoform CRA_a	5	1.00	0.97	0.86	0.42	0.89	0.92210312	0.125
1724	7.71	16.86	Q5PPH0	Enolase-phosphatase E1	4	1.14	1.04	0.82	0.69	1.03	0.89751005	0.0004
1725	7.68	2.21	F1LZX5	HECT domain E3 ubiquitin protein ligase 4	6	0.90	0.93	0.95	0.92	0.90	0.96179411	0.059
1726	7.66	21.90	Q3B8Q2	Eukaryotic initiation factor 4A-III	8	0.89	1.00	0.82	1.02	0.83	0.98671134	0.395
1727	7.66	5.08	O35276	Neuropilin-2	4	1.36	1.01	0.90	1.25	0.80	1.00082519	0.971
1728	7.65	18.07	Q6P9V7	Proteasome (Prosome, macropain) activator subunit 1	5	0.84	0.72	1.07	0.66	0.78	1.03211233	0.472
1729	7.65	40.14	P61078	Ubiquitin-conjugating enzyme E2 D3	4	0.74	0.97	0.66	1.08	0.72	0.96179411	0.401
1730	7.64	4.95	M0R5H1	Enhancer trap locus 4	10	0.90	1.15	0.94	0.95	0.69	1.00193575	0.981
1731	7.62	12.02	Q5XIC0	Enoyl-CoA delta isomerase 2, mitochondrial	4	0.73	1.11	0.87	0.71	0.61	1.04659263	0.133
1732	7.62	4.87	D4A6P1	RCG40132, isoform CRA_a	4	0.95	0.92	0.91	0.98	0.90	0.86693756	0.021
1733	7.62	30.59	F1M5V2	GLI pathogenesis-related 2	4	1.24	0.61	3.31	1.29	1.75	1.01367839	0.899
1734	7.61	6.87	F1LPW0	Uncharacterized protein	3	1.07	1.03	1.17	0.89	1.02	0.96714225	0.796
1735	7.6	13.51	Q3MHT2	Cysteine desulfurase, mitochondrial	6	0.88	1.15	1.12	1.11	1.22	0.99754235	0.938
1736	7.6	9.70	D4A7R0	Signal recognition particle subunit SRP72	4	1.04	1.05	1.04	1.11	0.95	1.07624013	0.059

1737	7.6	3.98	D3ZBB7	Ubiquitin specific protease 32 (Predicted)	6	1.09	0.90	0.93	0.96	0.90	1.08447741	0.103
1738	7.59	9.07	G3V679	Transferrin receptor protein	6	0.98	0.85	0.67	0.90	0.70	0.98746396	0.618
1739	7.59	12.31	O35162	Heat shock 70 kDa protein	4	0.97	0.96	1.02	0.92	0.97	1.08222465	0.00927
1740	7.58	15.52	B5DEY8	Sorting nexin	6	0.74	1.13	0.94	0.90	0.88	0.94618881	0.0109
1741	7.58	14.96	Q9WTY2	GTP-binding protein REM	6	0.69	0.59	0.43	0.85	1.19	1.03986771	0.378
1742	7.58	27.98	D4ABM5	Mitochondrial ribosomal protein S34	6	0.72	0.97	0.77	0.79	0.89	0.91636865	0.0514
1743	7.57	30.89	G3V7W1	Programmed cell death protein 6	5	1.08	1.05	1.03	1.01	1.05	0.95972967	0.154
1744	7.55	19.27	Q6AY86	Vacuolar protein sorting-associated protein 26A	6	0.39	0.52	0.41	0.84	0.64	0.9789452	0.721
1745	7.55	10.58	D3ZA31	Myotubularin related protein 2 (Predicted), isoform CRA_b	6	1.11	0.97	1.04	1.03	1.13	0.97454452	0.477
1746	7.55	26.77	G3V6C3	Polysaccharide biosynthesis domain-containing 1	6	0.72	0.78	0.76	0.45	0.71	0.92851685	0.123
1747	7.54	4.44	O09178	AMP deaminase 3	4	1.42	1.36	1.80	1.32	1.80	1.06127608	0.294
1748	7.54	10.19	P17425	Hydroxymethylglutaryl-CoA synthase, cytoplasmic	5	1.16	0.33	0.73	1.02	0.77	0.99754927	0.968
1749	7.53	16.52	Q6IN22	Cathepsin B	5	1.08	0.71	1.11	1.00	1.03	0.98329758	0.561
1750	7.52	5.12	D3ZC15	Capping protein regulator and myosin 1 linker 2	5	1.17	1.31	0.57	0.91	1.20	1.11496622	0.17
1751	7.51	3.46	D3ZC56	Dystonin	21	1.04	0.99	0.99	1.00	0.98	1.04906204	0.0476
1752	7.51	6.69	F7F3I7	RCG62756, isoform CRA_a	4	1.07	0.97	1.07	1.03	1.04	0.99130437	0.169
1753	7.5	18.47	Q9WVK7	Hydroxyacyl-coenzyme A dehydrogenase,	5	0.56	0.65	1.08	0.98	0.99	1.05292305	0.0463
1754	7.5	7.90	A2VD12	Pre-B-cell leukemia transcription factor-interacting protein 1	4	0.46	0.77	0.68	0.71	1.05	1.0000601	0.999
1755	7.48	5.24	G3V7I8	RCG57812, isoform CRA_b	6	0.95	0.96	0.98	1.00	0.95	1.03648555	0.0616
1756	7.48	17.44	Q6AXS5	Plasminogen activator inhibitor 1 RNA-binding protein	5	0.91	1.33	1.41	0.90	1.37	0.99900929	0.988
1757	7.48	30.19	D3ZZ95	60S ribosomal protein L36	6	1.14	0.71	0.95	0.95	1.04	0.95356289	0.371
1758	7.47	20.45	P22734	Catechol	4	1.27	0.82	1.12	1.21	1.46	0.9815271	0.509
1759	7.47	7.05	G3V6U9	Histone-lysine N-methyltransferase	4	1.00	0.95	1.07	1.09	0.97	0.98064305	0.139
1760	7.47	7.99	A0A0G2JSR2	Synaptotagmin III, isoform CRA_a	4	0.97	0.60	0.54	0.63	0.82	0.95521672	0.358
1761	7.47	40.64	E9PTV0	Guanylate kinase 1	5	1.13	0.68	1.42	0.67	1.47	1.03497793	0.144
1762	7.45	16.50	B0BNG0	ER membrane protein complex subunit 2	4	1.13	0.79	1.04	0.89	0.97	0.95515052	0.41
1763	7.45	26.57	D3ZX87	Uncharacterized protein	4	1.28	1.46	1.91	1.14	1.72	0.99368466	0.909
1764	7.45	31.87	B2RZ78	Vacuolar protein sorting-associated protein 29	6	0.96	0.99	1.00	0.97	0.95	0.92658806	0.0369
1765	7.43	14.29	Q10758	Keratin, type II cytoskeletal	7	0.22	0.20	0.24	0.11	0.59	1.68646222	0.09309
1766	7.43	9.49	Q6PW52	GABA-A gamma2 long isoform	4	1.03	1.03	0.95	1.05	0.98	0.85500218	0.0301
1767	7.42	5.34	A0A0G2JY22	Disco-interacting protein 2 homolog B	6	1.00	1.26	1.08	1.60	1.20	0.99082351	0.576
1768	7.42	27.82	Q5XI72	Eukaryotic translation initiation factor 4H	4	1.87	1.91	1.50	1.47	2.29	0.95283611	0.0988
1769	7.42	6.52	D4A510	SWI/SNF-related, matrix-associated, actin-dependent regulator of chromatin, subfamily c, member 2	6	0.97	0.96	0.97	0.95	0.99	1.03857111	0.228
1770	7.41	15.86	D3ZUX5	MIC	4	1.02	1.04	1.04	0.93	1.19	0.92338231	0.104
1771	7.39	21.34	P29410	Adenylate kinase 2, mitochondrial	5	0.98	1.17	1.42	1.22	1.22	0.97184625	0.344
1772	7.38	18.83	Q64361	Latexin	5	1.16	1.77	3.70	2.36	3.02	1.06555126	0.183
1773	7.37	44.66	F1LNY3	Neural cell adhesion molecule 1	53	1.32	0.21	1.42	1.53	0.93	0.95462101	0.451
1774	7.36	17.27	Q6AY20	Cation-dependent mannose-6-phosphate receptor	5	1.08	0.44	1.33	0.75	1.05	0.95085681	0.35
1775	7.35	8.14	F7EZ89	TBC1 domain family, member 15	5	1.10	0.73	0.71	0.86	1.19	1.00954135	0.702
1776	7.35	10.72	F1MAG0	Deafness, autosomal dominant 5 (human)	4	0.99	0.97	0.97	0.96	0.94	0.95164804	0.0316
1777	7.34	29.89	O08697	ADP-ribosylation factor-like protein 2	4	0.73	0.19	0.48	0.44	0.61	1.08598186	0.0186
1778	7.33	29.41	Q62829	Serine/threonine-protein kinase PAK 3	21	0.99	1.00	0.92	0.95	0.91	1.02590703	0.399
1779	7.32	19.46	Q9R0I8	Phosphatidylinositol 5-phosphate 4-kinase type-2 alpha	8	1.15	0.54	0.91	1.39	1.27	1.02932602	0.509
1780	7.32	7.76	D3ZML2	Serine/threonine-protein kinase BRSK2	5	1.19	0.76	0.89	0.93	1.14	0.99536668	0.867
1781	7.31	10.25	Q5FVS6	Phosphatidylinositol 3-kinase regulatory subunit	6	0.77	0.88	0.89	0.87	0.89	1.00497527	0.86

1782	7.31	6.66	R9PXS2	Inositol polyphosphate 5-phosphatase	4	0.89	1.07	0.68	0.55	0.70	1.04246576	0.684
1783	7.3	59.04	M0RC65	Cofilin 2	10	0.88	0.54	0.79	0.91	1.06	1.11496622	0.059
1784	7.3	39.62	B5DEL8	NADH dehydrogenase (Ubiquinone) Fe-S protein 5	4	0.82	0.79	1.08	1.41	1.22	0.94927632	0.252
1785	7.29	19.49	Q9JHW0	Proteasome subunit beta type-7	6	1.26	1.08	1.80	1.47	1.66	1.00632757	0.487
1786	7.27	39.26	P62912	60S ribosomal protein L32	5	1.67	0.65	1.27	0.76	1.58	0.882703	0.0181
1787	7.27	7.05	D3ZPT0	Proline-rich 36	5	1.21	1.04	1.13	0.95	1.14	1.00842235	0.87
1788	7.27	23.02	A0A096MJG7	Nebulette (Fragment)	4	0.96	1.06	0.92	1.03	1.03	0.951714	0.368
1789	7.26	26.72	Q5XIG4		4	1.50	1.15	1.12	0.83	1.13	1.02065833	0.431
1790	7.25	6.05	D3ZA89	Similar to RIKEN cDNA A630047E20 (Predicted)	4	0.88	0.99	1.03	0.98	1.10	1.00001213	0.999
1791	7.25	6.08	G3V7T3	Pantothenate kinase 4	4	1.08	1.14	1.02	1.05	1.10	0.9554816	0.128
1792	7.25	5.91	A0A0G2K2T6	Interleukin enhancer-binding factor 3	4	1.21	1.08	1.11	1.06	1.07	1.07400447	0.0691
1793	7.25	14.29	D3Z9L0	Acylglycerol kinase	5	1.58	0.70	0.76	1.15	1.36	0.98732708	0.42
1794	7.23	16.36	Q4V8B7	Inactive hydroxysteroid dehydrogenase-like protein	4	0.79	1.12	0.57	0.84	0.84	0.95919763	0.333
1795	7.2	30.83	G3V8C4	Chloride intracellular channel protein	9	1.45	1.08	2.42	1.36	2.17	1.10803235	0.156
1796	7.19	5.98	D4A3H5	Chloride channel protein	4	0.96	1.45	1.22	0.53	0.79	0.9975147	0.976
1797	7.18	10.02	Q6P6T4	Echinoderm microtubule-associated protein-like 2	5	0.81	0.60	1.09	1.06	1.01	0.99240438	0.91
1798	7.16	24.59	Q3KRE2	Methyltransferase like 7A	4	1.28	0.51	1.26	1.18	1.31	0.99573932	0.954
1799	7.15	11.03	Q3SWT7	Nuclear receptor binding protein	4	0.99	0.89	1.04	0.94	0.99	1.15348561	0.205
1800	7.14	12.95	A0A5D0	Cd200 molecule	4	1.07	0.64	0.80	0.77	1.22	0.88086937	0.235
1801	7.12	18.97	P70580	Membrane-associated progesterone receptor component 1	6	0.82	0.99	1.04	0.66	0.64	0.94121747	0.279
1802	7.11	7.47	B4F763	VPS53 GARP complex subunit	4	1.00	0.90	0.91	0.99	0.97	1.55617035	0.01445
1803	7.11	5.56	D3ZK73	Cullin 4B	5	1.66	1.13	1.24	0.72	0.95	1.02647607	0.531
1804	7.11	5.97	Q80WF4	Transmembrane protein	5	0.69	0.86	1.36	1.58	0.74	1.11187916	0.269
1805	7.11	29.10	Q1RP74	RCG53953, isoform CRA_a	5	1.26	1.05	1.24	0.69	1.33	0.97191362	0.594
1806	7.1	8.01	P28841	Neuroendocrine convertase	4	0.90	1.03	0.91	0.85	1.02	1.00940141	0.913
1807	7.1	20.00	P63326	40S ribosomal protein S10	5	1.02	1.21	1.39	1.09	1.19	0.96560162	0.348
1808	7.09	6.00	Q8K4M9		4	1.71	2.21	1.54	1.32	1.51	1.02087059	0.256
1809	7.09	13.03	F1LMQ3	Proteasome 26S subunit, non-ATPase 8	5	0.99	1.32	1.24	1.50	1.00	1.01945635	0.786
1810	7.08	10.06	Q6AYB2	RCG53912, isoform CRA_a	4	0.96	1.14	1.00	1.06	0.97	0.77431903	0.141
1811	7.06	12.26	A0A0H2UHZ6	Poly(U)-binding-splicing factor PUF60	4	0.95	0.86	0.79	0.78	0.84	1.01038141	0.409
1812	7.05	9.98	A0A0G2K2S2	Solute carrier family 2, facilitated glucose transporter member 1	5	0.82	0.95	1.47	1.50	1.10	0.99945395	0.99
1813	7.04	2.90	Q7TSU1	Brefeldin A-inhibited guanine nucleotide-exchange protein 2	4	0.96	0.99	1.04	0.98	0.98	0.99593259	0.765
1814	7.02	17.18	Q6P7P5	Basic leucine zipper and W2 domain-containing protein 1	6	0.98	1.25	1.54	1.29	1.37	1.17853941	0.0555
1815	7.01	20.79	Q63377	Sodium/potassium-transporting ATPase subunit beta-3	5	1.10	0.16	1.10	1.22	1.01	1.02818509	0.315
1816	7.01	11.62	Q5XIC6	Proteasome (Prosome, macropain) 26S subunit, non-ATPase, 12	6	0.64	0.87	0.74	0.78	0.78	1.0603937	0.115
1817	7.01	6.55	Q642A9	VPS16 C	4	0.75	0.85	0.94	0.48	1.04	1.00599281	0.849
1818	7.01	13.71	Q68FX1	Mannose-6-phosphate isomerase	4	1.03	1.02	0.97	1.07	1.04	0.98336574	0.337
1819	7.01	9.28	P46413	Glutathione synthetase	4	0.59	0.80	0.87	0.48	0.73	0.92980494	0.113
1820	7	13.92	Q6P9U8	Eukaryotic translation initiation factor 3 subunit H	3	1.74	1.84	1.43	2.19	1.87	1.08522937	0.139
1821	7	20.69	P58200	Vesicle transport through interaction with t-SNAREs homolog 1B	4	0.95	0.95	0.96	0.92	0.97	0.91130128	0.0652
1822	6.98	6.69	O88664	Serine/threonine-protein kinase TA	5	1.01	0.99	0.92	0.96	0.89	1.06481293	0.226
1823	6.97	12.85	B1WBX6	Small ArfGAP2	4	1.00	0.94	0.93	0.72	0.88	0.97576119	0.291
1824	6.96	3.21	P54282	Voltage-dependent P/Q-type calcium channel subunit alpha-1A	6	1.20	1.19	1.17	1.15	1.14	1.03770761	0.0051
1825	6.96	18.22	Q9Z2Q7	Syntaxin-8	5	0.52	0.98	0.78	1.26	0.86	1.02058759	0.441
1826	6.95	5.02	A0A0G2K7K9	Calmodulin-regulated spectrin-associated protein family, member 2	6	0.89	1.05	1.03	1.06	1.16	0.92402257	0.00773
1827	6.95	12.24	D4AE02	Family with sequence similarity 98, member B	4	0.99	0.96	0.99	1.00	1.04	1.00786332	0.558
1828	6.94	6.74	Q9QZ81	Protein argonaute-2	4	1.77	0.72	0.52	1.03	0.55	1.01515498	0.353

1829	6.93	14.25	M0RAX4	Guanine nucleotide-binding protein subunit beta-5	7	0.80	0.49	0.63	0.70	0.74	0.96727633	0.414
1830	6.93	17.31	Q6IMX4	Phosphatidic acid phosphatase type 2B	4	1.25	1.13	1.27	1.07	0.93	1.03813927	0.205
1831	6.92	13.04	D3ZD73	DEAD-box helicase 6	5	0.82	0.90	0.87	0.97	0.80	0.99192297	0.752
1832	6.92	6.52	A0A0G2K2V5	Exocyst complex component 1	4	0.95	0.93	0.98	0.96	0.93	1.13288389	0.0554
1833	6.92	21.88	Q5XII0	Mammalian ependymin-related protein 1	4	0.88	1.02	0.99	0.91	0.98	1.01073164	0.796
1834	6.91	17.81	Q6AY23	Pyrroline-5-carboxylate reductase 2	4	0.68	0.66	0.85	0.80	1.03	0.98561765	0.716
1835	6.9	11.62	B0K017	ADP-ribosylhydrolase like 2	6	1.19	1.22	1.49	1.01	0.98	1.00349262	0.932
1836	6.89	6.37	B4F7F3	Armadillo repeat gene deleted in velo-cardio-facial syndrome	4	0.87	0.82	0.94	0.84	0.82	0.79278414	0.0539
1837	6.89	14.84	Q920G2	Na(+)/H(+) exchange regulatory cofactor NHE-	4	1.08	0.99	0.94	1.02	1.01	0.95000038	0.424
1838	6.88	4.99	Q496Z0	Elongator complex protein 2	4	0.87	0.84	0.96	0.95	0.93	0.96032857	0.193
1839	6.87	8.71	D4A5Q9	Glycine decarboxylase	6	1.05	1.04	1.03	1.07	0.93	1.03239854	0.142
1840	6.86	10.84	A0A0G2JT30	Methionine adenosyltransferase 2 subunit beta	4	0.97	0.83	0.99	0.69	1.08	0.98643781	0.761
1841	6.86	22.01	P04639	Apolipoprotein A-I	5	0.80	1.26	0.89	1.58	1.26	1.02164927	0.804
1842	6.86	28.46	D3ZLL8	Uncharacterized protein	4	0.45	0.76	0.82	0.81	1.28	0.9523079	0.238
1843	6.85	3.57	A0A0A0MY0	Ubiquitin carboxyl-terminal hydrolase 15	4	1.02	0.72	0.74	0.47	0.60	0.99544258	0.916
1844	6.84	12.26	Q9ERQ6	Chondroitin sulfate proteoglycan 5	5	1.51	1.10	1.53	1.06	1.01	1.02839892	0.701
1845	6.84	17.14	A0A0G2K8E7	Glycerophosphodiester phosphodiesterase domain-containing protein 1	4	0.76	0.47	0.89	0.64	0.74	1.03619821	0.163
1846	6.83	38.21	Q4V8F6	Pcbp2 protein	16	2.31	1.12	1.58	1.85	1.80	1.02968282	0.475
1847	6.83	43.28	Q5U316	Ras-related protein Rab-35	11	1.01	1.03	0.97	0.99	1.04	1.00010883	0.996
1848	6.83	6.19	F1MAB7	Diacylglycerol kinase	5	0.97	0.95	0.95	0.94	0.97	0.99123566	0.813
1849	6.83	7.06	A0A0G2K012	UBX domain protein 6	3	0.98	1.05	0.93	0.90	0.94	0.96399663	0.0411
1850	6.81	6.70	F1LQZ3	Kinesin-like protein	6	0.99	1.02	0.99	1.07	0.98	0.96928982	0.343
1851	6.8	36.78	Q9NQR8	NADH dehydrogenase [ubiquinone] 1 alpha subcomplex assembly factor 4	5	1.10	1.09	1.04	0.95	1.07	0.96841679	0.297
1852	6.8	21.37	Q5BK81	Prostaglandin reductase 2	4	1.37	0.25	1.53	1.06	1.67	1.11573932	0.0162
1853	6.8	29.17	G3V6B8	RCG38965, isoform CRA_a	5	0.73	1.26	1.01	0.50	0.96	0.87903956	0.0625
1854	6.79	6.51	A0A0G2JWS2	Nebulette	8	1.00	1.03	1.04	1.09	1.14	1.00336742	0.975
1855	6.78	4.77	D3ZJH2	SURP and G patch domain-containing 2	3	0.58	0.99	0.70	0.82	0.47	1.00905163	0.724
1856	6.78	13.56	A0A0G2JYW3	Clathrin light chain	5	1.00	1.60	1.27	0.65	1.19	0.9429806	0.269
1857	6.78	12.54	Q4G064	2-methoxy-6-polyprenyl-1,4-benzoquinol methylase, mitochondrial	4	0.95	1.00	0.92	0.96	0.93	0.95720511	0.128
1858	6.77	43.97	D3ZCZ9	NADH dehydrogenase [ubiquinone] iron-sulfur protein 6, mitochondrial	5	0.90	0.88	1.10	1.51	1.21	1.04008397	0.53
1859	6.77	23.19	Q5FVL2	ER membrane protein complex subunit 8	5	0.96	0.90	0.90	0.95	1.00	0.92210312	0.159
1860	6.76	4.19	F1M446	Uncharacterized protein	6	0.81	0.63	0.44	0.44	0.50	1.00207466	0.93
1861	6.75	74.56	P19944	60S acidic ribosomal protein P1	4	1.22	0.96	1.42	0.53	1.20	0.98472992	0.657
1862	6.74	4.27	F1M775	Diaphanous-related formin	5	0.98	1.05	0.93	1.06	1.04	1.07326029	0.0163
1863	6.74	14.95	B1WC67	RCG29001	4	0.97	1.06	1.11	1.01	1.05	1.00609742	0.717
1864	6.74	6.88	D2XV59	GTP-binding protein 1	3	0.92	0.86	0.82	0.85	0.90	0.97353179	0.496
1865	6.72	33.33	Q7M0E3	Destrin	6	0.70	0.99	1.39	0.69	1.29	1.11342162	0.0703
1866	6.72	13.18	M0RC57	Small ArfGAP 1	4	0.72	1.04	1.04	0.83	0.88	1.01248463	0.344
1867	6.72	3.78	D3ZMJ7	WNK lysine deficient protein kinase 2	6	1.04	1.04	1.00	0.95	1.03	0.98493471	0.582
1868	6.7	33.22	B2GUZ5	F-actin-capping protein subunit alpha-1	9	0.61	0.45	0.95	1.06	0.86	1.03813927	0.186
1869	6.7	4.57	D3ZAS2	Small G protein-signaling modulator 1	5	1.03	0.98	0.90	0.94	0.90	1.02569372	0.215
1870	6.69	7.58	F1M9C3	B-Raf proto-oncogene, serine/threonine kinase	4	0.95	1.03	0.76	1.16	0.90	0.97103823	0.285
1871	6.69	20.92	P84100	60S ribosomal protein L19	5	0.95	0.96	1.02	1.01	1.00	0.9893823	0.467
1872	6.69	22.48	D3Z8L7	Ras-related protein R-Ras	6	1.00	0.95	1.20	0.95	1.08	1.04681028	0.274
1873	6.68	8.56	F1LUR6	Glutamate receptor ionotropic, delta-1	4	0.97	0.98	0.86	1.02	0.85	1.09581177	0.0699
1874	6.68	19.10	B2GV73	Actin-related protein 2/3 complex subunit 3	8	1.77	1.75	1.45	1.66	2.58	0.95813444	0.247
1875	6.67	8.97	D3ZHG8	Golgi associated, gamma adaptin ear containing, ARF binding protein 3	6	0.97	1.03	0.55	0.92	1.09	1.02186173	0.251
1876	6.67	3.05	E9PT22	Uncharacterized protein	3	0.80	0.69	0.40	1.32	1.38	1.04138245	0.081

1877	6.66	29.05	D3Z8I7	Glutathione S-transferase, theta 3	7	0.99	1.03	1.08	1.05	1.11	1.06355895	0.582
1878	6.66	11.20	Q568Z6	IST1 homolog	3	1.15	0.79	1.41	1.47	1.12	0.98849118	0.646
1879	6.66	6.22	Q5RKG9	Eukaryotic translation initiation factor 4B	4	0.95	1.07	0.68	0.64	0.68	0.88392753	0.00045
1880	6.65	20.09	A0A0G2JSL2	RCG58353, isoform CRA_e	4	0.42	0.85	0.55	0.68	0.57	1.09126388	0.0131
1881	6.65	10.56	F8WFH6	Protein FAM131B	3	0.99	1.10	0.99	1.05	0.95	1.06090833	0.273
1882	6.64	27.63	Q6YDN8	BWK-1	5	2.09	1.71	1.50	1.21	0.76	1.06710342	0.104
1883	6.64	48.59	Q75Q41	Mitochondrial import receptor subunit T	6	1.09	1.05	0.97	0.90	0.91	0.84557229	0.0378
1884	6.63	22.55	D3ZCA0	Proline synthetase co-transcribed (Predicted)	5	0.79	1.36	1.10	0.87	0.82	1.33607408	0.046
1885	6.63	17.36	Q6AY17	Protein ABHD17B	3	0.90	0.90	0.84	0.95	0.90	1.03082541	0.436
1886	6.62	7.58	E9PSY8	Epidermal growth factor receptor pathway substrate 15	4	0.88	0.90	0.97	0.98	0.95	1.02186173	0.824
1887	6.61	12.80	Q68G41	Dodecenoyl-Coenzyme A delta isomerase (3,2 trans-enoyl-Coenzyme A isomerase)	3	0.89	0.90	0.86	1.14	0.87	0.94180481	0.232
1888	6.61	17.39	D3ZA85	Histone cell cycle regulation defective interacting protein 5 (Predicted), isoform	3	1.04	1.06	1.03	0.92	1.02	1.00144973	0.955
1889	6.6	54.64	P61227	Ras-related protein Rap-2b	14	1.19	0.56	0.63	1.08	0.90	0.9859593	0.747
1890	6.6	7.05	Q68FW7	Threonine--tRNA ligase, mitochondrial	6	1.04	1.24	0.64	1.07	1.07	0.95224189	0.0021
1891	6.6	21.83	Q6AYK6	Calcyclin-binding protein	6	1.63	0.75	1.51	0.85	1.56	1.0060486	0.806
1892	6.6	4.23	A0A0G2JZA1	Roundabout guidance receptor 2	6	0.77	1.11	0.84	0.91	1.03	0.98630107	0.359
1893	6.58	7.43	A0A140UHX(	Protein kinase C delta type	6	0.69	0.66	1.31	0.72	1.25	1.05995278	0.333
1894	6.58	15.70	Q63707	Dihydroorotate dehydrogenase (quinone), mitochondrial	4	1.07	0.97	0.95	0.99	0.89	1.03132569	0.21
1895	6.58	9.29	G3V8T7	RCG51933, isoform CRA_a	3	0.98	0.95	0.90	0.94	0.95	1.03168318	0.271
1896	6.58	32.26	M0R919	Prefoldin subunit 3	5	0.98	0.90	0.95	0.91	0.94	0.97265495	0.321
1897	6.58	8.98	Q3ZAU6	RCG49400, isoform CRA_a	4	0.90	0.95	0.94	0.98	0.97	0.9520439	0.13
1898	6.57	9.27	F1LRV6	GMP reductase	3	0.82	0.85	0.93	0.86	1.05	0.97002915	0.352
1899	6.56	29.12	A0JN30	Canopy 2 homolog (Zebrafish)	4	1.03	1.00	1.03	0.92	0.95	0.91319825	0.201
1900	6.55	4.39	P97924	Kalirin	9	0.72	0.69	0.90	0.83	1.13	1.06341152	0.0311
1901	6.55	20.74	Q64537	Calpain small subunit 1	5	0.94	1.10	1.06	0.96	0.95	1.04232125	0.495
1902	6.53	7.65	E9PTK4	Threonine synthase-like 1	5	0.79	0.83	0.72	0.85	0.91	1.16231411	0.135
1903	6.53	10.16	Q91V33	KH domain-containing, RNA-binding, signal transduction-associated	4	1.07	1.07	1.15	1.02	1.15	0.96674011	0.255
1904	6.53	3.81	M0RD40	SIK family kinase 3	4	0.81	0.48	0.87	0.93	0.78	0.9727898	0.341
1905	6.52	5.81	Q921A2	Proton myo-inositol cotransporter	3	1.00	0.95	0.56	0.82	0.79	0.96593633	0.215
1906	6.51	3.63	E9PTG1	SWI/SNF-related, matrix-associated, actin-dependent regulator of chromatin, subfamily a, member 2	5	0.93	1.02	1.11	0.97	1.04	1.06806541	0.0169
1907	6.5	3.03	F1M031	Neuron navigator 1	4	0.93	1.05	0.90	0.96	0.95	1.02164927	0.11
1908	6.49	15.00	D3ZPF2	Malonyl-CoA-acyl carrier protein transacylase	5	0.91	0.92	0.88	0.92	0.79	1.06289567	0.0694
1909	6.49	6.80	Q8CGU9	Tryptophan 5-hydroxylase 2	4	1.18	1.07	0.99	1.20	0.95	1.0238469	0.244
1910	6.49	5.36	A0A0G2JX77	WD repeat-containing protein 44	3	1.00	1.00	1.02	0.94	1.02	0.96059486	0.103
1911	6.48	13.03	F1M4H5	N	4	1.66	1.77	1.39	1.07	1.06	1.02172008	0.191
1912	6.47	56.11	P84083	ADP-ribosylation factor 5	24	1.16	1.11	1.11	0.92	1.16	0.9382212	0.233
1913	6.47	51.44	Q9WVB1	Ras-related protein Rab-6A	15	0.99	1.04	0.96	1.00	1.00	0.98541272	0.337
1914	6.47	6.19	F1LUY5	Tetratricopeptide repeat domain 7B	4	0.96	0.95	0.90	1.01	0.85	1.0065857	0.847
1915	6.47	21.36	Q9JHW5	Vesicle-associated membrane protein 7	4	0.85	0.77	0.77	0.70	1.01	0.97400427	0.463
1916	6.46	28.57	P60868	40S ribosomal protein S20	4	0.97	0.95	1.43	1.33	1.49	1.08522937	0.104
1917	6.46	5.93	D4A0H4	Cullin 2	5	1.09	1.03	1.07	1.07	1.13	0.99171673	0.754
1918	6.45	6.08	D3ZLD5	Golgin A3	7	1.06	1.00	0.99	0.96	0.99	1.03225543	0.0896
1919	6.45	5.60	D4A031	DEAD (Asp-Glu-Ala-Asp) box polypeptide 42 (Predicted)	4	1.14	0.86	1.05	1.16	1.46	1.03118273	0.13
1920	6.45	16.94	Q66HP8	Mitochondrial carnitine/acylcarnitine carrier protein	5	0.99	0.96	1.05	0.98	1.06	1.00158163	0.968
1921	6.45	31.72	Q6P2A5	GTP:AMP phosphotransferase AK3, mitochondrial	6	1.02	1.10	1.04	1.05	1.04	0.97043265	0.702
1922	6.45	5.50	D4AD89	Seizure-related 6 homolog-like	3	0.93	0.92	0.93	0.91	0.96	0.96246101	0.485

1923	6.43	5.32	Q704E8	ATP-binding cassette sub-family B member 7, mitochondrial	3	1.00	1.02	1.04	1.10	1.09	1.02448581	0.175
1924	6.42	7.89	D4A8L9	BTB domain-containing 17	3	0.56	0.87	0.56	0.64	0.63	0.94304597	0.193
1925	6.42	31.85	D3ZFA8	Uncharacterized protein	5	1.42	0.63	1.39	1.04	1.54	1.04073301	0.0564
1926	6.41	9.17	E9PSJ4	Sperm-associated antigen 9	8	0.87	0.95	0.95	1.09	0.75	1.15749022	0.0573
1927	6.41	9.43	Q8R424	STAM-binding protein	4	1.29	1.06	0.70	0.72	0.68	1.10956947	0.0663
1928	6.41	10.65	B5DEH4	UDP-N-acetylglucosamine pyrophosphorylase 1-like 1	4	0.90	0.83	1.26	0.91	0.81	1.0167042	0.691
1929	6.4	16.89	B5DEM5	60S ribosomal protein L14	5	1.28	0.63	1.82	1.37	2.00	1.03404574	0.327
1930	6.4	24.84	Q5XFW8	Protein SEC13 homolog	5	1.09	0.70	1.47	1.28	1.29	0.92916067	0.251
1931	6.4	3.59	D3Z9D0	Similar to KIAA1549	5	0.79	1.00	0.55	0.77	0.53	1.3764956	0.0415
1932	6.39	34.90	P13668	Stathmin	7	1.84	1.60	1.15	0.59	1.16	0.90689033	0.0257
1933	6.39	7.09	D3ZXQ2	Calmodulin-regulated spectrin-associated protein family, member 3	5	0.96	0.92	0.92	0.92	0.98	0.88086937	0.00521
1934	6.39	12.50	D3ZII1	Raftlin family member 2	4	0.90	1.05	0.99	0.97	0.98	0.96600329	0.49
1935	6.38	31.72	G3V6I9	60S ribosomal protein L26	5	1.07	0.93	1.09	0.76	1.16	0.98309313	0.699
1936	6.36	14.39	Q03626	Murinoglobulin-1	17	0.37	0.50	0.42	0.97	0.83	1.11573932	0.0769
1937	6.36	13.94	Q9Z311	Enoyl-[acyl-carrier-protein] reductase, mitochondrial	4	0.91	0.90	0.90	0.90	0.89	0.98541272	0.653
1938	6.35	5.32	Q3ZBA0	Tectonin beta-propeller repeat-containing protein 1	5	1.05	0.90	1.02	0.82	0.92	0.94004388	0.221
1939	6.32	33.90	P08753	Guanine nucleotide-binding protein G(k) subunit alpha	20	0.97	0.94	1.00	1.08	0.99	0.95727146	0.345
1940	6.32	9.81	Q5I0F0	Developmentally regulated GTP binding protein 1	3	1.02	0.98	0.94	1.03	0.93	1.01747969	0.752
1941	6.32	14.13	Q09167	Serine/arginine-rich splicing factor 5	4	1.18	1.43	1.84	1.22	1.37	0.97576119	0.48
1942	6.32	7.37	Q7TP91	Ab1-205	5	0.87	0.99	1.00	0.86	0.90	1.00884183	0.795
1943	6.3	11.56	C6ZII9	SIRT3L mitochondrial	3	1.06	1.04	1.03	1.08	1.03	0.92980494	0.00367
1944	6.3	4.88	P26431	Sodium/hydrogen exchanger 1	4	0.89	0.96	0.93	0.91	0.91	0.99353314	0.865
1945	6.29	4.07	D4A7M0	Plexin C1	5	1.12	0.99	1.01	0.94	0.90	1.0081428	0.836
1946	6.29	3.53	D4A051	Integrin alpha-7	3	1.12	1.05	1.11	1.09	1.09	0.93174043	0.0878
1947	6.29	8.53	D3ZBM3	Ferrochelatase	3	0.63	1.09	0.91	0.80	0.90	0.94782985	0.169
1948	6.28	6.38	Q63312	Pleckstrin homology-like domain family B member 1 (Fragment)	3	0.99	1.17	1.04	0.96	1.05	0.99013696	0.59
1949	6.28	7.51	D4A1Q9	Tubulin tyrosine ligase-like 12	3	1.41	1.18	1.22	1.33	0.79	0.93174043	0.0374
1950	6.27	7.05	F1MAH8	CAP-Gly domain-containing linker protein 1	11	0.94	1.03	0.91	0.98	0.99	1.05117291	0.0346
1951	6.27	23.48	D4ABI6	Ubiquitin carboxyl-terminal hydrolase	4	1.08	1.20	0.95	0.71	1.16	0.91509917	0.56
1952	6.26	14.49	Q80W83	Serine/threonine-protein phosphatase 2A 56 kDa regulatory subunit beta isoform	6	0.95	0.83	1.00	0.97	0.90	1.02115368	0.573
1953	6.26	7.38	D3ZJF9	Galactosidase, alpha	3	0.73	0.57	0.76	0.78	0.69	1.01755022	0.189
1954	6.26	4.28	F1M450	Slit homolog 1 protein	4	0.33	0.69	0.38	0.39	0.37	1.06503438	0.0796
1955	6.25	8.89	B0BMT9	Sqrdl protein	3	0.79	0.79	0.94	0.95	1.26	0.95336463	0.25
1956	6.24	6.98	P97680	Ras and Rab interactor 1	5	0.68	0.90	0.95	1.33	1.51	1.07400447	0.336
1957	6.24	5.35	A0A0G2JV26	Protein phosphatase 4, regulatory subunit 4	3	0.90	1.32	1.11	0.48	0.88	1.03993979	0.134
1958	6.24	3.75	A0A0G2K902	Sortilin-related VPS10 domain-containing receptor	3	1.18	1.18	1.10	1.08	1.14	1.0435502	0.21
1959	6.23	47.71	P62142	Serine/threonine-protein phosphatase PP1-beta catalytic subunit	13	0.99	1.02	0.99	1.00	1.02	0.98589096	0.8
1960	6.23	50.57	P11030	Acyl-CoA-binding protein	5	1.34	1.27	2.03	0.81	1.27	0.99868389	0.963
1961	6.22	5.39	F1LQI6	Zyg-11-related, cell cycle regulator	3	0.96	1.06	1.05	1.03	1.16	1.11961289	0.119
1962	6.22	21.66	D3ZUL1	Coiled-coil domain-containing 124	3	0.74	0.99	0.86	0.90	0.77	1.06068774	0.0595
1963	6.19	22.52	D3ZM21	Catechol-	5	0.94	0.97	0.86	0.95	0.85	0.94487802	0.0629
1964	6.18	37.50	Q5RKJ9	RAB10, member RAS oncogene family	11	0.81	1.42	1.06	1.67	1.09	1.0087719	0.728
1965	6.18	5.10	G3V6B0	Pyridoxal-dependent decarboxylase domain-containing protein 1	3	1.05	1.06	0.93	0.99	0.92	1.03950738	0.406
1966	6.18	10.79	Q9R064	Golgi reassembly-stacking protein 2	4	0.84	1.21	1.11	0.86	1.32	1.04949842	0.546
1967	6.17	6.37	F7ES73	Negative regulator of ubiquitin-like proteins 1	3	1.02	1.04	0.95	0.98	0.97	0.89813237	0.00753
1968	6.17	5.83	D3ZVH6	VPS41 H	3	1.07	0.88	1.32	1.03	1.08	1.00863207	0.648
1969	6.17	13.92	Q9ESB5	N-terminal EF-hand calcium-binding protein 1	5	0.95	1.28	0.68	0.42	0.66	1.01740917	0.161
1970	6.15	13.54	B2GUW3	Hdac11 protein	3	1.01	0.90	0.98	0.95	0.97	0.96259444	0.0379

1971	6.13	10.60	F1MA54	[Pyruvate dehydrogenase (acetyl-transferring)] kinase isozyme 1, mitochondrial	4	1.25	1.24	1.11	1.12	1.01	0.96969302	0.456
1972	6.13	8.39	D3ZP06	Integrin beta	3	1.29	0.85	0.66	0.73	1.06	0.98357025	0.667
1973	6.13	34.26	B0K020	CDGSH iron-sulfur domain-containing protein 1	5	1.02	0.95	1.00	1.04	1.05	0.99589807	0.888
1974	6.13	28.46	B0BMX3	S100 calcium binding protein A16 (Predicted), isoform CRA_a	3	1.33	1.80	4.06	1.09	1.89	1.12583859	0.125
1975	6.12	19.72	Q99P74	Ras-related protein Rab-27B	6	1.19	0.59	0.83	0.71	0.88	1.01726814	0.221
1976	6.12	15.46	P43278	Histone H1.0	4	0.99	1.02	0.95	0.73	0.64	1.00912158	0.788
1977	6.12	20.54	Q499N6	UBX domain-containing protein 1	3	1.05	1.14	1.01	1.07	1.01	0.95840012	0.0168
1978	6.12	20.77	Q6MG85	1-acyl-sn-glycerol-3-phosphate acyltransferase	5	0.90	0.86	0.53	0.93	0.64	0.84557229	0.0832
1979	6.11	11.06	Q6TUD3	LRRGT00111	4	1.05	1.04	1.02	0.98	1.09	1.01988042	0.509
1980	6.1	4.81	A0A0G2JYR1	Exocyst complex	4	1.01	1.09	1.01	1.00	1.07	1.05533424	0.453
1981	6.1	38.81	F2Z3T8	Guanine nucleotide-binding protein subunit gamma	3	1.94	2.19	3.56	2.56	2.23	1.08899702	0.332
1982	6.1	23.44	Q9JKW1	Mitochondrial import inner membrane translocase subunit Tim22	3	0.77	0.96	0.97	0.97	0.79	0.88209137	0.28
1983	6.09	8.00	B2GV57	Cysteinyl-tRNA synthetase 2, mitochondrial	3	1.19	1.07	1.47	0.98	1.14	1.01628145	0.404
1984	6.09	11.84	D4A4T0	STIP1 homology and U-Box containing protein 1, isoform CRA_b	3	1.05	0.62	0.77	0.81	1.02	0.9523079	0.335
1985	6.08	17.71	P62997	Transformer-2 protein homolog beta	3	0.83	0.86	0.84	0.48	0.58	0.82645032	0.0968
1986	6.08	10.80	B0BN30	Mitochondrial carrier 1	6	1.09	1.05	1.08	1.09	1.03	0.97664083	0.488
1987	6.07	44.56	Q5PQN0	Neurocalcin-delta	13	1.36	0.37	0.91	0.63	0.90	0.93355052	0.52
1988	6.07	16.00	A0A0G2JZD1	Protein phosphatase 2, regulatory subunit B',	6	0.86	0.92	0.84	0.84	0.82	1.0210829	0.467
1989	6.07	10.02	Q76GL9	Amino acid transporter	4	1.04	1.77	1.17	1.89	0.77	0.97353179	0.353
1990	6.07	4.83	Q5PQN1	Probable E3 ubiquitin-protein ligase HERC4	3	1.07	1.54	2.01	1.11	1.14	1.0238469	0.394
1991	6.07	7.45	D4A5W8	Phosphatidylglycerophosphate synthase 1	3	1.01	0.97	0.95	1.01	1.03	1.05723786	0.131
1992	6.07	6.65	Q4QQS7	Uridine monophosphate synthetase	3	1.02	1.06	0.95	0.90	0.95	0.95468719	0.0434
1993	6.07	25.00	Q6P9V1	Tetraspanin	16	1.61	0.80	1.79	0.75	1.09	1.07251662	0.529
1994	6.06	21.38	Q6AY09	Heterogeneous nuclear ribonucleoprotein H2	12	0.98	1.02	1.03	1.04	1.10	1.00130397	0.918
1995	6.06	4.75	B2RYP8	Gamma-tubulin complex component	4	1.53	1.54	1.11	0.67	0.69	0.97725028	0.399
1996	6.06	45.21	P04166	Cytochrome b5 type B	7	0.91	0.95	0.99	0.96	0.93	0.94907894	0.697
1997	6.06	3.53	D3ZXD2	Uncharacterized protein	5	0.87	0.95	0.96	1.04	1.01	1.07400447	0.197
1998	6.06	18.18	M0R7V3	MIC	3	0.94	0.88	0.79	1.10	0.80	0.91130128	0.0822
1999	6.06	35.46	Q80W89	NADH dehydrogenase [ubiquinone] 1 alpha subcomplex subunit 11	3	1.06	0.79	1.00	1.07	0.85	0.94180481	0.209
2000	6.05	9.03	Q7TQ77	Ac1288	4	0.95	0.93	0.95	0.94	0.96	1.06068774	0.04
2001	6.05	12.70	P23711	Heme oxygenase 2	5	0.97	0.86	0.56	0.95	0.99	1.00526091	0.713
2002	6.05	18.14	A0A0G2JU77	Eukaryotic translation initiation factor 3 subunit K	3	0.91	1.33	1.34	0.70	1.42	1.07698638	0.218
2003	6.05	6.29	Q9QX73	Rho guanine nucleotide exchange factor 9	3	0.85	0.98	0.65	1.00	0.63	1.06186473	0.153
2004	6.04	9.29	Q9JI85	Nucleobindin-2	5	1.00	1.06	1.08	0.93	1.05	0.9893823	0.878
2005	6.04	4.85	D3Z8B2	Nucleoporin 133	3	0.98	0.88	0.99	1.00	1.02	0.99533219	0.875
2006	6.04	11.55	Q64244	ADP-ribosyl cyclase/cyclic ADP-ribose hydrolase 1	3	0.90	0.71	3.10	1.57	1.49	1.04949842	0.405
2007	6.04	20.87	P42930	Heat shock protein beta-1	3	0.66	0.50	5.92	0.98	2.63	1.10956947	0.548
2008	6.03	5.69	Q62599	Metastasis-associated protein MTA1	4	1.11	0.99	0.98	1.03	1.09	1.08598186	0.188
2009	6.03	8.59	P60905	DnaJ homolog subfamily C member 5	4	1.01	1.50	1.24	1.41	1.09	1.03540845	0.459
2010	6.03	25.31	Q6AXR6	Glutathione S-transferase omega 1	5	0.78	1.38	0.69	1.20	1.67	1.00779346	0.841
2011	6.03	16.74	Q6PDU1	Serine/arginine-rich splicing factor 2	4	0.98	0.86	1.25	0.62	0.77	0.89254697	0.247
2012	6.03	31.82	P02625	Parvalbumin alpha	3	0.94	1.61	1.11	0.21	0.74	0.9741393	0.365
2013	6.03	15.49	P40241	CD9 antigen	10	1.08	0.21	2.05	1.61	2.07	1.52943628	0.01628
2014	6.02	12.16	D3ZHV7	Serine dehydratase-like	3	1.00	0.95	1.15	1.02	0.95	1.02115368	0.349
2015	6.02	28.57	D4ACJ1	40S ribosomal protein S24	6	0.80	1.03	1.12	0.69	0.87	1.03792342	0.0727
2016	6.02	10.77	P97519	Hydroxymethylglutaryl-CoA lyase, mitochondrial	3	1.26	0.62	1.01	0.78	0.60	0.96252772	0.13
2017	6.02	24.14	Q9WVJ4	Synaptojanin-2-binding protein	3	0.94	0.82	0.70	0.93	0.79	0.85381771	0.0797
2018	6.02	35.65	P30904	Macrophage migration inhibitory factor	9	0.63	0.58	0.60	0.60	0.72	0.98664295	0.863
2019	6.01	33.14	P62332	ADP-ribosylation factor 6	5	1.00	1.00	1.04	1.06	1.05	0.97407178	0.234

2020	6.01	7.56	E9PU34	RCG41110	4	0.95	0.94	0.89	0.96	0.89	0.99654026	0.913
2021	6.01	20.45	Q5PQL7	Integral membrane protein	4	1.28	0.90	1.04	0.51	0.95	0.95939711	0.395
2022	6	82.00	A0A0G2JVG	Pyruvate kinase	86	0.78	0.84	1.46	0.85	0.84	0.98554934	0.616
2023	6	42.79	A1L1J8	RAB5B, member RAS oncogene family	17	0.96	1.00	0.96	0.99	0.98	1.03261324	0.259
2024	6	41.53	Q63666	Vesicle-associated membrane protein 1	9	0.68	1.28	0.79	1.03	1.07	0.86393819	0.0981
2025	6	13.60	M0RCH5	Glucosamine-6-phosphate isomerase	4	1.15	1.08	1.08	1.04	1.04	0.97070175	0.489
2026	6	13.09	G3V834	Protein PRRC1	3	1.09	1.09	0.83	0.91	1.04	0.97292466	0.639
2027	6	25.20	A0A0G2K1L	Receptor expression-enhancing protein	4	1.98	1.84	0.94	1.04	1.49	0.94533659	0.436
2028	6	26.09	D4A4F9	RCG20461	3	1.05	1.03	0.94	0.97	1.02	0.91446509	0.123
2029	6	68.18	M0RBJ0	Guanine nucleotide-binding protein subunit gamma	6	1.20	0.32	0.78	1.07	1.04	1.00226222	0.988
2030	5.98	20.21	B0BN83	Armadillo repeat-containing protein 1	4	0.75	1.03	0.94	1.38	0.89	1.00912158	0.458
2031	5.96	11.67	F1LTR1	WD repeat domain 26	4	0.89	0.82	0.96	0.83	0.89	0.96620418	0.217
2032	5.96	3.77	P07897	Aggrecan core protein	7	0.86	0.82	1.63	1.37	1.72	1.02009252	0.414
2033	5.95	8.39	Q5FVL8	ATP-binding cassette subfamily B member 10	5	1.37	1.33	1.21	1.49	1.14	1.02775757	0.137
2034	5.94	36.15	D4A3V2	NADH dehydrogenase [ubiquinone] 1 alpha subcomplex subunit 6	5	1.05	1.60	1.04	1.37	1.00	1.06747331	0.331
2035	5.93	7.08	B5DEI0	Pcyox1l protein	3	0.83	1.03	0.50	0.77	1.15	1.05379921	0.273
2036	5.92	16.72	A0A0G2K5K	NmrA-like family domain-containing protein 1	5	0.98	1.05	0.94	0.98	0.98	1.03089686	0.216
2037	5.92	13.64	P06214	Delta-aminolevulinic acid dehydratase	3	1.02	1.09	1.03	0.94	0.90	0.97535546	0.278
2038	5.92	72.37	Q8K3X8	Heat shock factor-binding protein 1	5	1.28	1.45	1.05	0.24	0.95	0.74690673	0.143
2039	5.91	29.09	P04646	60S ribosomal protein L35a	4	1.15	1.01	1.69	1.39	1.64	1.03849913	0.408
2040	5.91	15.53	B0BN55	Uroporphyrinogen decarboxylase	4	0.79	0.34	1.09	0.50	0.29	1.0181852	0.569
2041	5.9	7.55	A0A0G2JTV	Non-muscle caldesmon	4	0.95	1.47	1.08	1.27	1.12	0.93633715	0.0583
2042	5.9	20.11	Q62639	GTP-binding protein Rheb	3	0.88	1.14	0.94	0.60	0.88	1.14313834	0.0756
2043	5.9	15.05	B1WC73	ADP-ribosylation factor-like 6 (Predicted), isoform	3	0.79	0.97	0.79	0.94	0.80	1.00475935	0.841
2044	5.89	3.45	D3ZDU2	Regulatory-associated protein of MT	4	0.97	0.95	1.01	1.04	0.95	0.98732708	0.606
2045	5.88	19.01	Q62625	Microtubule-associated proteins 1A/1B light chain	3	1.64	1.28	0.67	0.79	1.67	0.87903956	0.141
2046	5.87	16.67	F1LPQ6	Uncharacterized protein	8	1.56	1.45	1.36	0.84	1.26	0.94835558	0.0258
2047	5.87	4.83	Q5U2R9	Sec1 family domain-containing 2	3	0.96	1.08	0.90	0.91	1.02	1.11110873	0.161
2048	5.87	18.92	M0RCH6	Uncharacterized protein	6	1.84	2.58	2.75	1.24	2.81	0.99034287	0.727
2049	5.87	3.12	A0A0G2JZT1	NACHT and WD repeat domain-containing 2	4	0.88	0.84	0.77	0.74	0.82	1.15668818	0.00323
2050	5.87	10.25	P27615	Lysosome membrane protein 2	4	0.74	1.06	1.64	1.31	1.29	1.01628145	0.305
2051	5.86	4.06	D3ZEY4	Diacylglycerol kinase	3	0.98	1.04	0.87	0.82	0.95	1.12194348	0.0517
2052	5.85	26.49	D4A7L4	NADH dehydrogenase (Ubiquinone) 1 beta subcomplex, 11 (Predicted)	5	0.77	0.87	0.90	0.81	0.61	0.849685	0.114
2053	5.85	47.46	Q8R2H0	V-type proton ATPase subunit G	6	0.93	0.98	0.91	1.02	1.06	0.83566696	0.051
2054	5.84	41.38	Q63362	NADH dehydrogenase [ubiquinone] 1 alpha subcomplex subunit 5	4	0.86	1.34	1.04	1.25	1.00	1.0017066	0.973
2055	5.84	5.11	Q62667	Major vault protein	4	0.83	0.99	1.16	0.77	0.95	1.00765376	0.72
2056	5.84	22.73	B0BN18	Prefoldin subunit 2	3	1.27	0.79	0.89	0.73	0.95	0.79885192	0.148
2057	5.82	12.18	O54783	Choline/ethanolamine	3	0.90	1.08	0.85	0.91	0.78	0.98493471	0.63
2058	5.81	15.25	Q6AXT5	Ras-related protein Rab-21	4	1.00	0.95	0.90	0.96	0.90	0.96319513	0.0818
2059	5.81	44.07	D4ABS5	PITH domain-containing 1	4	1.82	1.85	1.57	1.09	1.42	0.91636865	0.151
2060	5.8	32.28	A0A140TAF0	RCG62531, isoform CRA_g	13	0.85	0.20	1.08	0.49	0.83	0.90940825	0.389
2061	5.8	13.10	M0RD55	Growth arrest-specific protein 7	4	1.32	1.05	0.52	0.58	0.60	1.00793318	0.808
2062	5.8	6.00	F1M7N7	Ribosomal protein S6	3	1.11	0.82	0.74	0.63	0.81	0.97522026	0.0889
2063	5.8	7.94	Q3KR59	Ubiquitin carboxyl-terminal hydrolase 10	4	1.01	1.16	1.42	0.81	0.88	1.01122217	0.76
2064	5.8	8.43	A0A0G2KAE	Similar to CG12125-PA (Predicted), isoform CRA_a	3	1.07	0.99	1.01	0.98	0.99	0.99709993	0.963
2065	5.78	10.36	D4A3E8	Mitochondrial ribosomal protein S27	4	0.42	0.80	0.66	0.86	0.63	1.32317144	0.0727
2066	5.78	7.87	D4AEK9	Coiled-coil domain-containing 6	3	0.92	1.00	0.97	0.86	0.94	1.00255404	0.895
2067	5.78	11.95	Q5XIP1	Protein pelota homolog	5	1.03	1.19	1.12	1.13	1.16	0.84147948	0.233
2068	5.78	20.51	B2RZD4	60S ribosomal protein L34	3	0.79	1.03	1.25	1.21	1.22	1.07922824	0.00556
2069	5.78	18.99	Q5XIE0	Acidic leucine-rich nuclear phosphoprotein 32 family member E	3	0.84	0.74	1.31	0.49	1.31	1.10343374	0.36

2070	5.77	9.63	A0A0G2K9I8	Uncharacterized protein	4	1.03	1.00	1.11	1.07	1.08	1.02996835	0.62
2071	5.76	14.29	D4A4P4	Flavin adenine dinucleotide synthetase 1	4	0.90	0.77	0.90	0.85	0.84	1.11573932	0.472
2072	5.75	7.89	Q62720	Zinc transporter 1	3	0.90	0.98	1.13	1.07	1.05	0.9815271	0.395
2073	5.75	2.47	O88902	Tyrosine-protein phosphatase non-receptor type 23 (Fragment)	4	0.94	1.05	0.97	1.04	1.00	0.98493471	0.483
2074	5.75	13.38	Q6AZ50	Ubiquitin-like-conjugating enzyme ATG3	4	0.63	0.54	0.74	0.70	0.46	1.04470817	0.247
2075	5.74	11.51	Q8K3P6	Calcium-binding mitochondrial carrier protein SCaMC-2	4	0.77	1.26	0.82	0.92	0.83	1.0059719	0.781
2076	5.74	5.77	A2VD14	ATP-binding cassette subfamily F member 2	3	1.09	1.16	1.00	0.98	1.00	1.03096832	0.734
2077	5.72	3.25	D4AAX6	Phosphoinositide phospholipase C	3	0.96	0.86	0.84	1.12	0.72	1.01283559	0.0911
2078	5.72	11.24	D3ZEG8	Putative uncharacterized protein RGD1309188_predicted	4	0.90	0.95	0.97	1.04	0.95	0.96006235	0.159
2079	5.71	22.90	Q78PB6	Nuclear distribution protein nudE-like 1	5	0.81	0.68	0.98	0.60	0.79	1.03483446	0.475
2080	5.7	8.99	M0RDX2	Amyloid-like protein 2	6	1.18	1.16	1.45	1.22	1.04	0.98186733	0.718
2081	5.7	4.43	A0A0G2JTB2	1,4-alpha-glucan-branching enzyme 1	3	2.03	1.60	1.50	1.79	1.34	0.95310033	0.225
2082	5.7	4.48	A0A0G2K9J0	Tetratricopeptide repeat, ankyrin repeat and coiled-coil-containing 2	8	0.97	0.95	1.00	1.03	1.05	1.0442014	0.103
2083	5.7	12.04	B5DF95	Family with sequence similarity 164, member A	4	1.84	2.05	1.38	1.89	1.56	0.98254814	0.32
2084	5.69	13.10	F8WFK6	Glutathione peroxidase	4	1.05	0.45	0.70	0.67	0.87	0.9418701	0.27
2085	5.68	10.22	A0A0G2JWK([illegible]	APC membrane recruitment protein 2	3	0.97	1.07	0.94	0.97	1.08	1.02740144	0.538
2086	5.68	5.88	Q66HG5	Transmembrane 9 superfamily member 2	3	1.47	0.64	1.03	1.15	0.95	0.96976023	0.408
2087	5.65	9.04	A0A0G2K6I4	Enabled homolog (Drosophila)	3	1.47	1.61	0.93	1.27	1.72	0.99075483	0.746
2088	5.63	25.37	P00173	Cytochrome b5	3	0.50	1.00	0.81	0.40	0.74	0.99006833	0.692
2089	5.63	2.57	A0A096P6L6	Teneurin-2	4	0.95	1.09	0.98	1.08	1.02	0.98520783	0.204
2090	5.63	22.86	M0RDY2	Family with sequence similarity 185, member A	3	1.31	1.87	1.41	1.43	1.31	1.31676692	0.0109
2091	5.62	8.09	O35049	Sphingomyelin phosphodiesterase 3	4	0.95	0.95	0.94	0.97	0.93	1.00558149	0.921
2092	5.61	13.47	A1L1L6	Mitochondrial Rho GTPase	7	1.17	0.50	1.20	0.72	1.25	1.01705662	0.618
2093	5.61	3.06	D3ZF86	ARFGEF family member 3	5	0.85	0.96	0.61	0.74	0.55	1.00125538	0.941
2094	5.61	2.41	F1LY70	Baculoviral IAP repeat-containing 6	5	0.79	1.00	0.89	0.95	0.82	1.20246925	0.0112
2095	5.6	34.41	O35353	Guanine nucleotide-binding protein subunit beta-4	10	1.53	0.59	1.47	1.56	1.56	0.88576752	0.23
2096	5.6	4.68	E2E1S0	Cyclin-dependent kinase-like 5	3	1.33	1.12	1.08	1.03	1.04	1.05000777	0.271
2097	5.6	11.64	Q6MG11	Alpha-tubulin N-acetyltransferase 1	5	0.55	0.72	0.41	0.57	0.38	0.87115419	0.397
2098	5.6	3.55	A0A140TAB([illegible]	Golgin subfamily A member	3	1.11	1.11	0.94	0.66	0.82	0.91193317	0.0411
2099	5.59	10.18	F1LNE8	Gamma-aminobutyric acid receptor subunit alpha-2	4	1.47	1.67	1.25	1.56	1.31	0.90062598	0.138
2100	5.58	3.56	F1M9N5	Ubiquitin conjugation factor E4 A	3	1.15	1.09	0.86	0.89	0.86	1.04188786	0.00757
2101	5.57	4.55	F1LS01	Protocadherin 9	3	1.01	1.02	0.96	1.11	0.99	1.03368743	0.203
2102	5.56	8.53	Q4KM62	Palmdelphin	4	0.97	0.95	0.96	0.79	0.93	1.05474921	0.0339
2103	5.55	24.76	P05545	Serine protease inhibitor	9	0.65	1.64	1.61	1.74	2.36	1.0508815	0.224
2104	5.55	13.97	P63144	Voltage-gated potassium channel subunit beta-1	4	1.05	0.88	0.94	1.05	0.99	1.03504967	0.202
2105	5.55	10.05	B2RZ37	Receptor expression-enhancing protein 5	3	0.88	1.02	1.14	1.10	1.15	1.05168307	0.0593
2106	5.55	21.59	P62718	60S ribosomal protein L18a	5	1.05	0.68	1.46	1.37	1.22	0.99424272	0.914
2107	5.54	27.27	B5DF46	Phosphomannomutase	6	1.09	1.03	1.06	0.95	1.34	1.05687152	0.104
2108	5.54	15.04	D3ZTX0	Transmembrane emp24 domain-containing protein 7	3	0.97	0.94	1.00	0.98	0.99	0.96486567	0.589
2109	5.53	10.09	G3V6L7	Potassium voltage-gated channel subfamily A member 4	5	0.93	1.01	0.94	0.92	0.95	1.02129525	0.279
2110	5.53	8.71	Q4QQS3	Protein	3	0.47	0.91	0.49	0.65	0.77	0.94324209	0.362
2111	5.52	5.01	P05508	NADH-ubiquinone oxidoreductase chain 4	3	0.78	1.45	0.89	0.83	0.96	1.03785148	0.182
2112	5.5	3.37	G3V7T6	Splicing factor 3b, subunit 1	5	0.95	0.99	0.56	0.70	0.65	1.05095435	0.16
2113	5.5	20.62	Q6PDW2	60S ribosomal protein L21	3	1.19	0.96	1.28	1.08	1.22	1.04015606	0.341
2114	5.5	4.08	G3V8L7	Integrin alpha M	4	1.77	0.92	2.83	0.97	1.43	0.98425224	0.47
2115	5.49	11.31	G3V912	Thioredoxin domain containing 13	3	1.13	0.99	1.02	1.00	1.00	0.9843887	0.614
2116	5.48	39.13	P35280	Ras-related protein Rab-8A	13	0.89	1.02	0.79	0.93	0.95	1.00248455	0.922

2117	5.48	12.84	O88506	STE20/SPS1-related proline-alanine-rich protein	6	0.59	0.43	0.67	0.77	0.84	1.01487356	0.63
2118	5.48	9.62	Q99M64	Phosphatidylinositol 4-kinase type 2-alpha	4	1.20	1.01	1.09	1.10	1.08	1.06466533	0.0699
2119	5.48	11.93	Q6JE36	Protein NDRG1	3	1.16	1.08	1.12	1.27	1.34	1.01024135	0.767
2120	5.48	7.77	Q5FWT1	Protein FAM98A	3	0.96	0.84	0.79	0.77	0.76	1.20748059	0.0725
2121	5.48	49.12	B5DFD8	SH3 domain-binding glutamic acid-rich-like	3	0.93	0.54	0.65	0.54	0.38	0.92530443	0.385
2122	5.46	32.86	P14841	Cystatin-C	4	1.08	1.38	1.82	1.43	1.28	1.13760523	0.219
2123	5.46	66.67	M0R809	Guanine nucleotide-binding protein subunit gamma	4	0.54	0.28	0.55	0.34	0.66	0.96828255	0.779
2124	5.45	9.33	P20673	Argininosuccinate lyase	3	0.96	1.02	1.08	1.02	0.96	0.90125046	0.0135
2125	5.45	8.02	Q4V8F9	Hydroxysteroid dehydrogenase-like protein	3	0.42	0.69	0.98	0.96	0.61	1.03655739	0.0365
2126	5.45	10.28	F7F350	Synapse associated protein 1, isoform CRA_a	3	0.93	1.07	0.89	0.82	0.90	1.02129525	0.14
2127	5.45	22.63	B4F7C7	Hebp1 protein	4	1.06	1.00	0.95	0.98	0.99	0.86753869	0.0776
2128	5.43	13.68	D4A520	PTC7 protein phosphatase homolog	3	0.29	0.41	0.53	0.48	0.66	1.04441856	0.567
2129	5.42	2.69	D3ZCG3	M	5	1.05	0.98	1.13	1.09	1.02	1.02975419	0.219
2130	5.42	6.09	B1WC02	CTP synthase	3	1.00	0.95	0.95	1.01	1.05	0.98186733	0.6
2131	5.42	14.12	A6JFQ6	Clavesin-1	4	1.01	1.02	0.95	1.09	1.02	0.99935004	0.977
2132	5.41	76.63	P62836	Ras-related protein Rap-1A	17	1.15	0.95	0.91	0.85	0.96	1.00793318	0.833
2133	5.41	7.54	Q5U355	Itfg1 protein	4	1.00	1.00	0.87	0.90	0.92	0.95303427	0.0574
2134	5.41	3.58	Q5U2Y1	General transcription factor II-I	3	0.86	1.07	1.17	1.20	1.03	0.98329758	0.626
2135	5.41	11.78	A0A0G2K2P6	HBS1-like protein	5	1.12	0.96	1.12	1.14	1.18	1.0276151	0.156
2136	5.41	6.34	F1LNB3	A-kinase-anchoring protein 10	3	0.92	0.79	0.90	0.94	0.95	1.09505247	2.5E-05
2137	5.39	6.12	M0RBF0	Scaffold attachment factor B1	4	0.81	0.50	0.95	0.69	0.79	0.9789452	0.629
2138	5.38	7.79	P97694	Cytohesin-1	3	1.39	1.34	0.91	1.42	0.99	0.96019545	0.538
2139	5.37	10.11	G3V648	1-acylglycerol-3-phosphate	3	0.97	0.95	1.01	0.96	0.96	1.01733865	0.781
2140	5.36	16.74	Q6AYQ8	Acylpyruvase FAHD1, mitochondrial	3	1.00	0.95	1.00	0.96	0.94	1.01550687	0.616
2141	5.35	3.35	F1LSM8	Dedicator of cytokinesis protein 9	5	1.06	0.76	0.64	1.09	0.66	1.03196926	0.0593
2142	5.35	18.18	Q9QYL8	Acyl-protein thioesterase 2	5	1.06	1.15	1.27	0.87	1.47	0.98139104	0.551
2143	5.35	13.75	Q3B8N9	Biphenyl hydrolase-like	4	0.56	1.13	0.90	0.83	0.82	0.86154616	0.0125
2144	5.34	10.18	D3ZXM4	Ecotropic viral integration site 5-like (Fragment)	4	1.09	0.95	1.12	0.81	0.97	1.06688154	0.132
2145	5.33	3.23	G3V849	Discs, large (Drosophila) homolog-associated protein 1, isoform CRA_a	3	0.55	1.82	1.82	1.77	1.56	1.24228828	0.0273
2146	5.33	9.21	Q4QQW3	Hydroxyacid-oxoacid transhydrogenase, mitochondrial	4	1.21	1.02	1.17	1.20	1.15	0.94514003	0.158
2147	5.33	45.19	P11762	Galectin-1	4	0.98	1.00	1.10	1.00	1.14	0.96935701	0.549
2148	5.33	15.06	Q6MGB5	Estradiol 17-beta-dehydrogenase 8	3	0.89	0.98	0.87	0.87	0.82	0.97657314	0.595
2149	5.33	10.66	F1M625	Ubiquitin carboxyl-terminal hydrolase 46	3	0.60	0.64	0.66	1.00	0.50	0.98883383	0.822
2150	5.32	8.32	Q5XI17	Threonyl-tRNA synthetase-like 2	4	1.25	1.98	1.17	1.57	1.18	1.05233934	0.412
2151	5.32	9.34	A0A140TA96	Glutamate receptor-interacting protein 1	4	1.00	0.98	0.52	0.51	0.54	1.1011416	0.0853
2152	5.32	7.63	F1LSC8	RIMS-binding protein 2	6	0.99	0.89	1.05	0.39	0.83	1.00772361	0.637
2153	5.32	7.31	F1LRI8	Methionine aminopeptidase	4	0.86	1.02	0.95	1.01	0.90	1.10880064	0.00827
2154	5.32	12.91	O08730	Glycogenin-1	5	0.97	1.09	0.97	1.05	1.05	0.93789609	0.271
2155	5.31	7.17	D4ADU2	Solute carrier family 7 member 11	3	1.09	1.06	1.11	1.19	1.13	1.01052149	0.366
2156	5.31	6.68	Q8R4A1	ER	3	1.00	0.98	0.97	0.96	0.87	0.93238649	0.136
2157	5.31	7.78	Q5U2Q7	Eukaryotic peptide chain release factor subunit 1	3	1.07	1.02	1.05	1.06	1.00	1.10266916	0.0439
2158	5.31	8.73	A0A0H2UHF	ATP-sensitive inward rectifier potassium channel	3	0.93	0.88	0.86	0.93	0.86	1.00191492	0.97
2159	5.3	10.44	E9PU13	Sorting nexin 4	4	1.04	1.02	0.95	1.01	0.98	1.02619151	0.126
2160	5.3	27.62	Q5XI86	Peptidyl-tRNA hydrolase 2	3	0.87	0.87	1.16	1.08	0.99	0.8735729	0.0242
2161	5.3	12.69	A0A0G2JUS0	Quaking	3	0.89	0.97	1.02	1.00	1.05	1.12818214	0.0437
2162	5.28	23.36	P68182	cAMP-dependent protein kinase catalytic subunit beta	7	0.96	1.32	0.91	0.76	0.61	0.99006833	0.469
2163	5.28	7.06	D3ZRN5	TR	3	0.97	1.21	0.94	1.00	0.99	1.05723786	0.207
2164	5.28	2.61	A0A0G2JVM	MIA family member 3, ER export factor	3	0.88	0.93	0.87	0.89	0.96	1.06813944	0.0962
2165	5.28	38.20	Q9WV97	Mitochondrial import inner membrane translocase subunit Tim9	4	1.02	1.05	1.01	0.96	0.96	0.87964908	0.0638
2166	5.27	8.57	Q5EBC0	Inter alpha-trypsin inhibitor, heavy chain 4	7	1.32	1.39	0.91	1.11	1.13	1.02918333	0.00561
2167	5.27	39.06	Q6PDV8	RCG31311	4	0.95	0.98	0.95	1.05	1.00	1.03111125	0.426
2168	5.26	50.00	P61751	ADP-ribosylation factor 4	17	1.45	1.32	1.32	1.32	1.12	0.94559873	0.108

2169	5.26	11.75	G3V6W2	Prolactin regulatory element-binding protein	4	1.01	0.86	0.91	0.95	0.97	0.9802353	0.809
2170	5.25	15.26	Q76IJ9	ELAV-like protein	5	1.53	1.50	1.25	0.82	0.95	0.95541538	0.207
2171	5.25	7.17	A0A0G2K266	E3 UFM1-protein ligase 1	3	0.99	0.91	1.03	0.86	0.96	1.08748839	0.0918
2172	5.25	23.38	D4A4L5	Iron-sulfur cluster assembly	3	0.81	1.01	1.14	0.52	0.84	0.99318199	0.883
2173	5.24	10.02	A0A0G2JXH2	Coiled-coil domain-containing protein 51	3	1.04	0.94	0.99	0.92	0.98	0.80832087	0.0742
2174	5.24	6.28	F1LT58	Importin subunit alpha	3	1.01	0.95	1.07	0.99	1.00	1.00947137	0.774
2175	5.24	23.48	P01355	Cholecystokinin	4	1.39	1.63	1.74	1.42	0.73	0.86035263	0.377
2176	5.23	6.52	A0A0G2K8Y7	Transmembrane and coiled-coil domain family 3	3	1.00	1.13	1.01	1.12	1.09	0.96976023	0.165
2177	5.23	14.29	Q498U4	SAP domain-containing ribonucleoprotein	3	0.93	0.93	0.97	0.89	0.90	1.0324701	0.187
2178	5.22	8.36	Q9WVI4	Guanylate cyclase soluble subunit alpha-2	4	0.91	1.00	0.95	0.96	0.90	1.01367839	0.61
2179	5.21	13.19	Q6P9U0	Serine (	5	1.75	0.95	1.27	1.37	0.97	0.99582214	0.909
2180	5.21	7.37	F1M3D2	Dystrophia myotonica, WD repeat-containing	3	1.04	1.02	0.99	0.99	0.95	1.17364818	0.0376
2181	5.21	22.52	G3V9P0	26S proteasome non-ATPase regulatory subunit 9	4	0.78	0.79	1.02	0.93	0.96	0.9893823	0.828
2182	5.2	7.81	Q6P685	Eukaryotic translation initiation factor 2 subunit	4	1.00	1.01	0.97	0.95	1.01	1.06732534	0.286
2183	5.2	7.12	D3ZN61	Leucine-rich repeat LGI family, member 3	3	1.01	0.87	0.88	1.04	0.87	0.95369509	0.203
2184	5.19	8.19	A4GW50	RCG29601	3	0.99	0.92	0.91	0.97	0.97	1.27632877	0.00588
2185	5.19	6.98	Q157S1	CREB-regulated transcription coactivator 1	3	1.63	1.49	1.17	0.43	1.58	0.93776608	0.163
2186	5.18	17.00	A0A0G2K526	Guanine nucleotide-binding protein G(olf) subunit alpha	9	1.21	1.22	0.86	1.06	0.24	0.95733781	0.169
2187	5.18	21.43	Q8VIL3	ZW10 interactor	4	0.77	1.04	0.79	0.77	0.69	0.93044966	0.207
2188	5.18	15.92	D4ADG2	IBA57 homolog, iron-sulfur cluster assembly	3	0.30	0.60	0.34	0.17	0.66	0.84089642	0.0278
2189	5.18	6.75	Q9JHZ9	Sodium-coupled neutral amino acid transporter 3	4	1.69	1.41	1.39	2.05	2.19	1.02030466	0.355
2190	5.16	2.37	D4A0B4	Ankyrin repeat domain 17	4	0.99	1.61	1.13	0.36	0.82	0.9879432	0.503
2191	5.16	4.41	Q6Q0N0	Calsyntenin-1	4	1.09	0.83	1.14	0.95	0.95	1.04993499	0.0409
2192	5.16	6.41	D3ZMS3	Matrilin 4	3	0.91	0.87	1.05	0.85	0.96	0.92851685	0.0837
2193	5.16	19.35	B2RYS8	NADH dehydrogenase [ubiquinone] 1 beta subcomplex subunit 8, mitochondrial	8	0.96	1.04	1.03	0.95	0.93	0.85027416	0.0195
2194	5.15	18.70	P17078	60S ribosomal protein L35	5	0.65	0.99	0.84	0.84	0.76	0.96720929	0.28
2195	5.14	9.87	F1M1L9	Copine 2	5	1.06	0.66	0.47	0.60	0.57	0.8520441	0.0435
2196	5.14	3.05	A0A0G2JYI0	LPS-responsive beige-like anchor protein	8	1.27	1.47	1.33	1.13	1.11	1.03082541	0.0289
2197	5.13	13.76	D3ZYS7	G3BP stress granule assembly factor 1	5	0.79	1.08	0.91	0.42	0.57	1.01094184	0.952
2198	5.13	8.37	F1LN46	Carnitine	4	1.00	0.95	0.96	0.98	1.05	1.00162328	0.959
2199	5.11	5.46	B5DFL9	SEC14 and spectrin domain-containing 1	5	0.65	0.62	0.60	0.52	0.41	1.05811762	0.0102
2200	5.1	66.67	Q04970	GTPase NRas	16	1.66	0.97	1.58	1.50	1.47	1.0153661	0.624
2201	5.1	9.01	Q4QR73	DnaJ (Hsp40) homolog, subfamily A, member 4	4	1.01	0.97	1.04	1.01	1.10	1.01164281	0.391
2202	5.1	13.82	P97576	GrpE protein homolog 1, mitochondrial	3	0.95	0.91	1.03	0.90	1.02	1.04492544	0.126
2203	5.09	22.50	Q498R7	UPF0587 protein C1orf123 homolog	3	1.38	0.93	0.89	1.10	0.91	1.01290579	0.819
2204	5.07	4.93	D4A554	Eukaryotic translation initiation factor 4 gamma, 3	6	1.46	1.19	0.92	0.79	0.95	1.03011114	0.36
2205	5.07	3.93	G3V7T8	Disks large-associated protein 3	3	0.97	0.99	1.06	1.01	1.04	1.11265012	0.115
2206	5.07	25.11	Q4QQV8	Charged multivesicular body protein 5	4	1.19	1.38	1.08	0.90	1.28	0.99907854	0.973
2207	5.06	3.78	G3V9P7	Huntingtin	8	0.98	0.96	0.90	0.90	0.92	1.00898169	0.81
2208	5.06	4.15	F1M4A0	Tight junction protein 1	5	1.54	1.27	2.13	1.33	1.28	1.05409142	0.23
2209	5.05	15.08	A0A0G2K302	Adaptor-related protein complex 3, sigma 2 subunit	3	1.19	0.85	0.82	0.41	0.95	1.07177346	0.257
2210	5.04	2.77	F1LRL4	Similar to TBC1 domain family, member 8; BUB2-like protein 1; vascular Rab-GAP/TBC-containing (Predicted), isoform CRA_a	3	1.03	1.01	0.98	1.03	0.93	1.02754388	0.25
2211	5.04	8.12	P27867	Sorbitol dehydrogenase	3	1.10	0.86	1.36	1.04	0.99	1.00751408	0.805
2212	5.04	9.53	Q6DGF2	Clathrin interactor 1	4	1.00	1.02	1.16	1.05	1.07	0.89688816	0.235
2213	5.04	19.05	D3ZCI5	Syntaxin-binding protein 6	4	1.26	1.45	1.26	1.36	1.18	0.98003148	0.687
2214	5.03	7.06	D3ZVR9	Phosphoglucomutase 5	3	0.98	0.93	1.08	1.06	0.90	0.98671134	0.709
2215	5.03	36.59	B0K010	Thioredoxin domain containing 17	4	1.02	1.11	1.08	0.90	0.95	0.94520555	0.392
2216	5.02	6.33	Q7TPJ5	Ac2-190	3	1.02	0.79	0.82	0.67	0.83	1.02292474	0.494

2217	5.02	41.33	D3ZUP5	BRICK1, SCAR/WAVE actin-nucleating complex subunit	4	0.87	1.00	0.88	0.74	1.56	0.97623474	0.728
2218	5	43.64	M0R7B4	RCG45259	15	0.90	1.37	2.01	1.39	1.61	1.01952701	0.713
2219	5	17.68	Q3MIE7	C	3	0.85	0.91	0.86	0.76	0.82	1.01557726	0.555
2220	4.99	10.50	Q02589	[Protein ADP-ribosylarginine] hydrolase	4	0.41	1.37	1.08	0.82	1.39	1.03211233	0.402
2221	4.99	24.29	G3V714	Neuroendocrine protein 7B2	3	0.88	0.82	0.82	0.74	0.79	0.93848136	0.397
2222	4.97	19.75	D4A720	RCG61762, isoform CRA_d	4	1.21	1.39	0.84	0.60	0.79	0.83798714	0.0617
2223	4.97	15.21	F1LQI1	Hydroxyacyl glutathione hydrolase	4	0.86	1.34	1.66	1.22	2.17	1.03268482	0.328
2224	4.97	19.53	P54900	Sodium channel subunit beta-2	4	1.66	0.69	1.22	1.34	1.39	0.98814866	0.632
2225	4.96	65.31	Q62669	Globin a1	16	1.39	1.64	2.15	3.05	1.89	1.20246925	0.581
2226	4.96	16.05	Q562C6	Leucine zipper transcription factor-like protein 1	3	1.12	1.09	1.17	0.88	1.13	0.95700608	0.226
2227	4.96	41.43	D4AD70	Uncharacterized protein	4	1.16	1.09	1.37	0.98	1.33	0.94927632	0.42
2228	4.95	11.72	Q5XIA5	Coenzyme A synthase	4	0.91	1.05	1.00	1.03	0.96	1.0179735	0.806
2229	4.95	7.33	B1WC50	EWS RNA-binding protein	3	1.12	1.03	1.09	1.15	1.05	0.87599832	0.0644
2230	4.94	14.68	A0A0G2K4B(	G protein-coupled receptor, class C, group 5, member B	5	1.25	1.26	1.39	1.07	1.17	1.02016323	0.152
2231	4.94	6.22	F1LS72	Ubiquitin-like modifier-activating enzyme 2	3	0.92	0.99	0.97	0.91	0.92	0.99178547	0.759
2232	4.94	8.74	B2RYC9	Glucosylceramidase	3	0.93	0.97	0.97	1.05	0.92	0.96339544	0.118
2233	4.94	18.59	D4ABK1	Synaptogyrin 3	4	0.86	1.09	0.80	0.99	0.81	0.882703	0.0135
2234	4.93	8.70	F1M110	Glycylpeptide N-tetradecanoyltransferase	5	0.88	0.96	0.86	1.02	0.95	0.92851685	0.0633
2235	4.93	10.03	A0A0G2K528	Syntaxin 16	3	0.88	0.88	0.82	0.90	0.83	0.93174043	0.056
2236	4.93	27.34	D3ZZ21	NADH dehydrogenase (Ubiquinone) 1 beta subcomplex, 6 (Predicted)	4	0.72	1.50	1.04	1.42	1.13	0.93581808	0.184
2237	4.92	11.74	Q52KK3	Solute carrier family 25 member 51	3	0.92	1.03	0.99	1.01	0.95	0.96006235	0.353
2238	4.91	6.24	Q641Z2	Tyrosine-protein phosphatase non-receptor	4	1.08	0.48	0.70	0.91	0.80	1.03756376	0.0661
2239	4.91	13.92	Q5U2Y0	WD repeat domain phosphoinositide-interacting protein 4	3	1.03	1.09	1.00	1.06	1.00	0.99376043	0.899
2240	4.91	9.63	D3ZAZ0	Eukaryotic translation initiation factor 3 subunit M	4	0.96	0.90	0.90	0.90	0.88	1.01360813	0.649
2241	4.9	6.70	F1LNF0	Myosin heavy chain 14	13	0.97	1.01	0.95	0.95	0.94	1.0387151	0.218
2242	4.9	6.58	A0A0G2K5B(	TBC1 domain family, member 24	3	0.88	0.93	0.92	0.98	0.92	1.0432609	0.067
2243	4.9	5.51	Q63616	Vacuolar protein sorting-associated protein 33B	3	0.86	0.87	0.90	0.84	0.90	1.02398885	0.323
2244	4.89	5.39	D3ZH14	Transmembrane and coiled-coil domain family 1	4	1.13	1.20	1.21	1.19	1.20	1.02278294	0.292
2245	4.88	7.20	D4ADS9	EFR3 homolog A	5	0.89	0.93	1.13	1.15	1.17	1.08147476	0.156
2246	4.88	6.52	D3ZE49	Trafficking protein particle complex 12	3	0.88	0.91	0.70	0.61	1.13	0.96801412	0.343
2247	4.88	6.58	Q66H15	Regulator of microtubule dynamics protein 3	3	0.97	0.93	0.94	0.90	0.91	1.02150764	0.663
2248	4.88	2.18	F1M842	Tumor protein p53-binding protein 1	3	1.00	0.92	1.03	1.02	0.93	1.03734803	0.0686
2249	4.87	5.17	A0A0G2K3D9	Transmembrane protein 132B (Fragment)	3	0.77	0.48	0.69	0.83	0.47	0.82645032	0.352
2250	4.87	9.97	P16975	SPARC	3	1.08	0.52	0.75	0.52	0.67	0.95409181	0.122
2251	4.87	24.19	D3ZT71	BCL2-like 13	6	1.15	1.16	1.04	1.09	1.07	1.00450866	0.895
2252	4.87	10.76	P16232	Corticosteroid 11-beta-dehydrogenase isozyme 1	3	0.64	1.00	0.84	1.10	0.45	1.06186473	0.513
2253	4.86	2.58	O55164	Multiple PDZ domain	4	0.86	0.72	0.76	0.67	0.74	1.00574879	0.773
2254	4.86	9.16	M0RDJ3	Interferon-induced, double-stranded RNA-activated protein kinase	3	0.95	1.28	0.83	1.11	0.95	1.04232125	0.0789
2255	4.86	16.73	Q5U1Z9	Metaxin 2	3	1.71	1.12	0.85	0.54	1.31	1.04797188	0.394
2256	4.85	8.21	A0A0U1RRZ4	Probable 2-oxoglutarate dehydrogenase E1 component DHKTD1, mitochondrial	4	0.96	0.53	1.33	1.05	1.16	1.0059719	0.7
2257	4.84	5.27	G3V829	Far upstream element-binding protein 3	3	0.90	0.95	0.99	0.98	0.98	0.98336574	0.689
2258	4.84	4.30	D3ZET9	Uncharacterized protein	3	0.92	0.56	0.93	1.08	1.07	1.14393097	0.0513
2259	4.84	23.40	A0A0G2K464	Secretory carrier-associated membrane protein	7	1.09	1.39	1.07	1.32	1.24	0.93238649	0.0122
2260	4.83	4.95	A0A0G2K127	Vascular cell adhesion protein 1	2	1.09	0.99	0.92	1.08	0.97	1.08372597	0.0277
2261	4.82	15.49	P24473	Glutathione S-transferase kappa 1	4	1.00	1.09	1.04	1.08	1.03	1.06378013	0.132
2262	4.82	7.77	F1LS53	G protein-activated inward rectifier potassium channel	3	1.18	0.82	1.08	1.42	1.05	1.16231411	0.0191
2263	4.81	3.69	Q6TUE6	LRRGT00098	4	0.98	1.10	1.20	0.98	1.16	0.99792964	0.942

2264	4.81	17.79	D3ZIM7	Family with sequence similarity 177, member A1	4	0.96	1.18	1.29	1.22	1.16	1.06555126	0.22
2265	4.81	22.02	P63031	Mitochondrial pyruvate carrier 1	4	0.79	1.39	1.16	1.66	0.76	0.9952425	0.96
2266	4.79	9.89	Q6P736	Polypyrimidine tract binding protein 1, isoform CRA_a	5	1.00	0.70	0.90	1.18	0.92	1.02939737	0.0413
2267	4.79	6.52	D3ZFB2	LUC7-like 3 pre-mRNA-splicing factor	4	0.59	0.76	0.79	0.59	0.41	0.99469767	0.902
2268	4.79	16.34	A1L108	Actin-related protein 2/3 complex subunit 5-like protein	2	1.16	1.25	1.04	0.95	1.05	1.08147476	0.251
2269	4.78	9.16	P38656	Lupus La protein homolog	3	0.99	1.02	0.96	1.01	0.95	1.0118532	0.734
2270	4.75	11.16	G3V8P5	Similar to cDNA sequence BC017158	3	1.06	0.98	1.03	0.99	0.94	0.94245785	0.00912
2271	4.75	3.45	G3V667	Integrin subunit alpha 6	4	0.95	0.94	1.03	0.95	1.00	1.09581177	0.112
2272	4.75	11.04	D3Z956	Retinaldehyde binding protein 1 (Predicted), isoform CRA_a	3	0.95	0.95	0.95	0.97	0.91	1.04782661	0.246
2273	4.74	22.47	A0A0G2JTX2	PRA1 family protein	3	0.73	0.90	0.82	0.70	0.47	1.01402977	0.711
2274	4.73	20.23	Q5FVH8	Regulator of G-protein signaling 7-binding protein	3	1.19	1.13	1.06	1.16	1.14	1.06414888	0.0868
2275	4.73	2.01	O35821	Myb-binding protein 1A	3	0.92	1.03	1.06	1.15	1.05	1.00559543	0.806
2276	4.73	13.38	Q5U2T9	Peptidylprolyl isomerase	5	1.12	1.02	0.85	0.97	0.95	1.0546761	0.253
2277	4.73	9.03	D4A9Q5	Carboxypeptidase M	2	0.96	0.76	1.17	0.95	1.27	1.1867368	0.144
2278	4.73	10.56	D4A746	GDP-mannose pyrophosphorylase B	2	0.93	2.11	2.51	2.03	2.07	1.05701804	0.0298
2279	4.72	18.64	D4A8T3	Coatomer protein complex, subunit zeta 1	2	1.10	1.15	1.12	1.04	1.01	0.89875513	0.177
2280	4.71	5.80	F1M943	Armadillo repeat-containing	3	0.97	1.03	1.00	0.87	0.79	1.06865783	0.271
2281	4.71	8.53	Q3B8N7	TSC22 domain family protein 4	3	0.77	0.72	0.87	0.61	0.76	0.97171154	0.611
2282	4.7	3.31	D3ZKG9	Clustered mitochondria protein homolog	4	0.98	0.75	0.57	0.74	0.44	1.02285384	0.108
2283	4.7	4.28	Q8R553	Calsyntenin-3	3	0.92	0.95	0.91	1.03	0.80	0.95046144	0.335
2284	4.69	6.39	A0A140TAH7	Actin-binding LIM protein 2	3	0.81	1.05	0.86	0.98	0.78	1.03211233	0.137
2285	4.69	12.37	D3ZU54	Density-regulated protein	3	1.14	1.17	1.17	1.04	1.06	0.94802697	0.128
2286	4.69	11.96	Q5M7T9	Threonine synthase-like 2	4	0.91	0.46	0.79	1.17	0.52	1.02455682	0.648
2287	4.68	2.74	Q810W7	Microtubule-associated serine/threonine-protein kinase 1	4	0.63	0.82	0.88	1.24	0.99	1.06858376	0.0496
2288	4.67	8.72	A0A0G2K4U4	Voltage-dependent L-type calcium channel subunit beta-1	5	1.16	1.10	1.05	1.09	1.06	1.06333781	0.00672
2289	4.67	6.78	Q07116	Sulfite oxidase,	3	1.05	0.59	0.98	1.14	1.22	0.98917659	0.812
2290	4.67	10.74	G3V7F1	G protein beta subunit-like, isoform CRA_a	3	0.89	0.85	0.91	0.98	0.95	1.01431095	0.333
2291	4.66	35.17	A0A0G2JU07	Ubiquitin-conjugating enzyme E2 variant 2	6	1.58	0.57	1.60	1.12	1.54	0.99226681	0.881
2292	4.66	9.18	Q64602	Kynurenine/alpha-aminoadipate aminotransferase, mitochondrial	3	0.78	0.95	1.58	1.43	1.89	1.06910237	0.223
2293	4.66	8.92	B0BNA7	Eukaryotic translation initiation factor 3 subunit I	3	0.94	1.10	1.17	1.07	1.02	1.01790294	0.404
2294	4.65	21.31	D4A0W1	ER membrane protein complex subunit 4	3	1.56	1.60	1.87	0.79	1.21	1.22603486	0.0506
2295	4.65	3.88	D3ZUF9	Pitrilysin metallepetidase 1 (Predicted)	2	1.08	1.39	1.15	1.06	0.80	1.07474917	0.21
2296	4.65	12.95	D3ZE72	Methionine aminopeptidase	3	0.90	0.96	0.83	0.86	0.94	0.98561765	0.735
2297	4.64	2.86	F1LST0	HEAT repeat-containing 5B	3	1.03	0.75	0.75	0.77	0.48	1.03096832	0.0122
2298	4.64	3.92	D3ZQM0	Splicing factor 3a, subunit 1	3	1.06	1.01	0.95	0.98	0.92	0.97725028	0.258
2299	4.63	4.38	A0A0G2K5Z1	Serine/threonine-protein kinase MRCK alpha	6	0.60	0.72	0.65	0.94	1.04	0.99732803	0.892
2300	4.63	3.82	D3ZE26	Similar to RIKEN cDNA 1110063G11 (Predicted)	2	1.11	1.07	1.16	1.03	1.07	0.87539133	0.00229
2301	4.63	12.73	Q5FWY5	AH receptor-interacting protein	3	0.90	0.98	0.96	0.95	0.90	1.08899702	0.00674
2302	4.63	1.70	D3ZGN7	Microtubule-associated monooxygenase, calponin and LIM domain-containing	3	0.81	0.74	0.47	0.54	0.82	0.98145906	0.26
2303	4.63	10.17	Q5XIK2	Thioredoxin-related transmembrane protein 2	5	0.56	0.86	0.81	0.71	0.54	0.97023088	0.36
2304	4.62	5.50	P06238	Alpha-2-macroglobulin	7	3.44	1.04	1.54	0.79	0.41	0.99709302	0.846
2305	4.62	3.83	P0C0R5	Phosphoinositide 3-kinase regulatory subunit 4	5	0.67	0.81	0.61	0.66	0.87	1.02519617	0.0185
2306	4.62	7.65	Q9Z1Z6	Integrin-linked kinase-associated serine/threonine phosphatase 2C	3	1.06	1.22	1.19	1.17	1.02	1.00982129	0.801
2307	4.61	3.76	D3ZMS1	Splicing factor 3b, subunit 2	4	0.48	0.62	0.42	0.46	0.60	1.06260102	0.143
2308	4.61	2.90	A0A0G2K5D5	Rho GTPase-activating protein 26	2	1.42	1.42	0.78	1.10	0.43	1.04884392	0.418

2309	4.61	9.52	Q4V7F2	Cysteine-rich with EGF-like domain protein 1	3	0.95	0.87	1.00	0.94	1.01	1.04362253	0.117
2310	4.6	12.73	F1LYA6	Uncharacterized protein	6	1.08	1.00	0.98	0.80	1.60	0.97393676	0.347
2311	4.6	4.48	O35867	Neurabin-1	5	1.08	1.17	1.27	0.98	1.11	0.98357025	0.0499
2312	4.6	8.30	Q63945	Protein SET	2	0.83	1.25	1.19	0.49	0.95	0.99867697	0.966
2313	4.6	9.36	Q66HG4	Aldose 1-epimerase	2	1.53	1.47	1.46	0.77	1.60	0.951714	0.227
2314	4.58	21.09	Q99MC0	Protein phosphatase 1 regulatory subunit 14A	3	0.98	0.56	1.45	2.11	1.82	0.92402257	0.208
2315	4.58	3.75	D3ZH59	Adhesion G protein-coupled receptor L3	4	0.92	0.30	0.64	0.71	0.67	1.00919153	0.845
2316	4.57	13.39	G3V6L9	Peptidylprolyl isomerase	3	0.95	0.99	0.96	0.87	0.91	1.01931503	0.736
2317	4.56	12.83	Q63450	Calcium/calmodulin-dependent protein kinase type 1	5	1.05	0.95	0.88	1.03	0.89	0.94618881	0.694
2318	4.56	53.62	A0A0G2JSR6	Guanine nucleotide-binding protein subunit gamma	4	0.89	0.54	0.73	0.93	0.95	0.92980494	0.336
2319	4.56	10.48	Q6NX65	Programmed cell death protein 10	2	0.94	1.02	0.97	0.94	1.00	1.01417035	0.538
2320	4.56	5.15	A0A0G2K4I2	La ribonucleoprotein domain family, member 4B	2	0.92	1.04	0.92	1.07	0.99	0.90877812	0.0104
2321	4.55	4.36	Q9JKC9	Synergin gamma	5	0.82	0.84	0.57	0.60	0.77	1.05139152	0.0788
2322	4.55	42.11	P0DN35	NADH dehydrogenase [ubiquinone] 1 beta subcomplex subunit 1	3	0.76	1.06	0.77	0.70	0.68	0.90187538	0.03
2323	4.54	2.93	F1LYG2	RCG54282	4	0.95	0.85	0.82	0.82	1.27	0.95138422	0.145
2324	4.54	26.32	D4ADD7	Glutaredoxin 5	3	0.97	0.95	0.95	0.88	0.97	0.95277007	0.0625
2325	4.54	9.54	F1M2C7	Immunoglobulin-like domain-containing receptor	4	1.34	1.06	1.02	0.76	1.20	0.92018765	0.124
2326	4.54	9.02	A0A0G2K3L8	WD repeat and FYVE domain-containing 1	4	0.97	0.79	1.06	1.02	1.07	1.01684515	0.287
2327	4.53	1.62	A0A0G2K2M9	Serine/arginine repetitive matrix 2	3	0.89	0.89	0.98	0.92	1.02	0.98787472	0.724
2328	4.52	3.27	Q9R1K8	RAS guanyl-releasing protein 1	2	0.98	0.97	0.95	0.90	0.92	0.99785355	0.897
2329	4.52	24.59	A0A0G2K226	NudC domain-containing 3 (Fragment)	3	1.50	1.21	1.42	1.64	1.06	1.00556755	0.921
2330	4.51	4.60	F1LPQ9	A-kinase anchor protein 2	2	1.09	1.06	1.09	0.94	0.99	1.09353546	0.352
2331	4.51	14.98	A0A0H2UHF6	PRA1 family protein	3	1.05	1.37	0.85	0.81	1.17	1.00082519	0.967
2332	4.51	9.02	Q9EPI6	NMDA receptor synaptonuclear signaling and neuronal migration	4	0.91	0.95	0.90	1.06	0.92	0.97097092	0.331
2333	4.5	13.78	B2RYS6	Phosphoribosyl transferase domain containing 1	4	1.08	1.07	0.93	0.92	1.18	0.9613942	0.068
2334	4.5	4.59	Q5XHZ8	Component of oligomeric golgi complex 3	3	0.90	0.91	0.86	0.90	0.92	1.00284595	0.922
2335	4.49	8.50	A0A0G2K9B2	Glycerophosphocholine phosphodiesterase GPCPD1	5	1.20	1.21	0.70	0.52	0.82	1.08975211	0.00391
2336	4.49	12.16	D3ZZR5	Small nuclear ribonucleoprotein polypeptide A'	2	1.01	1.05	1.03	0.93	1.03	1.04514275	0.266
2337	4.48	8.63	Q66H98	Serum deprivation-response protein	3	0.72	0.91	0.79	0.87	0.78	1.04167122	0.0547
2338	4.48	32.20	B5DES0	RCG54604	3	1.00	0.98	0.99	0.93	0.99	1.07773315	0.152
2339	4.47	12.92	Q6AY55	Dephospho-CoA kinase domain-containing protein	3	1.37	1.28	1.13	0.66	1.92	1.06311672	0.0777
2340	4.46	3.36	F1M2D4	Rho GTPase-activating protein 23	5	1.09	0.88	0.85	0.86	1.00	0.99500108	0.839
2341	4.46	6.75	Q6AY65	Arfaptin-2	2	1.10	0.99	0.98	1.02	1.05	1.03325762	0.383
2342	4.45	20.21	P81377	cAMP-dependent protein kinase type I-beta regulatory subunit	6	0.98	1.03	1.03	0.90	0.96	1.04398429	0.0131
2343	4.45	8.74	B1H2A6	FMR1 autosomal homolog 2	4	0.95	0.82	0.93	0.85	0.90	0.94756709	0.333
2344	4.45	6.63	G3V702	Smu-1 suppressor of mec-8 and unc-52 homolog (C. elegans)	4	0.97	0.95	0.86	0.90	0.89	1.01698613	0.679
2345	4.45	17.09	D3ZES2	Trafficking protein particle complex 6B	2	0.77	1.21	1.14	1.36	1.09	0.95019795	0.202
2346	4.44	2.88	D4A8Q2	Kinase non-catalytic C-lobe domain-containing 1	3	0.40	0.90	0.56	0.37	0.25	0.99519421	0.821
2347	4.43	19.12	Q9EST6	Acidic leucine-rich nuclear phosphoprotein 32 family member B	6	1.53	1.36	1.66	0.87	1.26	1.05511482	0.425
2348	4.43	2.93	A0A0G2JXN8		2	0.92	0.95	0.90	0.90	0.99	1.02612039	0.51
2349	4.43	4.59	A0A140UHX4	Protein kinase AMP-activated non-catalytic subunit gamma 2	2	1.24	1.06	0.95	1.14	1.24	0.98043915	0.201
2350	4.43	2.14	A0A140TAA3	Ral GTPase-activating protein subunit alpha-1	3	1.36	1.79	1.24	1.28	1.32	1.2683914	0.0225
2351	4.42	44.95	P85515	Alpha-centractin	18	1.22	1.03	0.87	1.05	0.82	0.99130437	0.909
2352	4.42	7.49	A0A0G2JYD8	Calpain-5	4	0.65	0.95	0.57	0.67	0.79	1.03011114	0.425

2353	4.42	3.77	D3ZWV8	T-cell lymphoma invasion and metastasis 1	4	1.06	1.03	1.08	1.03	1.09	1.0676213	0.168
2354	4.42	8.62	G3V9R0	LUC7-like	3	1.37	1.19	1.25	0.53	1.00	0.94973702	0.264
2355	4.42	16.15	Q6PCU4	Emd protein	3	1.14	0.92	1.05	0.95	0.95	1.02918333	0.68
2356	4.41	50.56	P63170	Dynein light chain 1, cytoplasmic	9	2.00	2.38	2.88	3.02	2.70	1.07624013	0.196
2357	4.41	5.16	P16970	ATP-binding cassette sub-family D member 3	2	0.99	0.93	1.03	0.98	1.00	0.97670853	0.333
2358	4.4	5.86	Q5HZY3	Ubiquitin carboxyl-terminal hydrolase	2	1.06	0.90	0.93	0.85	0.93	1.10343374	0.26
2359	4.4	3.21	F1LMZ4	Ribosome-releasing factor 2, mitochondrial	2	0.86	0.59	0.84	0.77	0.93	0.98418402	0.56
2360	4.4	8.58	D4A8G7	SNW domain-containing 1	3	1.17	1.03	0.95	1.25	0.88	1.01557726	0.694
2361	4.4	9.24	A0A0H2UHY	Aspartoacylase	2	1.16	1.43	1.57	1.74	1.84	0.98105097	0.645
2362	4.39	3.31	F1M0N1	Tyrosine-protein kinase	6	0.96	0.98	1.04	0.96	1.12	0.98780625	0.71
2363	4.39	8.45	Q08851	Syntaxin-5	2	1.02	0.94	1.05	0.92	1.00	1.09353546	0.0428
2364	4.39	7.76	B2RZ74	Small nuclear ribonucleoprotein U1 subunit 70	4	1.07	1.03	1.05	1.11	1.08	0.88699631	0.416
2365	4.38	4.88	D3ZD48	RAB11 family interacting protein 2 (Class I) (Predicted), isoform CRA_a	2	0.95	1.01	1.07	0.90	1.02	1.03490619	0.0665
2366	4.38	9.38	A0A0G2K167	Regulator of microtubule dynamics protein 1	2	1.11	0.80	0.94	0.95	0.95	1.00545604	0.916
2367	4.37	7.12	A0A096MKH	Similar to RIKEN cDNA 1110059P08	2	1.12	0.78	0.90	0.65	0.68	1.02129525	0.625
2368	4.37	3.13	F1LSW6	Uncharacterized protein	3	0.70	1.18	1.10	0.63	0.93	1.03061107	0.637
2369	4.37	7.47	Q66H12	Alpha-N-acetylgalactosaminidase	2	1.34	1.17	0.51	0.61	0.48	1.05124577	0.0186
2370	4.37	11.52	P63074	Eukaryotic translation initiation factor 4E	2	0.95	0.89	1.74	0.84	1.34	1.0369167	0.267
2371	4.37	9.68	Q9ESM2	Hyaluronan and proteoglycan link protein 2	3	0.86	0.89	1.25	0.95	1.01	1.10803235	0.0288
2372	4.36	15.23	P35284	Ras-related protein Rab-12	5	1.12	0.97	1.09	1.08	1.06	1.04340554	0.184
2373	4.36	4.60	P11661	NADH-ubiquinone oxidoreductase chain 5	3	0.82	1.11	0.92	1.03	0.82	0.98227576	0.724
2374	4.36	15.04	D3ZD11	Signal peptidase complex subunit 2	3	0.64	0.50	0.58	0.42	0.18	1.00397267	0.913
2375	4.35	10.00	D4A7H9	CHMP family, member 7 (Predicted), isoform CRA_a	4	0.91	0.87	0.95	0.83	0.95	1.04217677	0.161
2376	4.35	21.31	M0R6L9	Ferritin	3	0.79	0.74	0.74	0.84	0.74	1.07922824	0.0267
2377	4.35	10.39	A0A0G2K5Y1	RCG20801, isoform CRA_c	3	0.77	0.75	0.74	0.77	0.73	0.95647555	0.599
2378	4.34	34.25	Q63941	Ras-related protein Rab-3B	18	1.18	0.54	0.72	0.95	0.43	0.97955608	0.694
2379	4.34	22.22	Q9Z269	Vesicle-associated membrane protein-	4	0.98	0.97	0.91	0.88	0.91	0.8913105	0.043
2380	4.33	25.00	Q62658	Peptidyl-prolyl cis-trans isomerase FKBP1A	4	1.29	1.89	1.12	1.37	1.61	1.01755022	0.774
2381	4.33	7.58	A0A0G2K7G	ArfGAP with FG repeats 2	3	0.83	0.86	0.90	0.81	0.98	1.01621101	0.806
2382	4.32	8.70	A0A0A0MXY	Casein kinase I isoform gamma-3	3	0.96	0.86	0.86	0.86	0.93	1.00520517	0.939
2383	4.32	22.05	Q6P9U3	C	3	1.13	1.10	1.20	1.16	1.09	1.00235253	0.935
2384	4.32	9.17	Q6AYC4	Macrophage-capping	5	1.03	1.51	5.45	2.61	0.88	1.17690674	0.3
2385	4.32	10.55	D3ZRM5	RAB23, member RAS oncogene family	2	0.92	0.90	1.03	0.94	0.97	1.04986222	0.571
2386	4.32	1.98	D4A4K4	Uncharacterized protein	6	0.92	0.90	0.97	0.90	0.98	1.01910309	0.219
2387	4.32	31.18	B2RZ27	SH3 domain binding glutamic acid-rich protein-	4	1.33	1.47	2.00	1.11	2.33	1.06917648	0.578
2388	4.31	7.73	Q62785	28 kDa heat- and acid-stable phosphoprotein	2	0.87	1.34	1.16	0.76	1.20	1.10649735	0.176
2389	4.31	15.75	A0A0G2K648	WD repeat-containing protein 61	3	0.82	1.47	0.70	0.70	1.19	0.86333956	0.0241
2390	4.31	12.68	Q56A26		2	1.13	0.74	0.31	0.53	0.50	0.82817066	0.0395
2391	4.3	6.97	Q6AY57	WD repeat domain phosphoinositide-interacting protein 2	2	0.96	0.88	0.94	0.95	0.90	0.94809268	0.0624
2392	4.29	3.23	D4A1Y0	SCY1-like pseudokinase 2	2	1.03	0.93	0.95	1.06	0.94	1.07997656	0.0618
2393	4.29	3.33	D3ZJ86	Sodium/hydrogen exchanger	2	1.04	1.04	1.03	1.05	0.99	0.95270403	0.317
2394	4.29	9.07	A0A0G2KAI8	Developmentally-regulated GTP-binding protein 2	2	0.98	1.06	1.05	0.94	0.95	1.04666517	0.442
2395	4.29	8.92	Q5RJY4	Dehydrogenase/reductase SDR family member 7B	4	1.04	1.05	0.95	1.04	0.93	1.01017133	0.768
2396	4.29	17.33	Q9JIX3	Bis(5'-adenosyl)-triphosphatase	2	0.89	1.04	0.95	0.93	0.96	1.02740144	0.406
2397	4.28	2.38	Q6MG08	ATP-binding cassette sub-family F member 1	2	1.46	0.94	1.11	0.89	1.09	1.98618499	0.02065
2398	4.27	5.84	A0A0G2JZC6	Rho guanine nucleotide exchange factor 11	7	0.97	0.97	0.92	1.02	0.99	1.02335025	0.173
2399	4.27	11.96	B0BNM9	Glycolipid transfer protein	2	1.20	1.14	1.25	1.17	1.29	1.07624013	0.0887
2400	4.27	5.48	Q4V7D9	Sphingomyelin phosphodiesterase, acid-like 3B	2	1.24	1.12	1.10	1.37	1.38	0.93309767	0.0614

2401	4.27	22.51	D3ZT16	MAP6 domain-containing 1	3	1.05	1.00	0.74	1.19	1.20	0.95627668	0.64
2402	4.26	2.31	F1LRS2	Dedicator of cytokinesis 7	4	0.88	0.77	0.75	0.52	0.75	1.01487356	0.364
2403	4.26	15.16	B2RYA8	DnaJ heat shock protein family (Hsp40) member B2	3	1.09	1.01	1.07	1.24	1.04	0.95687342	0.554
2404	4.25	2.60	F1LQB2	Structural maintenance of chromosomes protein	3	0.98	0.98	0.98	0.98	0.93	1.07773315	0.0536
2405	4.25	2.87	Q7TP05	Complement C2	3	0.67	0.82	0.89	0.90	0.72	1.05995278	0.011
2406	4.25	4.98	A0A096MIV6	Williams-Beuren syndrome chromosome region 17 (Fragment)	2	0.97	1.03	0.90	0.93	0.90	1.00737442	0.816
2407	4.25	3.12	D4AAR7	Coiled-coil domain-containing 136	2	1.07	1.29	0.65	0.37	0.64	0.95481954	0.046
2408	4.25	16.31	Q923M1	Mitochondrial peptide methionine sulfoxide reductase	3	1.00	1.10	0.90	0.97	1.01	1.0016094	0.947
2409	4.24	45.51	P63088	Serine/threonine-protein phosphatase PP1-gamma catalytic subunit	13	0.96	0.93	0.98	1.00	0.96	0.99295483	0.899
2410	4.24	4.35	A0A0G2KA25	Transmembrane 9 superfamily member	4	0.96	1.09	1.08	1.07	1.00	0.96727633	0.357
2411	4.23	4.94	A0A0G2K7Q9	Kinesin-like protein KIF21B	7	0.95	0.92	1.02	0.44	0.86	1.01578847	0.362
2412	4.23	5.94	A0A0G2K0L0	3'-phosphoadenosine 5'-phosphosulfate synthase 1	2	0.63	0.04	0.32	0.51	0.69	1.07400447	0.241
2413	4.23	23.86	D3ZAF6	ATP synthase subunit f, mitochondrial	3	1.11	1.63	0.94	1.07	1.32	0.97792789	0.701
2414	4.22	4.90	G3V886	ATP-dependent zinc metalloprotease YME1L1-like	2	1.63	5.15	1.77	3.66	4.06	1.08522937	0.0705
2415	4.22	5.58	F1LWG2	Protein arginine methyltransferase 8	2	1.05	0.97	1.17	1.12	1.15	1.06680759	0.036
2416	4.22	16.85	P62914	60S ribosomal protein L11	4	1.04	1.19	1.33	1.15	1.25	1.01339738	0.574
2417	4.22	3.32	Q924I2	Mitogen-activated protein kinase kinase kinase kinase	2	0.89	0.98	0.81	0.96	0.90	1.02697424	0.125
2418	4.22	11.86	Q7TS56	Carbonyl reductase family member 4	2	0.87	0.99	0.89	1.03	0.90	0.99907854	0.979
2419	4.21	36.53	M0R7K1	Protein lin-7 homolog A	11	1.16	1.16	1.15	0.99	0.93	0.98835416	0.552
2420	4.21	3.76	D3ZTB4	VPS11 C	3	1.01	1.03	1.11	1.07	1.03	1.00338829	0.8
2421	4.19	11.34	F7ESM5	Nitrilase 1, isoform CRA_a	2	1.12	0.88	1.00	0.24	0.71	1.02278294	0.175
2422	4.18	18.05	Q6J4I0	Protein phosphatase 1 regulatory subunit 1B	2	0.92	0.86	0.75	0.88	0.94	0.9208257	0.0504
2423	4.18	13.25	Q62876	Synaptogyrin-1	3	0.97	1.12	0.99	1.08	0.97	0.89564567	0.0577
2424	4.18	6.19	D3ZXK4	Abhydrolase domain-containing 11	2	1.09	0.95	0.93	1.01	1.02	0.91891288	0.168
2425	4.17	8.95	A0A0H2UI02	Serine/threonine kinase 11 (Predicted), isoform CRA_a	3	0.94	1.08	1.01	1.04	1.07	1.11806185	0.0125
2426	4.17	15.92	B2GV08	Adaptor-related protein complex 1, sigma 2 subunit	3	0.75	0.28	0.37	0.46	0.30	0.8882268	0.0864
2427	4.16	15.35	D3ZQL1	ER membrane protein complex subunit 7	2	1.03	1.13	1.00	1.12	1.07	1.00225527	0.967
2428	4.16	8.70	G3V907	Peroxisomal biogenesis factor 19-like	2	1.01	1.06	1.07	0.96	1.10	1.04550503	0.189
2429	4.16	17.59	Q6PEC1	Tubulin-specific chaperone	2	0.90	1.10	0.98	0.93	1.02	1.15588671	0.012
2430	4.15	9.06	P22831	Synaptoporin	2	1.41	0.28	0.95	0.81	1.04	0.92466328	0.474
2431	4.15	14.80	Q5HZA9	Transmembrane protein	3	1.17	1.32	1.13	1.07	0.95	1.01417035	0.656
2432	4.15	9.18	A0A0G2JVG4	Peroxisomal trans-2-enoyl-CoA reductase	2	0.90	0.54	0.38	0.72	0.53	1.1011416	0.343
2433	4.14	3.16	D3ZWA5	Kinesin-associated protein 3	3	1.08	1.14	1.01	1.17	1.07	1.09050773	0.122
2434	4.14	3.15	D3ZN95	Host cell factor C1	4	1.34	1.05	1.05	1.11	1.18	1.00961133	0.751
2435	4.14	33.64	D3Z8Q5	RCG25747	2	0.73	0.86	0.14	0.61	0.82	0.91066983	0.303
2436	4.13	6.46	D4A5X1	Stromal interaction molecule 2	3	0.82	0.70	0.49	0.86	0.53	1.01769129	0.482
2437	4.13	6.11	D3ZGM1	Pentatricopeptide repeat domain 3	3	0.07	0.42	0.22	0.42	0.31	0.95382731	0.0134
2438	4.13	5.67	F1LUW9	Tubulin gamma chain	2	1.00	1.19	1.15	0.95	0.74	0.96372939	0.167
2439	4.13	17.65	A0A0G2K8L9	Mitochondrial ribosomal protein S28	2	1.75	3.31	0.30	1.51	0.97	0.94507452	0.565
2440	4.13	10.44	Q6P7Q1	BRCA1-A complex subunit BRE	3	1.61	0.76	1.11	0.88	1.29	1.00793318	0.583
2441	4.13	17.37	Q5PQN7	Protein LZIC	2	1.11	1.04	1.00	0.59	1.03	0.90814842	0.139
2442	4.12	4.51	Q01992	Mitochondrial intermediate peptidase	2	0.58	1.09	0.83	0.91	0.86	1.00926148	0.796
2443	4.12	3.61	D4A2D7	Importin 4	2	0.86	0.64	0.42	0.69	0.91	1.00716496	0.809
2444	4.12	9.85	Q9WVS2	Probable tRNA N6-adenosine	2	1.12	1.02	1.09	1.09	1.11	0.90125046	0.00266
2445	4.12	17.32	M0RDU0	Coagulation factor VIII-associated 1	4	1.03	1.60	1.17	1.14	1.01	0.95191193	0.599
2446	4.1	13.00	Q9JI92	Syntenin-1	4	2.94	2.65	2.51	2.40	2.13	1.0242728	0.504
2447	4.1	15.42	P63164	Small nuclear ribonucleoprotein-associated protein N	3	0.94	1.00	0.92	0.91	0.95	0.88699631	0.00883

2448	4.1	11.08	F1LR33	Phospholipid phosphatase-related protein type 2	3	1.14	0.55	1.16	1.11	1.05	0.97009639	0.6
2449	4.1	13.04	Q5XIE1	Protein THEM6	3	0.93	0.90	0.93	0.92	0.95	1.01248463	0.902
2450	4.09	7.42	D4A8L5	Adenylyltransferase and sulfurtransferase M	3	0.89	0.89	0.77	0.91	0.97	1.01213379	0.719
2451	4.09	6.62	A0A140TAJ1	N-chimaerin	3	0.96	0.90	0.94	1.04	0.79	1.06437018	0.251
2452	4.09	12.62	Q5XID1	Anamorsin	2	1.27	1.85	2.01	0.75	1.66	1.06895417	0.178
2453	4.08	48.19	P62749	Hippocalcin-like protein 1	13	1.36	1.20	0.69	0.63	0.35	0.92916067	0.374
2454	4.08	3.20	D3ZF54	Anoctamin	2	0.68	0.88	0.83	0.83	0.92	1.05416449	0.184
2455	4.08	5.51	P23978	Sodium- and chloride-dependent GABA transporter 1	2	0.83	1.27	0.76	0.32	0.46	0.91891288	0.00052
2456	4.08	2.75	A0A0G2K6N2	Adhesion G protein-coupled receptor B2	2	1.29	1.09	1.04	1.50	1.05	0.94940793	0.0677
2457	4.08	43.10	Q9JJW3	Up-regulated during skeletal muscle growth protein 5	4	0.89	0.90	0.92	1.21	0.84	0.94986869	0.616
2458	4.07	10.07	Q6P0K8	Junction plakoglobin	8	0.90	0.95	0.90	0.79	0.97	0.96935701	0.356
2459	4.07	12.27	A0A0G2K9B1	Serine protease inhibitor	4	1.49	1.00	1.21	0.69	0.45	1.01860874	0.53
2460	4.07	6.86	F1LQ09	Atlastin GTPase 2	4	1.36	0.55	0.91	1.34	1.10	0.96935701	0.307
2461	4.07	3.82	D3ZXJ5	Elongation factor-like GTPase 1	3	0.74	0.79	0.84	0.78	0.73	1.03332924	0.232
2462	4.07	6.29	Q925D6	Mitogen-activated protein kinase kinase 6	2	1.06	1.06	0.95	1.10	1.07	0.97332937	0.451
2463	4.07	7.81	B5DFK7	Abhydrolase domain-containing 17C	2	0.79	0.73	0.43	0.50	0.57	1.10037861	0.141
2464	4.07	10.28	Q5RJK8	Acyl-CoA-binding domain-containing protein 6	2	1.04	0.99	1.15	0.96	0.98	1.08598186	0.109
2465	4.07	25.00	D3ZSP1	Uncharacterized protein	3	0.98	1.10	0.84	0.64	0.78	0.97359928	0.397
2466	4.06	22.00	G3V7K6	Single-stranded DNA-binding protein,	3	1.09	0.79	0.81	0.82	0.92	0.97616708	0.474
2467	4.06	4.20	D3ZQP6	Sema domain, immunoglobulin domain (Ig), and GPI membrane anchor, (Semaphorin) 7A	2	0.77	0.89	0.90	0.86	0.90	0.98329758	0.698
2468	4.06	15.08	M0R907	RCG60635, isoform CRA_b	2	0.86	0.86	0.74	0.72	0.72	0.95515052	0.319
2469	4.06	4.16	B4F7C1	G protein-coupled receptor 37-like 1, isoform CRA_a	2	1.45	0.26	1.67	1.49	1.33	1.0132569	0.815
2470	4.06	7.55	Q52KJ9	Thioredoxin domain containing 1	2	0.96	0.95	0.96	0.93	0.93	0.85263489	0.0812
2471	4.05	22.51	P53812	Phosphatidylinositol transfer protein beta isoform	5	0.69	0.64	0.98	0.25	0.81	1.00779346	0.789
2472	4.05	4.46	G3V6G7	Polyribonucleotide nucleotidyltransferase 1	4	0.87	0.90	1.00	0.96	0.86	1.0685097	0.0994
2473	4.05	5.00	Q5FWS6	Kazrin	3	0.90	0.92	0.96	0.72	0.92	0.95926412	0.0061
2474	4.05	2.84	M0RAK3	Solute carrier family 12 member 9	2	0.79	0.86	1.12	0.93	0.94	0.96332867	0.19
2475	4.05	3.18	Q7TPK0	Ac2-125	2	0.65	1.00	0.99	1.21	1.08	1.34443499	0.0235
2476	4.05	7.80	A0A0G2K756	Acyl-Coenzyme A binding domain containing 3	3	1.06	1.15	1.08	1.02	0.98	0.98691655	0.736
2477	4.05	6.71	Q68FS3	F-box only protein 7	3	1.24	1.00	0.97	1.00	0.95	0.97339684	0.306
2478	4.05	16.78	Q6AXU6	Hematological and neurological expressed 1 protein	2	1.31	1.22	1.11	1.01	1.20	0.93109482	0.0903
2479	4.05	9.36	F7F557	Acyl-coenzyme A thioesterase 8	2	1.27	0.59	1.15	1.08	0.97	1.01740917	0.739
2480	4.04	48.86	P15865	Histone H1.4	17	1.07	1.56	2.23	1.54	1.80	1.03648555	0.496
2481	4.04	11.84	A0A0G2K0J9	Gamma-aminobutyric acid receptor subunit beta-3	6	0.62	0.99	1.49	1.56	0.90	1.10037861	0.101
2482	4.04	8.74	B2RYN1	Fructosamine-3-kinase-related protein	3	0.74	0.91	0.67	0.61	0.38	1.05665177	0.0606
2483	4.04	20.87	D3Z8D7	40S ribosomal protein S26	2	0.90	0.91	0.91	0.96	0.89	0.97752127	0.605
2484	4.03	7.11	F1LQK6	Ephrin type-A receptor 6	6	0.94	0.83	0.86	0.99	0.96	1.0276151	0.632
2485	4.03	14.17	Q5FVM2	Glucose-6-phosphatase	3	0.87	0.83	0.68	0.60	0.72	1.01966836	0.604
2486	4.03	5.35	Q3KRC3	SRP receptor alpha subunit	3	1.00	1.02	1.04	0.96	0.97	0.97833469	0.491
2487	4.03	2.93	F1LTT7	DENN domain-containing	3	0.92	0.92	0.90	0.91	0.92	0.92851685	0.0612
2488	4.03	8.32	Q587K3	Potential RabGAP	3	1.07	0.94	1.01	1.16	1.03	1.05241229	0.0785
2489	4.03	1.23	F1M0U5	Neuroblastoma-amplified sequence	3	0.98	0.89	0.90	0.95	0.83	1.01115208	0.589
2490	4.03	7.03	F1LTX8	Lysophosphatidylglycerol acyltransferase 1	2	0.39	0.51	0.44	0.37	0.27	1.15188764	0.068
2491	4.03	7.64	Q4KLL0	Transcription elongation factor A protein 1	2	0.80	0.74	0.65	0.75	0.54	1.05168307	0.087
2492	4.03	5.66	Q5PQX1	Torsin-1A-interacting protein 1	2	0.66	0.60	0.82	0.73	1.25	0.99967705	0.991
2493	4.03	7.16	P02651	Apolipoprotein A-IV	2	0.65	0.61	0.85	1.00	0.82	1.1455179	0.0162
2494	4.03	4.53	D4A2H6	RNA binding protein fox-1 homolog	2	1.05	1.04	0.95	1.13	1.11	0.8532261	0.142
2495	4.03	27.71	P35171	Cytochrome c oxidase subunit 7A2, mitochondrial	5	0.89	0.96	0.91	0.96	0.88	0.93322703	0.318
2496	4.03	5.75	D3ZR52	Lysophosphatidylcholine acyltransferase 4	2	0.87	0.84	0.90	0.80	0.98	0.92723055	0.21

2497	4.03	14.81	Q9QYU2	Elongation factor Ts, mitochondrial	3	0.98	0.74	0.80	0.83	0.86	0.97225052	0.69
2498	4.03	12.87	P19103	Protein phosphatase 1 regulatory subunit 1A	2	0.99	0.94	0.95	0.89	1.13	0.97339684	0.768
2499	4.02	16.83	P63142	Potassium voltage-gated channel subfamily A member 2	6	1.07	0.97	0.82	0.95	0.92	0.91955005	0.171
2500	4.02	3.37	G3V7X4	ATP-binding cassette sub-family A member 2	7	0.92	0.93	1.10	0.94	0.99	1.005944	0.781
2501	4.02	7.69	D3ZZV7	RasGEF domain family, member 1A	3	0.90	0.77	0.79	0.74	0.77	1.01860874	0.0767
2502	4.02	1.70	D3ZHK4	RB1-inducible coiled-coil 1	3	0.85	0.72	0.77	0.77	0.72	1.04470817	0.0364
2503	4.02	4.10	Q63016	Large neutral amino acids transporter small subunit 1	2	0.90	0.89	1.17	1.42	1.02	0.98890237	0.785
2504	4.02	6.10	A0A0G2K9D7	Uncharacterized protein	3	1.11	0.94	0.96	1.01	0.98	0.95986273	0.381
2505	4.02	9.49	D4A6D9	HCLS1-binding protein 3	2	0.98	0.79	1.01	0.88	0.96	1.08748839	0.0237
2506	4.02	7.07	G3V9N7	Protein kinase C and casein kinase substrate in neurons	2	0.93	0.91	0.87	0.85	0.83	0.98268436	0.7
2507	4.02	13.24	F1M6C4	ADP-ribosylation factor-like GTPase 15	2	0.70	0.49	0.96	1.19	1.34	1.02939737	0.344
2508	4.02	14.40	D4A9A3	Centromere protein V	2	0.74	0.82	1.09	0.79	0.83	1.07095659	0.118
2509	4.02	10.61	Q6P9X2	Signal peptidase complex catalytic subunit SEC11	3	1.01	0.93	0.93	0.89	1.01	0.94802697	0.353
2510	4.02	27.08	B2RZB6	LSM8 homolog, U6 small nuclear RNA associated (S. cerevisiae)	3	1.15	1.14	1.04	1.04	1.11	0.92530443	0.164
2511	4.02	15.45	D3ZVI9	Parkinson disease 7 domain containing 1 (Predicted), isoform CRA_a	2	0.73	0.93	0.76	0.83	0.77	1.15668818	0.592
2512	4.01	26.84	Q62717	Calcium-dependent secretion activator 1	38	0.94	0.97	0.98	1.00	0.99	1.00744425	0.758
2513	4.01	5.73	A0A140TAB1	Phosphodiesterase	2	0.82	0.88	0.86	0.93	0.86	0.98739552	0.826
2514	4.01	4.75	G3V795	Solute carrier organic anion transporter family member	3	0.79	0.87	0.84	0.95	0.82	1.11728714	0.141
2515	4.01	3.21	P26769	Adenylate cyclase type 2	2	0.74	0.87	0.88	0.76	0.79	0.96486567	0.213
2516	4.01	7.88	Q66H09	Tetratricopeptide repeat domain 1	2	1.04	1.00	0.97	0.96	0.92	1.04898933	0.187
2517	4.01	6.08	D4A3D4	Abhydrolase domain containing 3 (Predicted)	2	1.09	0.48	1.37	0.93	1.33	1.00483596	0.918
2518	4.01	5.95	Q3MID9		2	1.11	1.00	1.10	0.86	0.90	1.50838008	0.02681
2519	4.01	9.57	Q63159	Ubiquinone biosynthesis	2	1.02	0.95	0.95	0.83	0.95	0.94605765	0.0773
2520	4.01	18.90	P38718	Mitochondrial pyruvate carrier 2	2	0.86	0.92	0.95	0.93	0.88	1.00842235	0.864
2521	4.01	28.26	A0A0G2K8I0	SH3 domain-binding glutamic acid-rich-like	2	0.79	1.02	1.21	0.61	1.45	1.01374866	0.748
2522	4.01	16.95	P62634	Cellular nucleic acid-binding protein	2	0.83	0.74	0.90	1.03	0.79	0.87721355	0.477
2523	4	22.26	F1M4G6	Uncharacterized protein	34	1.71	1.98	1.71	0.50	1.64	1.07624013	0.13
2524	4	43.46	A0A0G2JSM8	Cell division control protein 42 homolog	8	1.03	0.86	1.71	1.60	1.17	1.14076372	0.115
2525	4	9.92	Q4V7E5	Peptide chain release factor 1-like, mitochondrial	2	0.76	1.28	1.32	0.89	1.03	0.96580243	0.278
2526	4	5.11	D3ZFK6	Autophagy-related 16-like 1	2	0.67	0.74	0.80	0.32	0.74	0.99082351	0.66
2527	4	8.94	Q5PQQ1	tRNA modification GTPase GTPBP3, mitochondrial	2	0.70	0.70	0.62	0.77	0.71	1.09202055	0.268
2528	4	6.44	B2GV94	Fam134c protein	2	0.83	0.83	0.74	0.77	0.87	0.95032969	0.475
2529	4	5.63	Q66H63	Ganglioside-induced differentiation-associated-protein 2	2	1.28	1.63	1.21	1.19	1.21	1.08447741	0.228
2530	4	12.44	Q99P75	Ras-related protein Rab-9A	2	0.93	0.93	0.86	1.15	0.94	1.0181852	0.608
2531	4	16.08	Q6IN37	GM2 ganglioside activator	2	0.49	0.61	0.77	0.42	0.72	1.03562378	0.297
2532	4	6.80	A0A096MJY6	1,4-alpha-glucan-branching enzyme 1 (Fragment)	2	0.96	1.04	0.99	1.00	0.96	0.97157684	0.346
2533	4	1.26	A0A0G2KB93	Sodium channel protein	2	1.22	1.28	1.16	1.13	0.98	1.01988042	0.00585
2534	4	9.87	A0A0G2JZW1	Uncharacterized protein	3	0.34	1.26	0.81	0.39	1.13	0.93491039	0.321
2535	4	26.80	Q9WVA1	Mitochondrial import inner membrane translocase subunit Tim8 A	2	1.09	0.70	0.90	0.32	0.95	0.90000193	0.24
2536	4	29.20	B0K008	Eukaryotic translation initiation factor 1	2	0.80	0.92	0.79	0.88	0.90	0.95462101	0.354
2537	4	29.17	P62628	Dynein light chain roadblock-type 1	2	1.09	1.07	1.00	0.92	1.05	0.81056512	0.0156
2538	4	11.54	D4A110	CKLF-like MARVEL transmembrane domain-containing 4	2	0.54	0.76	0.46	0.59	0.57	1.0595855	0.382
2539	4	6.23	O35260	Nucleus accumbens-associated protein 1	2	1.07	0.88	1.02	0.69	0.99	1.03741994	0.633
2540	4	3.41	D4A1J3	Paralemmin 3	2	0.98	0.66	0.74	0.95	0.77	1.01621101	0.818
2541	4	38.71	A0A0G2K8Q8	Uncharacterized protein	4	1.37	0.66	0.81	1.09	0.86	0.91383145	0.383
2542	4	16.15	Q6P791	Ragulator complex protein LAMT	2	0.82	0.71	1.14	0.96	1.10	1.0807254	0.0269

2543	4	30.00	D3Z9R8	6.8 kDa mitochondrial proteolipid	3	4.61	3.19	2.61	1.26	3.73	1.0164928	0.749
2544	4	22.76	A0A0G2JZB6	Uncharacterized protein	5	0.87	0.93	0.86	0.95	0.74	0.99864235	0.98
2545	4	14.46	Q5XIG9	Mitochondrial fission process 1	2	0.63	1.16	0.84	0.65	0.69	0.87175824	0.149
2546	4	10.54	D3ZL45	Uncharacterized protein	2	0.58	0.82	0.77	0.54	0.65	1.03289958	0.427
2547	4	14.24	D3ZFB6	Proline-rich transmembrane protein 2	2	0.99	0.82	0.65	0.91	0.75	0.95468719	0.195
2548	4	18.18	Q7TPB7	MAL2A	2	1.07	1.32	0.92	1.21	0.96	0.86573657	0.00779
2549	4	18.92	D3ZJS3	RCG43475	3	1.17	0.80	0.73	0.86	1.04	0.86633686	0.201
2550	4	16.22	O08776	NADH dehydrogenase [ubiquinone] 1 alpha subcomplex assembly factor 3	2	0.89	1.07	1.03	1.03	1.03	1.07177346	0.406
2551	4	9.73	Q5U2U4	Secretory carrier-associated membrane protein	2	0.95	0.88	0.95	0.88	0.85	0.91446509	0.135
2552	3.99	5.25	M0R851	Similar to Ubiquitin carboxyl-terminal hydrolase 4 (Ubiquitin thiolesterase 4) (Ubiquitin-specific processing protease 4) (Deubiquitinating enzyme 4) (Ubiquitous nuclear protein), isoform CRA_b	4	1.22	0.85	0.87	1.06	1.08	1.08748839	0.188
2553	3.99	40.70	D4A9P7	RCG40058	3	1.12	0.59	1.26	0.63	1.07	0.97515266	0.741
2554	3.98	12.56	Q8CGS4	Charged multivesicular body protein 3	3	1.09	1.77	1.36	1.16	0.95	0.98397939	0.823
2555	3.96	9.26	Q5U211	Sorting nexin-3	2	0.79	0.95	1.37	1.09	1.02	0.95687342	0.187
2556	3.96	18.08	G3V616	Divalent cation tolerant protein CUTA, isoform CRA_b	2	1.13	0.42	0.72	0.31	0.71	0.86753869	0.0118
2557	3.95	3.44	F1LRN5	Netrin receptor DCC	4	0.94	0.85	0.94	0.95	0.85	1.02002181	0.0611
2558	3.95	5.10	Q6AXQ5	2',5'-phosphodiesterase 12	2	0.93	0.89	1.06	0.81	0.72	0.98999971	0.823
2559	3.94	3.58	F1M8V2	Ubiquitination factor E4B	4	1.21	0.78	0.75	0.96	0.86	1.06858376	0.223
2560	3.94	6.00	Q5XIG0	ADP-ribose pyrophosphatase,	2	2.03	2.07	1.60	1.85	1.77	0.98760086	0.893
2561	3.93	3.01	F1LQI2	Roundabout homolog 1	5	0.53	0.49	0.53	0.53	0.75	0.97779233	0.161
2562	3.93	7.54	Q6TUG0	DnaJ homolog subfamily B member 11	2	1.06	1.02	1.00	0.94	1.06	1.03548022	0.246
2563	3.93	5.03	D3ZVN7	Protoporphyrinogen oxidase	2	1.04	0.93	1.06	1.04	1.08	1.01248463	0.747
2564	3.92	16.55	F1LNW6	Lymphocyte antigen 6 complex, locus H (Predicted), isoform CRA_a	3	1.54	0.12	0.59	0.67	0.69	0.98698496	0.819
2565	3.91	7.48	Q5M893	Hydroxymethylbilane synthase	2	0.98	1.06	1.19	1.06	1.17	1.09202055	0.193
2566	3.89	7.16	A0A0G2K0J2	Voltage-dependent calcium channel subunit alpha-2/delta-2	6	0.32	0.37	0.72	0.52	0.61	0.97326191	0.207
2567	3.89	3.04	D4ABK9	Kinase D-interacting substrate of 220 kDa	4	0.72	0.53	0.75	0.50	0.48	1.00183158	0.932
2568	3.88	25.74	M0R9A7	Glutamate receptor 1	27	0.92	1.01	0.93	1.08	1.10	1.12194348	0.0532
2569	3.88	6.14	A0A0H2UHP1	Retinal dehydrogenase 1	3	0.90	0.79	0.88	0.77	0.95	1.02825636	0.169
2570	3.88	3.24	Q91ZY8	E3 ubiquitin-protein ligase TRIM9	2	0.90	1.05	1.03	0.99	0.97	1.00751408	0.803
2571	3.88	9.83	Q3B7D1	Ubiquitin-conjugating enzyme E2 Z	3	0.90	0.82	0.87	0.80	0.89	0.92787348	0.345
2572	3.88	10.56	G3V985	SC	2	0.89	1.01	1.07	1.10	1.01	1.15909595	0.0356
2573	3.87	12.05	Q9ES53	Ubiquitin fusion degradation protein 1	3	0.87	0.87	1.03	1.01	1.00	0.92594602	0.0493
2574	3.85	3.08	D3ZHL1	Kinase suppressor of ras 1	2	0.79	0.96	0.97	1.09	1.11	1.34630007	0.00697
2575	3.85	2.89	A0A0G2JTH7	Ubiquitin protein ligase	2	0.42	0.61	0.64	0.56	0.81	1.00521214	0.813
2576	3.84	28.57	D3ZMS0	Gene-rich cluster, C10 gene	3	0.75	0.90	0.76	0.64	0.95	1.00115823	0.984
2577	3.82	9.34	G3V8C0	Dynactin subunit 5	2	1.11	1.22	1.08	1.14	1.12	0.97110554	0.454
2578	3.81	4.51	A0A0G2K0S7	RAP1 GTPase-activating protein 2	2	1.01	1.00	1.11	1.01	1.07	0.99699626	0.908
2579	3.81	8.60	P84039	Ectonucleotide pyrophosphatase/phosphodiesterase family member 5	4	0.95	0.89	1.01	0.81	0.87	0.97758903	0.494
2580	3.8	23.67	Q9Z252	Protein lin-7 homolog B	5	0.80	0.83	1.04	1.15	0.81	0.96206082	0.254
2581	3.8	2.42	G3V7U1	Glutamate receptor, metabotropic 1, isoform CRA_b	4	1.41	1.18	1.45	0.93	0.65	1.03519317	0.312
2582	3.8	16.97	D3Z8Q7	Family with sequence similarity 96, member B	2	0.94	1.10	1.04	0.79	0.95	1.02818509	0.206
2583	3.79	25.87	D3ZP15	RAB9B, member RAS oncogene family	7	0.62	0.65	0.54	0.34	0.67	1.04131027	0.0289
2584	3.79	9.49	P05982	NAD(P)H dehydrogenase [quinone] 1	3	1.46	1.56	1.32	1.37	0.69	1.02533831	0.0506
2585	3.79	2.35	Q5FVK6	Coiled-coil and C2 domain-containing protein 1B	2	0.72	0.89	0.78	0.90	0.77	1.03011114	0.0889
2586	3.79	1.98	D3ZT64	Autophagy-related 2A	2	1.18	1.07	1.08	1.10	1.24	1.07251662	0.111

2587	3.78	2.19	D3ZS72	Phosphatidylinositol-3,4,5-trisphosphate-dependent Rac exchange factor 1	3	1.06	1.20	1.14	1.08	1.13	1.00744425	0.721
2588	3.78	2.74	D3ZHI8	Trafficking protein particle complex 11	3	1.24	1.50	1.25	1.21	1.22	1.00657872	0.79
2589	3.78	14.20	D4A732	Multivesicular body subunit 12B	2	1.08	0.98	0.96	1.07	1.33	1.11806185	0.153
2590	3.77	2.94	A0A0G2K562	Disintegrin and metalloproteinase domain-containing protein 10	2	1.07	0.98	1.10	1.13	1.16	1.01059154	0.687
2591	3.77	19.93	A2VCX1	TIP41-like protein	4	1.45	1.51	1.46	0.79	1.31	1.01234427	0.715
2592	3.77	11.07	Q5U3Z5	Bri3 binding protein	2	1.26	0.61	0.33	0.58	0.70	0.92274249	0.241
2593	3.76	3.59	Q8CJB9	E3 ubiquitin-protein ligase BRE1B	3	1.02	0.88	0.90	0.93	0.86	1.05204761	0.146
2594	3.76	6.60	Q9EPB1	Dipeptidyl peptidase 2	2	1.17	1.17	1.13	1.38	1.25	1.00320052	0.902
2595	3.76	16.41	D3ZX69	39S ribosomal protein L10, mitochondrial	3	0.88	0.83	0.99	0.77	0.80	0.99013696	0.888
2596	3.75	7.44	Q499Q3	Interferon-induced GTP-binding protein Mx2	3	0.86	1.03	0.95	0.91	0.82	1.07474917	0.192
2597	3.75	5.88	A0A0G2K824	Mannose-1-phosphate guanyltransferase alpha	2	1.03	0.97	0.95	1.05	0.94	0.76524839	0.137
2598	3.74	7.12	Q5XID7	Armadillo repeat-containing X-linked protein 3	2	1.21	1.25	1.05	1.11	0.99	1.02292474	0.321
2599	3.74	3.81	A0A0G2K5T1	Calmodulin-regulated spectrin-associated protein 1	4	1.00	1.09	1.14	1.06	1.01	1.0244148	0.4
2600	3.74	6.87	A0A0G2QC33	Cysteine protease	2	0.93	0.65	0.96	1.27	0.69	0.89751005	0.0342
2601	3.73	4.17	Q9JLH7	CDK5 regulatory subunit-associated protein 3	2	0.90	0.94	0.90	0.79	0.95	0.99942139	0.983
2602	3.73	5.38	D4A617	Ectonucleoside triphosphate diphosphohydrolase 1	2	1.12	1.04	1.16	1.22	1.14	1.01501426	0.763
2603	3.72	4.92	M0R617	SH2-B PH domain containing signaling mediator 1, isoform CRA_a	3	1.05	1.09	0.94	0.88	0.87	0.85856544	0.0158
2604	3.72	6.20	P16391	RT1 class I histocompatibility antigen,	2	0.41	0.86	0.74	0.40	0.48	0.86214355	0.024
2605	3.72	13.82	B2GUZ7	Tubulin folding cofactor C	4	0.59	0.60	0.95	0.31	0.79	0.97346432	0.515
2606	3.71	26.92	B2RYQ5	Enhancer of rudimentary homolog	3	1.13	0.93	0.90	1.04	1.03	1.0535801	0.177
2607	3.7	2.52	G3V927	Discs, large homolog-associated protein 4 (Drosophila)	2	1.18	1.31	1.10	0.98	1.33	1.02654723	0.432
2608	3.7	7.13	F1LNV5	Calcium uptake protein 1, mitochondrial	3	1.03	1.01	1.07	0.98	0.97	1.00709515	0.855
2609	3.69	6.55	P09034	Argininosuccinate synthase	3	0.90	0.92	0.92	0.90	0.93	0.96888678	0.451
2610	3.69	1.95	B5DEZ4	Tcerg1 protein	2	0.91	0.92	0.91	0.98	0.93	1.07698638	0.0246
2611	3.69	9.57	B0BNE3	Similar to trafficking protein particle complex 5 (Predicted)	2	2.03	1.36	2.42	1.19	1.92	1.02932602	0.405
2612	3.69	16.17	Q6BBI8	Ubiquitin-fold modifier-conjugating enzyme 1	3	1.14	1.32	1.13	1.47	1.00	0.97029813	0.263
2613	3.69	10.22	D4A1B8	Dynactin subunit 3	2	1.04	1.08	1.11	1.03	1.07	1.01402977	0.523
2614	3.68	1.15	D3ZLS5	HECT domain E3 ubiquitin protein ligase 1	4	0.69	0.79	1.50	1.75	0.99	0.97305955	0.255
2615	3.68	8.78	Q6P792	Four and a half LIM domains 1	2	1.27	0.79	1.45	1.10	1.45	1.0138892	0.635
2616	3.67	12.61	Q63798	Proteasome activator complex subunit 2	3	0.48	1.20	1.27	1.09	0.74	1.11651296	0.0167
2617	3.66	4.43	B2RYB8	Integrin beta	2	0.42	0.29	1.54	0.60	0.55	1.07773315	0.0734
2618	3.66	7.21	A0A0G2JXS8	Translocon-associated protein subunit alpha	2	1.02	1.09	1.21	1.17	1.28	0.99698935	0.949
2619	3.66	9.84	G3V6S3	Calumenin	2	0.70	0.99	1.05	0.50	0.68	1.01087177	0.81
2620	3.66	2.51	G3V9R2	Complement factor H	2	0.95	0.83	1.07	0.95	0.93	1.03677296	0.319
2621	3.65	3.16	F1LP76	Elongator complex protein 1	4	0.88	0.77	0.90	1.10	0.95	0.96787994	0.478
2622	3.65	19.44	A0A0G2JZZ4	Uncharacterized protein	3	0.88	0.95	0.82	0.63	0.82	1.03677296	0.683
2623	3.65	7.25	Q5U2X6	Coiled-coil domain-containing protein 47	3	0.83	0.96	0.96	1.04	1.13	1.02954008	0.294
2624	3.65	6.47	D3ZRH1		2	0.86	1.12	1.00	1.27	1.29	0.9862327	0.257
2625	3.64	13.39	A0A0G2K038		3	0.90	1.00	0.92	0.79	0.86	0.99247317	0.715
2626	3.64	3.05	Q4VFZ4	Katanin p80 WD40 repeat-containing subunit B1	2	0.91	0.94	0.89	0.93	0.89	1.00645314	0.835
2627	3.64	8.74	D3ZSU3	Similar to solute carrier family 7, member 14	4	1.39	1.46	1.17	1.28	1.29	0.98105097	0.5
2628	3.63	21.40	F1LW77	RAB33B, member RAS oncogene family	6	0.79	1.13	0.84	1.13	1.20	1.03146867	0.184
2629	3.63	5.11	A0A0G2JT26	Uncharacterized protein	3	0.67	0.78	1.06	1.51	0.88	1.04593993	0.0572
2630	3.62	4.79	F1M049	Ataxin 2	3	1.05	0.92	0.95	1.02	1.08	1.03469101	0.291
2631	3.62	18.87	D3ZJK8	ATPase phospholipid-transporting 8A1	2	1.32	0.74	0.67	1.01	1.46	0.91066983	0.475
2632	3.61	5.30	Q01177	Plasminogen	4	1.11	1.06	1.10	1.04	1.16	1.07922824	0.0884
2633	3.61	22.63	A0A096MJZ0	Dynein, axonemal, light chain 1	3	0.90	0.87	0.99	0.90	0.79	1.08824244	0.294

2634	3.61	31.30	P62890	60S ribosomal protein L30	3	0.95	0.92	0.96	0.95	0.86	1.05731115	0.345
2635	3.6	6.26	A0A0G2JSN5	Potassium voltage-gated channel subfamily D member 3	3	1.12	0.81	0.74	0.59	0.72	1.11265012	0.0267
2636	3.6	2.59	D3ZN39	Ubiquitin specific protease 8 (Predicted)	2	0.90	1.01	0.99	1.04	0.88	1.04811717	0.128
2637	3.6	8.91	D3ZY50	ATP synthase mitochondrial F1 complex assembly factor	2	1.39	1.04	0.82	0.94	1.16	0.91891288	0.299
2638	3.59	7.97	G3V828	Canopy FGF-signaling regulator 3	2	1.15	1.04	1.16	0.98	1.06	1.21335356	0.00154
2639	3.59	15.56	P02696	Retinol-binding protein 1	2	0.83	1.14	1.04	0.44	0.63	0.93627225	0.185
2640	3.58	25.77	B0BN19	RAB22A, member RAS oncogene family	4	0.71	0.96	0.45	0.80	0.44	0.87660572	0.156
2641	3.58	2.28	D4A517	Solute carrier family 39 (Zinc transporter), member 10 (Predicted), isoform	2	0.86	0.82	1.06	0.86	0.87	1.06754731	0.03
2642	3.58	6.76	D3ZAW4	Abhydrolase domain containing 4 (Predicted)	2	0.33	0.53	0.87	0.44	0.16	1.00434853	0.934
2643	3.57	11.28	D3ZR63	Glucose-fructose oxidoreductase domain-containing 1	4	1.58	1.07	0.90	0.90	1.26	1.02505406	0.645
2644	3.56	4.76	Q9R0T3	DnaJ homolog subfamily C member 3	3	0.42	0.56	0.95	0.49	0.59	2.12137548	0.02044
2645	3.56	7.80	A0A0G2K9B4	Mitochondrial ribosomal protein L15	2	0.89	1.13	0.98	1.07	0.89	1.02257028	0.605
2646	3.56	9.06	B5DFF4	Vacuolar protein sorting 37C (Yeast) (Predicted)	3	0.95	0.95	1.09	0.73	0.82	1.12818214	0.0268
2647	3.56	14.46	A0A0G2JUA5	AHNAK nucleoprotein	8	1.79	2.15	1.98	2.03	2.07	1.06821348	0.0487
2648	3.55	3.71	D3ZTM0	Neurexin-2	5	0.96	0.93	0.90	0.95	0.98	0.98671134	0.395
2649	3.55	2.84	D4A914	5'-3' exoribonuclease 2	2	0.33	0.65	0.52	0.82	0.51	0.98350207	0.54
2650	3.55	5.83	Q9WVJ6	Tissue-type	3	1.13	1.04	0.98	1.01	0.98	0.99965764	0.993
2651	3.54	5.56	Q5FVC4	DnaJ (Hsp40) homolog, subfamily B, member 12	2	0.79	1.01	0.97	0.65	0.91	1.01066159	0.623
2652	3.53	27.06	Q498E0	Thioredoxin domain-containing protein 12	4	0.93	1.04	0.95	0.97	1.06	0.96935701	0.811
2653	3.52	6.40	D3ZAW2	Phosphatidylserine decarboxylase proenzyme, mitochondrial	3	0.94	1.05	0.89	1.07	0.83	1.10726458	0.504
2654	3.52	19.73	P02767	Transthyretin	3	0.07	0.12	0.15	0.42	0.40	0.80888135	0.529
2655	3.52	6.07	A0A0H2UHM	Haptoglobin	2	2.05	1.17	0.94	1.20	0.91	1.04087729	0.451
2656	3.52	8.37	G3V7B6	Phosphatidylglycerophosphatase and protein-tyrosine phosphatase 1	2	1.05	1.13	1.05	1.12	0.99	0.95753691	0.203
2657	3.51	5.02	D3ZHG4		4	0.86	0.86	0.96	0.79	0.98	1.05350707	0.321
2658	3.51	7.07	P51907	Excitatory amino acid transporter 3	3	0.63	0.94	0.54	0.35	0.20	1.02975419	0.164
2659	3.5	6.95	Q63639	Retinal dehydrogenase 2	3	1.00	1.20	0.43	1.09	1.01	0.97252012	0.0795
2660	3.49	4.67	D4ACB6	Pleckstrin and Sec7 domain containing 2 (Predicted)	2	1.09	1.12	0.91	0.91	0.90	0.9797598	0.689
2661	3.49	5.57	Q9EQP5	Prolargin	2	1.10	1.32	1.00	1.24	1.32	0.94141321	0.00308
2662	3.48	3.50	F1LQG0	Huntingtin-associated protein 1	2	1.05	0.87	0.87	0.84	0.87	0.99795731	0.94
2663	3.48	4.39	D3ZGF1	CD44 antigen	2	2.38	1.58	10.67	5.15	6.03	1.33237483	0.0363
2664	3.48	9.07	F1LRJ9	Selenium-binding protein 1	3	0.72	0.61	0.45	0.51	0.39	1.04986222	0.0848
2665	3.46	2.67	D3ZAX5	Calcium homeostasis endoplasmic reticulum protein	2	0.74	0.77	0.61	0.60	0.63	1.01945635	0.845
2666	3.45	32.78	F1M5N3	Ankyrin 2	121	1.28	0.82	0.95	0.45	0.60	1.00143585	0.944
2667	3.45	13.13	E9PU42	DSCR3 arrestin fold-containing	3	1.07	0.86	1.26	1.16	1.10	1.01945635	0.239
2668	3.44	7.69	Q5PQL2	Cell differentiation protein RCD1 homolog	2	0.37	0.06	0.38	0.37	0.21	0.99320264	0.573
2669	3.43	8.99	Q6P7B6	Ephrin B1	2	0.97	0.90	0.98	1.08	0.97	1.10803235	0.0353
2670	3.42	5.02	P28572	Sodium- and chloride-dependent glycine transporter 1	2	0.90	0.95	0.99	0.91	0.94	1.03311439	0.567
2671	3.41	2.72	G3V8T3	Adenosine deaminase, RNA-specific, isoform	3	0.94	0.74	1.05	0.64	0.72	1.0276151	0.119
2672	3.41	6.24	A0A0G2K3W	Von Willebrand factor A domain-containing 8	2	0.23	0.80	0.69	0.42	0.54	0.98466167	0.526
2673	3.4	3.58	D3Z9E6	Cleavage and polyadenylation specific factor 2 (Predicted)	2	0.60	0.98	1.03	1.08	0.76	1.00271388	0.91
2674	3.39	3.22	O35346	Focal adhesion kinase 1	3	1.22	1.08	0.76	1.07	0.82	1.00468274	0.891
2675	3.39	11.44	A1L1K8	Hyaluronan binding protein	2	0.79	1.14	0.90	0.76	0.81	0.81676899	0.0148
2676	3.39	4.46	F1M9N7	Arf-GAP domain and FG repeat-containing protein 1	2	1.26	1.75	1.36	1.67	1.39	1.00485686	0.949
2677	3.39	4.40	A0A0G2JXG3	Cell cycle control protein 50A	2	0.80	1.19	1.03	0.65	1.01	1.07326029	0.0615
2678	3.39	8.50	Q6MG82	Proline-rich transmembrane protein 1	2	1.31	0.97	0.98	1.47	1.32	0.92723055	0.289

2679	3.38	1.99	F1MAQ4	Trafficking protein particle complex 10	2	0.94	0.97	1.03	1.05	0.93	0.98862823	0.805
2680	3.37	9.48	Q68G39	Cyclin-dependent kinase 16	4	0.95	0.86	0.98	0.97	0.84	0.98534442	0.517
2681	3.37	2.78	F1M7L9	Uncharacterized protein	2	0.86	0.91	0.86	0.96	0.87	0.9821396	0.334
2682	3.37	3.58	Q5M7V8	Thyroid hormone receptor-associated protein 3	3	1.03	1.00	1.09	0.99	0.92	1.08372597	0.0967
2683	3.37	17.09	P60522	Gamma-aminobutyric acid receptor-associated protein-like 2	2	1.61	1.63	1.29	1.53	1.34	0.9459265	0.0633
2684	3.37	3.95	Q6AYK3	Inositol-3-phosphate synthase 1	2	1.03	0.72	0.50	1.16	0.64	1.06584674	0.121
2685	3.35	3.53	Q80Z70	Protein sel-1 homolog 1	2	0.93	1.04	0.95	1.22	1.06	0.99709302	0.925
2686	3.35	3.86	D3ZJ91	DDHD domain containing 2	2	0.95	0.90	0.90	0.90	0.94	1.02306655	0.799
2687	3.35	2.14	B2RYI0	WD repeat-containing protein 91	2	0.87	0.92	1.06	0.98	0.98	0.88945899	0.105
2688	3.35	11.27	P70500	CDP-diacylglycerol--inositol 3-	3	1.03	1.03	1.04	0.86	1.14	1.01966836	0.095
2689	3.34	12.63	B0BN86	Transmembrane protein 11, mitochondrial	2	1.27	1.37	1.37	1.36	1.46	1.01508462	0.613
2690	3.33	12.48	Q56R17	Importin subunit alpha	4	1.43	0.95	0.69	0.79	0.63	1.03627004	0.63
2691	3.33	2.48	D3ZJ32	Extended synaptotagmin 2	2	1.37	0.71	0.89	0.91	1.08	0.92466328	0.0463
2692	3.32	8.81	A0A096MIT7	Synapsin-3	5	1.14	0.83	0.86	0.90	0.74	1.04478059	0.58
2693	3.32	5.43	B0BN56	28S ribosomal protein S31, mitochondrial	2	0.97	0.85	0.91	0.86	0.85	1.02868409	0.394
2694	3.31	2.76	A0A0G2JSH6	Transient receptor potential cation channel subfamily V member 2	3	0.85	0.62	0.38	0.86	0.55	0.96996191	0.00499
2695	3.31	2.81	G3V9M1	DEAD (Asp-Glu-Ala-Asp) box polypeptide 23 (Predicted), isoform CRA_b	2	0.82	0.78	0.69	0.90	0.75	0.97124017	0.392
2696	3.3	20.25	B5DFI3	Adaptor protein complex AP-1, sigma 1 (Predicted), isoform CRA_b	3	1.24	1.45	1.10	0.67	1.41	0.91003882	0.042
2697	3.3	1.29	D3ZLW4	Tuberin	2	0.61	0.78	0.44	0.64	0.49	1.04637502	0.00286
2698	3.3	3.43	Q811Q2	Chloride intracellular channel protein 6	2	0.65	0.78	0.52	0.82	0.82	1.0660684	0.12
2699	3.3	13.24	F1LZX7	60S ribosomal protein L17	2	1.02	1.07	1.04	1.02	1.10	1.01101192	0.804
2700	3.29	7.60	Q9Z1H9	Protein kinase C delta-binding protein	2	0.90	1.08	1.10	1.04	1.15	1.02555154	0.364
2701	3.29	19.19	D4A4P3	NADH:ubiquinone oxidoreductase subunit B3	4	1.06	1.38	0.88	0.74	0.87	0.84206295	0.00153
2702	3.28	15.04	P70567	Tropomodulin-1	4	1.14	0.96	0.75	0.66	0.74	1.03232698	0.045
2703	3.28	9.17	Q5I0K3	Citrate lyase subunit beta-like protein, mitochondrial	2	0.99	1.11	0.86	1.13	1.00	1.12272142	0.0293
2704	3.28	1.09	D4A3W2	Crumbs 2, cell polarity complex component	2	0.69	0.69	0.58	0.58	0.66	0.90375273	0.0274
2705	3.27	23.62	A0A0G2JW34	CDGSH iron sulfur domain	3	1.18	0.50	1.09	1.20	0.99	0.98821715	0.774
2706	3.27	11.40	Q8VHI8	Vesicle transport protein SEC20	3	1.02	1.16	0.95	1.06	1.00	0.96734338	0.444
2707	3.27	17.59	D3Z9I1	Cytochrome C oxidase assembly factor 3	3	1.04	0.95	0.98	0.93	0.91	0.88331505	0.102
2708	3.26	26.32	P62076	Mitochondrial import inner membrane translocase subunit Tim13	5	0.69	0.58	0.81	0.67	0.65	0.88392753	0.00357
2709	3.25	19.19	D4A304	DIRAS family GTPase 1	4	0.82	1.21	0.42	0.50	0.61	0.96593633	0.0986
2710	3.25	3.03	Q9QX19	Citron rho-interacting serine/threonine kinase	4	1.03	0.88	0.97	0.96	1.00	1.11651296	0.036
2711	3.24	2.07	A0A0G2K939	Dedicator of cytokinesis 10	4	1.01	1.16	0.90	0.86	0.71	1.0341891	0.05
2712	3.24	5.14	Q5U2P2	Immunoglobulin superfamily member 11	2	1.53	1.13	1.54	1.27	1.36	0.85381771	0.114
2713	3.24	14.04	Q2IBC6	Caveolin	2	1.02	1.04	1.29	1.46	1.27	1.1234999	0.0279
2714	3.23	9.66	P24090	Alpha-2-HS-glycoprotein	3	0.63	0.41	0.89	0.97	1.14	1.00283899	0.951
2715	3.23	10.47	Q6AYH6	ER membrane protein complex subunit 10	2	0.99	1.12	1.13	1.02	0.89	0.96586938	0.561
2716	3.22	3.20	A0A0G2JTD8	Microtubule affinity-regulating kinase 4	2	0.80	0.87	0.82	0.86	0.94	0.99347117	0.827
2717	3.21	19.35	D3ZZP2	RAB39, member RAS oncogene family (Predicted)	5	0.88	0.91	0.94	0.83	0.85	1.07326029	0.0924
2718	3.21	7.90	Q6AYT0	Quinone oxidoreductase	2	0.79	0.67	0.92	0.70	0.67	0.97982771	0.0886
2719	3.21	3.76	A0A0G2JZY3	RALBP1-associated Eps domain-containing 1	2	1.26	1.21	1.28	1.10	1.06	0.9641971	0.152
2720	3.2	2.88	Q5PXK5	Potassium voltage-gated channel subfamily C member 3	2	1.02	1.29	1.00	1.04	1.10	1.08975211	0.0164
2721	3.2	12.14	G3V7J2	Interferon-inducible double-stranded RNA-dependent protein kinase activator A	2	0.66	0.52	0.70	0.54	0.67	0.95813444	0.128
2722	3.19	11.48	A0A0G2K8M7	Tumor protein D52-like 1	2	1.39	1.56	0.71	0.61	1.00	1.00431372	0.489
2723	3.19	2.91	O54861	Sortilin	2	0.80	0.57	1.10	0.97	0.82	1.0081428	0.514
2724	3.19	10.90	M0R665	60S ribosomal protein L29	2	1.04	1.07	1.32	0.98	1.15	1.04840781	0.424

2725	3.18	7.16	Q7TP15	S-methyl-5'-thioadenosine phosphorylase	2	0.99	1.02	0.95	1.15	1.12	0.98951947	0.894
2726	3.18	4.38	D3ZT47	Family with sequence similarity 171, member A2	3	1.04	1.02	1.04	0.92	1.02	1.07251662	0.191
2727	3.18	21.49	P60841	Alpha-endosulfine	3	1.11	1.11	1.05	1.00	1.04	0.85145371	0.0861
2728	3.18	24.75	P05942	Protein S100-A4	3	0.93	1.27	12.59	3.10	4.83	1.42701451	0.0714
2729	3.17	18.71	P63081	V-type proton ATPase 16 kDa proteolipid subunit	4	0.91	1.10	0.85	0.77	1.02	0.8906929	0.224
2730	3.16	1.71	A0A0G2JZG4	Nuclear receptor coactivator 1	2	1.32	1.03	0.86	1.06	1.32	1.03562378	0.11
2731	3.16	5.91	Q1M168	Caytaxin	2	0.77	1.02	0.82	1.25	0.88	1.00465489	0.87
2732	3.16	8.04	D3ZLN7	C	2	1.18	1.25	1.00	1.05	1.04	1.00215801	0.957
2733	3.15	6.81	D3ZVU4	Ribokinase	2	0.37	0.41	0.85	0.70	0.69	0.8287449	0.019
2734	3.15	7.39	Q923V4	F-box only protein 6	2	0.94	0.97	0.82	0.91	1.09	1.02002181	0.464
2735	3.14	3.43	Q3ZU82	Golgin subfamily A member	2	0.71	0.73	0.85	0.68	0.64	1.12661923	0.015
2736	3.13	46.17	D3ZZ99	Alpha-adducin	35	0.95	1.27	0.99	1.11	1.09	0.94180481	0.0492
2737	3.13	3.48	A0A0G2JW85	Ras GTPase-activating protein 3	2	1.19	0.87	0.96	1.07	0.94	1.06252737	0.0284
2738	3.13	4.98	F1MA89	Cyclin Y	2	0.57	0.71	0.62	0.54	0.47	0.97522026	0.185
2739	3.12	8.19	B2RZ68	DDB1 and CUL4-associated factor 7	2	1.09	0.82	1.16	1.14	0.80	0.92980494	0.165
2740	3.12	6.51	A0A0G2K6G2	Adipocyte plasma membrane-associated	2	1.61	1.13	2.05	1.85	2.27	1.06017322	0.383
2741	3.11	2.40	G3V6H2	Pre-mRNA processing factor 8, isoform CRA_a	5	0.94	0.91	1.03	1.06	1.02	1.03018254	0.33
2742	3.1	2.19	G3V7M0	CCR4-N	5	1.01	1.08	1.04	1.00	1.04	1.15989966	0.0545
2743	3.1	13.76	Q642A4	UPF0598 protein C8orf82 homolog	2	1.18	1.01	1.46	1.50	0.98	0.98193539	0.866
2744	3.1	25.00	M0RA26	Ribosomal protein S27-like	3	1.41	0.19	1.22	0.74	1.26	1.03282799	0.819
2745	3.09	5.56	A0A0G2K9T1	E3 ubiquitin-protein ligase	5	0.90	1.07	0.90	1.08	1.05	0.99775672	0.959
2746	3.08	8.58	Q5U2U2	Crk-like protein	3	1.03	0.92	1.13	1.04	0.95	1.03950738	0.262
2747	3.07	7.39	G3V851	Solute carrier family 17 (Sodium-dependent inorganic phosphate cotransporter), member 6	4	0.52	0.65	0.53	0.68	0.45	1.00029532	0.993
2748	3.07	2.91	A0A0H2UHV4	Eukaryotic translation initiation factor 5B	2	1.02	1.08	1.10	1.07	1.02	1.00482203	0.801
2749	3.07	4.54	F1LVA8	Breast cancer anti-estrogen resistance protein 1	2	1.06	0.97	0.94	0.69	1.18	0.99757693	0.976
2750	3.07	6.07	A0A0G2QC38	Serine and arginine-rich-splicing factor 11	2	1.11	1.09	1.05	1.00	1.08	0.96406345	0.12
2751	3.06	1.62	D3ZWS0	Scribbled planar cell polarity protein	2	1.01	1.16	1.10	1.05	1.05	0.99164799	0.678
2752	3.06	7.06	Q4V8K5	BR	2	0.62	0.54	0.41	0.70	0.65	1.07624013	0.26
2753	3.06	3.28	F1LT71	Echinoderm microtubule associated protein like 4 (Predicted), isoform CRA_a	2	0.95	1.16	1.05	0.91	1.03	1.04030027	0.276
2754	3.05	3.01	D3ZD89	N(alpha)-acetyltransferase 15, NatA auxiliary subunit	2	0.89	0.83	0.95	0.88	0.94	1.07251662	0.115
2755	3.05	5.39	Q642B0	Glypican 4	2	0.88	0.96	1.25	1.01	0.79	0.99752852	0.916
2756	3.05	4.75	A0A0G2K0W1	Protein tweety homolog	2	1.27	1.72	1.41	1.24	1.12	0.94789555	0.0178
2757	3.04	30.49	D3ZUW0	Pleckstrin and Sec7 domain-containing 3	12	1.06	1.21	1.15	1.14	1.07	0.85145371	0.0245
2758	3.04	12.30	A0A0G2JXN2	Tripartite motif protein 46 (Predicted)	6	0.98	1.03	0.61	0.96	0.87	0.91827616	0.0439
2759	3.04	9.38	G3V9N1	RCG21137	4	1.09	1.11	1.06	0.97	0.94	1.00423019	0.648
2760	3.04	10.20	Q6AY58	B-cell receptor-associated protein 31	3	0.85	1.00	0.99	0.97	1.05	1.08598186	0.105
2761	3.04	29.41	Q5I0P2	Glycine cleavage system H protein, mitochondrial	2	1.21	0.24	1.56	0.44	1.21	0.99109826	0.839
2762	3.04	7.65	Q4KLZ3	DAZ associated protein 1	2	0.86	0.90	1.07	1.10	1.04	1.02009252	0.705
2763	3.04	9.04	M0RBL8	Uncharacterized protein	3	1.61	1.58	1.47	0.38	2.11	1.09581177	0.161
2764	3.04	11.58	D4ADD8	Dynactin 6 (Predicted), isoform CRA_b	2	1.00	0.88	0.89	0.88	1.05	0.88515377	0.125
2765	3.03	13.03	Q9EQH5	C-terminal-binding protein	7	1.37	1.19	1.25	1.34	1.15	0.93109482	0.189
2766	3.03	2.75	Q63433	Serine/threonine-protein kinase N1	3	1.11	0.99	0.99	1.03	0.97	1.01136237	0.714
2767	3.03	5.67	Q7TP14	Cc1-9	2	0.65	1.28	0.93	0.68	0.41	1.03677296	0.156
2768	3.03	4.46	F1M7L6	Secretogranin III, isoform CRA_a	3	1.10	1.28	1.27	0.70	1.12	1.00779346	0.442
2769	3.03	16.20	P0C5H9	Mesencephalic astrocyte-derived neurotrophic factor	2	0.82	0.63	0.82	0.36	0.77	0.95913115	0.339
2770	3.02	12.53	Q794E4	Heterogeneous nuclear ribonucleoprotein F	8	0.82	1.15	1.25	0.99	1.07	1.00109577	0.93
2771	3.02	3.60	D4A1C0	Cyclin M1 (Predicted)	2	0.76	0.78	1.04	0.74	0.79	0.952638	0.0882
2772	3.02	15.09	O35165	Golgi SNAP receptor complex member 2	3	0.82	0.92	0.88	0.87	0.95	0.98773778	0.742
2773	3.02	21.82	B2RYW7	RCG26543, isoform CRA_b	2	0.49	0.12	0.23	0.24	0.58	0.91319825	0.324
2774	3.01	8.49	D4A415	Family with sequence similarity 175, member B	3	0.95	0.98	0.95	0.95	0.91	1.07474917	0.341

2775	3	15.75	F1M8F2	Kinesin heavy chain isoform 5A	19	1.08	1.12	0.98	0.99	1.02	0.99261076	0.788
2776	3	6.30	Q5U1W6	MIC	2	0.53	0.93	0.63	0.74	0.62	1.04978945	0.169
2777	2.99	7.59	A0A0G2JT64	Uncharacterized protein	3	0.57	0.85	0.61	1.16	0.87	1.07474917	0.0675
2778	2.99	2.24	Q9Z136	Hamartin	2	1.10	1.11	0.98	0.86	0.99	0.94363445	0.0378
2779	2.99	5.43	A0A096MJZ2	RCG21429, isoform CRA_b	2	0.95	1.03	0.95	1.14	0.95	0.99185422	0.895
2780	2.99	6.42	Q5XI79	Protein arginine methyltransferase	3	1.05	0.86	0.95	0.91	0.81	0.95217589	0.137
2781	2.99	11.68	D3ZL85	NDUFAF7, mitochondrial	2	0.84	0.48	0.90	0.72	1.01	1.06341152	0.276
2782	2.99	3.52	A0A0G2JTF6	Cytochrome c heme lyase Thiamine-triphosphatase	1	0.92	1.07	0.78	0.51	0.92	0.75210188	0.00247
2783	2.97	4.41	A0A140TAF7	Protein	2	0.69	0.78	0.75	0.79	0.61	1.01698613	0.46
2784	2.97	2.45	D3Z9L5	WD repeat domain 11	3	0.95	0.76	1.17	0.89	0.89	0.97461207	0.314
2785	2.96	76.06	P01946	Hemoglobin subunit alpha-1/2	67	1.80	2.70	5.06	6.31	10.28	1.31950791	0.162
2786	2.96	17.50	P36506	Dual specificity mitogen-activated protein kinase kinase 2	11	1.18	1.00	1.25	1.12	1.04	0.96707521	0.318
2787	2.96	2.13	D3ZG78	Zinc finger ZZ-type and EF-hand domain-containing 1	5	1.07	0.94	0.89	0.96	0.95	1.0179735	0.587
2788	2.96	19.40	P04041	Glutathione peroxidase 1	3	0.46	0.72	0.62	0.66	0.72	1.0242018	0.416
2789	2.96	4.99	G3V9W5	Phosphatidylinositol 5-phosphate 4-kinase type-2 gamma	2	1.02	1.13	0.95	0.95	1.20	1.07162489	0.246
2790	2.95	10.55	P62813	Gamma-aminobutyric acid receptor subunit alpha-1	5	1.03	1.08	1.46	1.31	1.53	1.0387871	0.313
2791	2.95	16.54	P61972	Nuclear transport factor 2	2	1.09	1.32	1.19	0.98	1.14	0.91066983	0.0395
2792	2.94	7.16	A0A0G2K1W	RAB11 family-interacting protein 5	5	1.10	0.97	1.05	1.31	1.13	0.94842132	0.122
2793	2.94	8.65	O70352	CD82 antigen	2	1.10	1.02	1.06	1.49	1.33	1.04412903	0.199
2794	2.94	20.16	D4A1G1	Acylphosphatase	2	0.56	0.67	0.92	0.36	0.63	0.92210312	0.0748
2795	2.94	8.83	P04177	Tyrosine 3-monooxygenase	2	0.75	0.65	0.53	0.56	0.49	0.96781285	0.363
2796	2.93	10.26	Q4V898	RNA-binding motif protein, X chromosome	4	0.95	0.94	0.82	0.89	0.69	1.04964393	0.475
2797	2.93	5.34	P70604	Small conductance calcium-activated potassium channel protein 2	3	1.39	1.42	1.25	0.64	0.97	0.99151053	0.804
2798	2.93	3.05	F1LMI7	E3 ubiquitin-protein ligase RNF123	2	1.03	0.99	1.10	1.08	0.95	0.98084699	0.0424
2799	2.93	1.78	O35314	Secretogranin-1	2	1.41	1.24	1.12	1.04	1.34	0.93380939	0.00744
2800	2.91	3.15	D4A7I6	Uncharacterized protein	5	1.96	1.89	1.60	2.44	2.21	0.99534598	0.718
2801	2.91	29.41	P29418	ATP synthase subunit epsilon, mitochondrial	2	1.24	0.77	1.12	1.56	1.29	0.93978328	0.313
2802	2.91	8.44	G3V8Q1	Coatomer protein complex, subunit epsilon	2	0.90	1.01	1.02	0.93	1.01	1.11961289	0.00984
2803	2.91	19.39	D3ZTB5	S100 calcium-binding protein A13	2	2.03	1.47	6.25	1.05	3.13	1.22688498	0.299
2804	2.9	10.46	Q6AYC2	Immunity-related GTPase family M protein	3	0.73	1.43	0.37	0.21	1.06	1.08222465	0.0275
2805	2.89	11.55	Q62844	Tyrosine-protein kinase Fyn	6	1.03	1.10	1.10	1.15	1.05	1.0164928	0.589
2806	2.89	5.93	Q5XIP0	DnaJ (Hsp40) homolog, subfamily B, member 4	2	1.03	1.04	1.00	0.93	0.95	0.90563398	0.199
2807	2.89	8.11	D3ZIL6	Enoyl CoA hydratase domain-containing 2	2	0.74	0.84	0.79	0.70	0.78	0.88331505	0.0297
2808	2.89	13.73	F1LX20	TNF alpha-induced protein 8-like 3	2	1.47	1.47	0.72	0.78	0.45	1.02349212	0.627
2809	2.89	2.45	Q4FZS2	BUB3 mitotic checkpoint protein	2	0.95	0.98	1.01	1.02	0.93	1.02861279	0.34
2810	2.89	9.17	D4A2K5	Nuclear receptor interacting protein 3 (Predicted), isoform CRA_a	2	0.40	1.29	0.92	0.88	1.45	1.18099266	0.0852
2811	2.89	4.78	A0A0U1RRU	RCG59826	2	1.05	1.13	0.85	1.01	0.97	0.89688816	0.0516
2812	2.89	14.34	B2RYT7	Haloacid dehalogenase-like hydrolase domain containing 3	2	0.98	1.02	1.01	0.92	0.84	1.02704543	0.718
2813	2.88	18.50	D3ZDR2	Charged multivesicular body protein 6	3	0.81	0.69	0.83	0.85	0.85	1.06363267	0.0479
2814	2.87	2.02	A0A0G2JZH9	DExH-box helicase 57	2	0.84	0.90	0.88	0.90	0.86	1.0453601	0.164
2815	2.87	15.30	Q5RJK5	Chromobox 3	2	0.74	0.90	0.75	0.54	1.04	0.9802353	0.794
2816	2.87	3.95	P0CC10	Leucine-rich repeat-containing protein 4B	2	1.07	1.00	1.08	1.20	1.10	0.96687413	0.273
2817	2.87	4.45	F1M1A6	Similar to potassium channel tetramerisation domain-containing 12b	2	0.67	1.49	1.49	1.47	1.51	1.0115727	0.583
2818	2.86	26.11	P35286	Ras-related protein Rab-13	5	1.24	1.17	1.45	1.56	1.33	0.99932718	0.979
2819	2.86	3.41	F1M7U7	Tyrosine-protein kinase receptor TYR	3	1.00	0.97	0.96	0.95	0.92	0.91573369	0.214
2820	2.86	8.93	Q8K4F7	m7GpppX diphosphatase	2	1.08	1.10	1.01	0.92	0.95	1.09809281	0.0198
2821	2.86	5.31	Q99J82	Integrin-linked protein	3	0.88	1.10	1.07	1.12	0.94	1.07773315	0.215
2822	2.86	6.50	Q8CGU6	Nicastrin	3	0.74	0.55	0.71	0.74	0.81	1.0334725	0.512
2823	2.86	3.67	Q62902	Protein ERGIC-53	2	0.89	0.89	0.88	1.02	0.82	0.98931373	0.677

2824	2.86	5.10	Q5BJN3	Tia1 cytotoxic granule-associated RNA binding protein-like 1	2	1.03	1.20	1.14	1.08	1.20	1.12427892	0.00734
2825	2.86	20.24	P63090	Pleiotrophin	2	1.27	0.25	1.58	2.38	2.11	1.35472498	0.013
2826	2.85	1.67	D4A054	RAN-binding protein 2	4	0.97	0.99	0.98	0.97	0.95	1.05826431	0.0639
2827	2.85	5.16	A0A0G2K5G6	Magnesium transporter protein 1	2	1.33	1.08	1.34	1.15	1.28	1.03979563	0.385
2828	2.84	4.10	Q5XIS8	Mitochondrial dynamics protein MID51	2	0.83	0.84	0.99	0.87	0.96	1.06437018	0.00414
2829	2.83	25.00	P06302	Prothymosin alpha	2	1.14	1.51	1.28	0.95	1.12	1.21419488	0.0124
2830	2.82	5.94	D3ZMX6	Syntrophin, beta 2	3	0.90	0.91	0.94	0.77	0.94	0.99640903	0.887
2831	2.82	2.02	G3V7L1	Utrophin	6	0.95	0.94	1.03	0.98	1.04	1.07326029	0.314
2832	2.81	2.10	A0A0G2K0Q7	Myosin light chain kinase	4	1.51	3.28	1.01	1.45	1.58	1.07474917	0.098
2833	2.81	4.22	F1M7N2	Ectonucleoside triphosphate diphosphohydrolase 2	2	0.53	0.60	1.31	0.59	1.34	0.94809268	0.0668
2834	2.81	2.94	B4F7E8	Niban-like protein 1	2	1.69	1.89	2.11	2.25	1.47	1.12427892	0.0365
2835	2.8	17.76	Q9ESH6	Glutaredoxin-1	3	0.92	0.99	0.94	0.98	0.95	1.00947137	0.804
2836	2.8	4.02	A0A0G2K3S9	Solute carrier family 24 member 4	2	1.06	0.94	1.08	1.06	0.95	0.98071102	0.73
2837	2.79	13.16	Q71SY3	Translin	3	0.91	0.95	1.24	1.01	0.74	0.99144181	0.506
2838	2.79	2.00	D4AAH9	TBC1 domain family, member 23	2	1.04	1.26	1.09	1.19	1.04	0.9952563	0.907
2839	2.79	7.50	Q32PX2	Aminoacyl tRNA synthase complex-interacting multifunctional protein 2	2	0.82	0.74	1.02	1.03	0.96	0.9459265	0.0917
2840	2.79	10.36	Q5BK07	Coiled-coil domain-containing protein 43	2	0.35	0.13	0.13	0.34	0.18	1.08447741	0.0079
2841	2.79	8.59	Q6AY04	m-AAA protease-interacting protein 1, mitochondrial	2	0.95	1.00	0.90	1.00	0.95	1.08975211	0.00185
2842	2.79	5.51	F1LMK0	A kinase (PRKA) anchor protein 1, isoform CRA_a	2	5.86	2.11	3.28	3.77	2.27	1.12116608	0.0371
2843	2.78	2.45	A0A096MJV9	Phosphorylase b kinase regulatory subunit alpha, skeletal muscle isoform	3	1.11	0.70	0.88	0.60	0.84	0.97440943	0.0772
2844	2.78	5.07	G3V7N6	Membrane-bound	2	1.02	1.11	1.22	1.22	1.12	1.02925467	0.54
2845	2.78	8.43	O88794	Pyridoxine-5'-phosphate oxidase	2	0.90	0.82	0.93	0.94	0.90	1.07474917	0.0703
2846	2.77	3.10	D3ZRX7	Protocadherin 10	3	0.46	0.95	0.95	1.10	0.97	1.14710702	0.103
2847	2.76	2.76	F1M1B3	Uncharacterized protein	3	1.31	1.49	1.41	1.08	1.20	1.00835246	0.802
2848	2.76	8.94	A0A0G2JZB8	Neuronal membrane glycoprotein M6-b	4	1.28	0.06	1.34	0.77	1.41	1.14949485	0.36
2849	2.75	7.83	Q566R0	Acyl-coenzyme A thioesterase THEM4	1	1.21	1.66	0.92	0.94	0.68	0.96366259	0.577
2850	2.75	3.54	Q5PQJ7	Tubulin-specific chaperone cofactor E-like protein	1	1.27	1.11	1.07	0.89	1.06	0.98030324	0.666
2851	2.74	44.19	Q3B7V5	RAB2B, member RAS oncogene family	9	0.97	0.90	1.00	0.83	0.90	0.98357025	0.654
2852	2.74	5.89	Q6AYB4	Heat shock 70 kDa protein	2	1.01	0.89	1.03	1.31	0.79	1.12740041	0.0757
2853	2.74	16.95	P62775	Myotrophin	2	0.91	1.66	1.20	0.83	1.32	0.92787348	0.00848
2854	2.73	6.16	A0A0G2K9V4	Cytohesin-3	3	0.95	1.08	0.96	0.90	0.99	0.96006235	0.345
2855	2.73	2.85	Q5XI26	Signal transducer and activator of transcription	2	0.71	1.54	1.08	0.82	1.09	1.050663	0.087
2856	2.72	3.79	Q4KLI7	Splicing factor 3a, subunit 3	2	0.96	0.95	1.17	1.01	1.24	0.99347805	0.853
2857	2.72	3.08	M0RCT5	Echinoderm microtubule-associated protein-like 1	2	1.00	1.10	1.04	1.12	1.03	0.93776608	0.0336
2858	2.71	2.86	M0R7A6	Intersectin 2	4	0.86	1.22	0.97	1.27	1.14	1.0589247	0.00329
2859	2.71	4.75	Q8CHN6	Sphingosine-1-phosphate lyase 1	3	0.65	0.75	0.82	0.64	0.77	1.05241229	0.0852
2860	2.71	6.43	A0A0G2K590	Spastin	2	0.96	0.88	0.90	1.00	1.06	1.00393092	0.759
2861	2.7	4.87	F1M3L7	Epidermal growth factor receptor kinase substrate 8	3	0.98	1.16	1.06	1.08	1.11	0.99509764	0.46
2862	2.7	15.79	M0RAK2	RCG22622	2	1.28	0.45	0.65	0.86	1.17	1.07922824	0.0735
2863	2.7	8.83	P43138	DNA-(apurinic or apyrimidinic site) lyase	4	0.91	1.00	0.82	0.79	0.83	0.76524839	0.0341
2864	2.69	6.46	P47196	RAC-alpha serine/threonine-protein	4	0.99	0.96	1.04	1.05	1.13	0.99709302	0.935
2865	2.69	2.31	M0R965	Hypothetical protein L	2	0.94	0.94	0.94	0.96	0.96	1.04782661	0.129
2866	2.69	3.85	Q9ESQ7	PH and SEC7 domain-containing protein 1	1	1.01	0.88	0.89	0.99	0.98	1.04304399	0.632
2867	2.69	5.10	Q7TQ11	Aa1018	2	0.94	1.03	1.03	0.99	1.05	1.00377784	0.935
2868	2.68	3.27	A0A0H2UH90	von Willebrand factor A domain-containing protein	2	0.95	1.05	0.96	0.99	0.85	1.0535801	0.023
2869	2.66	11.15	D4A8N1	Dolichyl-phosphate mannosyltransferase subunit 1, catalytic	3	0.75	0.51	0.42	0.82	0.67	1.06017322	0.198
2870	2.66	7.61	Q99MB4	C	2	1.18	0.93	0.63	0.67	0.91	1.04753613	0.334
2871	2.66	3.13	D4A626	Calmin	2	1.31	1.25	0.77	0.73	1.47	1.02278294	0.405
2872	2.66	6.33	Q587K4	Leucine-rich repeat-containing protein 73	2	1.07	1.03	0.93	1.02	1.00	0.88945899	0.053
2873	2.65	7.47	Q64232	Very-long-chain enoyl-CoA reductase	3	0.99	1.00	0.98	1.06	1.18	0.98890237	0.64

2874	2.64	1.37	F1MAF8	Uncharacterized protein	2	0.88	0.81	0.77	0.80	0.76	0.96292811	0.0985
2875	2.64	1.81	E9PTY6	FRY microtubule-binding protein	4	0.94	0.53	0.33	0.47	0.74	1.04311629	0.00661
2876	2.63	7.96	D3ZVR7	Prostamide/prostaglandin F synthase	2	1.22	1.11	1.12	1.14	1.06	1.00961133	0.816
2877	2.62	2.47	D4A7D3	Similar to TANK-binding kinase 1	1	1.05	1.01	0.90	0.91	0.75	1.02115368	0.474
2878	2.62	9.23	A0A0G2JTP7	E3 ubiquitin-protein ligase RING2	2	0.83	0.74	0.82	0.86	0.79	0.99549778	0.846
2879	2.61	8.98	Q07014	Tyrosine-protein kinase Lyn	5	0.82	1.21	1.08	1.12	1.87	0.99683733	0.853
2880	2.61	14.56	P63138	Gamma-aminobutyric acid receptor subunit beta-2	5	1.12	1.00	0.87	0.93	1.00	0.92274249	0.0797
2881	2.61	6.80	Q5RK30	Ribosome maturation protein SBDS	2	0.96	0.84	0.85	0.88	0.95	1.05336104	0.0355
2882	2.61	20.00	Q3KRD8	Eukaryotic translation initiation factor 6	2	0.72	0.90	0.80	0.79	0.71	0.81733533	0.0449
2883	2.6	5.93	Q9JJH5	6-phosphofructo-2-kinase/fructose-2,6-bisphosphatase 2	2	1.33	1.11	1.13	1.07	1.06	1.04993499	0.478
2884	2.6	5.29	D4A0T8	Dehydrogenase/reductase 7	2	0.52	0.66	1.01	0.70	0.74	1.07624013	0.0353
2885	2.6	7.19	Q9R1T3	Cathepsin Z	2	1.01	1.04	2.13	1.71	1.56	0.95992926	0.451
2886	2.59	53.23	D3ZPP2	ADP-ribosylation factor-like GTPase 8A	10	1.05	1.03	1.08	1.04	1.06	0.98903947	0.794
2887	2.59	2.48	D3ZSZ6	Diacylglycerol kinase	2	1.07	0.76	0.99	0.92	0.90	1.01712712	0.739
2888	2.59	7.86	P14562	Lysosome-associated membrane glycoprotein 1	3	1.54	0.40	1.80	1.82	1.84	1.03519317	0.0506
2889	2.59	7.05	Q66H71	Serine/threonine-protein phosphatase CPPED1	2	1.03	0.96	1.22	0.72	0.98	0.92338231	0.611
2890	2.58	3.49	P51607	N-acylglucosamine 2-epimerase	1	1.21	1.06	1.07	0.90	0.95	1.10649735	0.0961
2891	2.57	3.18	G3V7I3	Cation-transporting ATPase	3	0.85	0.83	0.97	0.88	0.90	1.03440417	0.308
2892	2.57	2.65	D3ZBE5	Serine/threonine-protein kinase Nek7	1	0.83	0.94	0.89	1.05	1.01	1.01755022	0.814
2893	2.57	7.77	M0R776	Mitochondrial ribosomal protein S36	1	1.01	1.51	0.80	1.26	0.83	1.40249925	0.0795
2894	2.56	3.07	P20611	Lysosomal acid phosphatase	2	1.11	1.00	1.06	1.04	0.89	0.973127	0.239
2895	2.56	2.64	Q9EP80	PRKCA-binding protein	1	1.11	1.22	1.11	1.19	1.22	1.12427892	0.0989
2896	2.56	8.66	Q68G44	3-hydroxy-3-methylglutaryl-Coenzyme A synthase 2 (Mitochondrial)	3	1.16	1.17	1.05	1.16	1.09	0.99846932	0.932
2897	2.56	16.15	D4A3X3	ISG15 ubiquitin-like	2	1.32	1.64	2.25	1.51	1.22	0.91955005	0.0906
2898	2.56	7.28	F1M7K9	Elongator acetyltransferase complex subunit 4	2	0.79	0.95	0.92	0.82	0.82	0.94848706	0.538
2899	2.55	3.58	F1LXF1	BCR, RhoGEF and GTPase-activating protein	3	1.12	0.90	0.61	1.14	0.85	1.01980972	0.126
2900	2.55	5.03	Q9EQS4	Cystathionase (Cystathionine gamma-	2	null	null	null	null	null	1.01038141	0.787
2901	2.55	1.44	F1LS02	Nuclear pore complex protein Nup155	2	1.13	1.09	1.20	1.11	1.13	1.12661923	0.0129
2902	2.55	5.29	B0K030	DnaJ (Hsp40) homolog, subfamily B, member 1 (Predicted), isoform CRA_b	1	0.93	0.87	0.91	0.98	0.87	1.02989696	0.516
2903	2.54	1.74	D4A6P3	Shootin-1	1	1.09	1.07	1.16	0.92	0.85	1.14631219	0.0358
2904	2.54	12.56	Q6IN33	Calcipressin-1	2	1.24	0.98	1.10	1.05	1.04	1.02306655	0.725
2905	2.54	6.67	D3ZXB0	Paroxysmal nonkinesigenic dyskinesia	1	0.96	0.91	0.93	1.15	0.96	0.90877812	0.129
2906	2.53	2.94	D3ZEX7	Spire homolog 1 (Drosophila) (Predicted),	3	0.93	1.14	0.92	1.25	0.97	0.97779233	0.418
2907	2.53	6.22	Q6MG61	Chloride intracellular channel protein 1	2	0.52	2.15	3.08	1.58	1.21	1.01755022	0.86
2908	2.53	1.79	B2RYP3	Fam91a1 protein	1	0.83	1.07	0.82	0.79	1.02	1.0535801	0.202
2909	2.53	5.37	D4A7J8	PRP4 pre-mRNA processing factor 4 homolog	2	0.89	0.90	0.75	1.17	0.83	1.00599281	0.873
2910	2.52	7.59	A0A0G2K3L9	PTPRF-interacting protein alpha 1	8	1.11	0.93	0.93	0.63	1.06	1.00835246	0.648
2911	2.52	5.24	Q498T2	Chromatin target of PRMT1 protein	1	1.15	1.16	1.10	1.00	1.09	1.00442511	0.891
2912	2.51	1.59	A0A0G2JY30	Carboxypeptidase D	2	0.87	0.97	0.81	0.94	0.92	1.14631219	0.00694
2913	2.51	3.49	Q9Z1M9	Structural maintenance of chromosomes protein 1A	5	0.95	0.90	0.93	0.92	0.94	1.05401836	0.0167
2914	2.51	9.16	D3ZNY3	Methylmalonic aciduria (Cobalamin deficiency) cblA type (Predicted), isoform CRA_a	3	1.10	1.12	1.16	1.18	1.16	1.05826431	0.0371
2915	2.51	4.21	M0R7D1	La-related protein 7	2	1.02	0.96	0.84	1.07	0.88	1.01487356	0.15
2916	2.51	6.62	D3ZAQ0	FUN14 domain-containing	2	0.86	1.00	0.93	0.95	0.97	0.96446447	0.322
2917	2.51	8.54	D3ZKT8	HD domain containing 2 (Predicted), isoform CRA_b	1	1.15	1.07	1.09	0.77	0.82	0.95780243	0.502
2918	2.5	10.95	F1LZU7	Amyloid beta precursor protein-binding family B member 2	3	0.75	0.89	0.81	1.09	0.86	1.05943862	0.23

2919	2.49	1.57	A0A0G2K3N1	Golgi brefeldin A-resistant guanine nucleotide exchange factor 1	3	1.06	1.17	1.02	1.24	1.18	0.98220767	0.31
2920	2.49	2.66	A0A0G2JWH4	1-acyl-sn-glycerol-3-phosphate acyltransferase delta	1	0.95	0.90	0.95	0.95	0.97	1.01945635	0.796
2921	2.49	4.79	D3ZFF4	Lysocardiolipin acyltransferase 1	2	0.78	0.72	0.85	0.83	0.86	1.0012415	0.976
2922	2.48	3.23	Q4KLK7	N	1	0.70	0.79	0.63	0.27	0.36	1.05416449	0.0234
2923	2.47	5.07	Q4FZU2	Keratin, type II cytoskeletal 6A	3	1.16	1.20	0.97	1.21	1.26	1.96836804	0.08832
2924	2.47	6.15	G3V879	5-demethoxyubiquinone hydroxylase, mitochondrial	1	1.22	1.01	1.08	1.01	1.29	1.00256794	0.903
2925	2.47	2.90	D4A3M8	NCK adaptor protein 2	1	0.82	0.95	0.86	0.78	0.89	0.99061749	0.885
2926	2.46	32.58	D3ZW56	Neurofascin	37	1.01	1.10	0.96	1.10	1.10	1.03146867	0.326
2927	2.46	2.17	Q5EBC7	Rab GTPase-binding effector protein 2	1	1.09	1.07	1.07	0.90	0.98	1.02925467	0.12
2928	2.46	4.15	Q3SWS9	Janus kinase and microtubule-interacting protein 1	2	0.88	1.01	1.10	1.12	0.92	1.10419885	0.179
2929	2.46	3.61	Q5RKK3	Bifunctional epoxide hydrolase 2	1	0.76	0.61	0.44	0.70	0.69	1.06238008	0.276
2930	2.46	7.08	D3ZUB0	Reticulocalbin 1	2	0.96	0.96	0.98	0.95	0.98	0.98200345	0.827
2931	2.45	3.74	Q498T9	Volume-regulated anion channel subunit LRRC8C	2	1.11	0.79	0.99	1.12	0.83	1.03713234	0.0404
2932	2.45	4.98	D4A758	Leucine-rich repeat-containing 8 family, member B	3	1.19	1.10	1.11	1.13	1.17	1.00096394	0.969
2933	2.45	8.63	M0R5N4	Prefoldin subunit 4	1	1.05	1.20	1.10	0.89	0.79	0.97704709	0.103
2934	2.45	1.77	Q6AYM8	Glycosylphosphatidylinositol anchor attachment 1	1	1.11	0.99	0.97	1.06	1.00	1.01424065	0.674
2935	2.45	2.51	D3ZM09	Seryl-tRNA synthetase 2 (Predicted)	1	0.94	1.08	0.97	1.01	0.98	1.00586731	0.868
2936	2.45	3.69	G3V8I4	Syntaxin 4A (Placental), isoform CRA_a	1	1.60	1.01	1.36	1.64	1.05	0.96299486	0.295
2937	2.44	3.45	Q920F5	Malonyl-CoA decarboxylase,	2	1.13	0.90	1.02	0.95	1.07	0.93711629	0.00304
2938	2.44	2.41	D4A5B3	C2 calcium-dependent domain-containing 5	2	1.13	0.95	1.11	0.95	0.98	1.07549439	0.0601
2939	2.44	1.03	Q3ZB99	Tight junction protein 2	1	1.08	1.04	1.16	1.10	1.06	1.03154017	0.216
2940	2.44	38.30	P35467	Protein S100-A1	2	1.10	1.29	1.49	1.09	1.17	1.10956947	0.2
2941	2.44	7.33	Q4KM35	Proteasome subunit beta type-10	1	0.69	0.90	0.88	0.93	0.88	0.85381771	0.161
2942	2.44	6.47	Q63524	Transmembrane emp24 domain-containing protein 2	1	0.98	0.86	1.03	1.00	1.11	0.94756709	0.54
2943	2.43	9.18	A0A096MJE3	G1 to S phase transition 2	6	1.10	0.98	0.90	0.94	1.01	0.97670853	0.579
2944	2.43	4.15	Q4KLZ6	Triokinase/FMN cyclase	2	0.87	1.41	0.90	0.89	1.05	0.99116696	0.859
2945	2.43	2.13	D4ACD9	Protein tyrosine phosphatase domain containing 1 (Predicted), isoform CRA_a	1	0.98	0.85	0.90	0.81	0.91	0.9941807	0.819
2946	2.43	5.30	D3ZRE7	SWAP complex protein (Predicted), isoform CRA_b	3	0.91	0.98	1.09	0.99	0.89	1.05643207	0.122
2947	2.43	2.35	E9PU29	Ring finger protein 31	2	0.82	1.13	0.89	0.92	0.95	1.00272083	0.87
2948	2.43	4.40	A0A0H2UHR3	[3-methyl-2-oxobutanoate dehydrogenase [lipoamide]] kinase, mitochondrial	1	1.01	1.10	1.12	1.25	1.20	0.9843887	0.444
2949	2.43	9.29	D3ZZR9	Peptidylprolyl isomerase	1	1.13	1.08	0.96	1.15	1.17	0.98602764	0.554
2950	2.43	8.07	Q5I0L3	Tyrosine--tRNA ligase, mitochondrial	3	0.81	1.74	0.78	1.24	0.70	1.02818509	0.256
2951	2.43	5.35	Q05BA4	Myadm protein	1	1.25	1.20	1.14	1.26	1.25	1.00345784	0.96
2952	2.43	3.59	Q68FW4	Syntaxin-18	1	0.81	0.61	0.80	0.64	0.72	0.94258851	0.278
2953	2.42	5.18	F1LWT1	Calcium-dependent secretion activator 2	8	1.66	1.61	1.12	1.28	1.57	1.0118532	0.112
2954	2.42	6.19	D4A604	Phosphatidylinositol glycan anchor biosynthesis, class T	3	1.03	0.99	0.89	1.07	0.90	1.03039679	0.334
2955	2.42	1.39	D4A0F3		1	0.86	0.98	0.47	1.17	1.07	1.00933144	0.722
2956	2.42	1.11	P97570	85/88 kDa calcium-independent phospholipase A2	1	0.90	0.89	0.82	0.77	0.94	1.02711662	0.0046
2957	2.42	3.66	P48303	Sphingosine 1-phosphate receptor 1	1	0.89	0.89	0.82	0.94	0.99	1.01663373	0.513
2958	2.42	2.71	A0A140UHX9	CCZ1 homolog B, vacuolar protein-trafficking and biogenesis-associated	1	1.05	0.93	0.94	0.75	1.01	0.99075483	0.535
2959	2.42	7.86	M0R6L8	DnaJ heat shock protein family (Hsp40) member	1	0.97	0.79	0.83	1.05	0.87	0.92594602	0.462
2960	2.41	1.31	Q6P730	Disabled homolog 2-interacting protein	1	1.27	1.12	1.02	0.69	1.04	1.01614057	0.397
2961	2.41	1.49	D3ZF03	Apoptosis-inducing factor, mitochondria-associated 3	1	0.96	0.96	0.95	1.03	0.88	1.02861279	0.0733

2962	2.41	21.30	P21571	ATP synthase-coupling factor 6, mitochondrial	1	0.96	1.84	1.74	0.64	1.74	0.94973702	0.616
2963	2.41	4.38	B2GUX5	5'-nucleotidase	1	1.02	1.09	1.13	1.16	1.03	1.02548046	0.576
2964	2.41	9.06	Q5RK09	Eukaryotic translation initiation factor 3 subunit G	2	1.08	0.98	1.01	0.96	0.99	1.00046105	0.972
2965	2.4	3.88	O54701	Sodium/potassium/calcium exchanger 2	2	0.81	1.72	1.20	1.71	2.13	1.13288389	0.0448
2966	2.4	5.02	Q3KR55	RCG60540, isoform CRA_a	1	1.00	1.24	1.41	1.61	1.16	1.16070391	0.11
2967	2.39	2.10	G3V8Q8	RCG40648, isoform CRA_b	2	1.05	0.97	0.91	1.02	0.92	1.01059154	0.451
2968	2.39	4.86	Q9Z142	Transmembrane protein 33	1	0.87	0.82	0.82	0.85	0.94	1.04362253	0.333
2969	2.39	4.31	Q7TPI7	Ac2-269	1	1.19	0.91	0.90	0.99	1.10	1.04775398	0.529
2970	2.38	4.69	D3ZLA3	Copine 3	3	0.93	0.90	0.91	0.88	0.88	1.0324701	0.56
2971	2.38	7.16	D4A264	Zinc-binding alcohol dehydrogenase, domain-containing 2	2	0.86	0.82	0.97	1.04	0.95	1.09961615	0.003
2972	2.38	2.53	Q7TP58	Phosphoglycerate mutase	1	1.08	0.96	1.02	1.04	1.14	0.99267957	0.747
2973	2.38	7.41	P61150	Fibroblast growth factor 12	2	0.81	0.79	0.86	0.92	0.90	0.97718255	0.602
2974	2.38	8.05	Q4KM45	UPF0687 protein C20orf27 homolog	1	1.37	0.48	0.84	0.60	0.70	1.01017133	0.881
2975	2.38	20.88	D3ZBJ0	LSM5 homolog, U6 small nuclear RNA and mRNA degradation-associated	1	null	null	null	null	null	0.92530443	0.147
2976	2.37	2.47	G3V9R9	Afamin	1	0.76	0.36	0.14	0.42	0.36	1.00786332	0.722
2977	2.36	13.12	Q505J6	Mitochondrial glutamate carrier 2	5	1.03	0.90	1.14	1.17	1.06	0.91636865	0.175
2978	2.36	1.60	B5DF57	Erythrocyte membrane protein band 4.2	1	0.91	0.97	1.01	0.93	1.00	1.01374866	0.191
2979	2.35	4.65	Q9Z1W6	Protein LYRIC	1	1.53	1.61	1.46	0.04	1.19	1.0132569	0.697
2980	2.35	4.08	D4AE79	Charged multivesicular body protein 1A	1	1.00	1.01	0.92	1.07	1.11	1.07051128	0.0576
2981	2.35	3.09	D3ZUM4	Beta-galactosidase	2	0.86	0.87	0.75	0.99	0.79	0.92658806	0.00576
2982	2.34	3.20	Q4V7E8	Leucine-rich repeat flightless-interacting protein	1	1.94	0.98	0.93	0.60	1.10	1.04145464	0.204
2983	2.34	2.89	G3V628	Hypothetical protein MGC:15854	1	0.88	1.18	1.00	0.89	0.97	0.91256549	0.356
2984	2.33	2.05	Q4KLH7	RAD21 cohesin complex component	1	0.98	0.93	1.18	0.91	0.95	1.15588671	0.0886
2985	2.33	2.49	D4A6D8	Leucine-rich repeat transmembrane neuronal protein 1	1	1.01	1.21	1.02	1.07	1.01	0.94993454	0.0171
2986	2.33	3.31	A0A0G2JX45	Arginyl-tRNA--protein transferase 1	1	0.98	0.95	1.02	1.06	0.95	0.99829631	0.94
2987	2.32	1.25	A0A0G2JTA7	RAS protein activator-like 2	1	1.14	1.19	1.06	1.10	1.11	1.0132569	0.344
2988	2.32	1.55	P32198	Carnitine	1	0.90	1.01	1.06	1.15	1.04	1.04275483	0.414
2989	2.32	1.20	E9PU01	Chromodomain helicase DNA-binding protein 4	2	1.00	0.90	0.91	0.97	0.99	1.01628145	0.623
2990	2.32	39.39	D4A6X4	Acylphosphatase	3	1.20	1.27	1.19	1.20	1.21	1.01178307	0.715
2991	2.31	10.22	B1WC28	Core histone macro-H2A	4	0.94	0.87	0.99	0.91	0.84	0.93044966	0.0152
2992	2.31	6.24	Q80ZG1	Synembryn-A	2	0.99	1.07	1.02	1.08	1.00	0.99212926	0.848
2993	2.31	1.85	F1M8P2	Zyg-11 family member B, cell cycle regulator	1	0.73	0.65	0.74	0.79	0.74	0.99219803	0.66
2994	2.31	1.24	O88763	Phosphatidylinositol 3-kinase catalytic subunit type	1	1.00	0.98	1.06	1.01	1.11	0.97238531	0.329
2995	2.31	2.88	D3ZDI7	Protein phosphatase 2, regulatory subunit B', alpha	1	0.94	0.95	0.94	0.95	0.86	1.03182621	0.54
2996	2.31	1.98	B1WC06	M	1	0.83	1.02	0.85	1.04	0.82	0.85737604	0.419
2997	2.3	5.32	Q5D006	Ubiquitin carboxyl-terminal hydrolase 11	5	1.09	1.07	1.14	1.26	1.02	0.97258753	0.109
2998	2.3	2.76	G3V614	KIF1-binding protein	2	2.54	2.94	2.38	1.47	1.66	0.94265385	0.0871
2999	2.3	2.28	Q6AYT5	Protein-glutamate	1	1.11	1.18	1.01	1.14	1.14	1.01543648	0.542
3000	2.3	1.91	A0A0G2K249	Serine/threonine-protein kinase PRP4 homolog	1	1.56	1.19	2.00	0.96	0.97	1.03914718	0.0337
3001	2.3	3.11	D3ZEI6	Nuclear receptor coactivator 5	1	0.79	0.61	1.22	0.63	0.88	0.92980494	0.331
3002	2.3	7.14	P27274	CD59 glycoprotein	1	0.93	1.25	1.20	0.76	0.85	1.01508462	0.645
3003	2.3	25.00	P62329	Thymosin beta-4	1	1.07	1.79	1.49	0.14	2.15	0.85500218	0.618
3004	2.3	9.85	D4A4Q4		1	0.96	0.61	0.85	0.96	0.90	0.91636865	0.428
3005	2.29	1.69	F1LR42	RUN and FYVE domain-containing 1	1	1.13	0.96	0.67	0.95	0.90	0.99274838	0.689
3006	2.29	2.92	Q56AP7	Protein cereblon	1	0.97	1.25	1.08	1.14	1.04	0.9534968	0.15
3007	2.29	7.05	D3ZUI1	Methylthioribulose-1-phosphate dehydratase	1	1.25	0.92	1.09	0.94	0.99	1.05628563	0.0473
3008	2.29	6.86	A0A0G2KA48	39S ribosomal protein L37, mitochondrial	2	0.23	0.95	0.36	0.18	0.91	0.9665391	0.255
3009	2.29	3.92	Q6QI88	LRRG00120	1	1.19	0.36	1.33	0.74	1.22	0.98677974	0.605
3010	2.29	1.34	F1M9F9	Jouberin	1	0.93	1.02	0.97	0.87	0.92	0.99624329	0.858
3011	2.29	10.88	Q9WU49	Calcium-regulated heat stable protein 1	1	0.94	0.95	1.02	1.06	0.97	1.01790294	0.678
3012	2.28	2.76	D3ZF21	GPRIN family member 3	2	0.93	1.10	0.82	1.20	1.08	0.99603614	0.921
3013	2.28	1.23	A0A0G2K7Q7	Insulin-degrading enzyme	1	0.77	0.94	1.13	0.95	1.04	1.16231411	0.0288

3014	2.28	2.38	D4A5B8	Leucine rich repeat and fibronectin type III domain containing 1 (Predicted)	1	1.09	1.06	1.02	1.17	1.16	0.90437938	0.195
3015	2.28	7.64	Q6R5J6	NYGGF5	2	1.01	1.00	0.96	0.95	0.96	0.99102956	0.925
3016	2.28	7.33	Q5XIR9	Ubiquitin-associated domain-containing protein 1	2	0.98	1.09	0.95	1.09	1.00	1.07251662	0.0158
3017	2.28	3.64	Q3MQ06	Autophagy protein 5	1	0.82	0.93	0.93	1.00	0.90	0.86753869	0.0224
3018	2.27	80.90	Q3KRE8	Tubulin beta-2B chain	357	1.36	2.03	1.58	1.67	1.17	0.97191362	0.728
3019	2.27	27.15	A0A0G2K9N3	DEAD (Asp-Glu-Ala-Asp) box polypeptide 3	16	1.02	1.05	0.88	0.67	1.09	1.02469887	0.0261
3020	2.27	4.36	Q6AY19	Atypical kinase C	1	1.00	0.79	0.76	0.97	0.69	0.9208257	0.278
3021	2.27	5.17	P02680	Fibrinogen gamma chain	2	1.20	1.09	0.58	0.43	0.81	0.97609942	0.222
3022	2.27	8.37	D3ZTK0	Putative uncharacterized protein RGD1306256_predicted	1	1.01	1.02	0.80	1.02	0.96	1.09657159	0.00985
3023	2.27	3.45	G3V7P3	39S ribosomal protein L3, mitochondrial	1	0.96	1.02	1.12	1.05	1.09	1.06341152	0.0632
3024	2.27	5.99	P63047	Sulfotransferase 4A1	3	1.08	0.94	1.01	0.94	0.97	1.02370498	0.0386
3025	2.26	11.96	B5DEX3	Copine 8	7	1.51	1.18	0.82	0.73	1.26	0.80051481	0.0529
3026	2.26	1.70	G3V8Y5	DNA-directed RNA polymerase subunit beta	2	0.67	0.67	0.69	0.61	0.54	1.12038921	0.0843
3027	2.26	2.29	E9PTN4	SRSF protein kinase 1	1	0.90	1.11	1.11	0.94	0.86	1.09885422	0.102
3028	2.26	3.13	P97888	Serine/threonine-protein phosphatase 2A 55 kDa regulatory subunit B gamma isoform	1	0.68	0.74	0.49	1.14	0.90	1.06614229	0.00706
3029	2.26	3.42	O08623	Sequestosome-1	1	0.72	0.90	1.09	0.93	0.84	1.05416449	0.0694
3030	2.26	8.28	P47967	Galectin-5	1	0.80	1.03	0.79	0.84	0.93	0.84440089	0.0161
3031	2.25	1.20	A0A0G2K9C8	Phosphorylase kinase regulatory subunit beta	2	1.28	1.20	1.21	1.05	1.06	0.96533393	0.104
3032	2.25	3.78	A0A0G2KB61	Protein phosphatase 1, regulatory subunit 13B	4	1.15	0.95	0.95	0.96	0.92	0.97969189	0.306
3033	2.25	5.36	D3ZFY0	Selenophosphate synthetase	1	0.76	0.76	0.70	0.92	0.78	0.96593633	0.505
3034	2.25	2.30	Q5XIC2	Evolutionarily conserved signaling intermediate in Toll pathway, mitochondrial	1	0.84	0.91	0.81	0.79	0.81	1.00842235	0.777
3035	2.25	10.42	A0A0G2K2P3	TNF alpha-induced protein	1	1.24	1.42	0.89	1.22	1.03	1.02868409	0.596
3036	2.25	5.71	F1M9G8	Histidine triad nucleotide-binding protein 3	1	1.01	0.98	1.07	1.04	1.08	1.06739932	0.133
3037	2.24	6.70	A0A1B0GWN	Non-specific serine/threonine protein	4	0.72	0.75	0.66	1.05	0.72	0.97569355	0.318
3038	2.24	2.88	A0A0G2KAE2	Tumor suppressor candidate	1	1.04	0.88	0.76	1.08	0.89	1.0662901	0.517
3039	2.24	9.85	Q68FY1	Nucleoporin NUP53	2	1.39	0.85	0.79	1.53	0.96	1.0387151	0.471
3040	2.23	5.55	Q9ERB4	Versican core protein (Fragments)	16	0.92	1.17	1.72	0.94	2.00	1.01607014	0.709
3041	2.23	12.15	A0A0G2K7M2	RAD23 homolog A, nucleotide excision repair protein	3	1.09	1.15	0.99	1.02	1.05	1.08748839	0.309
3042	2.23	3.34	Q0D2L2	Mitochondrial ribosomal protein S22	1	1.15	1.11	1.10	0.94	1.07	1.02462784	0.716
3043	2.23	8.83	A0A0G2K1S9	CCM2-scaffolding protein	2	0.74	0.87	0.86	0.69	0.92	1.05760434	0.0691
3044	2.23	1.97	P46933	Amyloid beta A4 precursor protein-binding family B member 1	1	0.96	0.48	0.49	0.65	0.85	1.00954135	0.8
3045	2.22	5.74	Q642E5	Diphosphomevalonate decarboxylase	2	0.81	0.79	0.80	0.74	0.95	1.01628145	0.793
3046	2.22	2.15	Q9WTT7	Basic leucine zipper and W2 domain-containing protein 2	1	0.90	0.84	0.82	0.95	0.86	1.00996129	0.696
3047	2.22	12.09	A0A0G2KAA1	NADH:ubiquinone oxidoreductase subunit A3	1	0.82	1.17	1.00	0.67	0.65	0.99736951	0.93
3048	2.22	1.30	F1LR02	Collagen type XVIII alpha 1 chain	1	0.90	0.75	0.79	0.94	0.90	0.98931373	0.815
3049	2.22	4.83	D3ZC34	Ankyrin repeat and MYND domain containing 2 (Predicted)	1	1.01	1.05	1.51	1.08	1.29	1.0224994	0.366
3050	2.22	6.25	B5DFJ1	NFKB inhibitor interacting Ras-like 1	1	0.94	1.19	0.83	0.99	0.87	1.1455179	0.0331
3051	2.22	3.98	F1LVV4	Regulator of chromosome condensation 2	1	1.12	0.81	1.01	1.16	0.88	1.00541422	0.945
3052	2.22	7.10	D3ZY47	Similar to RIKEN cDNA 2310022B05	2	1.26	0.94	1.03	0.93	1.14	1.02505406	0.418
3053	2.22	4.15	D3ZZI0	NIPA-like domain-containing 3	2	1.04	0.93	0.84	0.97	0.85	1.11651296	0.0791
3054	2.22	19.47	P61805	Dolichyl-diphosphooligosaccharide--protein glycosyltransferase subunit DAD1	2	0.67	0.89	1.06	0.59	0.81	0.80385099	0.0905
3055	2.21	2.03	A0A0G2QC5(	Coronin	1	0.96	1.14	0.99	1.03	1.07	0.95554783	0.124
3056	2.21	14.29	D4ACN8	Plasminogen receptor (KT)	2	1.02	1.18	1.08	1.24	1.05	1.02654723	0.305
3057	2.21	16.16	G3V946	AIP1, isoform CRA_a	1	0.60	1.38	0.73	0.38	0.89	1.01269519	0.89

3058	2.21	3.60	Q5HZE0	Mitochondrial basic amino acids transporter	1	1.02	0.94	0.97	1.09	0.88	1.01705662	0.613
3059	2.21	10.00	D3Z9J8	Spermidine/spermine N1-acetyl transferase 2 (Predicted), isoform CRA_a	1	1.39	0.90	0.98	1.46	1.18	1.13918338	0.0659
3060	2.2	10.41	A0A0H2UHM	Vacuolar protein sorting 28 (Yeast) (Predicted), isoform CRA_c	3	1.08	0.87	0.95	1.09	1.00	0.91193317	0.26
3061	2.2	1.49	D3ZDD7	Spermatid perinuclear RNA binding protein, isoform CRA_a	1	1.05	1.00	1.05	1.34	1.10	1.08899702	0.0894
3062	2.2	2.68	Q925Q9	SH3 domain-containing kinase-binding protein 1	1	0.99	1.18	1.01	0.98	1.07	0.93426259	0.0221
3063	2.2	10.31	G3V721	WW domain binding protein 2, isoform CRA_b	4	1.74	1.53	1.26	1.24	1.45	0.92466328	0.0242
3064	2.2	4.60	B5DEK0	Regulation of nuclear pre-mRNA domain containing	1	0.59	1.01	0.48	0.34	0.37	1.01171294	0.822
3065	2.2	4.38	F7FNS3	Derlin	1	1.02	0.98	0.92	1.11	1.07	1.01839695	0.549
3066	2.2	3.29	Q5XIJ6	BRISC and BRCA1-A complex member 1	1	1.03	1.29	0.69	0.93	0.85	0.79996013	0.0321
3067	2.19	2.65	A0A0G2JW51	Putative ATP-dependent RNA helicase DHX30	3	0.94	0.86	0.99	1.04	0.95	1.00106108	0.976
3068	2.19	1.40	Q9QWG5	Type II inositol 3,4-bisphosphate 4-phosphatase	1	1.46	1.00	1.19	0.74	1.09	1.07698638	0.131
3069	2.19	1.70	Q924K2	FAS-associated factor 1	1	0.98	1.05	0.95	0.90	0.95	1.01213379	0.706
3070	2.19	5.47	P48508	Glutamate--cysteine ligase regulatory subunit	2	1.04	0.94	0.86	1.00	0.99	1.04224901	0.417
3071	2.19	5.17	D3ZZR3	Cathepsin S	1	1.26	0.88	1.46	1.02	1.15	1.09202055	0.248
3072	2.19	15.63	B2RZD1	Protein transport protein Sec61 subunit beta	1	1.01	1.07	1.26	1.17	1.32	1.06230644	0.252
3073	2.18	5.91	F7EY92	Methyl-CpG binding domain protein 3 (Predicted), isoform CRA_c	1	1.33	1.07	1.19	1.15	1.60	0.91827616	0.579
3074	2.18	4.41	Q4KM77	Etoposide-induced protein 2.4 homolog	2	0.67	0.82	0.80	1.04	0.85	1.01733865	0.779
3075	2.18	3.12	D3ZUD8	Transmembrane 9 superfamily member	2	0.64	0.18	0.75	0.74	0.88	0.95601158	0.363
3076	2.17	4.62	A0A0G2JWD2	Fragile X mental retardation syndrome-related protein 1	2	1.00	0.86	0.97	0.97	0.95	0.98910803	0.749
3077	2.17	8.28	D3ZBX9	Coiled-coil domain-containing 92	3	1.12	1.14	0.57	0.77	0.93	1.00123456	0.974
3078	2.17	0.91	D3ZIL9	DIS3-like exonuclease 2	1	0.95	1.15	1.05	1.01	0.92	1.01304622	0.73
3079	2.17	0.96	D3Z881	TBC1 domain family, member 4	1	1.36	0.86	0.92	0.93	1.08	1.02697424	0.422
3080	2.17	7.32	P50411	Protein phosphatase inhibitor 2	1	1.01	1.07	1.08	1.02	1.05	1.04030027	0.397
3081	2.17	9.02	D3ZX38	Prefoldin 1 (Predicted)	1	1.08	1.13	0.96	0.59	0.83	1.06947296	0.251
3082	2.17	13.48	Q9R1T1	Barrier-to-autointegration factor	1	1.02	0.77	0.80	0.80	0.89	1.1655412	0.116
3083	2.17	1.60	D3ZWJ9	Family with sequence similarity 234, member B	1	1.27	1.19	1.06	1.38	1.33	0.88576752	0.0115
3084	2.17	11.78	Q5XI39	Crystallin zeta-like 1	2	1.51	1.13	0.60	0.58	0.96	0.99925168	0.981
3085	2.17	3.73	A0A0G2KA82	Synaptophysin-like 1	1	0.90	0.36	0.99	0.90	0.67	1.01874996	0.162
3086	2.16	2.98	P47853	Biglycan	1	0.74	1.11	0.96	1.07	1.21	0.99504936	0.742
3087	2.16	1.53	A0A0G2K4R1	Protein phosphatase 1, regulatory subunit 12C	1	1.41	1.46	1.47	1.38	1.33	0.9942565	0.905
3088	2.16	1.92	Q1AAU6	Arf-GAP with SH3 domain, ANK repeat and PH domain-containing protein 1	2	1.16	0.79	0.82	0.85	0.65	1.04159902	0.0799
3089	2.16	2.56	D4A3V4	Ring finger protein 214	1	0.97	0.88	0.87	0.76	0.83	1.0597324	0.197
3090	2.16	1.83	D3ZX08	Alanine--tRNA ligase, mitochondrial	1	null	null	null	null	null	1.05350707	0.0429
3091	2.16	3.10	Q920A6	Retinoid-inducible serine carboxypeptidase	1	1.02	0.97	1.08	1.17	1.45	1.02214509	0.63
3092	2.16	1.13	F1LPG9	Uncharacterized protein	1	0.67	0.95	0.65	0.42	0.43	0.94664801	0.0298
3093	2.16	1.50	D3ZE55	Protocadherin-8	1	0.49	0.66	0.70	0.62	0.71	1.04593993	0.195
3094	2.16	6.35	M0R4S2	Apolipoprotein D	1	1.01	1.10	1.79	1.06	1.32	1.04131027	0.346
3095	2.16	5.82	D3ZXH7	Aly/REF export factor	1	0.95	1.02	1.01	0.98	0.96	0.95893172	0.135
3096	2.16	20.29	B0BN94	Protein FAM136A	2	1.05	1.18	1.09	1.21	1.06	0.93491039	0.273
3097	2.15	1.28	D3ZL11	Rabenosyn, RAB effector	1	0.86	0.43	0.95	0.70	0.41	1.04615745	0.0341
3098	2.15	5.91	Q09426	2-hydroxyacylsphingosine 1-beta-galactosyltransferase	2	1.09	1.39	0.96	1.02	1.41	0.95939711	0.281
3099	2.15	22.11	D3ZG88	Mammary tumor virus receptor 2, isoform CRA_a	2	0.84	0.33	0.60	0.41	0.72	1.00154691	0.913
3100	2.15	1.49	Q3B7D6	RCG39552, isoform CRA_a	1	0.99	0.95	0.96	0.97	1.03	1.07400447	0.109
3101	2.15	13.98	G3V960	Guanidinoacetate N-methyltransferase	2	1.01	1.15	1.13	0.97	1.12	0.9859593	0.572
3102	2.15	13.58	M0R5M7	Transcription factor BTF3	1	0.95	0.90	0.86	0.96	1.09	1.0546761	0.161
3103	2.14	31.25	P0C0S7	Histone H2A.Z	5	1.15	0.79	0.70	0.28	0.34	0.74898047	0.0167

3104	2.14	2.60	A0A096P6M3	Regulating synaptic membrane exocytosis protein 2	2	0.92	0.95	0.82	0.90	0.91	1.04572246	0.0579
3105	2.14	2.96	Q5PQL5	Phosphatidylserine synthase	1	1.09	1.03	1.14	1.21	1.06	1.00223443	0.907
3106	2.14	7.11	D3ZJB8	Ariadne RBR E3 ubiquitin protein ligase 2	3	0.98	1.11	0.80	1.03	0.55	1.03325762	0.226
3107	2.14	0.76	A0A0G2JVN4	RCG25629, isoform CRA_a	1	1.39	0.95	1.32	1.53	1.04	0.99634687	0.848
3108	2.14	5.43	Q5RK19	Vacuolar-sorting protein SNF8	1	0.92	1.10	1.18	0.76	0.90	1.01783239	0.627
3109	2.14	19.79	D4A478	Nucleoside-triphosphatase, cancer-related	2	1.07	1.06	1.10	1.03	0.92	0.99178547	0.84
3110	2.14	5.52	D3ZZ46	Ceroid-lipofuscinosis, neuronal 6, late infantile, variant	1	1.10	0.90	0.92	1.10	0.99	1.07400447	0.122
3111	2.14	3.20	M0RAG0	Putative uncharacterized protein RGD1310680_predicted	1	0.83	0.89	0.70	0.84	0.92	0.98513954	0.662
3112	2.13	0.93	D3ZYD7	Coiled coil domain-containing 88A	1	1.49	1.57	1.47	1.71	1.20	1.0222868	0.297
3113	2.13	2.23	A0A0G2JUA6	WW domain-binding protein 11-like 1	1	0.99	0.95	0.99	0.90	0.84	1.06717739	0.131
3114	2.13	1.14	Q925N6	Immunoglobulin superfamily member 1	1	0.63	0.75	0.89	0.69	0.95	1.0706597	0.162
3115	2.13	0.83	D4A3T0	S	1	1.22	1.08	0.96	1.21	0.89	1.03677296	0.135
3116	2.13	5.75	B2RYN3	Eukaryotic translation elongation factor 1 epsilon 1	1	1.18	1.15	0.90	1.15	1.07	1.02235766	0.416
3117	2.13	2.59	F1MA52	Nuclear RNA export factor	1	1.27	1.15	1.10	0.57	1.33	1.04369488	0.0139
3118	2.13	12.42	B1WC56	H/ACA ribonucleoprotein complex subunit 2	1	1.38	1.10	1.24	1.01	1.11	1.15588671	0.0559
3119	2.12	16.90	Q63910	Alpha globin	4	1.42	1.69	1.50	1.27	1.38	1.25092908	0.235
3120	2.12	6.63	F1SW39	PC4 and SFRS1 interacting protein 1	4	1.20	1.11	1.04	0.76	1.09	1.12194348	0.0263
3121	2.12	27.73	B2RZB7	Small nuclear ribonucleoprotein D1	3	0.92	0.65	1.03	0.69	0.82	0.91003882	0.195
3122	2.12	2.06	D4A2Z6	SEC63 homolog, protein translocation regulator	1	0.88	0.72	0.77	0.95	0.88	1.01297601	0.583
3123	2.12	4.09	A0A0G2K0E5	Brain-enriched guanylate kinase-associated protein	2	1.08	1.07	0.90	0.94	1.04	0.9996618	0.994
3124	2.12	3.02	Q5M9G9	Protein TBRG4	1	0.98	0.42	0.59	0.23	0.49	0.98418402	0.00875
3125	2.12	2.53	D4ACK7	Cyclin and CBS domain divalent metal cation transport mediator 3	1	0.93	1.03	1.11	0.90	0.96	1.03655739	0.183
3126	2.12	1.36	A0A140TAC8	E3 ubiquitin-protein ligase MIB2	1	0.42	0.64	0.50	1.11	0.66	1.02612039	0.124
3127	2.11	5.72	F1M9C9	Histidyl-tRNA synthetase 2	3	0.81	1.05	0.76	0.51	1.08	0.91763988	0.086
3128	2.11	2.28	D3ZHF8	Translation factor GUF1, mitochondrial	1	1.05	0.76	0.60	0.72	0.79	1.03032537	0.53
3129	2.11	3.25	Q8VHT6	Arsenite methyltransferase	1	0.79	0.90	0.90	0.82	0.97	1.01945635	0.838
3130	2.11	2.37	F1M3A4	Uncharacterized protein	1	0.91	1.11	1.24	1.24	0.94	1.04942568	0.0996
3131	2.11	7.52	Q5BJN7	40S ribosomal protein S30	1	1.00	1.14	1.06	1.00	1.02	1.00516337	0.965
3132	2.11	2.53	D3ZEH9	E3 ubiquitin-protein ligase MGRN1	1	2.07	1.43	1.51	1.14	1.00	1.01438126	0.869
3133	2.11	1.55	D4A896	ATP-binding cassette, subfamily G (WHITE), member 3-like 3	1	0.80	0.95	1.32	1.14	0.84	0.94121747	0.111
3134	2.11	3.68	A0A140TA89	Glycine amidinotransferase, mitochondrial	1	2.83	0.34	1.84	2.03	1.10	1.01066159	0.797
3135	2.11	4.53	Q5XIF0	Testis expressed 264	1	1.04	1.01	0.95	1.03	1.06	1.01607014	0.613
3136	2.11	9.55	G3V9Y9	Adaptor-related protein complex 3, sigma 1 subunit	1	0.94	1.05	1.09	0.97	1.00	1.16958766	0.0228
3137	2.1	5.80	G3V8P4	RCG50226, isoform CRA_a	10	0.49	0.89	0.91	0.80	0.68	1.01332714	0.761
3138	2.1	2.35	Q3MIB4	Lon protease homolog 2, peroxisomal	2	0.91	1.27	0.96	0.03	0.50	1.07251662	0.0179
3139	2.1	2.23	M0R6T4	MMS19 homolog, cytosolic iron-sulfur assembly component	1	0.83	0.38	0.64	0.42	0.52	1.00379175	0.863
3140	2.1	1.51	D3ZYR4	Semaphorin 4D	1	0.83	0.86	0.92	0.91	0.78	1.03849913	0.167
3141	2.1	3.01	D4A6D7	Tetratricopeptide repeat protein 19, mitochondrial	1	0.68	1.21	0.77	1.08	1.06	1.04181564	0.227
3142	2.1	5.94	A0A140TAD1	ADP-sugar pyrophosphatase	1	1.12	1.15	1.11	1.18	1.11	1.03569557	0.0109
3143	2.1	0.90	D4A899	Vacuolar protein sorting	2	1.02	0.96	0.94	1.05	0.87	1.07922824	0.0714
3144	2.1	3.64	G3V7Z8	Poly(A) binding protein, nuclear 1, isoform CRA_a	1	0.74	0.95	0.62	0.36	0.42	0.77808518	0.258
3145	2.1	4.94	Q6AYQ4	Transmembrane protein 109	1	0.79	0.67	0.72	0.86	0.86	1.09733194	0.227
3146	2.1	0.91	Q99J86	Attractin	1	0.96	0.61	0.80	0.72	0.74	0.95966315	0.24
3147	2.1	19.75	G3V8W9	Similar to CG12279-PA	2	0.71	0.47	0.69	0.60	0.77	0.95727146	0.0613
3148	2.1	5.86	Q4QQS6	ALG5, dolichyl-phosphate beta-glucosyltransferase	1	0.93	0.86	0.96	0.79	0.85	1.07110506	0.388
3149	2.09	4.28	F1LNG5	Phosphatidylinositol 3-kinase regulatory subunit	3	1.06	1.07	1.16	1.11	1.09	1.10496449	0.0101

3150	2.09	5.69	Q08415	Kynurenine--oxoglutarate transaminase 1,	2	0.52	0.71	0.61	0.39	0.36	0.98910803	0.84
3151	2.09	2.37	F1M855	Glutamate receptor ionotropic, kainate 2	2	0.99	1.21	0.82	1.00	0.78	1.01959768	0.431
3152	2.09	2.19	D3Z8L5	Pumilio RNA-binding family member 1	2	0.83	1.20	0.86	0.92	0.94	1.04181564	0.173
3153	2.09	2.29	P40190	Interleukin-6 receptor subunit beta	1	0.93	1.15	0.70	0.88	0.96	1.22688498	0.017
3154	2.09	5.21	D3ZI68	PRP31 pre-mRNA processing factor 31 homolog (Yeast) (Predicted)	1	0.78	0.95	1.06	1.20	1.02	0.95627668	0.273
3155	2.09	2.03	F1M589	Promyelocytic leukemia	1	0.86	1.11	0.82	0.72	0.73	0.88148016	0.0184
3156	2.09	3.91	Q5PPG2	Legumain	1	0.88	0.84	1.29	0.89	0.86	1.07922824	0.135
3157	2.09	2.78	P20417	Tyrosine-protein phosphatase non-receptor	1	1.25	1.08	0.82	1.07	0.89	1.02754388	0.503
3158	2.09	3.33	F1LQC1	Acyl-coenzyme A oxidase	1	1.14	1.13	0.93	1.07	1.01	1.17283495	0.0204
3159	2.09	4.42	F1LNX7	Tumor-suppressing subtransferable candidate 1	1	0.80	0.89	1.00	0.84	0.96	1.59770383	0.03106
3160	2.09	6.99	O88350	Putative hydrolase RBBP9	1	1.07	0.95	1.11	1.31	1.10	1.15188764	0.173
3161	2.08	3.18	P11530	Dystrophin	10	0.86	0.92	0.90	0.95	0.90	1.0285415	0.169
3162	2.08	2.35	F1M8K0	Dystroglycan 1	2	1.08	1.01	0.98	1.18	1.16	0.96513322	0.342
3163	2.08	3.02	D3Z8D8	SLIT and NTRK-like family, member 1	2	0.90	0.93	0.91	0.79	0.95	1.00052138	0.982
3164	2.08	2.07	D3ZL30	Microtubule-associated serine/threonine kinase 3	2	1.46	1.89	0.77	0.95	0.86	1.01564766	0.699
3165	2.08	1.93	F1MAH5	Protein phosphatase 6, regulatory subunit 3	1	0.77	1.06	0.95	0.82	0.84	1.04521519	0.00661
3166	2.08	4.85	Q5XI97	Alanyl-tRNA editing protein Aarsd1	1	0.55	0.30	0.30	0.34	0.76	1.01487356	0.53
3167	2.08	7.12	A0A0G2K5F1	LRP16 protein	1	0.79	1.06	0.93	1.07	0.84	0.91193317	0.0673
3168	2.08	1.56	P52631	Signal transducer and activator of transcription 3	1	1.79	1.32	1.57	1.26	1.75	1.08372597	0.189
3169	2.08	3.07	F1M9W9	Similar to TRS85 homolog (Predicted)	4	1.05	0.86	0.82	0.88	0.86	0.98268436	0.249
3170	2.08	7.06	Q4FZX7	Signal recognition particle receptor subunit beta	1	0.90	0.97	0.98	0.92	1.13	1.00807292	0.821
3171	2.08	15.90	G3V7P6	Nudix (Nucleoside diphosphate linked moiety X)-type motif 16 (Predicted), isoform CRA_a	2	1.07	0.63	0.75	0.65	0.64	0.94317671	0.0865
3172	2.08	2.77	A0A0A0MY0(	Short/branched chain-specific acyl-CoA dehydrogenase,	1	1.02	1.03	1.14	1.32	1.07	1.00499617	0.968
3173	2.08	4.62	Q5BK18	Cytosolic Fe-S cluster assembly factor NARFL	1	8.32	1.53	6.67	7.94	4.74	1.08598186	0.0537
3174	2.08	0.89	F1LZ38	Teneurin transmembrane protein 4	2	1.31	0.74	0.48	0.67	0.65	1.02790006	0.295
3175	2.08	0.60	A0A0U1RRZ.	Protein PRRC2A	1	1.04	1.02	0.97	1.11	1.11	1.01698613	0.631
3176	2.08	9.45	Q6MGC4	H2-K region expressed gene 2, rat orthologue	1	1.18	1.18	1.15	1.04	1.08	1.04405665	0.0067
3177	2.08	1.05	F1MAL5	Insulin receptor substrate 2	1	1.91	0.91	0.26	1.11	0.43	0.97969189	0.675
3178	2.08	3.20	Q80VL0	Interactor protein for cytohesin exchange factors	1	1.45	1.66	1.15	1.41	1.17	1.0522664	0.322
3179	2.08	6.36	Q07984	Translocon-associated protein subunit delta	1	1.03	1.06	0.97	1.06	0.97	0.87115419	0.0415
3180	2.07	33.58	P63041	Complexin-1	8	1.07	1.19	1.21	1.01	1.07	0.74277646	0.212
3181	2.07	9.63	Q00954	Sodium channel subunit beta-1	2	0.90	0.76	0.98	0.91	0.85	0.91891288	0.318
3182	2.07	12.87	D3ZFR9	Retinol dehydrogenase 13	3	1.13	0.95	0.78	0.85	0.96	1.00685086	0.795
3183	2.07	2.10	D3ZJW3	PPFIA-binding protein 1	2	0.85	0.89	1.00	1.05	0.93	1.02320839	0.17
3184	2.07	1.73	D4A2A3	Putative uncharacterized protein RGD1565522_predicted	1	1.05	0.82	0.28	0.96	1.15	0.99954055	0.983
3185	2.07	1.78	B4F7E7	Membrane palmitoylated protein 5	1	0.78	0.61	0.76	1.26	0.81	1.00202604	0.93
3186	2.07	3.77	A0A0H2UHS	RCG47431	1	1.18	1.02	0.98	1.04	1.02	0.98071102	0.495
3187	2.07	3.93	D4ADP1	Protein FAM162A	1	1.31	0.81	1.21	1.08	1.09	0.97097092	0.62
3188	2.07	4.11	Q63424	Solute carrier family 15 member 2	3	1.57	1.31	2.29	1.96	1.69	1.05591961	0.101
3189	2.07	8.04	Q9Z0G8	WAS/WASL-interacting protein family member 3	2	0.76	1.05	0.95	1.15	1.19	0.98814866	0.595
3190	2.07	2.78	Q505J7	Galactose-3-	1	0.94	0.87	0.90	0.87	0.89	0.8913105	0.013
3191	2.07	2.69	D3ZYY0	Short chain dehydrogenase/reductase family 39U, member 1	1	1.07	1.04	0.96	1.00	0.88	1.05299603	0.671
3192	2.07	6.32	D4A8N2	Ferredoxin 1-like	1	0.99	1.03	1.01	0.86	0.94	0.87721355	0.0919
3193	2.06	10.17	D4A5V8	Mitogen-activated protein kinase	5	1.14	1.19	0.86	1.16	1.05	1.17202228	0.0432

3194	2.06	19.79	M0R5T4	Ras-related C3 botulinum toxin substrate 3 (rho family, small GTP-binding protein Rac3)	5	0.72	0.86	0.41	0.69	0.52	0.94402698	0.69
3195	2.06	5.38	Q9QYJ4	ATP-binding cassette sub-family B member 9	4	0.82	0.69	0.93	1.03	0.80	1.10343374	0.17
3196	2.06	1.33	F1LSH0	Symplekin	1	0.91	0.30	0.72	0.23	0.74	1.08297505	0.0557
3197	2.06	2.84	A0A0G2K326	Regulator of G-protein signaling 12, isoform	1	0.97	1.15	1.08	1.26	1.04	1.01811463	0.583
3198	2.06	6.23	D3ZL21	Similar to TP53-regulating kinase (p53-related protein kinase) (Nori-2)	1	0.75	0.56	0.63	0.71	0.65	1.32776516	0.0495
3199	2.06	2.79	Q2A121	Alpha-ketoglutarate-dependent dioxygenase FT	1	0.69	0.52	0.52	0.82	0.76	1.04768136	0.164
3200	2.06	4.44	Q9ET55	Nocturnin	1	1.03	1.11	1.11	0.96	0.89	0.94520555	0.191
3201	2.06	2.45	A0A0G2JUM3	Retinitis pigmentosa 2 (X-linked recessive)	1	0.79	0.99	1.04	0.95	0.93	1.10037861	0.227
3202	2.06	1.19	D4ADX8	Ras association (RalGDS/AF-6) and pleckstrin homology	1	1.09	1.00	0.86	1.08	0.83	1.00926148	0.598
3203	2.06	2.34	A0A096MJJ6	Acyl-CoA-binding domain-containing protein 5	1	1.01	0.94	0.93	0.89	0.94	0.8882268	0.0131
3204	2.06	1.41	A0A140UHW0	tRNA (guanine(26)-N(2))-dimethyltransferase	1	1.20	1.04	1.12	1.25	1.08	0.91509917	0.138
3205	2.06	7.18	B0K014	D-tyrosyl-tRNA(Tyr) deacylase	2	1.29	1.18	1.05	0.95	0.98	1.00279034	0.932
3206	2.06	13.10	P62845	40S ribosomal protein S15	1	0.96	1.13	1.12	1.09	1.13	1.03857111	0.271
3207	2.06	2.73	P36202	PDZ and LIM domain protein 4	1	0.82	0.99	1.22	1.00	1.03	0.98917659	0.866
3208	2.06	5.16	G3V6K3	Exosome component 7	1	0.60	0.58	0.59	0.58	0.53	0.95402568	0.328
3209	2.05	45.90	D3ZK56	RAP2C, member of RAS oncogene family	12	1.11	0.46	0.49	0.41	0.36	1.00105414	0.977
3210	2.05	15.09	P35289	Ras-related protein Rab-15	4	0.92	1.00	1.01	0.96	1.08	1.01642235	0.208
3211	2.05	2.38	F1LPS6	Interferon-induced protein with tetratricopeptide repeats 1	1	0.90	1.98	0.84	0.97	1.19	1.0108017	0.573
3212	2.05	1.90	A0A0G2K2V3	Ubiquitin-specific peptidase 31	2	0.88	0.91	1.02	1.27	1.12	1.02405983	0.407
3213	2.05	2.03	F1LVV3	RAN-binding protein 9	1	1.14	0.95	0.93	0.99	1.07	1.07177346	0.0225
3214	2.05	1.83	P25977	Nucleolar transcription factor 1	1	1.03	1.20	0.75	0.52	0.78	1.06799138	0.0284
3215	2.05	2.27	A0A0G2K1P1	Myotubularin-related protein 7	1	1.07	1.01	1.03	0.91	0.99	1.01417035	0.613
3216	2.05	2.02	G3V7V0	SHC-transforming protein 3	1	1.07	1.05	0.87	1.03	0.95	1.09885422	0.0823
3217	2.05	2.76	A0A0G2KAX1	Similar to hypothetical protein FLJ10925	1	1.04	0.90	0.82	0.89	0.79	0.98986247	0.878
3218	2.05	0.85	A0A0G2JWP8	DNA topoisomerase 2	1	1.02	0.99	0.97	1.02	0.95	1.03025395	0.161
3219	2.05	4.91	D4ABK7	Heterogeneous nuclear ribonucleoprotein H3	2	0.71	1.38	1.00	0.98	0.74	0.77003717	0.14
3220	2.05	1.55	A0A0G2K5K1	Anoctamin	1	0.89	1.15	1.10	0.90	0.83	0.9843887	0.616
3221	2.05	4.96	Q5I0K8	28S ribosomal protein S7, mitochondrial	1	0.84	0.94	0.90	0.90	0.91	0.9840476	0.524
3222	2.05	2.01	O88764	Death-associated protein kinase 3	1	0.86	0.93	0.86	0.67	0.85	0.94828985	0.0594
3223	2.05	3.80	Q91ZW6	Trimethyllysine dioxygenase, mitochondrial	1	0.97	1.01	0.93	1.09	0.99	1.0588513	0.382
3224	2.05	8.66	D4A8F2	Ras suppressor protein 1	1	0.86	1.41	1.49	1.56	1.60	1.06962123	0.41
3225	2.05	2.36	D3ZPL1	Cleavage and polyadenylation specific factor 6, 68kDa (Predicted), isoform CRA_b	1	0.92	0.94	1.00	0.98	0.94	1.02391787	0.452
3226	2.05	6.90	Q9JHB5	Translin-associated protein	1	0.98	0.83	0.84	0.66	0.47	0.99846932	0.962
3227	2.05	10.42	G3V878	Signal peptidase complex catalytic subunit SEC11	1	1.61	0.94	0.81	1.00	0.98	1.01776184	0.54
3228	2.05	5.40	D4A1H7	Mitochondrial E3 ubiquitin protein ligase 1	1	1.96	1.58	1.00	1.92	0.86	0.91573369	0.0542
3229	2.05	3.03	Q4KM87	Actin-like 6A	1	0.83	0.90	1.10	1.13	0.95	0.91003882	0.267
3230	2.04	24.19	P01048	T-kininogen 1	9	1.61	1.46	0.96	1.09	0.95	0.82245007	0.243
3231	2.04	2.10	Q05030	Platelet-derived growth factor receptor beta	2	0.45	0.44	0.87	0.72	0.82	0.9393274	0.221
3232	2.04	0.82	G3V8G1	ATP/GTP binding protein 1 (Predicted), isoform CRA_a	1	0.97	1.27	1.14	1.09	1.25	1.00912158	0.555
3233	2.04	2.23	Q6AYI5	Leucine-rich repeat protein SH	1	2.25	2.05	1.57	2.38	1.87	1.00751408	0.778
3234	2.04	2.05	Q5U2N0	CTP synthase 2	1	0.81	0.74	1.03	0.93	0.87	0.99988356	0.997
3235	2.04	3.22	A0A0G2JYA2	ADP-ribosylation factor interacting protein 1, isoform CRA_b	1	0.95	0.93	0.73	0.87	0.86	1.01353787	0.497
3236	2.04	7.26	Q5M969	N-acylneuraminate-9-phosphatase	1	0.98	0.85	0.74	1.03	0.80	1.07474917	0.0414
3237	2.04	4.37	B2GV97		1	1.16	0.95	0.90	1.18	0.78	1.15749022	0.0923

3238	2.04	1.36	D4A5T6	Rho GTPase-activating protein 6	1	1.07	1.11	0.92	1.04	1.11	1.01712712	0.564
3239	2.04	3.40	Q6AYS6	Sorting nexin-17	1	0.78	0.77	0.96	0.80	0.86	1.00898169	0.767
3240	2.04	5.83	Q3ZAV2	Nuclease-sensitive element-binding protein 1	1	1.03	1.19	1.14	1.09	1.11	0.96366259	0.531
3241	2.04	3.34	D4ADT5	DEAD (Asp-Glu-Ala-Asp) box polypeptide 58 (Predicted)	1	1.02	0.73	0.72	1.50	0.79	1.06592062	0.154
3242	2.04	5.35	A0A0G2KA71	Nectin cell adhesion molecule 2	1	0.78	0.71	0.95	0.84	0.99	0.92402257	0.0687
3243	2.04	4.64	F1LQF5	Phosphoglycerate dehydrogenase like 1	1	1.34	0.92	1.13	0.77	0.93	0.93400359	0.101
3244	2.04	11.76	D4A7U6	LSM3 homolog, U6 small nuclear RNA and mRNA degradation-associated	1	1.15	0.95	1.15	0.92	0.94	0.98897092	0.814
3245	2.04	10.50	Q5M860	Rho GDP dissociation inhibitor beta	1	1.11	1.31	1.27	0.97	0.98	1.09050773	0.0549
3246	2.04	2.39	Q5XIA6	Sphingomyelin phosphodiesterase	1	1.03	1.20	0.95	0.99	1.04	1.0118532	0.823
3247	2.04	8.77	Q6P5P3	Tetratricopeptide repeat protein 9C	1	1.51	1.47	1.34	1.80	1.61	0.85145371	0.376
3248	2.04	9.39	A0A0G2K724	Malignant T-cell-amplified sequence	1	0.79	0.78	0.94	0.83	0.89	0.9989262	0.94
3249	2.04	3.06	P84025	Mothers against decapentaplegic homolog 3	1	0.95	0.83	0.86	0.84	0.83	0.90500646	0.0779
3250	2.03	13.95	Q5XIJ7	Calcium binding protein 39-like	5	0.57	0.66	0.70	1.06	1.24	1.04615745	0.0666
3251	2.03	2.35	Q6XDA1	Erythroid spectrin alpha	5	1.29	1.54	1.15	0.71	1.45	1.02413081	0.109
3252	2.03	10.87	B5DEZ6	Glucosamine-6-phosphate isomerase	3	0.93	0.97	1.10	0.95	0.95	0.90814842	0.0689
3253	2.03	2.49	F1LX81	Schlafen family member 5	2	1.15	0.92	0.78	1.21	1.06	1.21841026	0.252
3254	2.03	3.53	A0A096UWG	Diacylglycerol kinase	2	1.09	0.93	0.97	1.17	0.96	0.94756709	0.199
3255	2.03	1.10	A0A0G2K0E7	Ubiquitin protein ligase	1	0.86	1.04	0.69	0.71	0.91	1.02604926	0.375
3256	2.03	1.37	A0A0G2JZL6	Intraflagellar transport 122	2	0.86	0.99	1.01	0.90	0.89	1.01740917	0.495
3257	2.03	1.38	Q3KRD0	Aspartate--tRNA ligase, mitochondrial	1	0.89	0.83	0.79	0.93	0.86	1.04978945	0.225
3258	2.03	2.27	D3Z8X6	Deltex E3 ubiquitin ligase	1	0.21	0.51	0.72	0.05	0.36	1.05951205	0.101
3259	2.03	2.34	A1L1L2	Transmembrane protein 214	1	0.90	0.86	1.11	1.06	0.97	1.02633378	0.201
3260	2.03	3.37	D4A5K6	Zinc metallopeptidase	1	0.97	0.91	0.84	1.11	1.08	1.03161167	0.493
3261	2.03	5.37	Q6AYS8	Estradiol 17-beta-dehydrogenase 11	1	null	null	null	null	null	1.02790006	0.816
3262	2.03	4.24	A0A0H2UHT	Protein phosphatase 1G	2	0.90	0.92	0.85	0.73	0.77	0.96888678	0.392
3263	2.03	1.73	A0A0G2JYJ1	Growth factor receptor-bound protein 10	1	0.74	0.80	0.64	0.57	0.47	0.98568597	0.757
3264	2.03	3.36	Q02769	Squalene synthase	1	0.95	0.74	0.87	0.68	0.91	1.01045145	0.754
3265	2.03	6.77	Q63690	Apoptosis regulator BAX	1	0.92	0.89	1.29	1.00	0.94	1.06090833	0.331
3266	2.03	4.72	P13852	Major prion protein	2	1.26	0.06	0.99	1.10	1.27	1.04862584	0.738
3267	2.03	3.47	D3ZZM0	Solute carrier family 39 (Zinc transporter), member 14 (Predicted)	1	0.97	0.88	0.95	1.05	0.84	1.09885422	0.0522
3268	2.03	8.07	D3ZIP8	Endonuclease domain-containing 1	3	0.61	0.74	0.77	0.80	0.73	1.03576736	0.314
3269	2.03	7.89	M0R7S5	Uncharacterized protein	1	1.32	1.33	2.11	1.66	1.39	1.38991822	0.103
3270	2.03	3.88	D3ZI74	N-acetyltransferase 14	1	1.00	1.03	0.93	0.99	0.90	0.86453723	0.135
3271	2.03	0.88	Q4TU93	C-type mannose receptor 2	1	1.12	0.97	0.94	1.36	0.86	0.99994074	0.999
3272	2.03	5.01	D3ZAS9	DDRGK domain-containing	1	1.08	1.28	0.81	0.96	1.22	0.92146419	0.115
3273	2.03	8.21	D3ZRV0	DCN1-like protein	1	1.22	1.13	1.01	1.00	1.12	0.74898047	0.00385
3274	2.03	6.47	Q63327	Myelin-associated oligodendrocyte basic	1	1.11	1.08	1.00	1.07	1.18	0.93783108	0.446
3275	2.03	2.86	Q1HL14	Ceramide synthase 1	1	1.04	1.11	1.02	1.08	0.92	0.88392753	0.255
3276	2.03	14.41	P10818	Cytochrome c oxidase subunit 6A1, mitochondrial	1	1.71	1.06	1.18	0.90	1.09	0.94901316	0.526
3277	2.03	12.84	P00697	Lysozyme C-1	1	1.67	1.22	1.18	1.53	1.33	0.77754604	0.295
3278	2.02	11.40	P47861	Synaptotagmin-5	7	0.96	1.01	1.09	1.04	1.02	0.93044966	0.207
3279	2.02	16.20	P21818	Stathmin-2	4	0.81	0.92	0.78	0.83	1.10	1.03770761	0.4
3280	2.02	3.97	D3ZXB1	Mitogen-activated protein kinase kinase kinase kinase	2	1.09	1.26	1.02	0.96	1.20	0.97447697	0.0785
3281	2.02	0.98	A0A0G2K8V	C-reactive protein	1	1.61	1.15	1.16	1.09	1.19	1.04434617	0.00374
3282	2.02	3.76	G3V818	Alpha-parvin	1	0.93	1.04	1.10	1.06	1.08	1.22433639	0.1
3283	2.02	1.34	F1M6W2	Endoplasmic reticulum metallopeptidase 1	1	0.85	0.75	0.82	0.76	0.91	1.05797094	0.0441
3284	2.02	5.14	Q6AXS4	Renin receptor	1	1.67	0.95	1.20	1.22	1.29	0.92466328	0.466
3285	2.02	1.49	D3ZUV2	Mindbomb E3 ubiquitin protein ligase 1	1	1.19	1.22	1.17	1.54	1.24	1.0554074	0.0523
3286	2.02	5.96	D4A0M2	Nucleoredoxin	2	0.35	0.45	1.08	0.04	0.45	0.99590498	0.812
3287	2.02	2.01	Q71F54	E3 ubiquitin-protein ligase SH3RF1	1	0.47	0.79	0.64	0.63	0.78	1.03605458	0.448
3288	2.02	1.02	A0A0G2JWG	Alpha-mannosidase	1	1.92	1.43	1.22	1.10	1.19	1.00138032	0.965
3289	2.02	1.95	P98166	Very low-density lipoprotein receptor	1	0.99	0.79	0.84	1.22	1.39	1.10037861	0.121

3290	2.02	2.04	A0A0G2K6E5	Protein-serine/threonine kinase	1	0.95	0.79	0.71	0.68	0.89	1.02775757	0.383
3291	2.02	1.66	B2RYF6	Cleft lip and palate associated transmembrane	1	1.12	0.82	0.94	0.81	1.06	0.96132756	0.418
3292	2.02	1.49	F1LU97	SAM and SH3 domain-containing 1	1	0.81	0.86	0.77	0.77	1.01	1.03641371	0.443
3293	2.02	2.61	Q9ET64	Sphingomyelin phosphodiesterase 2	1	0.93	0.88	0.84	0.89	0.90	1.00540028	0.87
3294	2.02	3.80	D4A8V0	CUGBP, Elav-like family member 5	1	0.91	0.57	1.24	0.83	1.13	1.01346762	0.603
3295	2.02	1.38	D3ZAU7	Tau tubulin kinase 1	1	0.94	0.99	1.20	0.64	0.55	1.0367011	0.324
3296	2.02	1.36	D3ZR79	Transmembrane protein 245	1	1.16	1.10	1.03	0.97	1.08	1.04572246	0.49
3297	2.02	0.89	D3ZRF6	Patatin-like phospholipase domain-containing 6	1	0.53	0.60	0.89	0.43	0.56	0.94809268	0.0286
3298	2.02	5.15	D4ACS9	RCG27978	1	1.96	1.17	0.92	0.95	0.90	0.97637009	0.286
3299	2.02	4.50	P41243	Megakaryocyte-associated tyrosine-protein kinase	1	1.08	0.95	0.87	0.77	0.70	0.92787348	0.0472
3300	2.02	2.26	A0A0G2KB26	Limitrin, isoform CRA_b	1	3.84	4.02	1.37	0.27	2.31	0.93238649	0.0445
3301	2.02	1.65	D4A1U1	Zinc finger and BTB domain containing 20 (Predicted), isoform CRA_b	1	0.72	0.63	0.90	0.94	0.95	1.05562689	0.0253
3302	2.02	3.16	A0A0G2JZD9	G protein-activated inward rectifier potassium channel	1	0.98	0.49	0.80	0.97	0.49	0.89688816	0.371
3303	2.02	2.96	D4A030	Upstream-binding protein 1 (LBP-1a)	1	1.31	1.29	0.93	1.08	0.72	1.08899702	0.0266
3304	2.02	2.32	F1MAS1	CACN beta subunit-associated regulatory protein	1	1.18	1.05	1.03	0.81	0.90	0.86994735	0.0307
3305	2.02	10.00	A0A0G2JXL3	Mitochondrial ribosomal protein S18B (Fragment)	1	0.77	0.98	0.98	0.89	0.79	0.89937831	0.195
3306	2.02	5.15	B5DFN3	Ubiquinol-cytochrome-c reductase complex assembly factor 2	1	0.95	0.77	0.71	0.84	0.95	0.99640212	0.902
3307	2.02	1.32	A0A0G2JYI7	Ubiquitin-associated protein	1	0.90	1.03	0.73	0.85	0.65	1.01607014	0.612
3308	2.02	1.76	Q0D2L6	Ras-related GTP binding C	1	0.74	1.00	0.58	0.70	0.82	0.98924516	0.726
3309	2.02	4.52	F1LRJ0	Potassium channel modulatory factor 1	1	null	null	null	null	null	0.91066983	0.033
3310	2.02	4.52	G3V683	Hypothetical L	1	0.72	0.83	0.91	0.85	0.60	1.03562378	0.316
3311	2.02	2.64	G3V8P6	Zinc finger and BTB domain containing 7a	1	0.68	0.53	0.58	0.64	0.58	1.07698638	0.202
3312	2.02	12.82	D3ZF11	Hepatitis B virus x interacting protein (Predicted), isoform CRA_a	2	0.54	0.69	0.61	0.48	0.46	1.0561392	0.212
3313	2.02	7.65	Q9JLT6	BH3-interacting domain death agonist	1	0.98	0.93	0.94	0.99	0.90	1.04920748	0.14
3314	2.02	5.29	B3DMA0	Tumor protein p53-inducible protein 11	1	0.96	0.87	1.05	1.03	0.82	0.8532261	0.21
3315	2.01	12.40	F1LZW6	Solute carrier family 25 member 13	6	0.86	0.78	0.79	0.95	0.72	0.92980494	0.209
3316	2.01	7.74	A0A0G2JUZ5	Glycine decarboxylase	5	1.14	0.83	1.07	0.92	1.32	1.04405665	0.0812
3317	2.01	5.67	B2RYJ3	Cullin 4A	4	1.45	1.01	1.09	1.24	1.77	1.03404574	0.361
3318	2.01	8.12	B4F775	Golgi associated PDZ and coiled-coil motif containing	2	1.67	1.80	0.99	1.17	1.07	1.03075396	0.183
3319	2.01	26.92	Q04940	Neurogranin	3	0.85	1.51	0.95	1.71	1.56	0.63816438	0.01707
3320	2.01	9.38	Q62703	Reticulocalbin-2	3	1.14	1.10	1.16	1.18	1.04	1.18181155	0.0778
3321	2.01	3.41	D3ZAG3	Microtubule-associated protein 9	1	0.70	0.82	1.12	0.95	0.77	1.06223281	0.23
3322	2.01	1.96	M0R8J3	Junction mediating and regulatory protein, p53 cofactor	1	0.91	1.20	1.75	1.43	1.32	0.92530443	0.0565
3323	2.01	3.69	F7EUJ8	C1q and tumor necrosis factor-related protein 4	1	1.27	1.10	1.20	1.12	1.19	0.94796126	0.614
3324	2.01	4.14	B2RZA5	Prolyl-tRNA synthetase-associated domain-containing 1	1	0.60	0.80	0.74	0.60	0.78	1.01143247	0.728
3325	2.01	11.22	P01041	Cystatin-B	2	1.79	1.14	1.98	1.09	1.82	1.07162489	0.479
3326	2.01	1.91	F1M8U2	Actin-binding LIM protein family, member 3	1	0.65	1.12	1.33	0.97	0.89	1.00737442	0.765
3327	2.01	4.64	D3ZCU8	RAB33A, member RAS oncogene family	1	0.90	0.90	0.92	1.04	0.87	1.03792342	0.358
3328	2.01	1.45	D3ZBW3	Transmembrane protein 201	1	0.83	1.06	0.87	0.85	0.96	0.92851685	0.203
3329	2.01	5.56	D4A1V7	M	1	1.22	1.18	1.13	1.11	1.22	1.03821123	0.358
3330	2.01	1.66	A0A0G2K3C0	Protein strawberry notch homolog 1	1	0.94	0.95	0.82	0.74	0.87	1.05980585	0.16
3331	2.01	2.33	D3ZHY0	Serine/threonine/tyrosine kinase 1	1	null	null	null	null	null	1.01501426	0.615
3332	2.01	4.46	M0R6J0	Mitochondrial ribosomal protein L39	1	0.40	0.37	0.46	0.33	0.28	1.00268608	0.948
3333	2.01	3.53	D4AE17	Uncharacterized protein	1	0.93	0.91	0.91	0.88	0.79	1.09202055	0.0179
3334	2.01	3.16	Q9QZA6	CD151 antigen	1	1.57	0.48	1.24	1.32	1.37	1.00842235	0.759
3335	2.01	6.53	P31720	Complement C1q subcomponent subunit A	1	0.82	0.95	0.73	0.68	0.55	0.98602764	0.661

3336	2.01	10.46	D4ACG8	Fucose mutarotase	1	0.80	1.19	0.61	1.27	0.71	1.11419365	0.0134
3337	2.01	1.82	B1WC18	Podocalyxin-like 2	1	0.74	0.54	0.64	0.77	0.45	0.8863817	0.1
3338	2.01	0.83	P49793	Nuclear pore complex protein Nup98-Nup96	1	9.46	6.61	2.86	6.49	10.00	1.06319041	0.191
3339	2.01	2.50	D3ZXT1	Leucine rich repeat containing 4C (Predicted), isoform CRA_a	1	1.08	0.69	0.96	0.94	0.85	0.96875248	0.158
3340	2.01	3.51	Q5XI74	Endonuclease/exonuclease/ phosphatase family domain-containing protein 1	1	1.06	1.31	0.97	1.38	0.85	0.81000347	0.203
3341	2.01	8.82	Q497C3	UPF0585 protein C16orf13 homolog	1	0.89	0.86	0.92	1.04	0.76	0.981255	0.708
3342	2.01	17.65	Q5BJP3	Ubiquitin-fold modifier 1	1	1.13	1.18	1.06	1.10	1.08	1.00968131	0.827
3343	2.01	1.57	D4A731	Ankyrin repeat and IBR domain-containing 1	1	0.65	0.82	0.77	0.55	0.57	0.97380175	0.257
3344	2.01	3.38	D3ZJG3	Prolyl 4-hydroxylase, transmembrane	1	1.28	1.14	1.13	1.09	0.92	1.02271205	0.236
3345	2.01	3.00	M0RAS4	Immunoglobin superfamily, member 21	1	1.15	1.12	0.90	1.07	0.87	0.86753869	0.26
3346	2.01	2.52	D3ZXL1	Ariadne RBR E3 ubiquitin protein ligase 1	1	0.93	0.98	1.00	1.15	0.96	1.03893111	0.527
3347	2.01	2.49	A0A0G2K2F4	REVERSED Serine and arginine-rich-splicing factor 12	1	null	null	null	null	null	null	null
3348	2.01	4.72	D3ZWE1	Aspartate beta-hydroxylase domain-containing 1	1	1.27	0.77	1.11	1.25	0.71	1.01557726	0.732
3349	2.01	4.10	D3ZE85	D	1	0.72	0.78	0.83	0.93	0.71	1.0087719	0.874
3350	2.01	4.59	Q641Z8	Peflin	2	0.91	0.86	0.93	0.69	0.77	1.02484093	0.294
3351	2.01	8.94	Q08326	Guanine nucleotide exchange factor MSS4	1	0.81	0.99	0.86	1.17	0.79	1.11187916	0.186
3352	2.01	2.11	D3ZKX8	Family with sequence similarity 169, member A	1	0.93	0.95	1.09	0.94	0.91	1.01255481	0.826
3353	2.01	4.29	Q769K2	N-acyl-phosphatidylethanolamine-hydrolyzing phospholipase	1	0.93	0.64	1.09	0.52	0.83	0.82817066	0.179
3354	2.01	5.84	Q4KLG9	AN1-type zinc finger protein 2B	1	0.99	0.90	0.86	1.03	0.92	0.98842267	0.813
3355	2.01	5.88	D3ZW38	Exosome component 6	1	0.92	0.99	0.94	0.95	0.94	0.87903956	0.193
3356	2.01	3.50	Q5RKJ2	Cytochrome b reductase 1	1	1.07	0.95	1.42	0.65	1.12	0.98037119	0.846
3357	2.01	9.61	D4A7Q6	Putative uncharacterized protein RGD1564862_predicted	1	1.04	0.64	0.92	1.18	0.53	1.04906204	0.713
3358	2.01	15.15	A0A0U1RRQ	NADH:ubiquinone oxidoreductase subunit A1	2	0.96	1.01	0.90	0.94	0.86	0.85856544	0.0346
3359	2.01	14.47	P0C0A9	Small VCP/p97-interacting protein	1	0.91	1.15	0.93	1.10	0.99	0.88576752	0.207
3360	2	71.41	Q6IRK8	Spectrin alpha chain, non-erythrocytic 1	285	0.95	1.07	1.11	1.05	0.96	0.93044966	0.0487
3361	2	79.93	G3V7C6	Tubulin beta chain	347	0.52	1.94	2.07	1.50	1.94	0.98057508	0.82
3362	2	84.70	P68370	Tubulin alpha-1A chain	314	0.63	0.58	0.10	0.30	0.43	0.98411581	0.702
3363	2	82.18	Q6AYZ1	Tubulin alpha-1C chain	280	1.14	0.91	0.82	0.91	1.05	0.973127	0.462
3364	2	64.86	F1LZI1	Uncharacterized protein	157	null	null	null	null	null	1.01522535	0.739
3365	2	73.03	A0A0G2JSH5	Serum albumin	59	0.04	0.71	0.51	0.71	0.61	1.43993132	0.0233
3366	2	82.13	P63259	Actin, cytoplasmic 2	259	null	null	null	null	null	1.1011416	0.0056
3367	2	49.88	G3V6Y6	Alpha-1,4 glucan phosphorylase	62	1.41	1.45	0.72	0.75	0.71	0.89502507	0.0632
3368	2	46.70	M0RAQ6	Hexokinase-1	63	0.86	1.45	1.16	1.39	1.09	0.96046171	0.305
3369	2	56.50	P50137	Transketolase	41	0.74	0.74	0.86	0.56	0.51	1.03972356	0.342
3370	2	61.26	G3V8Q2	Alpha-internexin	37	0.88	1.08	1.05	0.50	1.38	1.03504967	0.73
3371	2	23.78	Q5SGE0	Leucine-rich PPR motif-containing protein, mitochondrial	26	1.19	1.06	1.32	0.35	1.45	1.00331178	0.896
3372	2	29.95	Q05140	Clathrin coat assembly protein AP180	33	0.99	1.24	1.19	1.26	1.27	0.96439762	0.154
3373	2	30.80	P14882	Propionyl-CoA carboxylase alpha chain, mitochondrial	20	null	null	null	null	null	0.97962398	0.262
3374	2	21.51	A0A0G2K3V4		19	0.50	0.35	0.61	0.61	0.65	1.01339738	0.173
3375	2	44.14	E9PTV9	Glyceraldehyde-3-phosphate dehydrogenase	49	1.15	1.24	0.84	1.02	0.86	1.08748839	0.0612
3376	2	35.86	P07633	Propionyl-CoA carboxylase beta chain, mitochondrial	16	0.65	0.59	0.58	0.56	0.72	0.92018765	0.0567
3377	2	30.65	P97710	Tyrosine-protein phosphatase non-receptor	16	2.19	1.37	1.09	1.01	0.83	0.95647555	0.147
3378	2	75.80	A0A0G2K1L	Brain acid soluble protein 1	19	1.09	1.37	1.67	1.22	1.80	0.98064305	0.643
3379	2	43.38	Q63942	GTP-binding protein Rab-	20	0.86	0.69	0.86	0.81	0.93	0.99027423	0.879
3380	2	31.42	A0A0H2UHX	Reticulon	14	1.47	1.21	1.27	0.89	1.19	0.99862159	0.961
3381	2	18.65	P10688	1-phosphatidylinositol 4,5-bisphosphate phosphodiesterase delta-1	10	0.90	0.59	0.79	0.80	0.75	1.05168307	0.0428
3382	2	35.75	P70550	Ras-related protein Rab-8B	11	0.98	0.98	0.90	0.92	0.92	0.99789505	0.937

3383	2	37.82	B2RYP0	Ras homolog family member C	14	1.17	0.90	1.54	0.95	1.11	1.00174131	0.985
3384	2	47.57	P63025	Vesicle-associated membrane protein 3	9	1.45	0.99	1.13	0.96	1.13	0.86094919	0.106
3385	2	8.56	P84060	Dystrobrevin beta	6	0.91	0.93	0.80	0.93	0.90	0.89502507	0.0226
3386	2	35.38	P0CC09	Histone H2A type 2-A	8	1.46	1.14	0.68	0.21	0.29	0.51121427	0.01788
3387	2	10.14	A0A140TAI1	Ubiquilin 1, isoform CRA_a	8	1.05	0.95	0.86	0.81	0.84	1.07997656	0.0293
3388	2	13.07	Q3MIF0	Ribulose-5-phosphate-3-epimerase	3	0.74	0.62	0.63	0.56	0.59	1.01227411	0.441
3389	2	19.12	Q5BJU0	RCG40097	6	1.01	0.82	0.89	0.97	0.86	0.99901622	0.984
3390	2	17.07	Q9JJL4	Rho-related GTP-binding protein RhoQ	3	1.22	0.87	1.33	0.50	1.00	1.14631219	0.261
3391	2	12.67	F1M1H2	Uncharacterized protein	3	1.31	0.95	1.37	1.25	1.32	1.08673486	0.0692
3392	2	3.27	Q68FT3	Pyridine nucleotide-disulfide oxidoreductase domain-containing protein 2	2	0.90	1.03	1.03	1.03	1.46	0.90814842	0.0115
3393	2	4.48	D4A6L1	TAP binding protein-like (Predicted)	2	0.80	0.53	1.09	0.54	1.17	1.10190512	0.242
3394	2	3.28	D4AE03	Endo-beta-N-acetylglucosaminidase	2	1.31	1.06	0.78	0.70	0.85	0.89316585	0.0251
3395	2	8.00	E9PU44	SWI/SNF-related matrix-associated actin-dependent regulator of chromatin subfamily E member 1	3	1.45	0.95	1.29	0.92	1.14	0.93452166	0.0285
3396	2	9.01	D4A1D4	Nudix (Nucleoside diphosphate linked moiety X)-type motif 14 (Predicted)	2	0.94	1.03	0.90	0.88	0.96	1.00144973	0.976
3397	2	3.90	D4A416	CLPTM1-like	2	0.63	0.96	0.79	0.85	0.52	1.01269519	0.63
3398	2	2.25	A0A0G2JX62	SRSF protein kinase 2	1	0.97	0.84	0.91	0.95	0.86	1.04935294	0.164
3399	2	5.72	M0R830	Mesoderm-specific transcript homolog protein	1	0.63	0.59	0.46	0.72	0.28	0.97393676	0.398
3400	2	1.19	D3ZP44	SLIT and NTRK-like family, member 6	1	0.18	0.19	0.23	0.29	0.25	1.07997656	0.0902
3401	2	2.80	Q99JC6	TAP binding protein	1	0.84	0.54	0.72	0.86	0.82	1.05694478	0.187
3402	2	19.44	Q6PCU8	NADH dehydrogenase [ubiquinone] flavoprotein 3, mitochondrial	1	0.73	0.98	0.86	0.67	0.97	0.99322329	0.838
3403	2	3.30	A0A0G2JSJ6	Solute carrier family 18 (Vesicular monoamine), member 2, isoform CRA_a	1	0.28	0.52	0.40	0.70	0.56	0.92146419	0.0991
3404	2	2.58	Q66HF9	Leucine-rich repeat flightless-interacting protein	2	1.34	1.07	0.98	1.25	1.25	1.02725902	0.12
3405	2	2.86	Q9ER31	Ectonucleoside triphosphate diphosphohydrolase 6	1	1.12	0.86	1.02	1.01	0.82	1.09657159	0.171
3406	2	1.09	A0A0G2K016	Sad1 and UNC84 domain-containing 1	1	1.50	1.33	1.32	1.50	0.90	0.96012889	0.249
3407	2	12.50	B0BN99	High mobility group box 3	2	0.86	1.41	1.32	1.36	1.15	1.17039864	0.0353
3408	2	5.35	Q6AYA6	Uncharacterized protein C17orf62 homolog	1	1.09	0.86	1.18	0.90	1.53	1.03146867	0.398
3409	2	14.50	Q6MG66	LSM2 homolog, U6 small nuclear RNA and mRNA degradation-associated	1	1.11	1.08	0.93	0.77	0.89	0.97535546	0.174
3410	2	4.26	D4AA31	Prolylcarboxypeptidase	1	1.03	0.99	0.93	0.93	0.82	1.01234427	0.671
3411	2	2.28	Q925B4	Synaptotagmin-8	1	1.04	0.78	0.98	0.72	1.31	0.96761162	0.411
3412	2	2.80	D3ZEJ9	Similar to Protein Njmu-R1	1	0.91	0.90	0.90	0.72	0.82	1.09353546	0.0968
3413	2	1.74	Q9QZG1	Solute carrier family 22 member 23	1	0.79	0.61	0.47	2.73	0.56	1.01607014	0.133
3414	2	2.66	P32738	Choline	1	1.19	2.19	1.50	1.53	0.95	1.0242018	0.614
3415	2	3.05	Q5FWT5	Glutamyl-tRNA(Gln) amidotransferase subunit A, mitochondrial	1	0.69	0.50	0.70	0.63	0.72	1.02740144	0.553
3416	2	4.69	A0A0H2UHK(	Swi5-dependent recombination DNA repair protein 1 homolog	1	1.36	1.49	1.51	1.17	1.09	1.11806185	0.174
3417	2	3.14	Q4V7F3	Probable tRNA N6-adenosine threonylcarbamoyltransferas	1	0.86	0.91	0.50	0.79	0.90	1.01438126	0.441
3418	2	4.15	Q75T81	Asc-type amino acid transporter 1	1	0.78	1.41	0.32	1.32	0.90	0.99604995	0.949
3419	2	5.99	F1M362	Golgin A7 family, member	1	0.86	1.06	0.90	1.08	0.91	1.03046821	0.452
3420	2	7.64	D4A2U6	L	1	0.44	1.50	1.09	1.24	1.53	1.04681028	0.169
3421	2	4.32	Q6AXQ4	Myotubularin	2	0.99	0.79	1.18	0.55	0.52	1.02882671	0.462
3422	2	4.11	Q6AYG7	NFATC2-interacting protein	1	0.53	0.82	0.51	0.41	1.07	1.04615745	0.121
3423	2	16.25	P59649	FXYD domain-containing ion transport regulator 7	1	1.13	0.89	1.00	0.99	0.92	1.02782881	0.371
3424	2	2.18	Q5U306	A kinase (PRKA) anchor protein 8-like	1	1.05	1.28	0.64	0.95	0.44	1.0546761	0.271
3425	2	2.99	Q9Z158	Syntaxin-17	1	0.92	1.09	0.98	0.89	0.98	0.99580834	0.861
3426	2	4.47	Q5FVN2	Transmembrane protein 41B	1	0.74	0.47	1.01	1.57	1.08	1.0510272	0.501

3427	2	3.34	D3ZCI2	Solute carrier family 9 (Sodium/hydrogen exchanger), member 7 (Predicted)	1	0.90	0.70	0.86	0.73	0.88	0.88148016	0.0423
3428	2	6.94	F1M5X2	Adaptor-related protein complex 4, sigma 1 subunit	1	0.71	1.54	1.84	0.89	0.80	0.95966315	0.399
3429	2	7.27	G3V6L0	Pro-MCH	1	0.77	0.72	0.34	0.09	0.64	0.98986247	0.685
3430	2	4.57	A0MV40	Proline rich membrane protein	1	1.16	0.84	1.74	1.38	0.95	0.98548103	0.836
3431	2	2.23	A0A0G2K0K3	NPR3-like, GAT	1	0.72	0.77	0.79	0.91	0.66	1.12583859	0.29
3432	2	5.08	Q5U2Q3	Ester hydrolase C11orf54 homolog	1	0.89	1.02	0.82	0.69	0.80	1.05474921	0.313
3433	2	6.77	Q6AYD1	Metallo-beta-lactamase domain-containing protein 1	1	1.07	0.95	0.63	1.08	1.06	0.87843047	0.301
3434	2	6.80	D3ZDP2	Uncharacterized protein	1	0.61	0.95	1.06	0.76	0.65	1.37078281	0.0197
3435	2	5.61	Q6P686		1	1.79	0.88	1.11	0.70	0.75	1.0188912	0.613
3436	2	13.64	M0R3S1	Mitochondrial ribosomal protein S21-like	1	0.74	0.88	0.89	0.95	0.64	1.0807254	0.422
3437	2	3.70	A0A0G2JXD5	Beta-chimaerin	1	1.15	1.26	1.50	1.29	0.90	0.95753691	0.601
3438	2	6.67	D4A8G0	LSM12 homolog	1	1.20	1.34	1.19	1.12	1.20	1.08447741	0.0121
3439	2	3.50	M0RDG7	Uncharacterized protein	1	0.98	1.02	1.12	0.30	1.16	1.04087729	0.41
3440	2	8.33	Q568Z4	Signal peptidase complex subunit 3	1	1.34	1.47	1.28	0.96	0.98	1.07021451	0.0225
3441	2	3.38	M0R521	Arrestin domain-containing	1	null	null	null	null	null	0.96166079	0.172
3442	2	2.14	D3ZF92	Tumor necrosis factor receptor superfamily member 21	1	0.99	0.63	0.88	1.19	0.69	0.97447697	0.163
3443	2	2.76	Q5PPL3	Sterol-4-alpha-carboxylate 3-dehydrogenase, decarboxylating	1	0.72	0.98	0.73	0.70	0.88	0.86994735	0.0584
3444	2	9.84	B2RZD5	RCG41580, isoform CRA_a	1	1.17	0.95	1.12	0.97	0.98	1.03756376	0.186
3445	2	2.08	P50430	Arylsulfatase B	1	1.26	1.27	1.37	0.91	1.03	1.05548056	0.0404
3446	2	0.60	E9PTE1	Son DNA-binding protein	1	1.28	1.46	1.21	0.68	1.16	1.06947296	0.244
3447	2	2.80	Q498C9	RCG33491, isoform CRA_b	1	1.51	1.25	1.92	1.51	1.32	1.04138245	0.435
3448	2	5.45	A0A0G2JXG5	Uncharacterized protein	1	null	null	null	null	null	0.95290216	0.119
3449	2	2.46	I6L9G6	REVERSED RCG31562, isoform CRA_c	1	null	null	null	null	null	null	null
3450	2	2.20	A0A0G2JT23	Nuclear receptor-binding protein 2	1	0.84	1.32	1.06	1.07	1.12	1.02954008	0.785
3451	2	7.19	Q811Q1	Single-strand selective monofunctional uracil-DNA glycosylase	1	0.97	1.07	1.05	1.08	0.73	0.93322703	0.594
3452	2	3.24	P12788	Trypsin-4	1	0.69	0.89	1.26	1.46	0.86	1.10037861	0.187
3453	2	5.16	B2RYM5	Lys-63-specific deubiquitinase BRCC36	1	1.13	0.82	1.18	0.90	0.93	1.00134561	0.935
3454	2	5.74	F1LQ29	Similar to RIKEN cDNA B230118H07	1	1.02	0.92	0.50	0.42	0.95	0.77431903	0.00522
3455	2	7.57	Q08013	Translocon-associated protein subunit gamma	1	1.13	1.19	1.05	1.13	0.86	0.92018765	0.375
3456	2	6.98	Q9R0Z7	Alpha- and gamma-adaptin-binding protein p34	1	1.08	0.90	0.99	0.91	0.92	1.0224994	0.328
3457	2	8.29	Q6IMA8	ADP-ribosylation factor-like 10	1	0.75	0.58	0.68	0.77	0.46	0.93335641	0.266
3458	2	4.29	P53563	Bcl-2-like protein 1	1	0.93	0.94	1.02	1.06	0.99	1.09505247	0.138
3459	2	6.37	Q5M818	39S ribosomal protein L16, mitochondrial	1	1.01	0.84	0.80	0.27	0.84	0.90814842	0.228
3460	2	6.62	Q5FVJ4	RELT-like protein 2	1	null	null	null	null	null	0.98159513	0.759
3461	2	5.94	Q68FS9	C	1	0.83	0.64	0.72	0.85	0.69	0.98636944	0.805
3462	2	10.19	Q6AXW1	Glutaredoxin-2, mitochondrial	1	null	null	null	null	null	0.99669915	0.919
3463	2	6.21	Q7M0E7	39S ribosomal protein L14, mitochondrial	1	1.14	0.94	0.70	0.95	0.85	0.91256549	0.208
3464	2	10.64	Q8CHN7	Neuron-specific protein PEP-19	1	0.90	1.09	0.92	0.90	1.10	0.98869675	0.856
3465	2	14.75	Q5RJZ6	Short coiled-coil protein	1	0.84	0.77	1.05	0.64	0.79	1.17364818	0.0491
3466	2	9.00	D3ZJZ0	RGD1563250 (Predicted), isoform CRA_a	1	0.86	1.34	0.89	1.09	0.84	1.03512142	0.341
3467	2	12.63	P61959	Small ubiquitin-related modifier 2	1	1.05	1.03	1.05	1.06	1.12	0.98227576	0.61
3468	2	10.78	P04550	Parathymosin	1	1.02	1.03	1.09	0.96	1.03	1.14155471	0.0439
3469	2	8.22	B0BNL4	Heme transporter HRG1	1	0.82	1.13	0.67	0.22	0.49	0.92787348	0.182
3470	2	6.79	Q9JJW1	Tetraspanin-2	1	1.11	1.71	1.41	1.25	1.20	0.98302499	0.807
3471	2	12.43	Q5FVJ7	Intraflagellar transport protein 22 homolog	1	1.06	1.17	0.94	0.63	0.80	0.95243992	0.208
3472	2	8.17	A0A0G2JV28	Male-enhanced antigen 1	1	0.69	0.78	0.79	0.53	0.88	1.01755022	0.912
3473	2	4.05	Q6QBQ4	Phospholipid scramblase 3	1	0.78	0.68	0.95	0.79	0.84	1.16392653	0.166
3474	2	13.04	P05506	NADH-ubiquinone oxidoreductase chain 3	1	0.67	0.04	0.83	1.22	0.90	0.95893172	0.728
3475	2	8.52	P0C0A1	Vacuolar protein-sorting-associated protein 25	1	0.72	0.82	0.45	0.52	0.90	0.69207486	0.119

3476	2	5.16	Q5BJP9	Phytanoyl-CoA dioxygenase domain-containing protein 1	1	1.47	1.20	1.80	1.29	1.34	1.08372597	0.606
3477	2	17.14	D3ZPL2	Uncharacterized protein	1	0.78	0.89	0.71	0.77	0.98	1.0097513	0.917
3478	2	8.57	D3ZA93	Acyl-CoA thioesterase 13	1	0.79	1.00	0.82	0.60	0.78	0.82645032	0.02
3479	2	5.40	A0A0G2JVS7	Probable cytosolic iron-sulfur protein assembly	1	0.92	0.88	0.89	1.05	0.82	0.87478477	0.313
3480	2	14.39	A0A0G2JW13	NACHT and WD repeat domain-containing protein 2-like	1	0.89	0.79	0.77	0.70	0.81	0.96902111	0.625
3481	2	17.76	D3ZBW5	Hypothetical protein L	1	1.00	0.88	0.86	0.94	0.80	0.77539321	0.139
3482	2	14.40	D4A465	Late endosomal/lysosomal adaptor, MAPK and MT	1	0.72	0.47	0.55	0.47	0.59	0.8461586	0.241
3483	2	19.00	A0A0G2K8Q1	Apolipoprotein C-III	1	null	null	null	null	null	1.14631219	0.12
3484	2	23.71	D3Z9A4	RCG20739, isoform CRA_b	1	0.54	0.34	1.01	0.43	0.74	0.99462183	0.988
3485	2	20.22	Q5M9I5	Cytochrome b-c1 complex subunit 6, mitochondrial	1	1.11	0.12	1.28	0.78	1.08	1.10880064	0.758
3486	1.99	3.00	A0A0G2JVP5	Poly(A)-binding protein-interacting protein 1	1	1.10	0.98	0.99	1.10	0.95	1.02469887	0.432
3487	1.99	5.58	A0A0G2JVL5	Similar to expressed sequence AW549877	1	1.26	0.71	1.01	0.94	1.00	1.04261029	0.143
3488	1.99	1.00	Q7TQ74	Ac1573	1	1.32	0.89	0.95	1.13	0.69	1.01206363	0.739
3489	1.98	1.41	D3ZL50	Tetratricopeptide repeat domain 37	2	0.95	1.05	0.93	1.06	0.96	1.0224994	0.421
3490	1.98	4.02	Q711G3	Isoamyl acetate-hydrolyzing esterase 1 homolog	1	0.95	1.08	0.95	1.07	0.88	0.92916067	0.0598
3491	1.98	4.79	Q5XIW0	Mitochondrial import inner membrane translocase subunit TIM23	1	0.93	0.97	1.00	1.05	0.82	1.03089686	0.238
3492	1.98	41.67	G3V6P8	Guanine nucleotide-binding protein subunit gamma	2	1.06	0.92	0.80	0.93	0.83	0.85678196	0.0836
3493	1.97	1.60	F1M981	Phosphatidylinositol 3,4,5-trisphosphate 5-phosphatase 1	3	0.88	0.95	0.63	0.67	0.57	1.01910309	0.403
3494	1.97	6.89	B1WBV1	Axin interactor, dorsalization-associated	2	1.11	1.05	1.02	1.18	1.02	1.0040701	0.861
3495	1.96	1.34	D4AB70	alpha-1,2-Mannosidase	1	1.06	0.95	0.93	1.08	0.86	1.06333781	0.0109
3496	1.96	4.59	M0R4J7	Dishevelled-binding antagonist of beta-catenin 3	2	1.58	1.41	1.46	2.00	2.00	0.76365875	0.0325
3497	1.95	4.43	A0A0G2JXT6	Myotubularin-related protein 6	2	1.00	0.89	0.80	0.87	0.86	1.04913476	0.0992
3498	1.95	1.59	F1LRE0	Voltage-dependent N-type calcium channel subunit alpha	3	1.13	1.31	1.19	1.29	1.33	0.99910624	0.972
3499	1.95	3.09	P17988	Sulfotransferase 1A1	1	null	null	null	null	null	1.00647407	0.727
3500	1.95	3.51	P0C6C0	A-kinase anchor protein SPHKAP	4	0.77	1.05	0.54	1.22	1.04	1.04188786	0.388
3501	1.95	4.22	Q5RK17	Diablo homolog	1	0.87	0.88	0.95	1.02	0.96	1.02271205	0.471
3502	1.95	7.58	Q0ZCA7	C-type lectin domain family 2 member L	1	1.18	1.22	1.28	1.33	1.56	1.07997656	0.0926
3503	1.95	3.45	B1H219	Dickkopf WNT-signaling pathway inhibitor 3	1	1.06	1.05	1.10	1.00	1.10	0.96720929	0.623
3504	1.94	2.55	G3V8U8	Branched-chain-amino-acid aminotransferase	1	1.28	1.21	1.09	1.25	1.26	0.93867654	0.306
3505	1.94	11.81	Q63396	Activated RNA polymerase II transcriptional coactivator p15	2	1.28	1.36	1.04	1.05	1.07	0.85086373	0.176
3506	1.94	4.56	A0A0H2UI06	NADH dehydrogenase (ubiquinone) complex I, assembly factor 6	1	0.88	1.17	0.86	0.96	1.01	0.98753241	0.714
3507	1.94	4.39	Q5XJW2	Growth arrest and DNA damage-inducible proteins-interacting protein 1	1	0.56	0.77	0.89	1.00	0.76	0.86633686	0.0222
3508	1.93	0.87	F1M4N6	Dedicator of cyto-kinesis 3	2	0.99	0.98	0.92	0.90	0.94	0.97474719	0.0142
3509	1.93	1.16	D4A962	Heterogeneous nuclear ribonucleoprotein U-like 1	1	1.25	1.49	1.21	1.19	1.24	0.98883383	0.541
3510	1.93	3.10	M0RDW3	Probable N-acetyltransferase CML2-like	1	1.24	1.02	1.11	0.96	0.98	0.86333956	0.0107
3511	1.93	2.27	Q5XIJ1	REVERSED Embryonic stem cell-specific 5-hydroxymethylcytosine-binding protein	1	2.19	1.42	0.95	0.96	0.98	null	null
3512	1.93	2.49	F1M7P8	SPRY domain-containing 3	1	0.45	0.52	0.70	0.56	0.62	0.99226681	0.865
3513	1.92	4.95	Q62796	RalA-binding protein 1	3	1.50	1.51	1.39	0.95	1.04	1.0118532	0.773
3514	1.92	1.40	F1LR98	Cadherin 10	1	0.91	1.01	1.06	1.03	0.96	1.0118532	0.734
3515	1.92	6.80	G3V871	Gastrin releasing peptide, isoform CRA_a	1	0.27	0.51	0.53	0.39	0.37	0.99378798	0.938
3516	1.92	9.84	M0RD87	Dual specificity phosphatase-like 15 (Predicted), isoform CRA_b	1	0.82	0.11	1.66	0.37	1.43	0.93900191	0.449
3517	1.92	5.20	D3ZWJ1	DNL-type zinc finger	1	0.92	1.67	1.92	1.29	1.36	1.07997656	0.348

3518	1.91	3.46	Q3MID3	ADP-ribosylation factor GTPase-activating protein 2	2	0.98	1.09	0.95	1.24	0.79	1.02377594	0.276
3519	1.91	1.29	F1M062	La ribonucleoprotein domain family, member 1	1	0.92	0.90	0.80	0.90	0.87	0.97609942	0.402
3520	1.89	0.98	D3Z8R4	RNA-binding motif protein 25-like 1	1	1.00	1.14	1.09	1.07	1.12	1.0242728	0.146
3521	1.89	3.68	D4A120	Metallophosphoesterase domain containing 1 (Predicted)	1	null	null	null	null	null	1.02491197	0.698
3522	1.89	2.36	B5DFJ4	VPS18 C	2	0.96	0.93	1.00	0.87	0.99	0.98712179	0.631
3523	1.89	5.46	O54857	Phosphatase and tensin homolog	2	1.07	1.04	1.17	1.21	1.20	1.03763568	0.0321
3524	1.89	2.57	G3V9U0	Acyl-CoA synthetase short-chain family member 2	1	0.80	1.13	0.96	1.12	1.01	1.01178307	0.495
3525	1.88	6.29	G3V805	EKC/KE	1	2.00	0.67	0.80	1.13	1.61	1.12116608	0.0559
3526	1.87	1.63	P0C5Y8	Alsin	3	1.05	1.36	1.25	1.24	0.45	1.11728714	0.101
3527	1.87	3.30	G3V8F1	Transporter	2	1.06	1.05	0.96	1.01	0.94	0.96520012	0.657
3528	1.86	12.32	Q5XI60	Receptor expression-enhancing protein 6	3	0.86	1.03	0.86	0.95	0.95	1.05372617	0.36
3529	1.86	3.35	Q5XIW8	U4/U6.U5 tri-snRNP-associated protein 1	2	0.82	0.44	1.15	0.79	1.39	1.01705662	0.41
3530	1.86	3.15	F1M400	Ankyrin repeat domain 28	2	0.82	0.76	0.94	0.95	1.05	1.08522937	0.00751
3531	1.86	16.83	D3ZWF5	Transcription and mRNA export factor ENY2	1	0.77	1.07	0.84	0.87	0.74	1.09126388	0.244
3532	1.85	3.64	F1LXF5	Golgi to ER traffic protein 4	1	0.94	1.19	1.01	0.91	1.01	1.03741994	0.471
3533	1.85	3.24	A0A096MJY1	Glypican 6	1	0.61	0.81	0.84	1.13	0.70	1.15829281	0.0495
3534	1.85	2.87	Q9JKY3	Zinc finger and BTB domain-containing protein	1	1.11	0.70	0.79	1.13	1.72	1.13131446	0.03
3535	1.85	3.77	E9PSP3	Delta/notch-like EGF repeat-containing	1	0.84	0.54	1.18	0.69	0.72	0.98931373	0.872
3536	1.84	8.52	Q9R0C9	Sigma non-opioid intracellular receptor 1	2	null	null	null	null	null	0.92018765	0.00539
3537	1.84	4.83	D4A414	C	2	1.11	1.16	1.03	1.19	1.24	0.94743574	0.29
3538	1.84	3.78	Q5XI31	GPI transamidase component PIG-S	1	0.93	1.06	1.04	1.06	0.96	0.94717309	0.022
3539	1.83	6.64	Q68FS1	Cytosolic Fe-S cluster assembly factor NUBP2	1	0.98	1.03	0.78	0.82	0.99	1.0153661	0.485
3540	1.83	1.93	A0A0G2K7Y(	Complement component receptor 1-like protein	1	0.86	0.81	1.07	0.79	1.11	0.95277007	0.165
3541	1.82	45.24	D4A817	Histone H2B	26	1.74	1.08	0.65	0.78	0.43	0.59998569	0.01095
3542	1.82	4.06	Q5I0M1	Apolipoprotein H	1	1.02	0.88	1.03	0.93	1.12	1.01719763	0.724
3543	1.82	14.29	P62275	40S ribosomal protein S29	1	0.92	1.11	1.18	1.32	1.19	1.0807254	0.0742
3544	1.8	1.53	D3ZKK3	Consortin, connexin sorting protein	1	0.70	1.11	1.06	0.69	1.28	1.02306655	0.426
3545	1.8	1.80	D3ZDA6	Sidekick cell adhesion molecule 2	2	0.82	0.86	0.46	0.63	0.55	0.98200345	0.468
3546	1.8	5.00	B2RZ57	Mitochondrial ribosomal protein L18	1	0.48	0.73	0.47	1.02	0.72	0.98261625	0.763
3547	1.78	9.25	D4A055	Voltage-dependent L-type calcium channel subunit beta-4	5	0.92	0.94	0.94	0.99	1.08	1.05306902	0.0328
3548	1.78	21.56	P05714	Ras-related protein Rab-4A	6	1.17	0.91	1.10	0.99	1.11	1.00674618	0.801
3549	1.78	3.52	F1M1C9	Phosphorylase kinase, alpha	1	0.72	0.81	0.61	0.93	0.64	1.02179091	0.679
3550	1.77	2.20	P56819	Beta-secretase 1	1	0.89	0.80	0.86	1.07	0.95	1.02016323	0.486
3551	1.76	8.09	F1LMN8	KH domain-containing, RNA-binding, signal transduction-associated	3	1.25	1.12	1.06	1.20	1.09	0.99862159	0.942
3552	1.76	4.35	F1M7H7	Membrane-associated guanylate kinase, WW and PDZ domain-containing protein 1	3	0.93	0.87	0.95	0.89	0.95	1.00173437	0.945
3553	1.76	8.46	Q4V8E4	Cilia- and flagella-associated protein 36	2	0.82	0.77	0.82	0.83	0.88	1.17039864	0.0473
3554	1.76	2.56	Q5XIJ4	Protein FAM210A	1	0.94	0.96	0.75	0.88	0.86	1.03146867	0.448
3555	1.75	4.78	F1MAJ0	Eph receptor B2	5	1.12	1.09	1.36	1.13	1.08	1.07848043	0.0979
3556	1.75	3.69	F7ENH8	Histone deacetylase	2	1.04	1.03	1.09	1.57	1.13	0.99586356	0.867
3557	1.75	0.74	D4A1P7	Meiosis-specific with coiled-coil domain	1	null	null	null	null	null	1.02740144	0.425
3558	1.75	11.88	D3ZM03	Vacuolar ATPase assembly integral membrane protein VMA21	1	1.01	0.95	0.89	0.86	0.81	0.86035263	0.155
3559	1.74	4.79	Q5XXR3	Rho guanine nucleotide exchange factor 6	3	0.92	1.22	1.12	0.94	1.02	1.00954135	0.445
3560	1.74	3.15	G3V8S7	Potassium intermediate/small conductance calcium-activated channel, subfamily	2	1.10	0.86	0.92	0.95	0.72	1.05819096	0.155
3561	1.74	7.93	D4A4U3	Magnesium-dependent phosphatase 1	1	1.09	1.18	0.96	0.88	0.84	0.83914964	0.0513
3562	1.74	8.62	Q5XI90	Dynein light chain Tctex-type 3	1	1.47	0.32	1.06	0.91	1.08	1.24573742	0.0082

3563	1.73	5.00	Q6AYP7	7-methylguanosine phosphate-specific 5'-nucleotidase	2	0.85	1.02	0.79	0.87	0.70	1.14472416	0.0931
3564	1.73	9.09	D4ABX9	ER membrane protein complex subunit 6	1	1.10	1.15	0.97	0.81	0.95	1.09050773	0.0391
3565	1.72	2.12	Q5RKJ4	Farnesyltransferase, CAAX box, alpha	1	1.10	0.54	0.78	0.33	1.08	1.50420375	0.03579
3566	1.72	5.09	A0A0A0MY0[illegible]	Junctophilin-4	2	1.17	0.93	1.05	0.80	1.16	0.96459819	0.0535
3567	1.72	13.68	M0RDZ5	Uncharacterized protein	1	1.18	1.69	1.85	0.72	1.00	0.87903956	0.188
3568	1.72	2.31	G3V6A2	SLAIN motif family, member 2	1	0.61	0.81	0.79	0.67	0.83	1.17772279	0.0221
3569	1.71	4.22	M0R959	Methylmalonic aciduria (cobalamin deficiency) cblB type	1	0.97	0.87	0.93	0.74	0.61	1.06054071	0.378
3570	1.71	1.54	P07687	Epoxide hydrolase 1	1	1.54	1.01	0.85	1.74	1.37	0.97772456	0.641
3571	1.7	10.63	A0A0G2JX25	GMP reductase	3	0.95	1.03	1.15	1.11	1.13	0.99946503	0.982
3572	1.7	9.97	Q9JL55	Glycerophosphodiester phosphodiesterase 1	3	0.72	0.73	0.93	1.10	0.81	0.99620185	0.889
3573	1.7	4.43	D3ZUQ0	RILP-like protein 1	1	0.94	1.06	1.01	0.99	0.92	1.0387871	0.305
3574	1.7	0.58	D3ZQY4	FRY-like transcription coactivator	1	0.84	0.84	0.92	0.94	0.95	1.01945635	0.486
3575	1.69	9.31	Q8VGC3	Voltage-dependent L-type calcium channel subunit beta-2	5	1.22	1.12	1.13	1.31	1.29	1.00926148	0.607
3576	1.69	3.17	D4ABL6	Multiple C2 and transmembrane domain-containing 1	2	0.59	1.00	0.73	1.02	0.93	0.97989563	0.263
3577	1.69	1.27	A0A096MK5[illegible]	RCG24674, isoform CRA_e	3	1.38	1.14	1.19	1.03	1.25	0.99837243	0.979
3578	1.69	2.02	E9PSP1	Phospholipid transfer	1	0.94	0.98	1.43	0.99	1.13	1.10726458	0.0253
3579	1.69	4.71	F7EVX2	Translation initiation factor eIF-2B subunit beta	1	0.75	0.90	0.82	0.87	0.72	0.98254814	0.56
3580	1.69	2.66	F1M6U2	Uncharacterized protein	1	0.87	0.75	0.67	1.26	0.89	1.07922824	0.0624
3581	1.68	0.77	A0A0G2K9I3	Golgin subfamily A member	2	1.00	0.91	1.08	1.01	0.95	0.95826727	0.0321
3582	1.68	1.95	B2GVB9	Fermitin family member 3	1	1.02	0.54	1.57	1.42	0.65	0.94494352	0.049
3583	1.68	4.15	Q63486	Ras-related GTP-binding protein A	1	1.01	0.74	0.95	1.01	1.01	1.00202604	0.97
3584	1.68	7.00	A0A0G2K6V[illegible]	Carboxylic ester hydrolase	2	1.03	0.86	1.01	0.89	0.86	1.07140208	0.024
3585	1.68	4.52	Q3B7D0		1	0.62	0.56	0.60	0.82	0.63	1.10266916	0.0903
3586	1.68	1.46	D4ADD3	HECT, C2 and WW domain-containing E3 ubiquitin protein ligase 2	2	1.03	1.00	0.84	0.80	0.96	0.96600329	0.199
3587	1.68	1.23	G3V6X1	Fibulin 2	1	0.83	0.94	2.73	1.61	1.74	1.02782881	0.423
3588	1.67	16.13	O88656	Actin-related protein 2/3 complex subunit 1B	4	1.29	1.26	1.67	1.74	1.60	0.93400359	0.064
3589	1.67	5.79	A0A0G2K191	Tubulin-specific chaperone	4	2.13	0.83	1.58	1.16	1.32	0.99687879	0.911
3590	1.67	3.01	G3V7V9	Leucine carboxyl methyltransferase 1	1	1.07	1.15	0.96	0.80	0.93	1.01550687	0.668
3591	1.67	1.40	D3ZW14	DEP domain containing 2 (Predicted)	1	1.00	0.70	0.75	0.96	0.95	1.00536544	0.878
3592	1.66	1.68	D3ZU57	Bifunctional lysine-specific demethylase and histidyl-hydroxylase N	1	null	null	null	null	null	1.11419365	0.149
3593	1.66	7.62	Q5PPH7	Dynein, axonemal, light chain 4	1	0.97	1.05	1.17	1.11	1.33	1.08748839	0.228
3594	1.65	2.19	G3V631	RAB guanine nucleotide exchange factor (GEF) 1 (Predicted), isoform CRA_a	1	0.86	1.01	0.83	0.66	0.85	1.08598186	0.0904
3595	1.65	2.86	F1LND1	Component of oligomeric golgi complex 1	2	0.66	0.78	0.73	0.93	0.86	1.00828256	0.767
3596	1.65	7.71	A0A0G2JXM[illegible]	DNA polymerase delta-interacting protein 3	2	0.91	0.89	0.91	0.82	0.87	1.00884183	0.437
3597	1.65	0.47	D3ZXX3	Unc-80 homolog, NALCN activator (Fragment)	1	1.18	1.31	0.62	0.96	0.85	1.08673486	0.12
3598	1.64	1.88	M0R4G0	Immunoglobulin superfamily-containing leucine-rich repeat 2	1	1.01	0.96	0.79	0.95	0.87	0.99157926	0.714
3599	1.64	3.70	Q5I0K5	Mycophenolic acid acyl-glucuronide esterase, mitochondrial	1	0.79	0.95	0.88	0.76	0.78	1.04058874	0.574
3600	1.64	2.70	Q9JMC1	Phosphatidylinositol 4,5-bisphosphate 5-phosphatase A	2	1.00	1.16	0.90	1.36	1.18	1.02115368	0.589
3601	1.63	6.65	Q08603	Geranylgeranyl transferase type-2 subunit beta	2	0.82	0.96	1.08	1.06	0.94	1.00786332	0.694
3602	1.63	2.19	D3ZV15	Cbl proto-oncogene	2	0.98	0.83	0.93	1.04	1.05	1.03282799	0.187
3603	1.63	2.52	D4A060	Sorting nexin family member 30	1	1.02	1.03	0.90	1.00	1.10	0.96079464	0.373
3604	1.63	4.85	F1M8I7	Ena/VASP-like protein	1	1.06	0.82	1.05	0.78	0.76	1.10956947	0.00513
3605	1.63	2.10	P32215	Pituitary adenylate cyclase-activating polypeptide type I receptor	1	0.74	1.24	0.60	0.89	0.62	1.02832764	0.372

3606	1.62	5.32	Q6P756	Adaptin ear-binding coat-associated protein 2	1	0.93	1.26	0.94	0.76	0.73	0.96106106	0.325
3607	1.62	7.59	Q5U2R7	LDLR chaperone MESD	2	1.06	0.83	0.94	0.90	0.96	1.0167042	0.255
3608	1.62	3.87	Q5PPM8	Type 1 phosphatidylinositol 4,5-bisphosphate 4-phosphatase	1	1.60	0.70	1.50	0.93	1.28	1.17202228	0.465
3609	1.62	9.46	Q5HZW9	Ecotropic viral integration site 2A	2	0.60	0.58	0.71	0.82	0.75	0.97434189	0.902
3610	1.61	10.40	A0A0G2JW8	Microtubule-associated protein	23	0.86	0.91	0.90	1.34	1.19	1.09505247	0.24
3611	1.61	8.70	Q6AYJ0	Serine/threonine-protein phosphatase	9	0.82	0.73	0.85	0.71	0.70	1.05767765	0.449
3612	1.61	4.16	Q5FVC7	Arf-GAP with coiled-coil, ANK repeat and PH domain-containing protein 2	3	1.11	1.16	1.09	1.05	1.08	1.03562378	0.308
3613	1.61	3.12	P47820	Angiotensin-converting enzyme	3	0.33	0.39	0.25	0.47	0.77	0.98363843	0.59
3614	1.61	2.66	Q4V896	Sorting nexin-15	1	0.59	0.70	0.70	0.86	0.41	1.05255819	0.185
3615	1.61	5.91	D4A9Y0	RCG36668	1	1.38	0.73	1.09	1.38	1.43	1.15668818	0.431
3616	1.61	8.60	Q62881	Nucleolar protein 3	1	0.73	0.80	0.90	0.69	0.72	0.9959602	0.938
3617	1.6	4.78	Q5EAN5	Secretory carrier-associated membrane protein	1	1.17	1.18	1.20	1.06	1.09	1.04731832	0.0922
3618	1.59	1.13	E9PU32	Chloride channel protein	1	0.72	1.01	0.79	0.95	1.02	1.01853814	0.361
3619	1.59	2.29	A0A0G2K174	LIM domain 7	2	1.45	1.49	0.71	0.80	0.69	1.31767995	0.0236
3620	1.59	4.69	B0BMU7	Elongator complex protein 5	1	0.59	0.95	0.88	0.46	0.58	0.95422408	0.212
3621	1.59	18.07	D3ZSE0	40S ribosomal protein S21	1	0.66	0.57	0.84	0.86	0.96	1.00245675	0.933
3622	1.59	5.42	D3ZGY1	PYM homolog 1, exon junction complex-associated factor	1	1.18	1.18	1.13	1.03	1.37	1.46003201	0.145
3623	1.58	4.92	A0A0G2JZ60	Fibronectin type III and SPRY domain-containing 1-like	2	0.92	0.93	0.90	0.90	1.04	0.99905084	0.946
3624	1.58	1.22	Q68FP9	Conserved oligomeric Golgi complex subunit 6	1	1.19	0.82	0.91	0.94	0.63	1.00437638	0.877
3625	1.58	1.39	A0A0H2UHA	Volume-regulated anion channel subunit LRRC8D	1	0.88	0.87	0.92	0.91	1.13	1.0238469	0.456
3626	1.58	1.47	A0A0G2KB5	Microtubule-associated protein 7	1	0.98	0.96	0.96	0.93	0.90	0.93109482	0.175
3627	1.58	1.99	Q64654	Lanosterol 14-alpha demethylase	1	1.12	1.01	0.97	0.95	0.94	1.03082541	0.409
3628	1.58	1.41	P20781	Glycine receptor subunit	1	0.45	1.14	0.79	1.32	0.40	0.96888678	0.692
3629	1.57	4.35	Q63688	25-hydroxycholesterol 7-alpha-hydroxylase (Fragment)	2	0.98	1.03	1.38	0.95	1.04	1.05255819	0.119
3630	1.57	1.42	Q4V8K2	Beta-catenin-like protein 1	1	0.69	0.74	0.79	0.61	0.72	0.99755618	0.939
3631	1.57	3.82	F2Z3T9	Similar to U2 (RNU2) small nuclear RNA auxiliary factor 2 isoform b	1	0.94	0.38	0.98	0.62	0.77	1.06925059	0.0763
3632	1.57	2.93	A0A0G2JYM	Hsp90 co-chaperone Cdc37-like 1	1	0.92	0.77	0.84	0.92	0.89	0.92466328	0.191
3633	1.55	1.56	B1WC03	REVERSED AAR2-splicing factor homolog	1	null	null	null	null	null	null	null
3634	1.55	2.45	Q62918	Protein kinase C-binding protein NELL2	1	1.04	0.98	0.92	0.87	0.94	1.01522535	0.65
3635	1.55	9.43	P83883	60S ribosomal protein L36a	1	1.01	1.38	1.61	1.89	1.58	1.04037238	0.0459
3636	1.55	0.99	B4F7C9	Serine/threonine-protein kinase Chk1	1	0.95	1.27	0.92	0.70	0.86	0.92466328	0.0627
3637	1.55	3.35	P29975	Aquaporin-1	1	0.70	0.82	0.64	1.09	1.06	1.05409142	0.392
3638	1.54	33.75	G3V7U2	Microtubule-associated protein 1 A, isoform CRA_c	81	1.60	1.13	0.70	0.88	1.04	0.90312651	0.0023
3639	1.54	2.75	G3V8M6	Folate receptor 1	1	0.18	0.18	0.08	0.48	0.70	0.82074161	0.588
3640	1.53	1.20	A0A0G2K3V	DNA topoisomerase 1	1	0.93	0.44	0.80	1.09	1.25	1.0637064	0.0647
3641	1.53	2.74	D3ZYQ8	Uridine kinase	1	1.06	0.84	1.05	0.85	1.07	0.95753691	0.542
3642	1.52	3.91	Q5FVF3	Golgi associated, gamma adaptin ear containing, ARF binding protein 1	3	1.04	1.10	0.82	0.89	0.96	1.06584674	0.007
3643	1.52	5.73	A0A0G2JUB	Protein FAM3C	1	0.95	0.92	0.99	0.72	0.90	1.0100313	0.832
3644	1.51	2.29	D3ZAS8	Squamous cell carcinoma antigen recognized by T-cells 3	2	1.08	1.06	0.95	0.94	1.09	0.97616708	0.171
3645	1.51	13.17	Q6P9X4	Protein tyrosine phosphatase type IVA 2	2	1.06	1.10	0.98	0.95	0.93	0.85916076	0.0406
3646	1.5	17.36	Q6XVN8	Microtubule-associated proteins 1A/1B light chain	3	1.38	1.31	1.14	1.12	1.37	0.90187538	0.217
3647	1.5	10.47	B2RYU7	Cbx5 protein	2	1.12	1.34	1.14	1.12	1.28	0.99518042	0.955
3648	1.5	5.62	Q6AYF8	RCG43931	2	1.03	0.90	0.90	1.02	0.96	0.96433078	0.0206
3649	1.49	62.31	Q9JHY2	Sideroflexin-3	28	0.54	4.09	2.75	1.82	1.47	0.95006623	0.0418
3650	1.49	1.67	A0A0H2UHK	Leucyl-cystinyl aminopeptidase-like	2	1.12	1.00	1.06	1.22	0.96	0.90563398	0.0786

3651	1.49	1.96	M0RBH7	REVERSED Tetratricopeptide repeat domain 16	2	null	null	null	null	null	null	null
3652	1.49	2.75	D3ZHP7	Serine/threonine-protein kinase ULK3	1	0.76	0.90	0.72	0.76	0.86	0.99027423	0.794
3653	1.48	3.81	F7FM32	Similar to CG9752-PA	1	1.14	1.74	0.92	1.21	0.70	1.00863207	0.78
3654	1.48	9.55	B2GV38	Ubiquitin-like protein 4A	1	1.07	0.92	0.79	0.81	0.90	1.20915568	0.0455
3655	1.48	8.97	Q62760	Mitochondrial import receptor subunit T	1	0.85	1.02	0.98	0.89	0.79	1.00996129	0.706
3656	1.47	53.26	A0A0G2K2M2	Mitochondrial fission factor-like	13	null	null	null	null	null	1.00385438	0.887
3657	1.47	4.08	Q5M7T2	SPRY domain-containing protein 7	1	0.72	0.89	0.69	0.90	0.72	1.07177346	0.00094
3658	1.47	15.46	D4A7Z2	Ribosomal protein L37	2	1.26	1.39	1.54	1.64	1.71	1.0442014	0.296
3659	1.46	5.14	M0R7R2	Similar to Protein C6orf203	1	0.65	0.94	0.76	0.86	0.93	1.0685097	0.08
3660	1.45	1.30	Q3T1G7	Conserved oligomeric Golgi complex subunit 7	1	0.79	1.02	0.72	0.82	0.77	1.02889802	0.284
3661	1.45	1.22	P21213	Histidine ammonia-lyase	1	null	null	null	null	null	1.03039679	0.355
3662	1.44	1.40	A0A0G2K344	Phosphatidylinositol 4,5-bisphosphate 3-kinase catalytic subunit alpha isoform	2	1.75	1.19	1.41	1.57	1.63	0.97177889	0.116
3663	1.44	1.77	Q7TP58	REVERSED Phosphoglycerate mutase	1	0.90	0.97	1.03	0.98	1.06	null	null
3664	1.44	2.43	B0K026	LETM1 domain-containing	1	0.94	0.84	0.94	0.82	0.81	0.96246101	0.0149
3665	1.43	2.78	A9CMA6	Transmembrane protein 163	1	1.07	0.97	0.93	1.05	0.86	1.07549439	0.0249
3666	1.42	2.07	F7F389	Complement component C9	2	2.29	1.61	0.94	0.82	1.17	1.09050773	0.115
3667	1.42	1.98	F1M7Z1	Enhancer of mRNA-decapping 3	1	0.85	0.78	1.13	0.79	0.97	1.0210829	0.154
3668	1.42	1.76	F1LSU0	RT1 class Ib, locus M4	1	null	null	null	null	null	0.8863817	0.0179
3669	1.41	1.49	A0A0G2K9H6	Synaptotagmin-17	2	0.92	0.99	0.99	0.95	0.86	0.94881584	0.172
3670	1.41	1.91	Q5XHY7	Signal transducing adapter molecule 2	1	0.55	0.22	0.50	0.48	0.70	0.99267957	0.734
3671	1.41	4.62	D4A463	RCG29268	1	1.02	0.92	0.96	0.83	0.75	0.98336574	0.656
3672	1.41	3.40	Q6JP77	A-kinase anchor protein 7 isoforms delta and gamma	1	1.04	1.12	1.15	1.13	1.10	1.02299564	0.263
3673	1.41	6.62	A0A0G2KB55	SUM	1	1.06	1.17	1.24	0.96	0.99	0.95197791	0.414
3674	1.41	3.02	G3V904	Phospholipase D family, member 4	1	0.51	0.95	0.56	0.93	0.80	0.93919719	0.0554
3675	1.4	1.90	Q5NDF0	Protein	1	0.77	0.89	0.77	0.79	0.92	1.04543256	0.418
3676	1.39	2.76	G3V843	Prothrombin	1	0.87	0.50	0.90	0.69	0.83	1.06208557	0.0226
3677	1.39	1.81	P25409	REVERSED Alanine aminotransferase 1	1	1.08	1.03	0.90	0.86	0.95	null	null
3678	1.39	2.59	A0A0G2JZY0	LSM family member 14B	1	1.08	1.14	0.86	0.93	0.96	1.01402977	0.583
3679	1.39	2.24	Q920R3	Fatty acid desaturase 1	1	0.78	0.73	0.69	0.82	0.93	1.00779346	0.855
3680	1.38	1.77	Q66H54	FTS and Hook-interacting protein	1	1.09	1.32	1.29	1.75	0.69	0.97901305	0.707
3681	1.38	5.53	Q4KMA3	L	1	1.02	1.02	1.05	0.95	0.94	0.92658806	0.0233
3682	1.38	3.18	Q80XF7	Gap junction gamma-2 protein	1	0.74	0.73	1.14	0.82	1.14	0.98459342	0.708
3683	1.37	3.69	A0A096MJ11	Gasdermin D	2	0.85	0.87	0.92	0.91	0.96	0.9821396	0.284
3684	1.37	0.80	D3ZGJ2	REVERSED Espin-like	1	0.91	0.95	0.98	0.82	1.12	null	null
3685	1.37	11.94	P11608	ATP synthase protein 8	1	0.92	1.07	1.02	1.03	0.92	0.96781285	0.358
3686	1.36	8.37	Q6IRG9	AP-3 complex subunit mu-1	3	0.41	0.53	0.73	0.58	0.52	0.97569355	0.374
3687	1.36	4.37	Q3B8R7	Mitochondrial ribosomal protein L47	1	0.89	0.91	0.69	0.63	0.95	1.02683189	0.42
3688	1.36	3.29	D3ZH36	Extracellular leucine-rich repeat and fibronectin type III domain-containing 2	2	1.04	0.96	1.10	1.10	0.96	1.09277774	0.147
3689	1.36	3.50	D3ZJY1	Mitochondrial ribosomal protein L28	1	1.20	1.22	1.33	1.24	1.28	0.97596411	0.527
3690	1.35	3.85	D3ZDI5	Rho GTPase-activating protein 12	2	1.00	1.11	1.05	1.06	1.06	0.99650573	0.879
3691	1.35	3.50	Q5XID0	Protein YIPF5	1	null	null	null	null	null	1.00082519	0.986
3692	1.35	11.24	M0R4A0	Protein phosphatase 1, regulatory subunit 3G	2	0.86	0.92	0.95	1.04	0.90	1.06017322	0.389
3693	1.35	3.09	D3ZQM7	Inositol-tetrakisphosphate 1-kinase	1	0.27	0.44	0.40	0.49	0.37	0.93568835	0.28
3694	1.34	12.89	G3V6U4	ELAV-like protein	5	1.17	1.08	0.64	0.85	0.86	0.9506591	0.0462
3695	1.34	5.37	O88801	Homer protein homolog 2	3	1.00	0.96	0.82	0.93	1.15	0.85440974	0.201
3696	1.34	1.32	G3V6Q9	BMP/retinoic acid-inducible neural-specific protein 1	1	1.72	0.72	1.64	1.69	1.54	1.03540845	0.466
3697	1.34	3.50	Q9JI56	SNAP-29 protein	1	0.98	1.02	0.97	1.10	0.87	0.97955608	0.0818
3698	1.34	3.28	F1LPB8	Ectonucleoside triphosphate diphosphohydrolase 5	1	0.68	0.90	0.76	0.35	0.77	1.05139152	0.16
3699	1.34	3.20	D4A1F3	Uncharacterized protein	1	1.01	0.93	0.84	1.00	1.26	0.96326189	0.191
3700	1.34	1.64	Q3ZAU5	DDHD domain containing 1	1	1.28	0.79	0.93	1.00	0.95	1.00076969	0.976
3701	1.33	0.73	D3ZZA8	SEC24 homolog A, C	1	null	null	null	null	null	0.99954401	0.983
3702	1.33	14.09	B2GV62	Mitochondrial ribosomal protein L20	1	1.15	1.57	0.93	0.95	0.93	0.99891235	0.971
3703	1.32	1.77	A0A0G2JVF1	RCG41069	2	0.90	1.02	1.02	1.17	1.10	1.08748839	0.162

3704	1.32	25.00	Q3KRE3	Guanine nucleotide-binding protein subunit gamma	2	1.56	0.86	1.41	0.93	1.25	0.86094919	0.0747
3705	1.32	1.24	D3ZI42	Leucine-rich repeat and sterile alpha motif-containing 1	1	0.97	0.94	0.90	1.08	1.11	1.05936518	0.139
3706	1.32	2.10	Q68FU7	Ubiquinone biosynthesis monooxygenase C	1	1.04	1.03	0.97	1.07	0.97	0.91891288	0.0179
3707	1.32	3.18	D4A709	Tubulin, gamma complex-associated protein 6	1	0.75	1.49	1.67	1.17	1.13	1.08297505	0.044
3708	1.32	2.67	P17955	Nuclear pore glycoprotein p62	1	0.82	1.08	1.24	0.72	0.79	1.09126388	0.0383
3709	1.32	1.92	Q9P290	Solute carrier family 22 member 17	1	0.86	1.18	1.10	1.01	0.90	0.97171154	0.355
3710	1.31	3.47	M0R9I6	Aminomethyltransferase	1	1.03	1.02	1.01	0.96	1.01	0.92210312	0.0558
3711	1.31	3.25	Q4QQU9		1	0.96	0.96	1.11	0.88	1.03	0.99906469	0.993
3712	1.31	2.17	D3ZV64	Similar to cDNA sequence BC003331	1	0.86	1.32	1.28	1.14	1.33	0.97969189	0.503
3713	1.31	3.45	Q5M876	N-acyl-aromatic-L-amino acid amidohydrolase (carboxylate-forming)	1	0.74	0.75	1.02	1.15	0.86	1.0320408	0.86
3714	1.3	43.90	D3ZT07	Platelet glycoprotein Ib beta chain	25	0.92	0.95	0.92	1.08	0.95	0.98760086	0.757
3715	1.3	3.69	P30713	Glutathione S-transferase theta-2	1	1.21	1.14	1.03	1.14	1.10	1.03132569	0.709
3716	1.3	0.58	D3ZW27	Mitogen-activated protein kinase kinase kinase 5	1	1.36	1.38	1.21	1.07	1.38	1.01255481	0.518
3717	1.3	1.52	Q62920	PDZ and LIM domain protein 5	1	1.03	0.95	1.16	1.18	1.12	0.981255	0.61
3718	1.29	0.86	P51792	H(+)/Cl(-) exchange transporter 3	1	0.90	0.90	0.90	1.12	0.95	0.97704709	0.4
3719	1.29	2.10	Q63186	Translation initiation factor eIF-2B subunit delta	1	0.86	0.81	1.00	1.01	0.90	1.11033883	0.503
3720	1.29	4.83	Q4FZT8	SPRY domain-containing protein 4	1	0.98	1.03	1.32	1.05	1.25	0.95873234	0.276
3721	1.29	6.68	D3ZQB6	Cat eye syndrome chromosome region, candidate 5 homolog (Human) (Predicted)	2	0.72	0.77	0.78	0.73	0.81	0.90940825	0.138
3722	1.28	0.84	A0A0G2K6Z8	Pleckstrin homology domain-containing A7	1	0.70	0.55	0.57	0.65	0.82	1.04833514	0.0112
3723	1.28	5.75	D3ZRE3	Casein kinase I isoform	1	1.01	0.39	0.82	0.29	0.69	1.10956947	0.101
3724	1.28	2.63	G3V6R7	3-oxoacyl-[acyl-carrier-protein] synthase, mitochondrial	1	0.68	0.85	0.82	0.60	0.83	1.02179091	0.476
3725	1.28	5.58	Q9JK00	Sodium channel subunit beta-3	1	1.45	1.15	0.96	1.13	1.15	0.8882268	0.034
3726	1.27	63.62	F1MAQ5	Microtubule-associated protein	150	1.03	1.22	1.12	1.22	1.13	0.88515377	0.0774
3727	1.27	3.52	Q5BK47		3	0.90	1.00	0.98	0.91	1.06	1.07400447	0.49
3728	1.27	1.68	D4A997	HIV TAT specific factor 1 (Predicted)	1	0.69	1.43	1.41	1.02	1.02	1.01931503	0.553
3729	1.27	2.95	A0A0G2JVQ7	Neurotrophin-3	1	null	null	null	null	null	null	null
3730	1.27	3.35	A0JPI6	Palmitoyltransferase	1	0.97	1.02	0.80	1.03	1.34	0.97874165	0.299
3731	1.27	2.88	D3ZDE4		1	null	null	null	null	null	0.88209137	0.122
3732	1.27	2.77	Q66H06	Tetraspanin	1	1.20	1.18	0.90	0.90	1.01	1.05474921	0.0167
3733	1.26	2.24	F1LVX2	EH domain-binding protein	2	0.64	0.54	0.94	0.31	0.99	1.14393097	0.0609
3734	1.26	1.25	D3ZYJ5	GRAM domain-containing 1B	1	0.87	1.02	1.43	1.10	0.84	0.9893823	0.392
3735	1.26	14.29	P80432	Cytochrome c oxidase subunit 7C, mitochondrial	1	0.90	1.17	1.08	1.24	1.04	0.99295483	0.68
3736	1.25	32.86	D4A8B3	Calcium-transporting	50	1.12	0.88	0.64	0.92	0.82	1.0339024	0.494
3737	1.25	1.31	F1LMP9	Disabled homolog 2	1	1.39	1.11	0.15	0.72	0.70	0.98268436	0.526
3738	1.24	2.73	D3ZMY7	5'-nucleotidase, cytosolic II	2	1.18	1.19	1.06	1.25	1.07	1.07006616	0.186
3739	1.24	1.24	D3ZMG0	Unc-51-like autophagy-activating kinase 1	1	1.28	1.01	1.26	1.01	1.34	1.09581177	0.0165
3740	1.24	11.74	G3V9C1	Kv channel-interacting protein 4	3	1.08	1.08	1.24	1.21	1.24	0.90814842	0.0593
3741	1.24	5.23	A0A0G2K1U5	Protein FAM134A	1	1.18	0.89	1.17	0.82	0.89	1.24487424	0.276
3742	1.24	10.95	Q6AYQ1	Golgin subfamily A member	1	0.97	1.15	0.85	1.06	0.74	1.1011416	0.332
3743	1.23	2.21	D3ZFC5	Phospholipid-transporting ATPase	3	0.80	0.74	0.80	0.84	1.06	1.0510272	0.071
3744	1.23	1.37	Q9R120	Heat shock factor 2	1	null	null	null	null	null	0.96466505	0.504
3745	1.23	15.79	D4ADS4	Microsomal glutathione S-transferase 3	3	null	null	null	null	null	1.01052149	0.855
3746	1.22	1.80	A0A0G2KAD3	Rab proteins geranylgeranyltransferase component A 1	1	0.95	1.03	0.98	0.89	0.89	1.04058874	0.0624
3747	1.22	2.74	D3ZF19	Transmembrane protein 68	1	0.62	1.04	0.93	0.91	0.70	0.94337286	0.311
3748	1.21	9.23	D4AAF8	RNA-binding protein Nova-	3	0.97	1.19	0.90	0.79	0.58	1.00479417	0.886
3749	1.21	2.44	Q9JLD2	Neuroserpin	1	1.49	1.38	1.53	1.29	1.26	1.02150764	0.501

3750	1.21	1.81	A1A5S1	Pre-mRNA-processing factor 6	1	0.92	0.98	1.15	0.91	1.03	1.01938569	0.335
3751	1.21	3.25	Q499S6	Cathepsin F	1	0.64	0.62	1.01	1.49	0.90	0.93174043	0.00343
3752	1.21	4.18	D4ACN9	Solute carrier family 25 member 36	1	1.02	1.22	1.14	1.63	0.99	1.04818982	0.18
3753	1.21	10.24	Q4KLI4	Peptidyl-prolyl cis-trans isomerase	1	0.90	1.09	0.68	0.76	0.99	1.05489543	0.261
3754	1.2	1.46	A0A0G2K8J8	Family with sequence similarity 120B	1	0.84	1.01	0.84	0.39	0.37	1.00308231	0.745
3755	1.2	2.00	Q6UPE0	Choline dehydrogenase, mitochondrial	1	1.04	1.22	1.45	1.58	1.08	1.01199348	0.761
3756	1.19	2.97	Q9Z2Z8	7-dehydrocholesterol reductase	2	0.95	0.95	0.99	1.10	1.08	0.95521672	0.425
3757	1.19	4.12	D4A719	Sorting nexin 12	1	1.05	1.11	1.09	1.18	1.10	1.0669555	0.102
3758	1.18	4.13	Q9ES71	Dihydroxyacetone phosphate acyltransferase	2	0.85	1.13	0.86	1.33	1.01	1.02583592	0.363
3759	1.18	0.65	Q9WTR8	PH domain leucine-rich repeat protein phosphatase 1	1	1.08	1.17	1.10	1.16	0.99	1.09126388	0.0525
3760	1.18	4.48	Q6P775	FGF1 intracellular-binding protein	1	1.00	1.02	1.17	1.04	0.91	0.99774289	0.931
3761	1.18	2.56	D4A0X3	BTB (P	2	0.94	0.98	0.95	1.00	1.06	1.00025372	0.99
3762	1.17	8.66	B2GV72	Carbonyl reductase 3	2	0.82	0.76	0.70	0.73	0.84	0.98009942	0.337
3763	1.17	4.35	D3ZWU9	Required for meiotic nuclear division 5 homolog A	2	1.00	1.14	0.74	1.14	0.97	0.99171673	0.835
3764	1.17	1.80	F1MAN2	Transient receptor potential cation channel, subfamily C, member 6	1	0.98	0.74	0.41	0.67	0.82	1.03842714	0.19
3765	1.17	2.37	G3V7W8	Similar to RIKEN cDNA D230025D16Rik	1	1.04	1.02	0.89	1.12	0.90	0.98643781	0.582
3766	1.17	5.65	Q6IV57	RING finger protein 141	1	1.22	1.03	1.04	0.96	0.85	0.94618881	0.347
3767	1.17	10.83	M0R549	Hypothetical protein L	1	0.70	1.20	0.85	1.11	0.87	0.82645032	0.209
3768	1.17	4.72	D3ZSC2	Programmed cell death 2-	1	0.70	1.16	0.84	0.76	0.97	0.67361679	0.219
3769	1.16	3.26	B5DEJ5	Eefsec protein	2	1.06	0.82	0.70	0.83	0.95	1.01691564	0.528
3770	1.16	4.96	F1LU14	Sorting nexin	2	1.04	1.09	0.98	1.02	0.95	1.02925467	0.567
3771	1.16	6.11	Q9JHU6	Stathmin-3	1	0.81	1.08	1.15	0.94	0.88	0.97353179	0.635
3772	1.16	1.74	F1M9Q1	TBC1 domain family, member 5	1	1.13	0.85	0.97	1.15	1.01	1.07922824	0.105
3773	1.16	2.30	M0R6W5	Uncharacterized protein	1	0.98	1.01	1.25	0.98	0.80	1.08147476	0.121
3774	1.16	3.14	Q5BJX0	N-terminal Xaa-Pro-Lys N-methyltransferase 1	1	null	null	null	null	null	1.09961615	0.232
3775	1.15	38.40	D4A8X4	Carboxypeptidase E	21	1.72	6.67	3.10	2.99	4.45	0.9208257	0.0103
3776	1.15	4.43	D4AC38	Argonaute 1, RISC catalytic component	3	0.77	0.93	0.77	0.92	0.90	1.02037538	0.273
3777	1.15	2.20	F1LVB0	Uncharacterized protein	1	1.06	0.86	0.95	0.79	1.06	1.00695555	0.249
3778	1.15	1.56	Q66HL0	5' nucleotidase, ecto	1	1.01	1.01	1.16	1.24	1.28	0.96680712	0.11
3779	1.15	0.67	F1LQC5	Receptor protein serine/threonine kinase	1	0.96	1.26	1.16	0.86	1.25	1.33792756	0.0511
3780	1.14	1.20	A0A0G2K9E8	Unconventional myosin-Ie	1	0.68	0.86	0.70	0.93	0.82	1.01769129	0.463
3781	1.14	4.04	M0R9C5	REVERSED Coiled-coil domain-containing 160	2	1.03	0.96	0.82	1.06	1.10	null	null
3782	1.14	2.53	F1LNS2	Family with sequence similarity 45, member A	1	0.85	1.20	0.88	1.26	1.14	1.01614057	0.52
3783	1.13	5.40	D3ZVM5	Heat shock protein family A (Hsp70) member 12B	3	1.05	0.89	0.95	1.02	0.91	0.95105455	0.266
3784	1.13	2.03	Q63632	Solute carrier family 12 member 4	2	1.00	0.91	0.92	1.13	1.16	1.01571806	0.396
3785	1.13	0.85	D4A5F4	Hypothetical L	1	1.08	0.96	1.04	1.13	1.02	0.92787348	0.0819
3786	1.13	1.98	D3ZRJ0	AarF domain containing kinase 1 (Predicted)	1	0.65	0.56	0.67	0.81	0.81	1.07922824	0.0201
3787	1.12	1.14	D4A978	Phosphoinositide phospholipase C	2	0.90	1.14	1.10	1.22	0.96	1.0589981	0.0742
3788	1.12	2.01	D4ABT4	NADPH-dependent diflavin oxidoreductase 1	1	1.25	0.91	1.01	0.99	0.88	0.91509917	0.0923
3789	1.12	2.10	Q68FY4	Group specific component	1	0.69	0.41	0.50	0.37	0.91	0.98691655	0.214
3790	1.12	5.00	D3ZF12	Signal peptidase complex subunit 3	1	0.70	0.86	0.85	0.74	0.88	1.06962123	0.212
3791	1.12	8.03	D3ZFK5	RCG42279	1	0.74	0.96	0.81	0.78	0.86	0.83450928	0.0948
3792	1.11	62.42	Q6AYS2	Sideroflexin	25	1.10	1.09	0.95	0.98	0.99	0.87599832	0.0277
3793	1.11	5.64	Q5XIE8	Integral membrane protein	2	0.92	0.82	1.20	0.94	1.04	1.02264116	0.486
3794	1.11	1.16	A0A0G2K8X8	Pleckstrin homology domain-containing family G member 5	1	0.90	0.63	0.80	0.70	0.90	1.05826431	0.152
3795	1.1	6.31	P28492	Glutaminase liver isoform, mitochondrial	3	1.26	1.49	0.69	0.91	0.99	1.05731115	0.00763
3796	1.1	2.63	Q9ET50	Staufen double-stranded RNA-binding protein 1	1	0.83	0.56	0.95	0.91	0.80	0.9964781	0.922
3797	1.1	7.05	D3ZXF8	Mitochondrial ribosomal protein L43	1	0.90	0.79	0.90	0.92	1.22	0.94763277	0.124
3798	1.09	1.09	M0R4S7	REVERSED Coiled-coil domain-containing 13	1	null	null	null	null	null	null	null

3799	1.09	4.33	D4AC65	Cytochrome c oxidase assembly factor 7	1	0.98	0.64	0.93	0.81	0.88	1.00828256	0.872
3800	1.09	11.97	D3ZYU4	C	1	0.93	0.65	0.54	0.42	0.53	1.17446197	0.0254
3801	1.08	17.41	D4A9Z8	Chromatin-modifying protein 4B-like 1	6	null	null	null	null	null	0.98664295	0.395
3802	1.08	1.08	Q62886	Cys2/His2 zinc finger	2	1.20	1.21	1.18	1.25	1.51	1.04993499	0.0564
3803	1.08	1.18	F1LXW4	REVERSED MACC1, MET transcriptional regulator	1	0.85	0.97	1.49	1.06	0.87	null	null
3804	1.08	0.91	F1LNN8	Death-associated protein kinase 1	1	1.41	1.29	0.93	1.14	0.93	1.03433248	0.0477
3805	1.08	5.50	Q9EPJ3	28S ribosomal protein S26, mitochondrial	1	1.05	0.90	1.10	1.16	0.97	0.92210312	0.108
3806	1.07	61.88	P84082	ADP-ribosylation factor 2	26	0.73	0.92	0.75	0.79	0.79	0.92018765	0.0265
3807	1.07	7.65	Q63210	Guanine nucleotide-binding protein subunit alpha-12	5	0.77	0.95	0.99	1.00	1.02	1.06592062	0.351
3808	1.07	1.43	Q5FVM7	DnaJ homolog subfamily C member 16	1	0.92	1.20	0.97	0.95	0.98	1.04543256	0.0408
3809	1.07	1.24	D3ZQY0	Protocadherin gamma subfamily A, 7	1	1.12	1.16	1.19	0.95	1.01	1.10803235	0.173
3810	1.07	1.23	M0R7E6	Uncharacterized protein	1	1.15	1.12	1.05	1.20	0.99	1.02996835	0.212
3811	1.07	2.56	D4AAU4	Regulation of nuclear pre-mRNA domain-containing	1	0.93	1.13	1.11	0.96	1.04	0.95933061	0.16
3812	1.06	2.55	P40615	H/ACA ribonucleoprotein complex subunit 4	1	0.72	0.95	0.60	0.71	0.91	1.01578847	0.452
3813	1.06	0.64	D3ZYR0	Rho guanine nucleotide exchange factor 12	1	0.92	0.87	0.90	0.92	0.91	0.9961259	0.883
3814	1.06	2.71	A0A0H2UHZ4	Zinc finger Ran-binding domain-containing protein 2	1	0.86	1.02	1.03	0.97	1.02	1.00381959	0.933
3815	1.05	3.08	F1LRZ9	RUN domain-containing 3A	2	0.77	0.94	0.73	0.96	0.93	1.09505247	0.199
3816	1.05	3.10	D3ZJG4	Phosphofurin acidic cluster sorting protein 2	3	0.79	0.86	0.92	0.91	1.00	0.93607758	0.0235
3817	1.05	1.35	F1LT94	Rho/Rac guanine nucleotide exchange factor 18	1	0.89	0.86	0.79	0.70	0.64	1.02455682	0.256
3818	1.05	1.93	P35433	Amidophosphoribosyltransferase	1	1.02	0.97	1.08	1.11	1.09	1.04232125	0.115
3819	1.05	1.11	Q99P99	Histone deacetylase 4	1	0.80	0.75	0.81	0.77	0.96	0.99607066	0.913
3820	1.05	10.41	P0C6T3	Proline-rich protein 7	1	1.22	0.83	1.77	1.45	0.68	0.91891288	0.554
3821	1.05	8.21	Q569C0	Transmembrane protein 100	1	0.86	0.90	1.07	1.05	1.01	1.03986771	0.548
3822	1.04	5.86	A0A0G2K4S6	Engulfment and cell motility 1	4	1.09	0.69	0.70	0.60	0.83	1.09505247	0.0577
3823	1.04	3.57	Q5PPI4	Lysosome-associated membrane glycoprotein 5	1	1.05	1.27	1.15	0.98	1.21	1.08598186	0.0106
3824	1.04	5.05	Q5I034	Uncharacterized protein C12orf43 homolog	1	1.18	1.18	1.02	1.22	1.33	0.86633686	0.205
3825	1.04	5.10	Q498C8	Protein RER1	2	0.90	1.09	0.98	1.19	0.99	0.98561765	0.861
3826	1.03	2.47	D3ZYR1	F-BAR domain only protein	2	1.27	0.54	0.91	1.15	0.98	1.05008055	0.203
3827	1.03	4.52	Q6AY97	Coiled-coil domain-containing protein 91	2	1.12	0.91	1.03	0.90	1.13	1.06717739	0.218
3828	1.03	2.35	G3V6A8	Golgi autoantigen, golgin subfamily b, macrogolgin 1, isoform CRA_c	9	0.84	0.73	0.74	0.93	1.17	1.03018254	0.186
3829	1.03	1.08	D4A8A0	Carbamoyl-phosphate synthetase 2, aspartate transcarbamylase, and dihydroorotase	3	1.04	1.03	0.99	1.07	1.07	1.04015606	0.0383
3830	1.03	0.50	F1LM33	REVERSED Leucine-rich PPR motif-containing protein, mitochondrial	1	1.01	0.97	0.96	1.13	0.90	null	null
3831	1.03	2.06	Q02293	Protein farnesyltransferase subunit beta	1	0.79	0.95	1.04	0.92	0.90	1.06680759	0.015
3832	1.02	33.15	Q6AYU5	Poly(RC) binding protein 2	16	1.84	1.17	1.60	1.69	1.39	1.03011114	0.464
3833	1.02	8.44	Q63862	Myosin-11 (Fragments)	13	1.05	2.91	1.21	1.87	1.58	1.00229695	0.776
3834	1.02	3.60	Q641X3	Beta-hexosaminidase subunit alpha	2	0.98	1.15	0.86	0.96	0.90	0.93387412	0.143
3835	1.02	0.86	F1M3A0	Fibrosin-like 1	1	null	null	null	null	null	0.99484247	0.868
3836	1.02	2.53	Q5RK00	39S ribosomal protein L46, mitochondrial	1	null	null	null	null	null	0.9857543	0.788
3837	1.02	8.80	D3ZZV1	Presequence translocase-associated motor 16	1	1.13	1.18	1.00	1.14	0.96	1.1671581	0.0377
3838	1.02	7.56	G3V9W0	Centrin 2	1	0.70	0.80	0.70	0.54	0.76	0.91827616	0.067
3839	1.01	3.28	G3V798	Serine and arginine-rich-splicing factor 4	2	null	null	null	null	null	0.99137309	0.862
3840	1.01	1.52	G3V7J3	Leucine rich repeat and fibronectin type III domain containing 2	1	1.20	0.64	0.54	0.70	0.50	0.93828623	0.0676
3841	1.01	1.05	Q5BK10	Calpain-13	1	0.70	1.09	0.69	1.03	1.06	1.01860874	0.687
3842	1.01	3.34	Q32Q54	RCG37273, isoform CRA_a	1	0.80	0.61	0.93	0.73	1.29	1.07326029	0.104
3843	1.01	3.14	Q641X0	RCG41866, isoform CRA_b	1	0.96	0.83	0.93	0.95	0.89	0.97204836	0.287
3844	1.01	4.43	Q5XIU5	Proteasome inhibitor PI31 subunit	1	1.19	1.36	1.06	1.17	1.15	1.07773315	0.055

3845	1	9.06	G3V8L6	Potassium voltage-gated channel subfamily A member 6	3	0.80	0.92	0.90	1.17	0.57	0.96821544	0.592
3846	1	1.16	G3V7I7	REVERSED Leucine-rich repeat-containing 40	1	0.99	0.93	0.82	0.79	0.92	null	null
3847	1	3.26	G3V8E4	Similar to D7Wsu128e protein	1	0.91	1.07	0.88	0.90	0.85	0.96714225	0.516
3848	1	8.40	B2RYW4	39S ribosomal protein L53, mitochondrial	1	1.01	1.01	1.01	1.09	0.90	0.91130128	0.29
3849	0.99	2.14	Q5MYW4	REVERSED Zinc finger protein 667	2	1.05	1.05	1.11	1.09	1.31	null	null
3850	0.99	7.17	F1LPC7	Hepatoma-derived growth factor	1	0.93	0.98	1.09	0.92	0.91	0.88086937	0.116
3851	0.99	8.25	M0RAK4	Frataxin, mitochondrial	2	1.21	1.08	0.89	0.83	1.00	0.95660816	0.162
3852	0.99	1.28	A0A0G2JZ48	RCG56136, isoform CRA_a	1	1.20	0.70	0.67	0.90	0.90	1.04188786	0.515
3853	0.99	5.48	Q6MGA6	Proteasome subunit beta	1	0.77	0.95	0.95	0.60	0.85	1.08222465	0.105
3854	0.98	16.87	Q9JI66	Electrogenic sodium bicarbonate cotransporter 1	22	1.08	1.09	0.93	1.09	1.02	1.01129227	0.722
3855	0.98	2.67	A0A0G2JU01	Rho guanine nucleotide exchange factor 1	2	0.37	0.86	1.00	0.60	0.67	1.03268482	0.166
3856	0.98	0.79	Q02485	Voltage-dependent L-type calcium channel subunit alpha-1S (Fragment)	2	1.34	1.26	1.17	1.12	1.17	1.11573932	0.053
3857	0.98	0.94	D3ZN76	SEC16 homolog A, endoplasmic reticulum export factor	3	0.97	1.07	0.74	1.04	0.96	1.00264438	0.951
3858	0.98	1.81	G3V735	Angiomotin-like protein 2	2	null	null	null	null	null	0.941087	0.0509
3859	0.97	17.81	G3V997	Neuronal migration protein doublecortin	6	0.72	0.82	0.70	1.14	0.90	0.92851685	0.0381
3860	0.97	1.89	Q5XI67	F-box only protein 30	1	1.00	1.12	0.46	1.01	0.74	1.09581177	0.0979
3861	0.97	1.60	F1LM32	CMP-N-acetylneuraminate-beta-1,4-galactoside alpha-2,3-sialyltransferase	1	null	null	null	null	null	0.9962571	0.933
3862	0.97	4.25	D3ZXL9	Potassium channel tetramerization domain-containing 4	1	0.86	0.63	0.82	0.54	0.64	1.04811717	0.357
3863	0.97	2.62	O70150	Calcium/calmodulin-dependent protein kinase type 1B	1	1.26	1.53	1.77	1.24	1.14	1.06245372	0.284
3864	0.97	3.18	A0A0H2UHN	Isopentenyl-diphosphate Delta-isomerase 1	1	1.39	0.93	1.38	1.26	1.22	0.99267957	0.88
3865	0.97	4.48	B3DMA1	Ataxin 2-like	2	0.96	0.90	1.01	1.08	1.03	0.9857543	0.752
3866	0.96	6.28	F1M754	Mitogen-activated protein kinase kinase kinase kinase	8	1.19	1.14	1.21	1.00	1.17	0.92723055	0.21
3867	0.96	1.11	B2RYM3	Inter-alpha trypsin inhibitor, heavy chain 1	1	0.95	1.10	0.95	1.15	0.95	1.03777954	0.0431
3868	0.96	5.51	Q642E6	Tripeptidyl peptidase I	3	0.77	1.06	1.04	1.01	1.03	1.04695541	0.172
3869	0.96	0.00	A0A0H2UHF	Alpha-1-acid glycoprotein	0	30.20	11.48	7.87	2.61	0.86	1.10803235	0.187
3870	0.96	5.33	Q9QYW3	M	1	0.86	0.88	0.94	1.04	1.16	0.99171673	0.606
3871	0.95	4.80	F1LRK4	G-rich RNA sequence-binding factor 1	2	1.36	2.07	1.92	1.87	1.16	1.04275483	0.161
3872	0.95	3.43	Q6MG73	Complement C2	2	0.95	0.83	1.13	1.15	0.79	0.97481476	0.291
3873	0.95	3.96	Q923W4	Hepatoma-derived growth factor-related protein 3	1	1.05	1.16	1.06	0.86	0.95	0.8438158	0.0234
3874	0.95	3.19	Q642G4	Peroxisomal membrane protein PEX14	1	0.97	1.04	1.06	0.95	0.95	0.94520555	0.0396
3875	0.95	2.79	O08835	Synaptotagmin-11	1	0.81	0.93	0.86	0.83	0.95	1.09581177	0.0122
3876	0.95	2.13	A0JN29	Limb and neural patterns	1	0.84	0.82	0.79	0.91	0.76	0.89502507	0.181
3877	0.95	0.65	B1WC16	BCL2-associated transcription factor 1	1	0.97	1.06	0.93	0.85	1.00	0.96607025	0.249
3878	0.94	25.76	Q5HZY2	GTP-binding protein	4	0.97	1.03	0.94	0.94	0.90	1.06245372	0.319
3879	0.94	5.22	Q63484	RAC-gamma serine/threonine-protein kinase	2	1.05	0.98	0.86	1.10	0.94	1.08372597	0.0041
3880	0.94	1.86	Q10758	REVERSED Keratin, type II cytoskeletal 8	1	null	null	null	null	null	0.9627279	0.615
3881	0.94	2.50	O70617	Inward rectifier potassium channel 13	1	0.59	0.69	0.76	0.94	0.93	1.04601243	0.0243
3882	0.93	3.02	G3V7Z4	Glia-derived nexin	1	1.16	1.06	1.01	1.20	0.89	0.96747749	0.112
3883	0.93	1.08	Q6Q3F5	SID1 transmembrane family member 1	1	3.66	1.50	3.60	2.83	2.99	1.00625085	0.847
3884	0.92	0.97	D3ZUP3	Very long-chain acyl-CoA synthetase	1	null	null	null	null	null	1.00625085	0.847
3885	0.92	3.25	Q9JKM5	Sphingosine 1-phosphate receptor 5	1	1.00	0.74	0.77	0.96	0.74	0.88331505	0.19
3886	0.92	3.14	P62603	Tripartite motif-containing protein 26	2	0.64	0.56	0.57	0.28	0.36	0.90877812	0.0423
3887	0.92	2.14	D4A7F5	Kelch repeat and BTB (P	1	1.18	1.28	0.92	1.19	0.86	1.01578847	0.335
3888	0.92	3.45	P31325	Phosphorylase b kinase gamma catalytic chain, liver/testis isoform	1	0.90	0.82	0.95	0.65	0.89	1.04398429	0.243

3889	0.91	0.69	D3ZLD5	REVERSED Golgin A3	1	1.32	0.86	0.99	1.26	1.28	null	null
3890	0.91	3.89	F1M065	Junctional adhesion molecule 2	1	0.90	1.17	0.95	0.96	0.91	1.18838311	0.476
3891	0.91	8.26	D3ZU88	Similar to RIKEN cDNA 1700025G04 gene	1	1.19	1.18	1.20	1.38	1.22	1.20246925	0.029
3892	0.9	0.00	Q9JIM0	Double-strand break repair protein MRE11	0	0.80	0.94	1.08	1.22	0.99	0.96499943	0.32
3893	0.9	3.00	Q5XIP9	Transmembrane protein 43	1	1.19	0.82	1.26	1.12	1.14	0.99781897	0.946
3894	0.9	6.94	A0A0G2JVA7	Eukaryotic translation initiation factor 1A	1	0.97	1.07	1.17	0.99	1.05	1.04622997	0.219
3895	0.9	2.29	Q63054	Islet cell autoantigen 1	1	0.78	1.01	0.92	1.02	0.87	0.94422331	0.116
3896	0.89	2.58	D4A9T0	Cask-interacting protein 2	2	0.22	0.35	0.38	0.17	0.30	0.92018765	0.0325
3897	0.89	2.66	Q5U209	Ribonuclease H2 subunit A	1	null	null	null	null	null	1.03011114	0.281
3898	0.89	1.31	Q5U2Y6	Tuftelin-interacting protein 11	1	1.58	0.92	1.15	1.31	1.53	1.01621101	0.646
3899	0.89	1.24	D3ZWU1	REVERSED Bromodomain containing 3 (Predicted), isoform CRA_b	1	null	null	null	null	null	null	null
3900	0.89	2.75	Q642A4	REVERSED UPF0598 protein C8orf82 homolog	1	null	null	null	null	null	null	null
3901	0.88	2.14	D3ZXB9	Sorting nexin 19	2	0.83	0.87	0.92	0.94	0.90	1.00475935	0.886
3902	0.88	0.82	Q2I6B1	REVERSED V-type proton ATPase subunit a	1	null	null	null	null	null	null	null
3903	0.88	1.39	D4IGX4	Alpha-(1,6)-fucosyltransferase	1	0.89	0.84	0.68	0.77	0.79	1.02363402	0.117
3904	0.88	0.69	Q6IE24	Inactive ubiquitin carboxyl-terminal hydrolase 54	1	1.24	0.70	0.92	0.65	1.06	1.06201195	0.0126
3905	0.88	6.55	M0RBI3	N-alpha-acetyltransferase 50-like	1	0.76	0.93	0.91	1.14	0.74	0.9938982	0.812
3906	0.88	3.25	G3V7M5	Choline-phosphate cytidylyltransferase B	1	1.28	1.75	1.03	1.12	1.37	0.96453133	0.0718
3907	0.88	3.52	Q9EPH2	MARCKS-related protein	1	1.69	1.33	1.71	0.64	0.69	0.86934456	0.145
3908	0.87	47.98	A0A0G2K943	AP-2 complex subunit alpha-2	58	1.27	0.69	1.07	1.04	1.26	0.9843887	0.661
3909	0.87	3.46	Q4VBH2	tRNA nucleotidyl transferase 1	2	0.90	0.90	1.18	0.82	1.13	1.04993499	0.1
3910	0.87	3.44	A0A0G2K808	ArfGAP with SH3 domain, ankyrin repeat and PH domain 2	3	1.01	0.84	0.91	1.10	0.98	1.03196926	0.177
3911	0.87	0.00	D4AEG7	TBC1 domain family, member 13	0	0.86	0.77	0.62	0.49	0.94	1.10190512	0.0766
3912	0.87	1.99	F1M670	REVERSED Formyl peptide receptor 2	1	null	null	null	null	null	null	null
3913	0.87	7.86	A0A0G2K0T8	Family with sequence similarity 25, member A	2	null	null	null	null	null	1.0238469	0.691
3914	0.86	2.35	Q9JID1	Programmed cell death protein 4	1	1.15	1.12	1.20	1.14	1.24	1.04521519	0.216
3915	0.86	1.29	Q5PPG5	Chga protein	1	0.95	0.94	1.09	1.00	1.14	0.96741043	0.248
3916	0.86	2.63	P53987	Monocarboxylate transporter 1	1	1.01	1.03	1.13	1.00	0.89	1.09126388	0.0144
3917	0.85	9.32	D3ZNK1	Metaxin 3	4	0.93	0.93	0.95	1.01	0.95	0.95145017	0.0299
3918	0.85	2.22	Q9WVC1	Slit homolog 2 protein (Fragment)	1	1.09	0.37	0.72	0.83	0.85	1.10496449	0.064
3919	0.85	3.16	G3V674	Claudin domain containing 1, isoform CRA_b	1	0.95	1.05	0.96	1.14	0.87	0.93465122	0.0744
3920	0.85	4.58	Q641X9	39S ribosomal protein L9, mitochondrial	1	1.11	1.18	0.96	1.00	0.69	1.13288389	0.0541
3921	0.84	5.39	P19969	Gamma-aminobutyric acid receptor subunit alpha-5	3	1.07	1.24	1.01	0.87	0.87	1.10726458	0.224
3922	0.84	0.85	F1LX47	IQ motif-containing with AAA domain 1	1	null	null	null	null	null	null	null
3923	0.84	3.29	Q80W98	Small glutamine-rich tetratricopeptide repeat-containing protein beta	1	0.81	1.18	0.97	0.99	0.95	0.90437938	0.218
3924	0.84	0.94	F1LN42	Tensin 1	1	0.95	1.15	0.89	1.14	0.99	1.13131446	0.0844
3925	0.83	1.53	Q5U329	REVERSED Anion exchange protein	2	null	null	null	null	null	null	null
3926	0.83	4.54	F7EZZ0	Death-associated protein 3	2	1.02	0.77	0.77	0.74	0.83	1.07177346	0.0202
3927	0.83	0.91	D4A927	Transforming, acidic coiled-coil-containing protein 1	1	1.03	1.14	0.97	1.10	0.91	0.97758903	0.366
3928	0.83	1.46	A0A0G2JZT0	P2X purinoceptor 7	1	1.09	0.90	1.00	0.95	1.05	1.02612039	0.204
3929	0.83	5.74	Q3MIE9	Spermine synthase	1	1.15	1.26	1.08	1.29	1.04	1.07698638	0.241
3930	0.83	3.67	D3ZPG5	Ubiquitin carboxyl-terminal hydrolase 30	1	0.97	1.22	1.18	1.20	1.18	1.01220394	0.517
3931	0.83	4.71	A0A0A0MXX	3-hydroxybutyrate dehydrogenase type 2	1	1.38	1.07	1.26	1.28	1.21	1.03598276	0.402
3932	0.83	5.75	B2RYU6	Trafficking protein particle complex subunit 2-like protein	1	1.28	0.82	1.05	1.04	1.02	0.97386925	0.816
3933	0.83	5.53	Q5BJQ5	GTP-binding protein Rit2	1	0.94	0.92	1.10	0.81	0.98	1.04702798	0.283

3934	0.82	1.56	A0A0A0MP8	Peroxisomal targeting signal 1 receptor-like	1	null	null	null	null	null	1.0807254	0.0769
3935	0.81	3.83	F1LNZ2	Protein numb homolog	3	1.20	0.98	1.02	1.21	1.04	0.96326189	0.571
3936	0.81	2.17	P97564	Glycerol-3-phosphate acyltransferase 1, mitochondrial	2	0.96	1.20	0.92	0.56	0.84	0.99649191	0.82
3937	0.81	2.29	F7F3Z1	Lectin, mannose-binding 2-like	1	0.86	0.86	0.87	0.76	0.80	1.02555154	0.134
3938	0.8	4.27	D3ZYS1	Purine-rich element-binding protein G	2	0.95	0.92	0.91	0.89	0.94	0.97150949	0.329
3939	0.8	1.25	G3V9L1	Testis expressed gene 2	1	0.94	0.97	0.92	1.06	0.86	1.10880064	0.0682
3940	0.8	1.30	D3ZTY5	REVERSED Usher syndrome 1G	1	0.74	0.76	0.88	1.33	0.96	null	null
3941	0.8	1.18	D4A831	Adhesion G protein-coupled receptor B3	2	0.97	0.98	0.91	0.92	1.00	0.94691052	0.127
3942	0.8	4.15	D3ZX74	Coenzyme Q10 homolog A (Yeast) (Predicted), isoform CRA_b	1	0.98	1.01	1.03	1.04	1.04	0.88454044	0.0125
3943	0.79	2.07	F1M8L9	Rap guanine nucleotide exchange factor 1	3	0.78	0.96	0.90	1.00	0.79	1.0669555	0.121
3944	0.79	0.94	P51794	H(+)/Cl(-) exchange transporter 4	1	0.85	0.86	0.76	0.91	0.89	1.07922824	0.058
3945	0.79	0.00	Q06486	Casein kinase I isoform	0	0.95	1.09	0.96	0.99	0.94	0.98609599	0.832
3946	0.79	4.09	D4A5J2	REVERSED 5',3'-nucleotidase, mitochondrial	1	1.24	1.43	0.86	1.36	0.97	null	null
3947	0.79	1.36	A0A0G2JSQ9	Proline-, glutamic acid- and leucine-rich protein 1	1	1.20	1.12	1.17	1.03	1.46	1.0179735	0.465
3948	0.79	2.73	Q4V897	Coiled-coil domain-containing protein 90B, mitochondrial	1	0.69	0.91	0.87	0.77	0.71	0.99815101	0.939
3949	0.79	1.23	Q5PXK5	REVERSED Potassium voltage-gated channel subfamily C member 3	1	null	null	null	null	null	null	null
3950	0.79	6.90	P60042	Somatostatin	1	5.70	3.08	2.38	0.95	1.14	0.93516964	0.208
3951	0.78	1.25	D4A1U8	Uncharacterized protein	1	2.07	2.19	0.51	1.14	0.63	1.03375908	0.156
3952	0.78	1.64	D3ZTL2	Rab effector MyRIP	1	2.05	1.63	0.67	0.98	0.91	0.98636944	0.457
3953	0.78	10.40	B2RYS9	Similar to RIKEN cDNA 0610038D11 (Predicted), isoform CRA_b	2	0.82	1.10	0.98	1.18	0.83	0.83277577	0.043
3954	0.77	3.91	Q9Z2X5	REVERSED Homer protein homolog 3	2	null	null	null	null	null	null	null
3955	0.77	7.48	Q03344	ATPase inhibitor, mitochondrial	1	0.36	1.39	1.00	1.32	1.37	1.13053057	0.0801
3956	0.77	0.00	D3ZWH2	Junctophilin 3	0	1.04	1.05	0.91	1.03	1.07	0.94717309	0.0733
3957	0.77	1.70	G3V7Q4	Protein tyrosine phosphatase, non-receptor	1	0.95	1.04	0.97	0.85	0.72	0.9959602	0.845
3958	0.77	1.19	D4A3X9	Zinc finger protein 192	1	null	null	null	null	null	0.98999971	0.558
3959	0.77	4.00	A0A0G2K946	SPARC/osteonectin, cwcv and kazal-like domains proteoglycan 2	2	1.06	1.00	1.15	1.02	1.12	1.12974722	0.247
3960	0.77	2.15	M0RD63	RAN-binding protein 3	1	0.77	1.05	0.78	0.79	0.79	1.32133841	0.0355
3961	0.77	3.72	D3ZZU4	Putative uncharacterized protein RGD1564549_predicted	1	1.03	0.99	1.09	1.03	1.00	0.91700404	0.0614
3962	0.77	2.24	M0R565	WD repeat domain 82	1	1.08	0.95	0.95	1.14	1.10	0.89254697	0.21
3963	0.77	3.61	G3V9I9	Splicing regulatory glutamine/lysine-rich protein 1	1	1.03	1.20	1.24	1.06	1.31	0.97211574	0.871
3964	0.76	15.48	Q9JIR4	Regulating synaptic membrane exocytosis protein 1	19	0.56	0.94	0.70	0.47	0.70	0.98288873	0.074
3965	0.76	1.17	Q63615	Vacuolar protein sorting-associated protein 33A	1	0.89	0.85	1.00	1.00	0.90	1.00982129	0.735
3966	0.76	4.49	D3ZDJ3	ELM	2	1.08	1.02	0.95	1.21	1.22	1.06799138	0.174
3967	0.76	1.02	A0A0G2KA1	C-type lectin domain family 16, member A	1	0.67	0.84	0.49	0.95	0.82	1.04008397	0.0686
3968	0.76	4.74	D3ZRB3	RCG21806	1	1.01	1.00	1.14	1.09	1.03	1.04181564	0.173
3969	0.76	2.24	D3ZIL5		1	null	null	null	null	null	0.91636865	0.114
3970	0.75	0.62	Q9JMK8	Vacuolar protein sorting-associated protein 54	1	null	null	null	null	null	0.99710684	0.908
3971	0.75	1.44	F1LP46	ATP-dependent RNA helicase SUPV3L1, mitochondrial	1	0.92	1.22	0.97	0.96	0.98	1.0243438	0.159
3972	0.75	5.32	Q6AYD9	Nucleoside diphosphate-linked moiety X motif 19	2	0.61	0.67	0.58	0.79	0.79	0.92916067	0.0315
3973	0.75	3.14	D4A3T5	Complement C1q-like 3	1	0.83	1.26	1.26	0.82	0.85	0.95959663	0.416
3974	0.75	8.08	A0A0G2KAY	Selenoprotein 15	1	0.97	1.12	1.15	0.90	1.04	1.02044611	0.462
3975	0.75	2.42	M0RAS3	Coiled-coil domain-containing 137	1	null	null	null	null	null	1.1320989	0.0284
3976	0.74	5.84	A0A0G2K9S3	Protein-serine/threonine kinase	4	0.74	1.18	0.88	1.04	1.33	0.99295483	0.615

3977	0.74	0.99	A0A0G2JWJ5	REVERSED Copper-transporting ATPase 2	2	0.87	1.00	0.90	1.05	0.95	null	null
3978	1.71	1.54	P07687	Epoxide hydrolase 1	1	1.54	1.01	0.85	1.74	1.37	null	null
3979	0.74	1.83	D3ZTT8	REVERSED Polypeptide N-acetylgalactosaminyltransferase	1	null	null	null	null	null	null	null
3980	0.74	4.00	Q5XIM4	ATP synthase subunit s, mitochondrial	1	0.84	1.24	0.88	1.13	0.87	1.01874996	0.504
3981	0.74	2.43	D4A9W3	Similar to hypothetical protein FLJ20950	1	null	null	null	null	null	1.01564766	0.627
3982	0.74	0.00	P05964	Protein S100-A6	0	1.03	1.42	1.96	1.20	1.14	1.1234999	0.142
3983	0.74	3.43	Q5FVL6	Tetraspanin-13	1	1.09	1.26	1.10	1.09	1.16	0.97860598	0.684
3984	0.74	4.28	Q9Z122	Fatty acid desaturase 2	1	0.63	0.64	0.77	0.90	0.65	0.95813444	0.161
3985	0.73	39.48	P62716	Serine/threonine-protein phosphatase 2A catalytic subunit beta isoform	15	null	null	null	null	null	0.95727146	0.0141
3986	0.73	4.28	Q4KLL7	Vacuolar protein sorting 4 homolog B	3	0.78	0.67	0.95	0.91	0.95	1.03979563	0.159
3987	0.73	1.72	D4A4J0	SPT16 homolog, facilitates chromatin-remodeling	2	0.39	0.16	0.10	0.17	0.10	0.97819907	0.524
3988	0.73	3.70	Q99ND9	RWD domain-containing protein 1	1	null	null	null	null	null	1.09353546	0.00605
3989	0.73	6.04	P19132	Ferritin heavy chain	1	0.39	0.36	0.28	0.82	0.34	0.98821715	0.894
3990	0.72	3.82	G3V6J5	Cyclin-dependent kinase 17	3	0.80	1.32	1.27	1.02	1.50	1.00961133	0.715
3991	0.72	1.28	F1M4T7	Transglutaminase 6	1	null	null	null	null	null	1.16231411	0.138
3992	0.72	1.77	A0A0G2K713	REVERSED Hydroxyacid oxidase 2	1	1.16	1.15	1.16	0.95	1.18		
3993	0.72	0.98	D3ZQK4	Thioredoxin domain-containing 16	1	null	null	null	null	null	0.96586938	0.117
3994	0.72	2.65	D4A255	TBC/LysM-associated domain-containing 1	1	0.91	1.14	0.83	1.51	1.27	1.00226222	0.979
3995	0.72	4.05	B1WC84	Canopy 4 homolog (Zebrafish)	1	1.05	0.86	0.88	0.54	0.77	1.00124844	0.966
3996	0.71	5.61	A0A0G2KA1	Kinesin-like protein KIF1B	9	0.73	0.82	0.74	0.77	0.92	0.99199173	0.778
3997	0.71	2.84	Q9QUL0	Prolactin-3C1	1	1.02	1.25	1.53	1.19	1.09	1.01677467	0.501
3998	0.71	1.52	Q2YDU8	Protein spinster homolog 1	1	1.19	1.38	0.87	1.18	0.92	0.82359102	0.339
3999	0.71	4.56	A0A0G2K2F4	Serine and arginine-rich-splicing factor 12	1	0.88	0.85	0.85	0.70	0.90	0.85916076	0.0472
4000	0.71	4.62	M0RA39	Matrix-remodeling-associated 7	1	0.85	0.95	0.91	0.90	0.86	0.98931373	0.654
4001	0.71	10.25	F1M9I6	Tetraspanin	2	1.09	1.25	1.09	1.18	1.12	0.94402698	0.172
4002	0.7	4.57	P47197	RAC-beta serine/threonine-protein kinase	3	0.86	0.95	0.84	1.12	0.98	0.97184625	0.586
4003	0.7	2.01	B2GUW4	Exdl2 protein	1	0.81	0.93	1.06	0.86	1.04	1.08748839	0.0785
4004	0.7	0.85	F1LMI3	Cadherin 3	1	null	null	null	null	null	1.02896934	0.0598
4005	0.7	4.01	Q06000	Lipoprotein lipase	1	0.76	1.13	0.82	1.27	0.81	0.96159413	0.394
4006	0.69	3.31	O70513	Galectin-3-binding protein	1	0.95	0.87	0.98	0.90	0.75	1.00849225	0.873
4007	0.69	1.23	D3ZTT7	Sad1 and UNC84 domain-containing 2	1	null	null	null	null	null	0.92594602	0.213
4008	0.69	3.68	F1LU03	REVERSED Uncharacterized protein	1	1.26	1.18	1.26	1.56	1.18	null	null
4009	0.69	3.66	Q5M7T4	Protein YIPF4	1	1.01	1.14	1.29	0.95	1.13	0.95933061	0.174
4010	0.69	0.00	P32577	Tyrosine-protein kinase	0	1.54	1.92	1.09	1.06	0.83	0.98582263	0.796
4011	0.69	1.83	A0A0H2UI14	F-box/LRR-repeat protein	1	1.34	1.53	1.06	1.25	1.25	1.00067466	0.989
4012	0.69	1.56	Q5U2Y3	REVERSED MAGUK p55 subfamily member 7	1	0.86	1.12	0.70	0.89	0.87	null	null
4013	0.69	1.53	D4AAS1	G protein-coupled receptor 162	1	0.99	0.87	1.08	0.95	0.86	1.04224901	0.338
4014	0.69	9.72	P37200	REVERSED Arrestin-E (Fragment)	1	null	null	null	null	null	null	null
4015	0.68	1.16	A0A0G2K988	Dishevelled-associated activator of morphogenesis	2	0.33	0.26	0.14	0.22	0.26	0.99075483	0.484
4016	0.68	1.22	A0A0U1RRS	UPF2 regulator of nonsense transcripts homolog (yeast) (Fragment)	1	null	null	null	null	null	0.94618881	0.152
4017	0.68	1.68	G3V747	REVERSED Gap junction protein	1	null	null	null	null	null	null	null
4018	0.68	1.44	Q6XQG8	REVERSED Carbohydrate sulfotransferase 7	1	null	null	null	null	null	null	null
4019	0.67	10.50	F1LWK7	Actin-binding LIM protein 1 (Fragment)	4	1.67	1.16	1.18	0.83	1.18	0.92018765	0.0127
4020	0.67	2.49	D3ZZB2	Peroxisomal biogenesis factor 1	3	0.58	0.36	0.55	0.27	0.54	1.03282799	0.379
4021	0.67	0.57	D3ZBP4	[F-actin]-methionine sulfoxide oxidase MICAL1	1	0.96	1.10	0.99	1.11	1.20	1.00332569	0.844
4022	0.67	1.13	Q04931	FACT complex subunit SSRP1	1	1.63	1.25	1.22	0.99	0.98	1.02172008	0.559
4023	0.67	1.10	F7FDM3	REVERSED Mitochondrial translational initiation factor 2	1	1.03	0.95	1.28	0.95	1.06	null	null

4024	0.67	3.82	P97577	Fasciculation and elongation protein zeta-1	2	1.08	0.99	0.86	0.94	1.03	0.97231791	0.495
4025	0.67	1.38	G3V8S2	REVERSED SHC-transforming protein 1	1	0.60	1.16	1.31	2.13	0.54	null	null
4026	0.66	7.84	A0A0G2JUG4	Regulator of G-protein-signaling 6	3	1.24	1.27	1.34	1.56	1.15	1.07400447	0.0482
4027	0.66	1.00	D3Z991	Ribosomal protein S6 kinase C1	1	1.07	0.47	0.90	0.65	0.73	1.12116608	0.0596
4028	0.66	0.74	D3Z854	REVERSED Similar to KIAA0406-like protein (Predicted)	1	null	null	null	null	null	null	null
4029	0.66	3.17	Q4G075	Leukocyte elastase inhibitor A	1	0.72	0.95	0.90	0.91	1.32	1.06201195	0.0006
4030	0.66	1.87	O08837	Cell division cycle 5-like protein	1	1.00	1.05	1.01	1.01	1.09	1.02150764	0.642
4031	0.66	2.16	G3V8V1	Granulin, isoform CRA_c	1	0.90	0.89	1.43	0.86	1.07	1.15029189	0.0208
4032	0.65	5.25	P63035	Cytohesin-2	2	1.07	1.05	0.90	0.83	0.83	0.97826688	0.174
4033	0.65	1.97	Q64620	Serine/threonine-protein phosphatase 6 catalytic subunit	1	0.81	0.85	0.81	0.72	1.04	1.19416319	0.312
4034	0.65	2.71	D4A1X2	Exosome component 10	1	0.64	0.96	0.85	0.93	0.86	0.98486644	0.799
4035	0.65	2.97	M0R8K1	E3 ubiquitin-protein ligase RNF187	1	1.10	1.09	1.01	1.13	1.02	0.88945899	0.012
4036	0.64	1.05	A0A0G2K101	Leucine zipper protein 1	1	0.93	0.96	1.07	0.95	1.06	1.02101213	0.099
4037	0.64	2.21	D3ZW19	Leucine-rich repeat and Ig domain-containing 3	1	1.41	1.20	0.77	1.21	1.00	0.97697937	0.644
4038	0.64	0.97	Q6XUX2	Dual serine/threonine and tyrosine protein kinase	1	1.13	1.07	0.96	0.84	1.01	1.00430676	0.895
4039	0.64	0.88	A0A0G2JUV5	Coiled-coil domain-containing 40	1	1.37	1.26	1.28	1.32	1.02	0.95899819	0.23
4040	0.64	2.01	Q569C9	Golgi phosphoprotein 3	1	1.53	0.83	1.96	1.33	0.79	1.04637502	0.123
4041	0.64	0.52	D4A1D3	Sacsin molecular chaperone	2	0.83	0.69	0.55	0.80	0.63	1.06754731	0.0617
4042	0.63	1.10	P51556	Diacylglycerol kinase alpha	1	1.28	1.01	1.16	0.98	1.32	1.05431064	0.0939
4043	0.63	1.79	P35560	REVERSED ATP-sensitive inward rectifier potassium channel 1	1	2.91	1.29	1.38	1.42	1.61	null	null
4044	0.63	2.16	P41034	REVERSED Alpha-tocopherol transfer protein	1	null	null	null	null	null	null	null
4045	0.63	0.96	M0R979	Thrombospondin 1	1	0.88	0.90	0.90	0.82	0.83	1.01776184	0.663
4046	0.63	2.78	D3ZFC8	EF-hand calcium-binding domain 9	1	null	null	null	null	null	0.96580243	0.359
4047	0.62	1.70	Q70AM4	Kinesin 13B	2	0.95	0.99	0.60	0.72	0.72	1.05496856	0.00247
4048	0.62	0.81	Q6F6B3	Protein TANC1	2	1.02	1.01	1.00	1.14	1.16	1.04731832	0.216
4049	0.62	3.23	Q5U2S4	REVERSED Leucine rich repeat containing 2	1	null	null	null	null	null	null	null
4050	0.62	9.30	Q80W96	Iron-sulfur cluster assembly 1 homolog, mitochondrial	1	0.65	0.80	0.71	0.82	0.60	0.97576119	0.667
4051	0.62	1.69	F1LPL9	Stromelysin-1	1	1.17	2.13	1.31	0.09	1.18	1.07549439	0.0889
4052	0.62	1.01	M0RDA4	REVERSED EPH receptor B4	1	null	null	null	null	null	null	null
4053	0.62	2.91	Q5EB90	Polymerase (RNA) II (DNA directed) polypeptide C, 33kDa	1	2.47	1.07	1.74	0.69	1.80	0.99716904	0.947
4054	0.62	0.92	P14925	REVERSED Peptidyl-glycine alpha-amidating monooxygenase	1	null	null	null	null	null	null	null
4055	0.62	4.37	D3ZZM9	Centriole, cilia and spindle-associated protein	1	0.99	1.02	1.05	1.03	1.09	0.98105097	0.195
4056	0.62	3.42	D3ZAR8	REVERSED	1	0.90	0.95	1.09	0.77	0.89	null	null
4057	0.61	12.55	A0A0G2K2V7	Tyrosine-protein kinase	7	0.77	0.92	0.84	0.97	0.73	1.01059154	0.724
4058	0.61	3.91	D4A346	ArfGAP with coiled-coil, ankyrin repeat and PH domains 3	2	null	null	null	null	null	0.89564567	0.266
4059	0.61	1.04	Q8CF82	ATP-binding cassette sub-family A member 5	1	1.00	0.79	0.74	2.19	0.55	1.1352421	0.0267
4060	0.61	1.68	Q0H8V1	DNAJC18	1	null	null	null	null	null	1.05015334	0.0805
4061	0.61	4.76	Q4V7D7	Pre-mRNA-splicing factor RBM22	1	0.54	1.18	1.18	0.66	0.69	1.04891662	0.237
4062	0.61	2.63	Q5PQN9	39S ribosomal protein L38, mitochondrial	1	0.64	0.83	0.90	1.29	0.89	0.95000038	0.477
4063	0.61	1.94	D3ZDC2	Solute carrier family 43 member 2	1	0.77	0.77	0.81	0.93	0.90	1.11573932	0.355
4064	0.61	6.03	D4A6F2	Ly6/neurotoxin 1	1	1.01	1.10	0.92	1.04	1.04	1.03562378	0.368
4065	0.6	0.94	Q99P77	REVERSED Nucleolar GTP-binding protein 1	1	null	null	null	null	null	null	null
4066	0.6	1.25	Q7TT51	Lengsin	1	null	null	null	null	null	1.03018254	0.626
4067	0.6	0.71	F1LVT9	REVERSED Similar to novel protein	1	null	null	null	null	null	null	null
4068	0.6	1.34	Q66HC5	Nuclear pore complex protein Nup93	1	1.04	0.92	1.04	0.82	1.13	1.03698858	0.0818

4069	0.6	2.16	D3ZGL1	Rho GTPase-activating protein 25	1	0.95	0.90	0.85	0.95	1.10	1.15428542	0.0948
4070	0.6	0.53	Q7TP54	REVERSED Protein FAM65B	1	0.55	0.63	0.55	0.76	0.72	null	null
4071	0.6	1.21	D3ZIC4	Protein phosphatase 1 regulatory subunit	1	1.10	1.06	0.90	0.85	1.02	1.05826431	0.0032
4072	0.6	1.05	Q3MID9	REVERSED	1	null	null	null	null	null	null	null
4073	0.6	4.79	D4A4A9	Mitochondrial ribosomal protein L19	1	1.16	1.16	1.25	1.10	1.08	1.15588671	0.00384
4074	0.6	6.97	Q920P0	L-xylulose reductase	2	1.03	1.03	1.02	1.10	0.93	0.93984842	0.159
4075	0.6	1.89	F1M9C0	Mitogen-activated protein kinase-activated protein kinase 2	1	null	null	null	null	null	1.03763568	0.217
4076	0.59	2.03	Q5U330	Guanylate cyclase 1, soluble, alpha 3	1	1.00	0.73	1.47	1.01	0.91	1.06459154	0.362
4077	0.59	1.09	G3V7Q7	IQ motif containing GTPase activating protein 1 (Predicted), isoform CRA_b	2	0.98	0.95	0.92	1.12	0.98	1.06569899	0.106
4078	0.59	0.88	D3ZM57	REVERSED Golgi integral membrane protein 4	1	null	null	null	null	null	1.08222465	0.254
4079	0.59	2.05	A0A0G2JZH0	REVERSED Calcium-binding protein 39	1	null	null	null	null	null	null	null
4080	0.59	1.89	D3ZB65	BRCA2 and CDKN1A-interacting protein	1	null	null	null	null	null	null	null
4081	0.59	2.05	Q4KLY8		1	null	null	null	null	null	1.0565053	0.143
4082	0.59	0.96	M0R7E2	Phospholipid-transporting ATPase	1	1.26	1.07	1.21	1.21	1.57	0.96848392	0.0285
4083	0.59	5.37	A0JPP1	Dr1-associated corepressor	1	1.72	0.80	1.12	0.97	0.88	1.07326029	0.114
4084	0.58	2.43	P56522	REVERSED NADPH:adrenodoxin oxidoreductase, mitochondrial	2	null	null	null	null	null	null	null
4085	0.58	1.49	D3ZQE8	Exportin 5	2	1.05	1.32	1.10	0.97	0.97	null	null
4086	0.58	2.15	B4F762	Fanconi anemia group C protein homolog	2	null	null	null	null	null	0.98712179	0.706
4087	0.58	6.34	M0R7V1	REVERSED Death effector domain-containing 2	2	0.79	0.79	0.86	1.42	0.70	0.94369986	0.0522
4088	0.58	1.80	D4AC89	BTB domain-containing 11	2	1.14	0.79	0.89	0.68	0.86	0.98541272	0.377
4089	0.58	1.68	Q8K3Y6	Zinc finger CCCH-type antiviral protein 1	1	0.98	0.77	0.91	0.84	1.13	0.98091498	0.0973
4090	0.58	1.99	A0A0G2JT43	Solute carrier family 2, facilitated glucose transporter member 5	1	1.33	0.96	1.03	1.02	0.95	1.00772361	0.733
4091	0.58	1.64	B2GV39	Mannosyl (alpha-1,3-)-glycoprotein beta-1,4-N-acetylglucosaminyltransferase, isozyme B	1	0.95	0.83	0.83	0.74	0.88	1.01769129	0.459
4092	0.58	3.62	Q5M9F0	UPF0705 protein C11orf49 homolog	1	0.90	0.87	0.88	0.75	0.77	0.90250073	0.0339
4093	0.58	2.01	A0A0G2K6M8	DEP domain-containing 5	2	1.41	0.54	0.90	0.65	0.88	0.99702391	0.856
4094	0.58	1.89	A0A0G2JZN2	Netrin receptor UNC5A	1	0.83	1.26	0.95	1.12	0.87	0.9666061	0.539
4095	0.58	2.40	D3ZCB9	Family with sequence similarity 92, member B	1	null	null	null	null	null	1.7666301	0.03615
4096	0.57	10.75	Q5U1Z0	Rab3 GTPase-activating protein non-catalytic subunit	14	0.69	0.84	0.91	0.71	0.89	0.99327837	0.341
4097	0.57	3.62	A0A0G2JW52	Protein arginine N-methyltransferase 3	2	0.50	0.56	0.58	0.97	0.68	0.99786738	0.939
4098	0.57	13.41	D3ZYH3	Nudix (nucleoside diphosphate-linked moiety X)-type motif 10	2	1.26	0.44	1.56	1.09	1.27	1.01234427	0.953
4099	0.57	5.02	Q5M887	Trafficking protein particle complex subunit 13	1	1.43	1.32	1.22	1.10	1.12	0.9802353	0.633
4100	0.57	8.23	D4A3I4	Transcription factor BTF3	2	0.72	0.86	0.90	0.83	0.78	1.08598186	0.00461
4101	0.57	2.11	P20272	Cannabinoid receptor 1	1	0.81	1.25	1.06	1.10	1.12	1.11265012	0.0053
4102	0.57	1.65	Q7TP42	Ab2-292	1	0.91	0.99	0.99	1.02	0.94	1.04181564	0.263
4103	0.57	1.31	Q9WVR6	Large neutral amino acids transporter small subunit 2	1	0.80	0.76	0.84	0.97	0.81	0.98732708	0.886
4104	0.57	4.49	D3ZC87	REVERSED MAF bZIP transcription factor F	1	0.52	0.81	0.97	0.71	0.66	null	null
4105	0.57	6.09	G3V7R5	REVERSED Glutaredoxin-like protein	1	null	null	null	null	null	null	null
4106	0.56	0.00	F2Z3T0	Parafibromin	0	0.95	0.99	0.88	0.78	0.85	1.05277709	0.144
4107	0.56	3.22	G3V969	Tcfcp2 protein	1	0.55	0.52	0.45	0.57	0.47	1.06311672	0.215
4108	0.56	1.95	D4A7U1	Zyxin	1	1.07	1.16	0.95	1.15	1.11	0.88209137	0.0911
4109	0.55	3.09	A0A0G2K6T0	RCG27287, isoform CRA_b	5	1.06	0.95	1.12	1.22	1.42	0.99164799	0.674
4110	0.55	12.95	P04218		4	0.87	0.97	1.01	1.11	0.90	0.90626194	0.215
4111	0.55	7.07	F1LQH9	Bcl2-associated athanogene	2	0.74	0.90	0.77	0.79	0.67	0.97332937	0.594
4112	0.55	3.07	Q63713	Ras GTPase-activating protein 2	2	1.56	0.92	0.64	1.00	0.75	0.98986247	0.644
4113	0.55	3.39	Q6AYG2	DnaJ (Hsp40) homolog, subfamily C, member 28	1	1.04	0.93	1.12	0.97	0.95	1.01853814	0.654

4114	0.55	1.74	Q9ES39	REVERSED Nuclear distribution protein nudE homolog 1	1	null	null	null	null	null	null	null
4115	0.55	2.00	P63251	G protein-activated inward rectifier potassium channel	1	0.94	1.11	0.83	0.81	0.97	1.0807254	0.156
4116	0.55	6.19	Q4KLK9	RNA polymerase II subunit A C-terminal domain phosphatase SSU72	1	0.83	0.60	1.60	2.40	1.75	1.06488674	0.105
4117	0.55	13.19	Q5XFV6	Mitochondrial ribosomal protein L34	1	0.68	0.60	0.94	0.88	0.65	0.94330747	0.805
4118	0.54	1.04	A0A0G2K8N9	NIMA-related kinase 9	1	1.17	1.85	1.58	1.06	1.45	1.15668818	0.0856
4119	0.54	0.00	Q5FVQ2	REVERSED Peter pan homolog (Drosophila)	0	2.11	1.47	1.54	1.58	1.24	null	null
4120	0.54	2.03	F1LQ27	Family with sequence similarity 98, member C	1	1.10	1.09	0.96	1.31	1.47	1.03605458	0.202
4121	0.54	7.46	D4A193	Receptor expression-enhancing protein	1	1.31	1.27	1.18	0.89	1.14	0.98118698	0.741
4122	0.54	2.36	A0A0G2JVB1	SIX homeobox 2	1	0.89	1.50	1.12	0.90	1.21	0.98234385	0.757
4123	0.53	1.71	D3ZTG3	Family with sequence similarity 171, member B	1	1.04	0.97	1.01	1.37	1.05	1.00793318	0.802
4124	0.53	0.00	D3ZWT6	RCG31450	0	1.33	1.11	0.80	1.05	0.90	0.97908092	0.596
4125	0.53	3.98	Q5M8C7	Thioredoxin domain containing 9	1	0.61	0.86	0.94	0.91	0.77	1.1352421	0.027
4126	0.53	7.19	M0R3K2	Glutamyl-tRNA(Gln) amidotransferase subunit C, mitochondrial	1	0.64	0.79	1.41	0.92	0.95	1.04261029	0.696
4127	0.53	1.18	G3V8I7	REVERSED SEL1 domain containing protein RGD735029, isoform	1	null	null	null	null	null	null	null
4128	0.53	2.42	M0R9L2	Ciliary neurotrophic factor receptor subunit alpha	1	2.49	1.71	1.12	0.62	0.81	0.87417862	0.00622
4129	0.52	0.93	A0A0G2K7F1	REVERSED	1	null	null	null	null	null	null	null
4130	0.52	1.28	B1WC52	REVERSED RCG25586, isoform CRA_a	1	null	null	null	null	null	null	null
4131	0.52	0.62	Q0V8T5	REVERSED Contactin-associated protein like 5-2	1	null	null	null	null	null	null	null
4132	0.52	0.00	Q5XIP6	Flap endonuclease 1	0	1.16	0.82	1.17	1.10	0.81	1.01360813	0.652
4133	0.52	4.40	Q6PCT8	Succinate dehydrogenase [ubiquinone] cytochrome b small subunit, mitochondrial	1	1.54	1.18	0.96	1.24	1.38	0.91003882	0.00844
4134	0.52	0.86	H9N1L3	B-cell CLL/lymphoma 11B	1	null	null	null	null	null	0.98999971	0.56
4135	0.51	15.11	M0R544	Glucosidase, alpha, acid, isoform CRA_a	9	0.54	0.49	0.54	0.55	0.87	0.97393676	0.54
4136	0.51	1.79	Q9JIH7	Serine/threonine-protein kinase WNK1	4	0.94	0.85	0.92	0.91	0.82	1.02690306	0.256
4137	0.51	2.47	F1LX74	Transmembrane protein	2	1.10	1.14	1.12	0.95	1.09	1.08673486	0.219
4138	0.51	1.31	D3Z8N7	F-box protein 15	1	null	null	null	null	null	1.02115368	0.0494
4139	0.51	0.00	Q32KJ5	N-acetylglucosamine-6-sulfatase	0	1.16	1.12	1.49	1.21	1.24	1.01360813	0.584
4140	0.51	2.36	F1LNL9	Nucleoside diphosphate kinase 7	1	1.13	0.94	0.69	0.59	0.90	0.94796126	0.41
4141	0.51	2.27	F1M790	Prostaglandin F2 receptor negative regulator	1	0.89	1.02	0.72	1.20	0.96	1.01599972	0.287
4142	0.51	1.06	Q4V7B1	REVERSED Uncharacterized protein	1	null	null	null	null	null	null	null
4143	0.51	2.14	Q80WD1	Reticulon-4 receptor-like 2	1	0.90	0.80	0.52	0.71	0.79	0.87843047	0.393
4144	0.51	1.59	Q8K4Q9	Gap junction alpha-8	1	0.41	0.72	0.68	0.54	0.47	0.85618829	0.0499
4145	0.5	1.75	Q68SB1	Double-stranded RNA-binding protein Staufen homolog 2	1	1.22	1.41	1.60	1.67	1.42	1.09353546	0.0611
4146	0.5	1.03	G3V777	RCG31239, isoform CRA_a	1	null	null	null	null	null	1.00279729	0.925
4147	0.5	1.49	Q5PQM8	REVERSED Protein FAM214B	1	1.45	1.02	1.34	1.16	1.58	null	null
4148	0.5	1.52	Q2I6B1	V-type proton ATPase subunit a	1	0.91	1.14	1.11	1.22	1.05	0.941087	0.0354
4149	0.5	0.79	G3V6S2	REVERSED Aconitate hydratase	1	null	null	null	null	null	null	null
4150	0.5	3.81	F1LYS7	Sarcoglycan, delta	1	0.95	0.93	0.89	1.03	0.97	1.03979563	0.496
4151	0.5	0.00	A0A0G2JVT3	PILR alpha-associated neural protein	0	1.37	0.89	0.54	0.56	0.55	1.01755022	0.842
4152	0.5	2.42	M0RAS3	REVERSED Coiled-coil domain-containing 137	1	null	null	null	null	null	null	null
4153	0.49	3.19	A1A5P4	Kinesin-like protein	3	null	null	null	null	null	1.03354414	0.325
4154	0.49	4.71	A0A0G2JSR4	Signal transducer and activator of transcription	4	0.95	0.84	0.85	0.96	0.96	1.04275483	0.201
4155	0.49	3.01	F1MAF5	PCI domain-containing 2	1	0.95	1.07	0.99	1.00	0.98	1.03018254	0.388
4156	0.49	1.15	Q6QI09	REVERSED ATP synthase subunit gamma, mitochondrial	1	null	null	null	null	null	null	null
4157	0.49	1.99	P15887	REVERSED S-arrestin	1	null	null	null	null	null	null	null

4158	0.48	2.54	Q5RKH0	Putative oxidoreductase GLYR1	2	0.63	0.92	0.50	0.69	0.87	0.9713075	0.538
4159	0.48	1.21	A0A0A0MXY[illegible]	E3 ubiquitin-protein ligase TRIP12	2	1.31	1.15	1.01	0.97	0.90	1.02668955	0.56
4160	0.48	0.58	G3V7U1	REVERSED Glutamate receptor, metabotropic 1, isoform CRA_b	1	null	null	null	null	null	null	null
4161	0.48	0.96	D3ZJR6	Smith-Magenis syndrome chromosome region, candidate 8	1	0.88	0.91	0.97	0.83	0.88	1.02242853	0.694
4162	0.48	1.82	P35355	Prostaglandin G/H synthase	1	0.63	0.74	0.88	1.00	0.56	1.19664896	0.0758
4163	0.48	0.00	Q66H68	REVERSED RNA-binding protein 47	0	null	null	null	null	null	null	null
4164	0.48	1.46	A0A0G2JTI7	Pre-mRNA-processing factor 3	1	0.67	0.76	0.90	0.77	1.00	1.0546761	0.125
4165	0.48	0.00	D3ZL05	Sialic acid-binding Ig-like lectin 15	0	null	null	null	null	null	0.99274838	0.76
4166	0.48	0.00	G3V942	B2 bradykinin receptor	0	0.56	0.90	0.96	1.24	0.42	1.01988042	0.662
4167	0.48	7.41	Q498D8	Ring-box 1	1	1.10	0.95	1.11	1.05	1.21	0.96493255	0.634
4168	0	1.68	P03957	Stromelysin-1	1	null	null	null	null	null	null	null
4169	0.04	2.64	A0A0G2K5N[illegible]	ArfGAP with coiled-coil, ankyrin repeat and PH domains 3	2	null	null	null	null	null	null	null
4170	0.47	0.00	Q63488	Sodium-dependent phosphate transporter 2	0	0.95	1.02	0.99	1.11	1.00	0.90375273	0.0453
4171	0	9.77	Q5FVH7	Male-enhanced antigen 1	0	null	null	null	null	null	null	null
4172	0.47	0.87	F7FNT9	Zinc finger protein 709	1	null	null	null	null	null	1.11496622	0.0666
4173	0.47	1.91	B4F7F1	Cleavage stimulation factor subunit 2	1	null	null	null	null	null	1.02462784	0.241
